# Supplementary material for: Soy-Derived Equol Induces Antioxidant Activity in Zebrafish in an Nrf2-Independent Manner
Source: Int J Mol Sci. 2022 May 8;23(9):5243. doi: 10.3390/ijms23095243 (PMC9105299; doi:10.3390/ijms23095243)
Supplement: Supplementary file 1 [file ijms-23-05243-s001.zip › ijms22.watanabe.TableS1.pdf]

Table S1. Fold change in gene expression by equol treatment.

Genes	Equol-treated vs untreated in wild-type larvae	Equol-treated vs untreated in *nfe2l2a*^{fh318} larvae
egr2a_2	4.615384615	1.697674419
immp1l	4.272727273	0.723404255
fosl1a	3.630434783	1.960526316
btg2	3.557291667	1.666666667
gsta.1	3.468235294	1.963636364
arhgap39	3.333333333	0.5
rgs2	3.028571429	2.791666667
fosab_1	2.966507177	0.828101644
si:ch73-335l21.4	2.95890411	1.265625
si:dkey-224k5.13	2.823529412	1.470588235
hspb9	2.730519481	3.135371179
sgk1	2.719587629	1.83032491
jdp2b	2.719512195	1.108225108
si:ch211-215p11.5	2.615384615	1.363636364
si:dkey-7i4.12	2.6	1
CR855274.1	2.547169811	1.633757962
enpp7.1	2.545454545	3.526315789
si:dkey-14d8.23_1	2.545454545	4.153846154
fosaa	2.544303797	0.87628866
fosb	2.53125	1.968253968
epgn	2.509433962	3.058823529
si:ch73-54n14.2	2.5	1.411764706
avp	2.470588235	0.4
BX005085.3	2.466666667	2.125
igfbp1b	2.463414634	2.5
FO704777.1	2.461538462	1.7
csrnp1a	2.45631068	2.427083333
cebpb	2.421238938	1.379770992
DNAJB5 (1 of many)	2.420689655	0.817307692
si:dkey-259p7.1	2.411764706	1
egr3	2.405405405	2.256410256
si:dkey-43p13.5	2.363636364	0.8
zgc:198419	2.35359116	1.376044568
adamts1	2.3515625	1.570588235
si:ch211-130h14.6_2	2.333333333	2.708333333
5S_rRNA_693	2.3125	0.65625
dusp1	2.286021505	1.503793627
si:dkeyp-55h4.2	2.285714286	2.25
pnrc1	2.275	0.951612903
atoh1c	2.272727273	1.894736842
asmt	2.25	0.705882353
npas4a	2.243902439	0.309734513
si:ch73-249k16.4	2.238095238	0.826086957
nr4a1	2.23495702	1.820247934
smtlb	2.230769231	1.414893617
gadd45ba	2.217665615	1.765072765
nr4a3	2.214285714	1.557377049
si:ch211-227n13.3	2.2	1.6
OTUD1	2.192771084	1.802469136
egr1	2.173584906	1.071979434
socs3a_1	2.160493827	0.195555556
si:dkeyp-2e4.5	2.133333333	2.333333333
rgs13	2.130434783	1.90625
dusp2	2.113207547	1.426035503
si:dkey-162b3.5	2.106382979	1.298245614

cyp24a1	2.103286385	2.649350649
si:cabz01069015.1	2.1	1.111111111
si:dkey-29b5.3	2.1	1.926229508
si:dkey-79d12.5	2.077777778	4.24137931
ms4a17a.2	2.076923077	1.928571429
cyr61	2.075313808	1.246268657
tcap	2.072380952	1.939265537
slc25a38a	2.071428571	1.5
si:dkey-88n24.12	2.066666667	3.090909091
dusp5	2.064516129	1.689265537
rnf165b	2.052631579	0.566666667
egr4	2.044117647	1.328947368
pax8	2.043478261	0.323529412
histh1l	2.040633245	0.833909287
ets2	2.038834951	1.288389513
znf1163	2.037037037	1.125
cyr61l1	2	0.709677419
hsp70.1	2	0.733333333
MSC	2	0.791666667
ggcta	2	1.075757576
CU929391.1	2	1.363636364
CABZ01044960.1	2	1.4
helt	2	1.785714286
si:dkey-267n13.1	2	1.8125
ERRFI1 (1 of many)	2	2.007575758
si:dkey-242g16.2	2	3.539473684
junbb	1.997058824	0.658772874
f2rl1.2	1.974683544	2.475
mmp13a	1.970588235	1.142857143
rabgef1l	1.96	0.741935484
srsf3a_2	1.96	2.941176471
tmem14cb	1.958333333	1.035087719
arrdc3b	1.953525641	1.439849624
CU469570.2	1.942857143	0.662337662
ier2	1.941860465	1.292735043
GPR151	1.939393939	1.138888889
si:ch211-212k5.1	1.933333333	1.631578947
hs6st3a	1.928571429	0.75
si:dkey-108k21.21	1.928571429	1.5
si:ch211-163b2.2	1.918918919	1.081632653
znf512	1.9	1
si:dkey-17e16.17	1.894444444	1.235099338
bsx	1.888888889	1.894736842
gadd45bb	1.884615385	1.683168317
hamp	1.873015873	3.145833333
znf1161	1.869565217	1.789473684
si:ch211-266c8.1	1.866666667	0.826086957
cyp2u1	1.863636364	1.148148148
fut10	1.857142857	1.111111111
si:dkey-47k20.6	1.853658537	1.857142857
zgc:109934	1.849315068	1.647181628
mpg	1.846153846	0.9375
gip	1.846153846	1.466666667
spx	1.846153846	1.583333333
NR4A2 (1 of many)	1.84375	0.222222222
oxt	1.84375	0.24
zgc:175284	1.842105263	1.888888889
zgc:174259	1.842105263	2.590909091
rasd1	1.841791045	1.538461538
nfkbiaa	1.830827068	2.116618076
si:dkey-201c13.2	1.828571429	0.981481481

si:dkey-271j15.3	1.826086957	0.68
zgc:113274	1.823529412	1.6
si:ch211-87m7.3	1.822580645	1.4
rars2	1.821428571	0.861111111
kcnj1a.4	1.819148936	2.293103448
si:dkey-14d8.22	1.818181818	0.484848485
CHST8	1.818181818	0.64
zgc:152698	1.818181818	1.590909091
zgc:172215	1.813953488	1
zgc:153911	1.808510638	2.783783784
CABZ01068208.1	1.8	0.56
ODF3L2 (1 of many)	1.8	0.807692308
znf1171	1.8	2.111111111
ofd1	1.793103448	1.19047619
si:dkey-103i16.6	1.789473684	0.739130435
otc	1.787234043	1.272727273
he1a	1.785714286	0.113636364
aasdh	1.785714286	1.272727273
CABZ01041610.1	1.78125	0.282608696
cebpd	1.781094527	1.661966156
si:dkey-47k16.4_2	1.777777778	2.846153846
plek2	1.777777778	1.837837838
doc2d	1.775	0.452380952
erfl1	1.772727273	0.625
si:ch211-119d14.2	1.772727273	1.066666667
ppcdc	1.772727273	1.615384615
CABZ01064860.2	1.769230769	1.4
si:dkey-15h8.16	1.769230769	2.071428571
CABZ01012147.1	1.769230769	2.083333333
slc25a25a	1.765333333	1.273076923
si:ch211-114l13.11	1.765060241	1.788018433
Metazoa_SRP_38	1.76	0.9
si:ch211-158m24.12	1.76	1.18503937
RAP2A (1 of many)	1.759259259	0.78125
bcl6a	1.75308642	0.833333333
tmem187	1.75	0.868421053
pogza	1.75	0.903225806
zgc:152863	1.75	1.095238095
si:ch211-157e21.2	1.75	1.118421053
mepce	1.744680851	1.09375
btr04	1.740740741	1.80952381
crp4	1.739130435	1.053797468
si:ch211-71k24.7	1.736842105	0.511627907
vipb	1.734177215	0.928
TMEM125 (1 of many)	1.733333333	0.769230769
btr31	1.733333333	1.277777778
si:dkey-149m13.4	1.733333333	1.357142857
xkr5	1.727272727	0.857142857
fam188b2	1.727272727	0.928571429
sowahd	1.727272727	1.083333333
si:dkey-50i6.6	1.725806452	1.964912281
CABZ01083501.1	1.725490196	0.365384615
adgrb1b	1.71875	0.348837209
si:ch73-44m9.1	1.714285714	1.375
si:dkey-246j7.1	1.714285714	2
sik1	1.711934156	1.617586912
brd2a	1.71024735	1.487804878
LECT2 (1 of many)_1	1.709677419	1.625
catip	1.705882353	1.071428571
ccr9a	1.705882353	2.357142857
slc16a9a	1.7	0.788888889

znf653	1.7	0.842105263
zgc:162611	1.7	1.25
prdm12b	1.696428571	0.5
klf2a	1.696048632	1.5
WDR31	1.695652174	0.944444444
ldlrap1b	1.695652174	1
si:ch73-214d15.1	1.693333333	0.183333333
zgc:92749	1.692307692	0.483870968
mlnl	1.689655172	1.518518519
c1ql3b_2	1.682926829	0.388888889
mlc1	1.681818182	1.387096774
socs1a	1.679012346	1.339449541
PRTFDC1 (1 of many)	1.666666667	0.756097561
acyp1	1.666666667	0.836734694
wt1b	1.666666667	0.85
SIRT6	1.666666667	0.901785714
tor4aa_1	1.666666667	0.928571429
CABZ01087111.1	1.666666667	1.043478261
NMNAT3	1.666666667	1.285714286
hist2h3c	1.666666667	1.333333333
CABZ01021450.4	1.666666667	1.727272727
ropn1l	1.666666667	2
si:ch211-113a14.22	1.666666667	2.083333333
si:cabz01023255.2	1.666666667	2.5
actr3b	1.666666667	0.666666667
nme5	1.666666667	1.043478261
si:dkey-21o19.2	1.666666667	1.631578947
nelfa	1.661971831	0.6125
stk19	1.657142857	2.151515152
kcnj1a.3	1.653465347	2.651515152
hspb11	1.649350649	2.014388489
nfkbiab	1.648967552	1.43081761
si:ch211-209l18.8	1.646153846	1
si:dkey-66i24.8	1.636363636	0.764705882
VPS72 (1 of many)	1.631578947	1.2
cldnb	1.631100083	1.455399061
mettl21a	1.62962963	1.04
nab1b	1.62962963	1.055555556
ciartb	1.629268293	0.915555556
agtr1b	1.625	1.111111111
ca4c	1.625	2.391304348
kcnj1a.6	1.62195122	2.71875
GRM7	1.615384615	0.25
odf2b	1.615384615	1.4
mppe1	1.615384615	1.714285714
svilb	1.613636364	1.170731707
si:dkey-33c14.7	1.611111111	1.689655172
si:ch73-59f11.3	1.611111111	2.230769231
trib3	1.607287449	1.192857143
paqr4a	1.607142857	0.432432432
lyplal1	1.606060606	0.977272727
wars2	1.606060606	1.116666667
GRIK5 (1 of many)	1.605263158	0.514285714
mcoln1b	1.6	0.875
chd	1.6	0.904761905
zgc:158320	1.6	1.12
si:ch211-9d9.1	1.6	1.4
minpp1b	1.6	1.652173913
si:ch73-92e7.6	1.6	2.769230769
ctnnd2a	1.6	0.246575342
nkx2.4a	1.6	0.384615385

si:dkey-163f14.6	1.6	1
si:ch211-56a11.2	1.596330275	0.763736264
zgc:114175	1.594339623	0.4
agbl4	1.590909091	0.75
CU469093.1	1.589285714	1.025641026
si:dkey-28g23.6	1.588235294	0.95
zgc:112163	1.588235294	1.333333333
rbm41	1.588235294	2.272727273
znf1175	1.586206897	0.888888889
slc16a9b	1.585365854	0.886877828
bpifcl	1.583333333	0.733333333
si:dkeyp-68b7.7_2	1.583333333	0.814814815
si:dkey-71b5.3	1.583333333	1.677419355
upp2	1.583333333	5
fam120b	1.580645161	0.837837838
h1f0	1.578983249	0.942133443
si:ch211-59m15.1	1.578947368	1.147058824
si:dkey-85k7.7	1.576923077	1.951020408
cx32.2	1.571428571	1.916666667
si:dkey-221l4.10	1.571428571	1.535714286
si:ch73-141c7.1	1.569948187	1.489473684
ppp1r27a	1.569072165	1.148820327
si:ch211-186e20.6p	1.568965517	1.857142857
h1fx	1.568770227	1.164246824
irx4a	1.566666667	0.35
plekhn1	1.565217391	1.098360656
CAMSAP3 (1 of many)_1	1.56097561	0.208333333
wu:fj16a03	1.560818084	1.305324158
PCMTD2 (1 of many)	1.557692308	1
lhx8a	1.555555556	1.256410256
si:ch73-299h12.6	1.555555556	1.652173913
ccdc3a	1.555555556	0.586956522
lbh	1.5546875	1.456790123
myf6	1.553191489	2.515151515
zgc:152948	1.551724138	1.125
si:ch211-113a14.12	1.551724138	1.319148936
htr3a	1.55	0.545454545
znf1160	1.55	0.612903226
rtn4rl2a	1.548387097	0.384615385
sdr42e2	1.546153846	0.736607143
trim9	1.545454545	0.305084746
si:dkey-151m15.8	1.545454545	0.52
spata6	1.545454545	1
tlx3b	1.545454545	1.095238095
carm1l	1.545454545	1.2
si:dkey-248g15.2	1.545454545	1.608695652
fosl2	1.544642857	1.108108108
ece2a	1.541666667	1.277777778
dazap1	1.541666667	0.576470588
hsp70.3	1.538461538	0.509803922
nt5c1ab	1.538461538	0.518518519
med19b	1.538461538	1.130841121
si:ch211-224b1.5	1.538461538	1.454545455
BX004873.4	1.538461538	2.227272727
SH2B2	1.536585366	0.424242424
tmem260	1.535714286	0.6
U2_1	1.533333333	0.796296296
ppp1r3da	1.533333333	1.307692308
snx20	1.533333333	1.388888889
si:ch1073-365n21.1	1.531914894	1.725
si:dkey-84j12.1_1	1.53125	0.309090909

lmx1ba	1.53125	2.074074074
ccka	1.52991453	1.17721519
CABZ01071972.1	1.529411765	0.733333333
kcnmb2	1.529411765	1.023255814
klhl35	1.529411765	1.28
sesn2	1.527777778	1.25
si:dkey-57c15.11	1.527559055	0.839285714
si:ch1073-363c19.3	1.527272727	1.296875
si:ch211-197k17.3	1.525974026	1.175496689
si:ch1073-443n13.2	1.525	1.173076923
adcy8	1.524590164	0.408450704
si:ch73-248e17.1	1.523809524	0.716981132
fabp10a	1.523186683	0.974218572
cx44.1	1.52173913	0.888888889
csrnp1b	1.521604938	1.48048048
coa5	1.520900322	1.191374663
esrrgb	1.52	1.111111111
aplnr2	1.52	1.583333333
si:ch73-359m17.2	1.519230769	1.210526316
kcnj1a.2	1.516666667	1.227722772
f2rl1.1	1.516129032	1.153846154
drgx	1.515151515	0.741935484
myd88	1.514018692	2.008403361
CABZ01111953.1	1.513513514	0.703703704
MFSD4B	1.513513514	1
kirrel3a	1.512820513	0.512195122
ogfod2	1.512820513	2
ap4m1	1.507692308	1.171052632
nr4a2b	1.506493506	1.128571429
gadd45ab	1.502538071	1.864321608
hsp70l	1.5	0.538461538
dusp4	1.5	0.637931034
etaa1	1.5	0.947368421
wdpcp	1.5	1.133333333
renbp	1.5	1.971428571
foxb1a	1.5	0.318181818
mcm8	1.5	0.647058824
si:ch73-233f7.3	1.5	0.674418605
C7 (1 of many)	1.5	0.717391304
uts2b	1.5	0.8
syt15	1.5	0.9
BX663505.2	1.5	0.916666667
si:ch211-73m21.1	1.5	1.066666667
ptk2ba	1.5	1.142857143
si:dkey-221l4.12	1.5	1.151515152
angpt2a	1.5	1.2
erich3	1.5	1.9
dpyda.1	1.5	0.489795918
kcng4a	1.5	0.566666667
si:ch211-255f4.11	1.5	0.8125
lrrc3b	1.5	0.85
pus3	1.5	1.291666667
BX005085.2	1.5	1.384615385
si:ch73-80e5.4	1.5	1.916666667
si:dkey-30k6.7	1.5	2
bcl2l11	1.5	2.071428571
cdc42l2	1.5	2.9
her4.1	1.496240602	1.559701493
paics	1.495741056	0.739617666
anapc13	1.494382022	1.326829268
zmat5	1.491935484	1.465838509

arg2	1.491525424	1.934086629
tsc22d3	1.490865766	1.498489426
otpa	1.489675516	0.339393939
AC024175.12	1.488095238	0.8
socs9	1.487179487	0.705882353
rps6kl1	1.482758621	1
mknk2a	1.481927711	1.105769231
MALSU1	1.481927711	1.1328125
asic2	1.481481481	0.52
cish	1.480686695	1.40077821
jund	1.479768786	1.425373134
slc38a11	1.477272727	0.787878788
si:dkey-32e23.4	1.476923077	0.776119403
si:ch73-71d17.2	1.476190476	0.736842105
ppp1r16b	1.476190476	1.055555556
hdhd3	1.475	1.142857143
junba	1.473333333	0.540145985
frmd4bb	1.472727273	0.975609756
ddit4	1.472650771	1.569248826
si:ch1073-464p5.5	1.47080292	2.005050505
hs3st1	1.470588235	0.68
rbbp8	1.470588235	0.851851852
CR376723.1	1.470588235	1.260869565
BX511021.2	1.470588235	1.573170732
AP3B2	1.470588235	0.367521368
rnf114	1.468253968	1.28
gpr186	1.466666667	0.767857143
tekt3	1.466666667	1.4
znf1182	1.466666667	1.84
CU694219.1	1.466666667	0.787878788
zgc:162730	1.464803313	0.900864937
AC024175.9	1.464667214	0.603244838
lrrc32	1.464285714	0.948717949
ovca2	1.464285714	1.363636364
gstp1	1.464110485	1.391147053
arvcfa	1.462962963	0.320754717
rmnd1	1.4625	1.24137931
fabp1b.1	1.461553102	1.415071913
dkk1b	1.461538462	1.145454545
pqlc3	1.461538462	1.01754386
AC024175.1	1.461387862	0.984079913
cdkn1a	1.461038961	1.523529412
si:ch73-233f7.4	1.459459459	1.25
creg2	1.455813953	1.017667845
efna2a	1.454545455	0.515625
si:dkey-199m13.7	1.454545455	1.333333333
BX000703.1	1.454545455	1.416666667
tmem230a	1.454545455	2.416666667
st6galnac6	1.452380952	0.35483871
si:ch211-153a8.4	1.451612903	1.052631579
wdsub1	1.451612903	0.768595041
nab1a	1.450980392	1.131578947
snx10a	1.450549451	1.219512195
kdelc1	1.45045045	1.333333333
FO704584.1	1.45	1.27027027
b3galnt2	1.45	1.482758621
si:ch211-39i2.2	1.45	1.783333333
cdk19	1.448275862	0.5
si:ch211-240g9.1	1.448275862	0.608695652
nuak2	1.447368421	1.023255814
nln	1.447058824	1.364864865

si:dkey-174n20.1	1.445783133	0.865979381
znf1008	1.444444444	0.681818182
RHOBTB2 (1 of many)	1.444444444	0.722222222
soat1	1.444444444	0.769230769
aasdhppt	1.444444444	0.784810127
cyldb	1.442857143	2.357142857
nr1d2a	1.442185515	1.168119552
ap5m1	1.441860465	0.882352941
gpr37b	1.44	0.37704918
FRRS1L	1.44	0.716981132
si:dkey-155h10.4	1.44	0.866666667
si:dkey-74i1.3	1.44	1.966666667
NPAS3	1.43902439	0.311320755
rasl11b	1.4375	1.283464567
si:ch1073-80i24.1	1.4375	0.433333333
si:dkeyp-51f12.2	1.4375	0.55
zgc:92275	1.4375	0.869565217
pgbd4	1.4375	0.875
BX530018.2	1.4375	1.6
BX649453.2	1.4375	1.846153846
znf1162	1.434782609	2.363636364
sgsh	1.43373494	0.902912621
CABZ01057122.1	1.433333333	1.076923077
xxylt1	1.433333333	1.083333333
zgc:109986	1.433333333	1.75
rccd1	1.432432432	1.214285714
metap1d	1.432098765	1
zfand5b	1.43003413	1.25199362
sppl2	1.43	0.676923077
BX088711.1	1.428571429	0.590909091
xylt2	1.428571429	0.8
clcn6	1.428571429	0.870967742
znf1011_1	1.428571429	1
tpst2	1.428571429	1.416666667
CABZ01041812.1	1.428571429	1.677419355
znf1011_2	1.428571429	1.833333333
TCF24	1.428571429	2
CU467905.1	1.426470588	4.532258065
si:ch211-256e16.11_2	1.425	1.033898305
nr1d1	1.424607961	1.107235142
serpine1	1.424528302	1.355140187
camta2	1.424242424	0.826086957
abca1a	1.423963134	0.818181818
si:ch211-210k13.3	1.423076923	1.179487179
zgc:153116	1.423076923	2.384615385
cpne4b	1.421052632	0.578947368
wnt7bb	1.421052632	0.696969697
si:ch211-106k21.5	1.421052632	1.692307692
si:dkey-7i4.9	1.421052632	3
zgc:92066	1.420093701	1.546777547
naglu	1.419354839	0.785714286
znf1134_2	1.419354839	1.8
rhbdd2	1.418604651	1.066666667
BX640518.1	1.418181818	1.466666667
slc13a2	1.416762342	1.103448276
cluhb	1.416666667	0.666666667
si:dkeyp-121d2.7	1.416666667	0.857142857
cyb561	1.416666667	0.916666667
si:ch211-268b13.5	1.416666667	0.916666667
tmem151ba	1.416666667	0.96969697
zgc:153352	1.416666667	1

zgc:136963	1.416666667	1.117647059
CABZ01075125.2	1.416666667	1.266666667
si:ch211-193l2.4	1.416666667	1.434782609
gtpbp3	1.416666667	1.461538462
ccdc14	1.416666667	1.727272727
hist2h2l	1.416555407	1.21704918
fbxw8	1.415584416	0.965909091
ube2o	1.415584416	1.057692308
bhlhe41	1.414201183	1.26006192
si:ch73-299h12.3	1.413043478	1.896551724
ipo8	1.412371134	1.151162791
ksr2	1.411764706	0.384615385
si:ch73-233f7.5	1.411764706	0.625
CABZ01041223.1	1.411764706	0.6875
si:dkeyp-79b7.13	1.411764706	0.916666667
cabz01076234.2	1.411764706	1.25
BX649620.1	1.411764706	1.666666667
CABZ01079870.1	1.411764706	1.7
lins1	1.411764706	2.076923077
pik3ip1	1.411214953	1.390510949
jazf1b	1.411111111	0.80952381
klf4	1.410958904	1.935897436
exd2	1.409090909	0.833333333
aptx	1.409090909	1.3
si:dkey-23n7.10	1.409090909	1.306122449
crp	1.409090909	1.341637011
si:dkey-57k2.6	1.409090909	1.157894737
lrrc61	1.409090909	1.310344828
si:ch211-114l13.1	1.409090909	1.5
zgc:171490	1.407407407	1.111111111
mest	1.406976744	1.386363636
pfkfb3	1.406417112	1.224770642
ucp1	1.403988183	1.535234899
TMEM42 (1 of many)_1	1.403846154	1.241935484
zgc:153952	1.403508772	1.166666667
EARS2	1.402777778	0.95890411
adam10b	1.402597403	0.637209302
ttc9b	1.401785714	0.480519481
txnipa	1.400692477	1.79281768
ttll7	1.4	0.26
pcsk9	1.4	0.6
fam131c	1.4	0.7
C1QTNF4 (1 of many)	1.4	0.744680851
mrap2a	1.4	1.027027027
galn	1.4	1.345454545
nkx3-1	1.4	1.5
dnajb5	1.4	1.5
tph2	1.4	1.542857143
CABZ01074203.1	1.4	0.454545455
si:ch211-284o19.8	1.4	0.619047619
BX663610.1	1.4	0.833333333
cx28.8	1.4	0.833333333
DXO	1.4	1
zdhhc3b	1.4	1.020833333
alkbh2	1.4	1.054054054
si:ch211-250n8.1	1.4	1.172413793
CABZ01054392.1	1.4	1.545454545
si:ch73-106l15.4	1.4	1.678571429
zgc:110340	1.398457584	1.523595506
fam160b1	1.398373984	1.053846154
si:ch211-276i12.11	1.397727273	0.917695473

crp3	1.397212544	1.055555556
faima	1.396946565	1.568807339
ptgs2b	1.396946565	3.1125
parp12b	1.396551724	1.06557377
pla2g4aa	1.396551724	1.176470588
actr5	1.396226415	1.125
si:ch211-199f5.1	1.394736842	0.361702128
slc31a2	1.393939394	2.222222222
si:dkeyp-1h4.9	1.393442623	0.571428571
tal2	1.393063584	0.611398964
ppil1	1.392857143	1.219330855
phlda2	1.392097264	1.603399433
nr1d4b	1.391304348	0.625
sdccag8	1.391304348	1.035714286
slc35a5	1.391304348	1.166666667
zgc:101130	1.390243902	0.939393939
ylpm1	1.389830508	0.794392523
hal	1.38961039	0.680709534
zgc:109889	1.389312977	0.373913043
fam134a	1.388888889	1.034482759
si:ch211-203h15.7	1.388888889	1.048780488
si:dkey-10p5.7	1.388888889	1.105263158
sv2	1.388888889	1.15
tex264b	1.388888889	1.25
znf1165	1.388888889	1.454545455
wbscr27	1.388888889	1.75
mrps18c	1.388679245	1.269784173
tdp2b	1.387755102	1.446808511
unc5db	1.387096774	0.52
specc1lb	1.387096774	0.75862069
AC024175.13	1.386896685	0.963559546
sumf2	1.386666667	1
si:ch211-154a22.8	1.386363636	1.8
si:dkey-53i3.1	1.386363636	2.078947368
CABZ01066482.1	1.385964912	0.899408284
nsmce2	1.385964912	1.421875
zgc:162945	1.385542169	1.218390805
arrdc2	1.384831461	1.697819315
CR352258.2	1.384615385	0.780487805
cks2	1.384615385	1.942857143
si:ch211-194p6.12	1.384615385	0.625
si:ch211-258f14.2	1.384615385	0.777777778
si:dkey-4e4.1_2	1.384615385	1
FQ377903.2	1.384615385	1.090909091
tekt4	1.384615385	1.117647059
znf1116	1.384615385	1.137931034
crfb2	1.384615385	1.307692308
rad51ap1	1.384615385	1.4
calhm3	1.384615385	2.266666667
vps50_1	1.384615385	2.7
zgc:153383	1.382352941	1
cbx6b	1.381818182	0.780487805
arl5c	1.38125	1.489177489
slx4_1	1.380952381	0.7
stxbp6l	1.380952381	1.285714286
htatip2	1.38	1.431578947
clk2b	1.378378378	0.709090909
CABZ01084612.1	1.377777778	1.511111111
MTERF4	1.37755102	1.181818182
si:ch211-34m5.2p	1.377358491	0.93125
nub1	1.376623377	1.151898734

uchl1	1.376033058	1.179310345
si:dkeyp-86d6.2	1.375	0.454545455
MARCH4 (1 of many)	1.375	0.6875
ARL3 (1 of many)	1.375	0.710526316
FUT8 (1 of many)	1.375	0.769230769
si:dkey-7i4.16	1.375	0.888888889
snai1b	1.375	0.906976744
si:dkey-188i13.7	1.375	0.925925926
pusl1	1.375	0.944444444
rpgrip1l	1.375	1
slc1a8b	1.375	1.195652174
si:ch211-74m13.1_1	1.375	1.222222222
si:ch211-147d7.5	1.375	1.388888889
znf1002	1.375	1.4
fn3krp	1.375	1.444444444
zgc:173577	1.375	2
kcnn1a	1.373333333	0.533333333
tmem68_1	1.37254902	1.320754717
wt1a	1.371428571	0.659090909
TBC1D13	1.371428571	1.212121212
zgc:194627	1.370967742	1.101010101
nfe2l1a	1.370967742	1.892307692
jdp2a	1.37037037	0.742857143
CABZ01084613.1	1.37037037	1.035714286
si:dkey-77f5.4	1.37037037	1.615384615
si:ch211-101l18.8	1.37037037	2.421052632
capsla	1.369565217	0.875
irs2b	1.369565217	0.843478261
midn	1.368932039	1.254587156
sec23b	1.368534483	1.641891892
ttbk1b	1.368421053	0.627906977
gsx1	1.368421053	0.633802817
znf1169	1.368421053	1.3125
dfna5b	1.368421053	1.340909091
zgc:112148	1.367088608	0.990990991
prcc	1.366666667	0.75862069
si:ch211-59d17.3	1.366666667	0.407407407
mt2	1.366438356	1.436027714
pcdh1a4	1.366071429	0.505747126
cers4a	1.366071429	0.602150538
pigw	1.365853659	1
spata7	1.365853659	1.14
fggy	1.365517241	0.758865248
ctgfa	1.364864865	0.825581395
FLRT2 (1 of many)	1.363636364	0.457142857
CABZ01112210.1	1.363636364	1
erg	1.363636364	1.071428571
terf1	1.363636364	1.7
si:dkey-165a24.10	1.363636364	0.786259542
BX510922.1	1.363636364	0.9375
plcxd2	1.363636364	1
creb5b	1.363636364	1.25
DDO	1.363636364	1.642857143
sult1st5	1.363636364	2
si:ch211-270n8.3	1.363636364	2.705882353
syn2b	1.362962963	0.192982456
sat1a.2	1.362944162	1.852216749
ptprr	1.362318841	0.766233766
aak1b	1.361111111	0.545454545
si:dkey-90l8.3	1.361111111	1
asic1b	1.36	0.317073171

si:ch211-207m11.3	1.36	0.65
ube2j2	1.36	1
si:dkey-30c15.2	1.36	1.76
EHMT2	1.359375	0.678571429
snap47	1.358974359	1.023622047
hbp1	1.358870968	1.06741573
cdh7	1.358208955	0.298245614
pdlim7	1.357894737	0.947859922
plk3	1.357487923	1.313084112
slain1a	1.357142857	0.436363636
cd226	1.357142857	0.678571429
zgc:195245	1.357142857	0.685714286
aqp3b	1.357142857	1
oxld1	1.357142857	2.117647059
glceb	1.355769231	0.859813084
plk2b	1.355421687	1.562962963
cetn4	1.355263158	1.469387755
xkr7	1.35483871	0.384615385
si:dkey-6i22.5	1.35443038	0.923809524
ndufaf6	1.354330709	0.986206897
prdm1b_1	1.354166667	0.896551724
zgc:65997	1.354166667	1.357843137
trim36	1.352941176	0.549019608
TUBGCP6	1.352941176	0.625
CR382372.2	1.352941176	1.235294118
si:cabz01021426.2	1.352941176	1.857142857
map1lc3a	1.352490421	1.010909091
prelid3b	1.352173913	1.380588877
rpp38	1.352112676	0.73255814
RNF182 (1 of many)_1	1.351851852	0.682926829
pld3	1.351851852	1.06122449
elfn1b	1.351351351	0.807692308
march7_3	1.350553506	1.020477816
psmg3	1.350393701	1.081395349
sorbs2a	1.35	0.476190476
ddx43	1.35	0.976190476
si:ch1073-224n8.1	1.35	0.754716981
zgc:92249	1.35	1.904761905
braf	1.349514563	0.505050505
gata2a	1.34939759	0.689655172
stra13	1.349056604	0.807453416
spg11	1.348837209	0.829787234
auts2a	1.348484848	0.562874251
ftr85	1.347826087	1.411764706
ercc6l2	1.347826087	1.842105263
GRAMD1B (1 of many)	1.346153846	0.947368421
trpv6	1.345238095	1.754716981
cda	1.345132743	2.438596491
tctn1	1.344827586	1.142857143
letmd1	1.344827586	1.357142857
cyp2aa6	1.344262295	1
ap4e1	1.34375	0.866666667
hexim1	1.343570058	1.018711019
si:ch211-153b23.4	1.343137255	1.224806202
si:ch73-233f7.6	1.342857143	0.927272727
ppp1r15b	1.342857143	1.212765957
CU929391.2	1.342857143	1.548387097
ptgs2a	1.342857143	1.672413793
per1a	1.342657343	0.963768116
pcnx	1.341772152	0.763888889
cspp1b	1.340909091	1.388888889

pigl	1.340909091	1.574468085
tab3	1.340659341	0.595959596
si:dkey-251i10.2	1.340350877	1.045372051
ctu1	1.34	0.803921569
trim16	1.339805825	1.300884956
kcnc1b	1.338983051	0.321428571
si:dkey-234i14.2	1.338235294	0.896103896
scrn3	1.3375	0.715789474
nrg3b	1.337349398	0.473684211
nrxn2b	1.336734694	0.22972973
mid1ip1a	1.336492891	1.814580032
cipca	1.336448598	1.257731959
ing5a	1.336	1.559055118
hbegfa	1.33447099	1.856573705
PRRC2B	1.334269663	0.713498623
slc1a2b	1.333553938	0.193804035
slc45a1	1.333333333	0.420289855
si:ch211-256e16.3	1.333333333	0.6875
znf1021_1	1.333333333	0.714285714
zgc:110852	1.333333333	0.952380952
zranb3	1.333333333	1
otud3	1.333333333	1.064102564
zgc:113209	1.333333333	1.064516129
KDELR3 (1 of many)	1.333333333	1.085106383
si:dkey-66i24.7	1.333333333	1.101694915
si:ch211-157e21.22	1.333333333	1.111111111
CABZ01043646.1	1.333333333	1.333333333
si:dkey-207m2.4_1	1.333333333	1.523809524
cfap52	1.333333333	1.615384615
slit1b	1.333333333	0.276923077
evx2	1.333333333	0.342105263
tlr7_1	1.333333333	0.418181818
si:dkey-215k6.1	1.333333333	0.470588235
si:ch211-165g14.3	1.333333333	0.571428571
zgc:110697	1.333333333	0.782608696
armc3	1.333333333	0.8125
zgc:163083	1.333333333	0.837837838
si:ch211-133l5.5	1.333333333	0.864864865
PDE2A	1.333333333	0.928571429
zgc:193726	1.333333333	0.960526316
wnt2bb	1.333333333	1
zgc:153595	1.333333333	1.24137931
ankrd52b	1.333333333	1.277777778
lipt2	1.333333333	1.391304348
si:dkeyp-2e4.6	1.333333333	1.533333333
si:dkeyp-91h6.2	1.333333333	1.631578947
lyve1a	1.333333333	1.642857143
ccdc129	1.333333333	1.714285714
si:dkeyp-79b7.6	1.333333333	1.97826087
si:dkey-165o8.2	1.333333333	2.055555556
usb1	1.333333333	2.058823529
agrp	1.333333333	2.3
chac1	1.332381292	1.48869964
lef1	1.331360947	0.606936416
dtd1	1.330645161	1.172661871
dnph1	1.32967033	0.603448276
CABZ01067086.1	1.329113924	0.695121951
pcnxl4	1.328358209	0.615384615
si:ch211-14a17.11	1.328125	0.93902439
gcnt4a	1.328125	2.131147541
cldn11a	1.327922078	1.549071618

kcnk5b	1.327868852	1.574074074
r3hcc1	1.327868852	2.075
hddc2	1.327683616	1.662721893
si: zfos-932h1.3	1.327586207	0.648648649
tada2a	1.327272727	0.795275591
atp9b	1.327272727	0.880952381
orai1b	1.327272727	1.086956522
jmjd4_1	1.327272727	1.387096774
marco	1.326923077	1.232876712
pkp2	1.326086957	0.979166667
sox14	1.325925926	0.526666667
rnf4	1.325301205	1.746478873
emc4	1.324786325	0.983739837
zgc:174224_1	1.324324324	1.285714286
si:ch211-181d7.1_2	1.324324324	1.285714286
TSTD1	1.324324324	1.649484536
si:ch211-284b7.3	1.323383085	0.912408759
pbrm1	1.323076923	0.54
vhl	1.322834646	1.634615385
CR628323.1	1.322580645	0.863636364
si:ch73-138e16.8	1.322580645	1.022222222
ptprt_1	1.322222222	0.351851852
si:ch211-106h11.3	1.322222222	0.686046512
klf5b	1.321428571	0.483870968
chrna3	1.321428571	1.4
cx34.4	1.321428571	1.465517241
zgc:113030	1.321428571	1.692307692
nf2a	1.321428571	1.727272727
pou4f1	1.320754717	0.333333333
irs4a	1.320754717	0.431818182
wdr47a	1.320754717	0.593406593
cyp2aa8	1.320450886	0.850385039
CABZ01115881.1	1.32	1.087719298
CABZ01078427.1	1.32	1.85
otud5a	1.31981982	0.756410256
PRKAR2B	1.319444444	0.19047619
phactr3b	1.319327731	0.768
b3gnt7	1.319148936	1.061946903
ssbp2	1.318681319	0.8
kif1ab	1.318181818	0.261538462
tbc1d16	1.318181818	0.782608696
manba	1.318181818	1.380952381
METTL21C (1 of many)_2	1.318181818	1.413793103
ved	1.318181818	1.764705882
zgc:158398	1.317948718	1.035353535
dnajc14	1.317073171	0.87037037
si:dkeyp-68b7.7_1	1.317073171	1.422222222
brinp1	1.316666667	0.229166667
wdr92	1.316455696	1.610169492
pank2	1.316091954	1.04964539
cep112	1.315789474	0.866666667
rnf152	1.315789474	0.907692308
im:7152348	1.315789474	0.967741935
foxi2	1.315789474	1.153846154
si:ch211-235i11.7	1.315789474	1.571428571
hyal1	1.315789474	1.583333333
znf1106	1.315789474	2.25
lrrc4.2	1.314814815	0.347826087
josd2	1.314516129	1.090225564
gsto2	1.314387211	1.497050147
unk	1.314285714	0.891891892

top3a	1.314285714	0.90625
eaf1	1.314102564	1.202531646
si:dkey-175g6.2	1.313559322	0.663043478
gpx4a	1.313220505	1.164470888
AC024175.18	1.313151927	0.907657993
mknk2b	1.312787489	1.480440098
si:ch211-199g17.9	1.3125	0.705882353
pgap1	1.3125	0.84
TDRD7 (1 of many)	1.3125	0.888888889
RDH13 (1 of many)_2	1.3125	1.1875
si:dkey-5n18.1	1.311926606	0.934210526
odc1	1.3113879	0.835036496
si:dkey-95p16.2	1.311377246	1.316666667
ythdc1	1.311046512	0.944805195
aifm4	1.310606061	2.107692308
znhit6	1.310344828	1.050420168
syn3	1.310344828	0.379310345
kcns3a	1.310344828	0.690909091
tmem241	1.310344828	1.608695652
chmp4ba_2	1.310344828	2.25
zgc:101846	1.310114857	1.089416914
kif1c	1.30952381	0.788461538
CABZ01044099.1	1.30952381	0.805194805
ncdn	1.30952381	0.850574713
si:ch73-15b2.5	1.309090909	0.890410959
apool	1.308695652	0.856115108
gins2	1.308641975	0.925925926
WHAMM	1.307692308	0.961538462
hapln2	1.307692308	0.916666667
nudt17	1.307692308	0.943089431
si:dkey-51d8.6	1.307692308	1
si:dkeyp-53d3.3	1.307692308	1.529411765
znf1122	1.307692308	1.631578947
si:dkey-225f5.5	1.307692308	2.357142857
FEZ2 (1 of many)_2	1.306818182	1.454022989
lrrc4bb	1.306569343	0.310344828
spry1	1.305882353	0.666666667
COX5B (1 of many)_1	1.305882353	0.670731707
anapc10	1.305785124	0.921052632
thap1	1.305084746	0.966666667
wu:fj30f06	1.304347826	0.833333333
recql5	1.304347826	1.078431373
si:dkey-3p4.7	1.304347826	2.2
CRISP3	1.303738318	1.568527919
zgc:113348	1.303571429	1.128712871
gatm	1.303316444	1.411181435
si:dkey-245n4.2	1.303030303	0.307692308
zgc:113518	1.303030303	0.658536585
brinp3a.2	1.303030303	0.727272727
si:dkey-117i10.1	1.303030303	1
BX005085.5	1.303030303	1.333333333
si:ch211-222k6.2	1.303030303	1.647058824
R3HDM2	1.302325581	0.676470588
vti1a	1.302197802	1.352657005
zgc:172051	1.302158273	1.853211009
celsr2_1	1.301886792	0.365384615
klhl3	1.301886792	0.844444444
iah1	1.301886792	1.274193548
tle3a	1.301775148	0.614035088
stard14	1.301694915	2.248995984
ankrd39	1.301587302	0.895348837

pcdh19	1.301310044	0.38172043
sfswap	1.301204819	1.048979592
serp2	1.301204819	1.141304348
ankrd9	1.301107754	1.108751062
robo4	1.3	0.35
robo2_1	1.3	0.367816092
gabrd	1.3	0.509433962
CABZ01055534.1	1.3	0.555555556
CABZ01066035.1	1.3	0.684210526
si:dkeyp-117b8.4	1.3	0.712121212
zgc:171673	1.3	0.8
her4.3	1.3	0.846153846
zgc:112320	1.3	0.854545455
slc41a2b	1.3	0.887096774
c1qc	1.3	0.909090909
pyya	1.3	0.973180077
FUK	1.3	1.057142857
gemin7	1.3	1.224852071
cbln4	1.3	1.296296296
sst6	1.3	1.342857143
CR762483.1	1.3	1.416666667
zgc:101100	1.3	1.481481481
cishb_1	1.3	2.4
synrg	1.3	0.779069767
errfi1	1.299492386	0.764444444
scn1lab	1.298701299	0.363636364
abcc5	1.298387097	1.116666667
jun	1.298245614	0.891213389
ATG14	1.298013245	0.987179487
prickle2a	1.29787234	0.268292683
cldnd1b	1.297520661	1.073426573
fam73a	1.297520661	1.138211382
TSC2	1.297297297	0.763157895
cln5	1.297297297	1.146341463
si:ch211-284e13.5	1.296296296	1.034482759
MAD2L2	1.296296296	1.378787879
kiaa1549la	1.294573643	0.292929293
slitrk2	1.294117647	0.306122449
neo1b_1	1.294117647	0.53125
CABZ01052576.1	1.294117647	0.559322034
slc9a7	1.294117647	0.575757576
ireb2	1.294117647	0.731034483
tut1	1.294117647	0.803030303
b3galt4	1.294117647	0.958333333
chrna7	1.294117647	1
si:ch211-26b3.2	1.294117647	1
GNG12 (1 of many)	1.294117647	1
rwdd2b	1.294117647	1.125
pih1d1	1.294117647	1.314285714
dusp19a	1.294117647	1.4
EMB	1.294117647	2.214285714
si:dkey-3k24.5	1.293333333	0.705882353
senp5_2	1.293333333	0.776470588
DUSP27	1.293103448	0.923611111
plppr2a	1.292682927	0.384615385
srpk3	1.292682927	0.608391608
CABZ01086806.1	1.292682927	1.178571429
abi2b	1.291946309	0.793103448
gstcd	1.291666667	0.852941176
fam168b	1.29144385	0.431554524
atrnl1b	1.291139241	0.554054054

dact3a	1.291139241	0.818181818
pgghg	1.290909091	1.612244898
phc1	1.290322581	0.759259259
dus4l	1.290322581	1.174418605
rnf216	1.29	0.87
btg3	1.289719626	1.104347826
LAMP1	1.289473684	1.196078431
LAMP1	1.289473684	1.196078431
sox9a	1.289377289	0.783882784
ercc2	1.289256198	1.296296296
irx2a	1.289156627	0.768292683
CABZ01113371.1	1.288888889	0.357142857
TTC37	1.288888889	0.826086957
dph6	1.288888889	1.5625
mafbb	1.288770053	0.829131653
tiam2a	1.288461538	0.962962963
mapk8ip3	1.288209607	0.327014218
pdk2b	1.288154897	2.473895582
zgc:158343	1.287671233	2.363157895
trim37	1.287356322	0.792207792
sst1.2	1.286885246	0.900943396
atp5s	1.286764706	0.908450704
il10rb	1.286516854	1.077319588
bhlhe40	1.286491387	0.795899348
pbx1b	1.285714286	0.53968254
otol1b	1.285714286	0.619047619
mtmr4	1.285714286	0.642857143
si:dkey-81l17.7	1.285714286	0.6875
CABZ01078944.1	1.285714286	0.76744186
hps3	1.285714286	0.844155844
si:ch211-10d23.5	1.285714286	0.892857143
gdf3	1.285714286	1
zcchc4	1.285714286	1.014492754
acer3	1.285714286	1.052631579
ten1	1.285714286	1.142857143
mblac2	1.285714286	1.142857143
wdr83	1.285714286	1.148148148
slc39a1	1.285714286	1.166666667
si:ch73-314g15.3	1.285714286	1.473684211
zgc:136308	1.285714286	1.523809524
si:zfos-1451h6.1	1.285714286	1.896551724
vrk2	1.285714286	2.5
edn1	1.285714286	2.833333333
auts2b	1.285714286	0.654320988
znf652	1.285714286	0.732673267
wdr73	1.285714286	1.16
si:dkey-97a13.12	1.285714286	1.166666667
ftcd	1.283969466	1.032727273
spra	1.283783784	1.759036145
mettl17	1.28358209	1.086956522
pim1	1.283464567	0.926974665
slc24a5	1.283333333	1.5625
st7	1.28313253	0.716216216
TMEM74	1.283018868	0.466666667
pcsk2	1.282828283	0.373417722
irx5b	1.282608696	0.731707317
dlx2a	1.282442748	0.822033898
usp20	1.282352941	0.793478261
sox4b	1.282287823	0.701940035
atp2b2	1.282178218	0.290322581
ddhd1b	1.282051282	0.84

epdr1	1.281105991	0.761682243
cmip	1.280898876	0.614785992
dnajb12b	1.280898876	0.72972973
tmem192	1.280898876	1.161290323
si:ch211-150g13.3	1.28	0.448275862
tmem179b	1.28	0.754716981
zgc:162962	1.28	1.513513514
glt1d1	1.28	1.615384615
si:dkey-31n13.4	1.28	2.35
fam91a1	1.279411765	0.573643411
ppip5k1a	1.279411765	0.637931034
tgfbrap1	1.279069767	0.69047619
cep41	1.279069767	0.7
rbm33a	1.279069767	1.046153846
rap2ab	1.279069767	1.166666667
gria4a	1.278688525	0.318181818
abhd17aa	1.27826087	1.028571429
si:ch73-206p6.1	1.27826087	1.524271845
igsf9ba	1.277777778	0.434210526
gpr63	1.277777778	0.493975904
si:dkey-91d10.9	1.277777778	0.722222222
plekhh2	1.277777778	0.8
pax3a	1.277777778	1
pecam1	1.277777778	1.046728972
si:dkey-248g21.1	1.277777778	1.144578313
zgc:165515	1.277777778	1.166666667
man1a2	1.277310924	0.728070175
wu:fi18e02	1.277192982	1.107594937
si:ch211-130m23.5	1.276923077	0.506849315
zcchc2	1.276923077	0.625
tmem185	1.276595745	1.102941176
ankrd29	1.276595745	1.040816327
pus10	1.275862069	0.482142857
foxl2	1.275862069	0.533333333
camsap1b	1.275862069	0.596491228
znf395a	1.275862069	0.879895561
si:ch211-161m3.2	1.275862069	0.885714286
rnd1b	1.275362319	0.601503759
ext1b	1.275362319	0.857142857
si:ch73-100l22.2	1.275362319	1.436781609
paqr3a	1.275229358	1.298850575
CR407583.1	1.27510917	0.900497512
JMJD1C (1 of many)	1.275	0.808219178
zbtb49	1.275	0.815789474
LGALS8 (1 of many)	1.275	0.846153846
rpl38	1.274864376	0.887610223
si:dkeyp-118h3.6	1.274725275	1.532467532
rpl35	1.27406847	1.047146834
kcnj1a.5	1.273381295	1.777777778
dcp2	1.273333333	1.212624585
cspg5a	1.273053892	0.238566131
cc2d2a	1.272727273	0.709677419
smad4a	1.272727273	0.8
cntnap5b	1.272727273	0.413793103
lgals3bpa	1.272727273	0.923076923
fbxl15	1.272727273	0.971428571
mad2l1bp	1.272727273	1.074074074
si:dkeyp-91h6.3	1.272727273	1.142857143
tnni3k	1.272727273	1.166666667
wipf2b	1.271929825	0.485981308
sidt2	1.271875	0.858513189

zgc:193538	1.271604938	1.359375
gfm2	1.271428571	1.569230769
fetub	1.271357395	0.827765654
ndufa9b	1.271317829	1.176470588
si:dkeyp-82a1.6	1.271186441	1.189655172
rbm15b	1.271028037	0.539130435
XKR7 (1 of many)	1.27027027	0.461538462
zbtb44	1.27027027	0.933333333
p2rx5	1.27027027	0.969230769
aldob	1.269491231	0.994037366
sp5l	1.269230769	0.655172414
si:dkey-13n15.2_1	1.269230769	0.75
hmces	1.269230769	1.105263158
hoxb13a	1.269230769	1.369230769
si:ch211-59d2.2	1.269230769	2.818181818
sqrdl	1.269035533	1.293103448
clcn5a	1.268421053	0.82122905
si:ch73-191k20.4	1.268292683	1.458064516
lingo1a	1.268041237	0.320987654
kif21a	1.267857143	0.765957447
atf3	1.267857143	1.115044248
dagla	1.267605634	0.548387097
abhd14a	1.267326733	0.885714286
slc3a2b	1.266717909	1.546944858
si:ch211-51e8.2	1.266666667	0.263157895
nphp4	1.266666667	0.6875
mapkap1	1.266666667	0.75
gpr137c	1.266666667	0.950819672
BX324179.2	1.266666667	1.769230769
apoa4b.3	1.266475645	2.761061947
shisa7b	1.266233766	0.404580153
sprn2	1.265625	1.097560976
zgc:66014	1.265060241	0.647058824
si:dkey-100j22.4	1.265060241	1.361979167
polr2k	1.265	1.281553398
cbln20	1.264957265	1.673913043
zgc:153913	1.264864865	0.956607495
lingo2b	1.264705882	0.362318841
armc9	1.264705882	0.529411765
si:dkey-219c3.2	1.264705882	0.583333333
si:ch73-100l22.3	1.264705882	0.768115942
tmem138	1.264705882	0.931034483
ankrd22	1.264705882	3.263157895
cbll1	1.264367816	0.6
zgc:158254	1.264150943	0.62295082
p3h3	1.264150943	1.530612245
pmaip1	1.264150943	2.164835165
ctnna2	1.264044944	0.395480226
uros	1.264	1.228187919
dcaf15	1.263888889	0.805970149
trim46b	1.263157895	0.461538462
si:ch211-185a18.2_1	1.263157895	0.7
dusp19b	1.263157895	0.823529412
malt1	1.263157895	0.875
kdm4aa	1.263157895	0.917808219
nelfb	1.263157895	0.993710692
si:ch211-235i11.5	1.263157895	1.066666667
tmem168a	1.262626263	0.81
csnk1e	1.262376238	0.516129032
bbs4_1	1.262295082	0.980392157
mmp9	1.261682243	1.35443038

hecw1b	1.261538462	0.392156863
pvalb3	1.261390887	1.184852375
arid1b	1.260869565	0.494117647
wnt4b	1.260869565	0.5
CABZ01021220.1	1.260869565	0.64
zgc:153953	1.260869565	0.833333333
MANBAL	1.260869565	1.093862816
zgc:110224	1.260869565	1.928571429
cebpa	1.260869565	1.049342105
cfap36	1.260504202	0.75
tfpt	1.260204082	0.97107438
tmem56a	1.25984252	0.981818182
sez6l2	1.259668508	0.166666667
mgst3b	1.259416446	1.348904506
tmprss13a	1.259259259	1.262626263
CABZ01080379.1	1.259259259	0.43902439
tjp1b	1.259259259	0.480392157
CABZ01089030.1	1.259259259	0.5
daam2	1.259259259	0.735294118
corin	1.259259259	0.857142857
SLC25A47 (1 of many)	1.259259259	0.857142857
adat2	1.259259259	1.260869565
irgq2	1.259259259	1.571428571
ndufb7	1.258877434	1.178147268
vps8	1.258741259	0.991596639
pgp	1.258687259	1.01754386
nfatc3a	1.258064516	0.71875
ppp1r10	1.258064516	1.024922118
tmem141	1.258064516	2.339285714
arl5a	1.257861635	1.138121547
tcf7l2	1.257731959	0.42118863
tfap4	1.257352941	0.707964602
tecrl2b	1.257142857	0.786885246
mbd2	1.257142857	1.102272727
lrp10	1.256830601	0.952879581
znf277	1.256756757	1.983870968
igfbp1a	1.256694367	1.675503712
igsf21a	1.256097561	0.280487805
si:cabz01066312.1	1.256097561	0.666666667
her13	1.255813953	0.704545455
INPP1	1.255813953	0.923076923
si:dkeyp-68b7.5	1.255813953	1
stau1	1.255725191	0.775119617
proser1	1.255474453	0.508064516
si:cabz01071911.3	1.254901961	1.117647059
stx11b.1	1.254807692	2.855263158
ankrd55	1.254545455	0.740740741
lgi1a	1.254464286	0.540540541
si:ch73-335m24.5	1.254237288	0.829268293
zdhhc1	1.253968254	0.827586207
atp2b3a	1.253869969	0.290441176
rheb	1.253623188	0.745519713
smarcad1b	1.253521127	0.619047619
si:zfos-128g4.2	1.253521127	1.042857143
arhgap35	1.253333333	0.631578947
zeb1a	1.253333333	0.836363636
si:ch211-117c9.5	1.252873563	0.831168831
tob1b	1.252723312	1.540214477
trpm7	1.252427184	0.952941176
mmp24	1.25210084	0.375
ppfia3	1.251336898	0.302325581

ppp1r13bb	1.251162791	0.692307692
slc9a6b	1.25	0.549450549
notchl	1.25	0.583333333
pigm	1.25	0.608695652
zranb1a	1.25	0.643636364
si:ch211-67e16.2_2	1.25	0.666666667
si:ch73-215d9.1	1.25	0.666666667
uhrf1bp1	1.25	0.714285714
SLC5A10	1.25	0.736842105
pax7b	1.25	0.740740741
smarcc1b	1.25	0.7875
AC024175.3	1.25	0.79787234
zgc:172122	1.25	0.80952381
gsx2	1.25	0.882352941
zgc:162872	1.25	0.891304348
sema4ga	1.25	0.9375
gtpbp2	1.25	0.938271605
klhl2	1.25	0.947368421
hmha1b	1.25	0.958333333
nog2	1.25	0.961904762
dnajc16l	1.25	0.981132075
krt96	1.25	1
si:ch211-201b11.3	1.25	1.047619048
SNORA47	1.25	1.109090909
rcbtb1	1.25	1.146341463
mks1	1.25	1.2
MFAP4 (1 of many)_2	1.25	1.333333333
atxn1l	1.25	1.333333333
zgc:113278	1.25	1.35483871
si:ch73-195i19.3	1.25	1.47826087
RDM1	1.25	1.571428571
ccnh	1.25	1.633802817
si:cabz01083442.1	1.25	1.642857143
GRXCR1	1.25	1.7
si:ch211-223o1.4p	1.25	1.733333333
si:ch73-166c6.2	1.25	3.090909091
rnaseh1	1.24852071	1.42
bcl11ba	1.248407643	0.442622951
zic2a	1.248366013	0.598130841
si:ch211-216l23.2	1.247863248	0.741007194
si:ch211-209j10.5	1.247058824	1.181818182
dcp1a	1.246575342	0.744186047
cpsf4	1.246511628	1.236363636
gb:bc139872	1.246511628	1.30472103
tbc1d25	1.246376812	0.882352941
klhdc3	1.246376812	0.941580756
sgk2b	1.246031746	1.6125
OSBP2 (1 of many)	1.245901639	0.75
plxna4	1.245614035	0.353982301
tnni2a.2	1.245535714	1.12565445
si:ch211-85n16.3	1.245508982	1.205128205
tango6	1.245283019	1.084745763
slc12a9	1.245283019	1.088888889
lyrm2_1	1.244897959	0.902912621
mdm2	1.244680851	1.48
cdc26	1.244604317	0.941333333
gcdhb	1.244604317	1.277777778
xkr8.3	1.244444444	1.245283019
tmem55bb	1.244444444	1.393939394
si:dkey-238c7.12	1.244103547	0.692060469
ephx4	1.243902439	0.644444444

pars2	1.243902439	1.086956522
cdc37l1	1.243902439	2.115384615
pdf	1.243589744	1.632352941
cyp2aa9	1.243478261	0.7
snap29	1.243478261	1.202020202
slc36a1	1.243243243	0.9375
si:ch211-120g10.1	1.242990654	0.659090909
TMEM100	1.242718447	0.923076923
pdpk1a	1.242424242	0.896551724
AL954741.1	1.242424242	1.25
si:dkey-82o10.4	1.242424242	1.264705882
fkbpl	1.242424242	1.684210526
psmd1	1.242339833	0.721399731
peli3	1.242307692	0.831831832
natd1	1.242105263	1.039106145
zdhhc17	1.242038217	0.842975207
egfl6_2	1.241545894	0.808300395
robo2_2	1.241525424	0.316939891
snx19a	1.24137931	0.7
znf1009_1	1.24137931	1
LHX3	1.24137931	1.142857143
si:dkey-211f22.5	1.241116751	1.063157895
prmt8b	1.240740741	0.666666667
fbxl2	1.240506329	0.807228916
dbpb	1.240506329	1.004975124
si:dkey-222l20.1	1.24	0.789473684
hs2st1b	1.24	0.815789474
akt3a	1.24	0.833333333
map3k5	1.24	1.061538462
si:ch211-161c3.6	1.24	1.222222222
wu:fc17b08	1.24	1.477272727
si:ch211-261p9.4	1.24	1.765625
kif3cb	1.23943662	0.729323308
ndufa7	1.239244491	1.108763693
trim46a	1.239130435	0.470588235
dalrd3	1.239130435	0.652777778
SYNPR (1 of many)	1.239130435	0.71641791
mecr	1.239130435	1.441558442
aspscr1	1.239130435	0.670391061
pou4f2	1.23902439	0.365
prkacab	1.23828125	0.510548523
atrn	1.238095238	0.681415929
gdap1	1.238095238	1.129411765
si:dkey-121j17.6	1.238095238	1.146341463
APBB3	1.237288136	0.27027027
mtf2	1.237288136	0.727272727
dph1	1.237288136	0.735294118
si:dkey-210j14.3	1.237288136	1.127272727
scxa	1.237288136	1.42
zgc:172053	1.237288136	2.047244094
adi1	1.237226277	1.191056911
mrpl42	1.23655914	1.032989691
spata20	1.236363636	0.529411765
atp1a1a.2	1.23630137	1.285240464
dmbx1a	1.236111111	0.834782609
ppp1r12a	1.235849057	0.637254902
ppdpfb	1.23571147	0.829990317
cstf3	1.235449735	0.964497041
ca9	1.235294118	0.55
jag1a	1.235294118	0.588235294
zgc:163061_1	1.235294118	0.769230769

si:dkey-52d15.1p	1.235294118	1.125523013
nab2	1.235294118	1.384615385
zgc:91909_2	1.234972678	1.184210526
prkcbb	1.234693878	0.5125
si:dkey-100j22.7	1.234693878	2.14
fzd5	1.234375	0.519230769
ash1l	1.234375	0.682692308
ppp2r2ba	1.234234234	0.510204082
tor3a	1.234234234	1.139784946
trib2	1.234042553	0.622807018
med13a	1.233576642	0.625
tceb3	1.233415233	0.783980583
ccs	1.233333333	0.651162791
wfs1b	1.233333333	1.296296296
gamt	1.233034212	0.949419002
slc17a5	1.233031674	0.842034806
si:ch211-14c7.2	1.233009709	0.345238095
tmx4	1.232876712	0.936507937
ppp2r3c	1.232876712	1.29375
ubap1	1.232758621	0.838095238
ralgps2	1.23255814	0.897435897
cst14a.2	1.232334047	1.254545455
wbscr17	1.232142857	0.676470588
zgc:77486	1.231884058	0.79389313
tbcd	1.231884058	0.898305085
LAMTOR4	1.231843575	1.097323601
chsy1	1.231707317	0.865671642
tmem60	1.231578947	1.608695652
arrdc3a	1.231511254	1.830232558
pou3f2b	1.230769231	0.381147541
spsb4a	1.230769231	0.568807339
si:dkey-256h2.1	1.230769231	0.846153846
eva1a_2	1.230769231	0.875
fgfr1b	1.230769231	0.944444444
fan1	1.230769231	0.971428571
tnfrsf11b	1.230769231	0.972222222
CABZ01072853.1	1.230769231	1.072727273
pigb	1.230769231	1.104651163
zgc:55943	1.230769231	1.125506073
si:ch211-266g18.6	1.230769231	1.18
tmem8a	1.230769231	1.418604651
si:ch73-299h12.1	1.230769231	1.709677419
isca1	1.230686695	1.342034203
cbr1l	1.230612245	1.618473896
taf7	1.230538922	1.327272727
zgc:55781	1.23	0.771428571
ttc14	1.229885057	1.092307692
clcn2c	1.22972973	1.187878788
ggps1	1.229357798	1.157894737
mri1	1.229299363	0.539823009
si:dkey-194g4.1	1.229166667	1.1875
aarsd1	1.229007634	1.216666667
mtg2	1.228915663	0.989247312
med28	1.22875817	0.761658031
INSM2	1.228571429	0.428571429
bbs9	1.228571429	0.681818182
si:ch1073-210e22.1	1.228571429	1.733333333
r3hdm1	1.228365385	0.356962025
si:dkey-105e17.1	1.22826087	0.134831461
ALKBH3 (1 of many)	1.22826087	1.2375
NACC1 (1 of many)	1.228070175	0.553571429

wdr24	1.228070175	0.846153846
ube2j1	1.227941176	0.891666667
pink1	1.227941176	1.509615385
ppm1la	1.227564103	0.793650794
fcho1	1.227272727	0.481481481
si:ch211-274k16.6	1.227272727	0.92
dbx1b	1.227272727	1.209302326
armc7	1.227272727	1.225806452
b3gntl1	1.227272727	1.363636364
tmem144a	1.227272727	1.523809524
BX572103.7	1.227272727	2.125
si:cabz01032474.1	1.227272727	2.133333333
tgif1	1.226804124	0.565853659
zranb2	1.226470588	1.699300699
pdrg1	1.226415094	1.489795918
mych	1.226107226	1.12962963
zdhhc4	1.226086957	1.223140496
atat1	1.225806452	0.626168224
si:dkey-4c15.13	1.225806452	0.657894737
GTSF1 (1 of many)_2	1.225806452	0.680851064
si:ch211-107n13.1	1.225806452	1.068965517
irf2bp2a	1.22537112	0.879503106
mier2	1.225352113	1.423076923
josd1	1.225274725	1.315508021
cpsf6	1.225247525	0.636363636
si:ch1073-339l18.2	1.225	1.182795699
psph	1.224806202	0.769230769
tgfbr1b	1.224806202	1.114583333
cacng3b	1.224719101	0.35443038
dctn5	1.224683544	1.530120482
ppp4r1l	1.224489796	0.798319328
epc1a	1.224489796	0.758241758
CLMP	1.224489796	0.765957447
znf609a	1.224299065	0.652631579
pigp	1.224242424	0.797413793
lyrm4	1.224242424	1.6796875
myo16	1.223880597	0.326530612
lmo3	1.223188406	0.599378882
ap1s3b	1.223140496	1.109540636
npl	1.223140496	1.633333333
zgc:110063	1.222748815	1.115384615
samm50l	1.222627737	0.8
stub1	1.222627737	0.944636678
dnajc8	1.22244898	0.758691207
bsnb	1.222222222	0.25
insm1b	1.222222222	0.375796178
coro7	1.222222222	0.727272727
ypel2a	1.222222222	0.80952381
CABZ01069287.1	1.222222222	0.863636364
snx6	1.222222222	0.886363636
si:ch73-40a17.7	1.222222222	1
wu:fe17e08	1.222222222	1.181818182
si:zfos-223e1.2	1.222222222	1.181818182
dtwd1	1.222222222	1.215384615
bik	1.222222222	1.315789474
si:ch211-253p18.2	1.222222222	1.3625
si:ch211-110e21.4_1	1.222222222	1.368421053
si:dkey-159n16.2	1.222222222	1.384615385
pik3r3a	1.222222222	1.4453125
pcdh2ab9	1.222222222	1.533333333
si:cabz01063885	1.222222222	1.586206897

urp2	1.222222222	1.638888889
polg2	1.222222222	1.958333333
ankdd1b	1.222222222	2.052631579
TMEM216	1.222222222	3.578947368
BEGAIN	1.222222222	0.372093023
si:ch211-194b1.1	1.222222222	0.631578947
zgc:162928	1.222222222	0.703703704
si:dkey-92i15.4	1.222222222	1.947368421
znf839	1.221428571	0.6
cdk12	1.221374046	0.64028777
si:ch211-244c8.4	1.221311475	0.247311828
CABZ01076616.1	1.221266134	0.940280066
ctnnd2b	1.221238938	0.404494382
si:dkey-33m11.8	1.221153846	1.23015873
sp2	1.221052632	0.666666667
depdc5	1.221052632	0.947368421
fam222a	1.220588235	0.615384615
LMF1	1.220588235	1.298245614
mrpl10	1.220472441	1.207792208
kdm6ba	1.220408163	0.847619048
pou4f3	1.220338983	0.838709677
CABZ01092282.1	1.22	0.186528497
reps1	1.21978022	0.857142857
zfand1	1.21978022	1.932432432
grk6	1.219512195	0.62962963
FNIP2	1.219512195	0.65625
aig1	1.219512195	0.827956989
rnf19b	1.21875	0.612903226
ppm1lb	1.21875	0.714285714
si:dkey-9c18.3	1.21875	0.891304348
xpa	1.21875	1.214285714
eif3eb	1.218487395	1.247311828
rpl39	1.218194025	0.863281668
elac1	1.218181818	0.962962963
slc8b1	1.218181818	1.043478261
grm1a	1.217948718	0.402985075
KLHL29 (1 of many)_2	1.217948718	0.697674419
ubxn4	1.217821782	0.753731343
stm	1.217619987	2.044
lonrf1l	1.21761658	1.183976261
pomca	1.217391304	0.625
osbpl10	1.217391304	0.84375
znf1144_2	1.217391304	0.9
jam2a	1.217391304	1.044247788
sh3glb2a	1.217391304	1.214285714
cxcr4b	1.217391304	1.464788732
xpnpep3	1.217391304	1.483606557
tmem170a	1.217391304	1.492537313
eif4ba	1.216981132	0.785714286
fbxo41	1.216666667	0.4375
hoxc13a	1.216666667	1.412698413
ctso	1.216666667	1.714285714
slc29a4	1.216494845	0.682926829
ptprsa	1.21641791	0.64957265
rbp2a	1.216043141	1.295418221
mntb	1.215909091	0.731707317
hdac5	1.215909091	0.960526316
scml4	1.215686275	0.764705882
fam69b_1	1.215686275	0.789473684
ubox5	1.215686275	1.183673469
med11	1.21559633	1.603015075

rsrc1	1.215517241	0.940740741
mgst2	1.215517241	0.981132075
kif3b	1.215384615	0.888888889
mybphb	1.215197168	0.881933644
nup160	1.215	0.756345178
si:rp71-1h20.9	1.214285714	0.944444444
gpx1b	1.214285714	0.983333333
pcdh1g26	1.214285714	1
ftr51	1.214285714	1
CABZ01080371.1	1.214285714	1.538461538
rtn4rl2b	1.214285714	1.545454545
apela	1.214285714	1.8
furinb	1.214285714	0.725806452
ulk3	1.214285714	0.75
taf4a	1.214285714	0.847826087
ndufaf7	1.214285714	1
mylipa	1.214285714	1.242236025
pak2b	1.214123007	0.73923445
ccng2	1.214057508	1.801980198
rpl32	1.214057193	0.947064691
AC024175.4	1.213909282	0.725573831
phtf2	1.213793103	0.708074534
dmxl2	1.213675214	0.436781609
chd3	1.213675214	0.536842105
clec14a	1.213675214	1.707964602
dusp7	1.213483146	0.855421687
fmnl2b	1.213333333	0.5
vipas39	1.213333333	1.169014085
NOCT (1 of many)	1.213333333	1.426470588
mff	1.213178295	1.085106383
l3mbtl1a	1.213114754	0.368421053
UMAD1	1.213114754	1.386363636
stxbp1a	1.213053263	0.252267106
ogfr	1.212903226	0.705479452
gcm2	1.212765957	1.402298851
foxi3b	1.212643678	1.245989305
brdt	1.212598425	0.948979592
si:dkey-35h6.1	1.212328767	2.734265734
ttbk2b	1.212121212	0.52173913
si:dkey-200l5.4	1.212121212	0.772727273
senp7b	1.212121212	1.216216216
rhbdd1	1.212121212	1.379310345
znf703	1.212025316	0.759197324
gstp2	1.211886305	2.145708583
ankrd13d	1.211864407	0.666666667
med18	1.211764706	1.05
phox2bb	1.211678832	0.558823529
grm8a	1.211538462	0.268292683
irf2bpl	1.211169284	0.503311258
stam	1.211155378	0.753303965
rps29	1.211133592	0.940761583
si:ch211-126j24.1	1.211111111	0.554054054
dia1b	1.211111111	0.786516854
PYURF	1.211111111	0.980327869
PLPP2 (1 of many)	1.211009174	1.294736842
stk16	1.2109375	1.100917431
babam1	1.210884354	1.051851852
grifin	1.210691824	2.116883117
si:dkey-221l4.11	1.210526316	0.423076923
rbm14b	1.210526316	0.561151079
arhgap33	1.210526316	0.5625

dock7	1.210526316	0.65248227
si:dkey-245f22.5	1.210526316	0.756756757
dbx1a	1.210526316	0.907216495
acy3.1	1.210526316	1.047619048
dtl	1.210526316	1.08
si:ch73-142c19.1	1.210526316	1.2
si:ch73-280o22.2	1.210526316	1.3125
slc26a11	1.210526316	1.368421053
si:ch211-262i1.5	1.210526316	1.510638298
sesn3	1.210227273	0.779342723
cnot4a	1.210084034	0.723214286
tnika	1.21	0.38372093
prdx1	1.20995671	1.70609319
SHC3	1.209677419	0.454545455
mtbl	1.209677419	0.873261206
zgc:92907	1.209302326	1.088235294
MAPK8IP1	1.208955224	0.290909091
ttyh3b	1.208502024	0.409090909
scube2	1.208333333	0.516129032
nkx3.2	1.208333333	0.647619048
CABZ01053976.1	1.208333333	0.792207792
hps4	1.208333333	0.885714286
rnf34b	1.208333333	1
CLCC1	1.208333333	1.274509804
cybrd1	1.208333333	1.396226415
si:dkey-4c15.16	1.208333333	1.470588235
esrrga	1.208121827	0.409937888
elmo2	1.208092486	0.530201342
rngtt	1.208	0.706766917
akap10	1.208	0.854368932
irx1b	1.207792208	0.398148148
synj1	1.207792208	0.481818182
atp6ap1a	1.207650273	0.376623377
FAM189B	1.20754717	0.371428571
sema6bb	1.20754717	0.70212766
acot9.1	1.20754717	1.87804878
ccdc106a	1.20746888	1.307692308
mark2a	1.207446809	0.812154696
srebf1	1.207207207	0.842105263
pwwp2b	1.207142857	0.687898089
zgc:101716	1.206896552	0.974358974
si:dkeyp-2e4.3_1	1.206896552	1.090909091
vipr2	1.206896552	1.095238095
spi1a	1.206896552	1.214285714
gnao1a	1.206673843	0.333806818
tpra1	1.206666667	1.082278481
strada	1.206060606	1.067567568
otpb	1.206008584	0.578680203
trib1	1.205882353	1.876623377
cntn5	1.205882353	0.345454545
prkag3a	1.205882353	1.576923077
pcgf6	1.205882353	1.6
si:dkey-33i11.4	1.205438066	1.456888889
foxo3a	1.205323194	0.522222222
pbxip1a	1.205298013	1.097902098
SZT2	1.205128205	0.693877551
pdp2	1.205128205	0.75
mkxa	1.205128205	0.810810811
cyr61l2	1.205128205	0.948717949
nudt3b	1.205128205	1.04040404
RAB21	1.204878049	0.805739514

AC024175.15	1.204865557	0.883454735
chmp4ba_1	1.204771372	1.150326797
ralgapb	1.204724409	0.75
tprg1l	1.204724409	1.20212766
efnb3b	1.204678363	0.702702703
kdm4ab	1.204545455	0.972727273
hcn2b	1.204545455	0.347826087
exd3	1.204545455	0.644067797
rbm25a	1.204545455	0.710526316
cfap97	1.204545455	0.976744186
tab1	1.20441989	0.783783784
si:dkeyp-110a12.4	1.203703704	1.051851852
mknk1	1.203703704	1.520547945
vps11	1.203539823	0.754901961
rab11bb	1.203389831	0.607361963
epha7	1.203389831	0.653846154
nxph1	1.203333333	0.616
poglut1	1.203125	1.471698113
CABZ01045062.1	1.202898551	0.530973451
si:dkeyp-69b9.3	1.202898551	0.793103448
rnd2	1.202898551	1
si:dkey-286j15.3	1.202898551	1.339622642
CYP27A1 (1 of many)	1.202898551	1.412698413
nrxn3a	1.202702703	0.346153846
tmed1a	1.202702703	0.598360656
bcas3	1.202702703	0.865671642
gpr108	1.202702703	1.224669604
ppfia1	1.202614379	0.939597315
bmp7b	1.202380952	1.153846154
etv5a	1.202247191	0.822134387
pan2	1.202247191	0.746835443
rap2c	1.202185792	0.788834951
pcdh10b	1.202072539	0.443181818
si:ch211-39k3.2	1.201923077	1.292682927
cttnbp2	1.201219512	0.64375
fryb	1.201183432	0.651515152
rpl29	1.200093067	1.053584102
si:ch73-362m14.4	1.2	0.227272727
taf3	1.2	0.588477366
dgcr2	1.2	0.652173913
tfb1m	1.2	0.661971831
mbd5	1.2	0.676056338
ptger1b	1.2	0.6875
phlpp1	1.2	0.75
si:ch211-210b2.1	1.2	0.80952381
sox8b	1.2	0.833333333
map4k3b	1.2	0.842105263
slc29a3	1.2	0.87012987
lztr1	1.2	0.905405405
kdm8	1.2	0.945454545
pofut1	1.2	0.991735537
CABZ01072827.1	1.2	1
si:ch211-214j8.12	1.2	1.05
c18h3orf33	1.2	1.066666667
timm9	1.2	1.090909091
ago2	1.2	1.1
wwox	1.2	1.136363636
nudt19	1.2	1.144578313
si:dkeyp-117b11.2	1.2	1.176470588
ptger4b	1.2	1.227272727
dph3	1.2	1.24137931

dctd	1.2	1.245614035
sertad2a	1.2	1.253731343
ttc22	1.2	1.275
AL954146.2	1.2	1.290322581
FGD4 (1 of many)	1.2	1.3
selt1b	1.2	1.302052786
rell2	1.2	1.333333333
wdr35	1.2	1.333333333
im:7145024	1.2	1.384615385
btr25	1.2	1.409090909
si:ch211-147m6.1	1.2	1.471698113
tbxa2r	1.2	1.583333333
si:dkey-1f1.3_3	1.2	1.583333333
MCUR1	1.2	1.629213483
znf1115_2	1.2	2.181818182
si:dkeyp-92c9.4	1.2	2.285714286
hmgb1a	1.199280728	1.160188722
si:ch73-209e20.6	1.198979592	1.035087719
tmeff2a	1.198529412	0.503267974
zc3h4	1.198529412	0.64084507
clgn	1.198369565	0.718475073
cul1a	1.198347107	0.8
tob1a	1.198347107	1.098688751
fzr1b	1.198198198	0.810483871
fbxl5	1.198019802	0.983695652
mrpl13	1.197959184	1.019417476
nkiras2	1.197860963	1.068783069
cnot11	1.19760479	0.623036649
dscamb	1.197530864	0.254237288
sbf2	1.197530864	0.813333333
stard4	1.197368421	0.662790698
heca	1.197368421	0.842105263
akap8l	1.197324415	0.788091068
meis2a	1.197201018	0.448529412
rsf1a	1.197183099	1.090909091
mast1b	1.196969697	0.347826087
si:dkey-161j23.5	1.196969697	0.764705882
ccdc126	1.196969697	0.987804878
HSCB	1.196969697	2.017241379
si:ch211-266o15.1	1.196581197	0.705357143
PDZD4 (1 of many)_2	1.196428571	0.428571429
CABZ01067060.1	1.196428571	0.692307692
kif13a	1.196428571	0.881355932
CABZ01069339.1	1.196261682	0.87
pim3	1.196261682	0.871134021
shox2	1.196078431	0.912280702
slc2a12	1.196078431	1.306122449
gle1	1.195876289	0.946236559
TBC1D9B	1.195652174	0.829787234
si:cabz01015814.1	1.195652174	1.035714286
pdhx	1.195599022	0.834763948
ttc33	1.195488722	1.18852459
gfra1b	1.195121951	0.829268293
gdap1l1	1.195121951	0.838709677
igf1rb	1.195121951	0.853658537
zgc:56409	1.195121951	0.881355932
naf1	1.195121951	0.902173913
si:ch211-67n3.9	1.195121951	0.933333333
scrn2	1.195121951	0.987804878
epc1b	1.195	0.67816092
mief2	1.194915254	0.936507937

cops3	1.194835681	1.131111111
dnm1b	1.194756554	0.402061856
klhl20	1.194690265	0.74796748
ppfia4	1.194444444	0.416666667
dock10	1.194444444	0.666666667
cxxc5b	1.194444444	0.673684211
klhl32	1.194444444	0.902439024
CABZ01090749.1	1.194444444	1.15625
nkd2a	1.194444444	1.25
gfi1ab	1.194444444	1.612903226
ncam1a	1.19434629	0.458715596
si:dkey-28b4.8	1.194331984	0.442708333
fbrsl1	1.19375	0.589041096
fam208ab	1.19375	0.623188406
eif4a2	1.193684211	1.026923077
grid1a	1.193548387	0.483870968
si:dkeyp-68b7.10	1.193548387	1.157894737
qkib	1.193181818	0.628571429
meis2b	1.193133047	0.498721228
ier5	1.193087008	1.219714964
nkx2.2a	1.192771084	1.022727273
NABP2	1.192771084	1.508474576
si:dkey-189h5.6	1.19266055	2.037735849
cactin	1.192307692	0.738853503
caskb	1.192307692	0.487804878
mettl15	1.192307692	1.095238095
znf1015_2	1.192307692	1.166666667
c1galt1a	1.192307692	1.304347826
TATDN3	1.192307692	1.367816092
TATDN3	1.192307692	1.367816092
lmln	1.192307692	1.5625
cep44	1.192307692	1.6
cdk5rap1	1.192307692	2.416666667
BX255883.1	1.192307692	3.142857143
fbxw11b	1.192192192	0.683246073
nsa2	1.192162323	1.02663274
prkcea	1.192090395	0.72027972
ciao1	1.192	1
CABZ01087568.1	1.191950464	0.695652174
rprd2b	1.191780822	0.529411765
si:dkey-96g2.1	1.191780822	1.597826087
rnd1a	1.191709845	0.698324022
BRSK1 (1 of many)_1	1.191666667	0.352941176
AC024175.17	1.191509889	1.060769231
mkxb	1.191489362	1.1875
btbd9	1.191304348	0.952380952
wdr48b	1.191304348	1.329787234
NUPR2	1.190789474	0.985981308
astn1	1.190751445	0.28030303
smarce1	1.190737834	0.613018598
srpk2	1.19047619	0.452991453
si:ch73-382f3.1	1.19047619	0.52
dgkg	1.19047619	0.612903226
xpo6	1.19047619	0.614379085
tcf19l	1.19047619	0.636363636
si:ch211-193l2.6	1.19047619	0.68
tiparp	1.19047619	0.831578947
frmd6	1.19047619	0.927536232
CABZ01059408.1	1.19047619	1.090909091
rab36	1.19047619	1.307692308
ephx5	1.19047619	1.32967033

mbtps2	1.19047619	1.556962025
si:ch211-110p13.9	1.19047619	1.764705882
znf1041	1.19047619	2
exoc5	1.190184049	1.349315068
faim2a	1.190184049	1.440366972
nat9	1.190045249	1.640625
rltpr	1.189873418	0.208333333
si:ch211-11n16.2	1.189873418	1.322580645
crbn	1.189873418	1.364864865
gng13a	1.189781022	1.077348066
si:dkey-221l21.2	1.189655172	0.522058824
si:ch73-233f7.7	1.189655172	0.970588235
rbfox1	1.189616253	0.509043928
exoc3l2a	1.189473684	1.385416667
def8	1.189427313	1.288288288
coq7	1.189393939	1.282208589
SMARCA4 (1 of many)	1.189189189	0.392857143
cax1	1.189189189	1.125
ikzf5	1.188888889	0.547368421
ythdf3	1.188679245	0.794117647
inpp5e	1.188405797	0.68115942
als2b	1.188235294	0.662921348
rel	1.188235294	1.404040404
arhgef9a	1.188118812	0.577464789
iscub	1.188118812	1.027522936
exosc5	1.187969925	1.275280899
mrpl28	1.187951807	0.950471698
her4.2_2	1.187919463	1.838383838
rprd1b	1.187793427	1.022813688
emsy	1.1875	0.572254335
foxn4	1.1875	0.581818182
srrm4	1.1875	0.451612903
RECQL4	1.1875	0.578947368
shc2	1.1875	0.846153846
si:dkey-97a13.5	1.1875	1
unc119.2	1.1875	1
wdfy1	1.1875	1.026143791
cmklr1	1.1875	1.103448276
CABZ01072765.1	1.1875	1.181818182
znf692	1.1875	1.4
rpl36	1.18737142	0.820887889
mpped2	1.186813187	0.577319588
xylt1	1.186440678	0.6
ubn2a	1.186440678	0.630434783
nek1	1.186440678	0.92
ubr2	1.186335404	0.93006993
dlgap3	1.186046512	0.444444444
heatr6	1.186046512	0.673913043
KIAA0895L	1.186046512	0.782608696
glcci1	1.186046512	0.825
ccdc77	1.186046512	1.027777778
alms1	1.186046512	1.064516129
rab34b	1.186046512	1.245283019
nlgn2a	1.185714286	0.363636364
iba57	1.185714286	1.14
dis3l	1.185714286	1.215686275
ldlrad3	1.185430464	0.951388889
gsta.2	1.185410334	1.562913907
fstl4	1.185185185	0.545454545
zbtb11	1.185185185	0.805309735
focad	1.185185185	0.826086957

atm	1.185185185	1.238095238
acp2	1.185185185	1.249042146
fkbp5	1.185125303	1.074660633
ube2c_2	1.184971098	1.135483871
ctsh	1.18487395	1.90990991
CACFD1	1.184782609	1.393442623
rpl31	1.184776059	0.86596673
ppfia2	1.184615385	0.5
fbxw5	1.184357542	0.696629213
DHX29	1.184210526	0.892307692
fopnl	1.184210526	1.042553191
nrbf2b	1.184210526	1.096491228
pdgfba	1.184210526	1.233333333
sh2b1	1.184210526	0.801242236
dpagt1	1.18404908	1.329032258
pax2a	1.183908046	0.313432836
ntn2	1.183673469	0.72972973
tor2a	1.183673469	1.553846154
lysmd3	1.183544304	1.16
slc30a4	1.183431953	1.111111111
saraf	1.183431953	1.15625
PROC (1 of many)	1.183333333	0.72815534
thap4	1.183333333	1.190082645
gde1	1.183333333	1.257142857
hint2	1.183229814	1.132275132
fam219ab	1.183035714	0.493243243
dnajc19	1.183035714	1.06122449
st3gal8	1.182608696	1.473214286
ccdc6a	1.182481752	0.700680272
ptprna	1.182222222	0.328042328
sv2a	1.182186235	0.261006289
si:dkey-183c6.8	1.181818182	0.487179487
exosc3	1.181818182	0.890052356
nemp2	1.181818182	1.065217391
BX640406.1	1.181818182	1.206896552
ms4a17a.10_2	1.181818182	1.321428571
klhl13_1	1.181818182	0.379310345
kifap3a	1.181818182	0.680555556
atr	1.181818182	0.761904762
cab39_1	1.181818182	1.024691358
arhgef3	1.181818182	1.1
cep78	1.181818182	1.117647059
cd164l2	1.181818182	1.163043478
tspan33b	1.181818182	1.222222222
FO704635.1	1.181818182	1.235294118
itgb1bp1	1.18134715	1.28342246
ing3	1.181333333	1.251572327
thumpd1	1.181208054	0.60989011
plaa	1.181208054	0.651315789
tpmt.1	1.180995475	1.530612245
abtb1	1.180851064	1.342465753
rnf167	1.180851064	1.351851852
trrap	1.180722892	0.703125
iars2	1.180722892	0.9125
si:dkey-12e7.1	1.180722892	1.625
cacng8a	1.180555556	0.350877193
brpf3b	1.180555556	0.757142857
rpl27	1.180343511	0.919593008
znf451	1.18018018	0.870689655
b4galnt1b	1.18	0.513513514
pofut2	1.18	1.126315789

bloc1s1	1.18	0.989473684
nat14	1.179775281	1.3
myeov2	1.179755672	1.121985816
mrps17	1.1796875	1.15
nova2	1.179577465	0.353080569
atp5ia	1.179523142	0.995520717
ints1	1.179487179	0.651785714
ATG2A	1.179487179	0.73015873
vps53	1.179487179	1.024390244
irs1	1.179487179	1.027777778
paox1	1.179487179	1.6
znf1001_1	1.179487179	2.034482759
CABZ01066813.1	1.179310345	0.819548872
gemin2	1.179104478	1.188841202
rpl22	1.179029642	0.88250974
si:dkeyp-115e12.7	1.178861789	0.683823529
BUD13	1.178861789	1.168316832
pcsk1	1.178807947	0.52
magi2a	1.178571429	0.636363636
saal1	1.178571429	0.85915493
grem1a_2	1.178571429	1.041666667
dexi	1.178571429	1.25
AC024175.5	1.17826751	0.94675239
tbc1d30	1.178217822	0.626506024
grip1	1.178082192	0.577235772
dpysl5b	1.17804878	0.394936709
kansl1b	1.177966102	0.752475248
mrpl48	1.177966102	1.439814815
usp19	1.17791411	0.888059701
arpc5la	1.177777778	0.709375
zgc:174224_2	1.177777778	1.133333333
twf2b	1.177777778	1.722222222
atp6ap1b	1.177710843	1.439102564
per1b	1.17768595	0.668789809
rangap1b	1.177570093	1.212121212
cdip1	1.177514793	0.857142857
efna3b	1.177419355	0.418181818
ccdc85al	1.177419355	0.660714286
chsy3	1.177419355	1.301886792
tuba1b	1.177364865	0.700315457
si:ch1073-390k14.1	1.17721519	1.038167939
cadpsb	1.176923077	0.338797814
si:ch73-127m5.2	1.176923077	1.411764706
psmf1	1.176870748	0.792207792
pcbp2	1.176817289	0.757352941
myl10	1.176808654	1.014589941
fam122b	1.17679558	1.305970149
gnsa	1.176724138	0.833333333
NUDT16	1.176470588	0.5
gsg1l2b	1.176470588	0.538461538
yeats2_2	1.176470588	0.545454545
NHLRC3	1.176470588	0.648148148
hdlbpb	1.176470588	0.754098361
ptpn23a	1.176470588	0.768115942
armc5	1.176470588	0.774193548
jade2	1.176470588	0.8125
dusp22b	1.176470588	0.826086957
DRAXIN (1 of many)	1.176470588	0.892857143
pamr1	1.176470588	0.894736842
pamr1	1.176470588	0.894736842
adrb2b	1.176470588	1.117647059

si:dkey-77p23.6	1.176470588	1.142857143
znf1167	1.176470588	1.76
TCERG1 (1 of many)	1.176056338	0.826771654
ndufc2	1.175989086	1.100929054
gtf2h4	1.175925926	0.719178082
tmem9	1.175925926	1.568627451
ftr83	1.175879397	1.069651741
zgc:77058	1.175572519	0.274725275
epha6	1.175438596	0.461538462
adprh	1.175438596	1.090909091
wdr41	1.175438596	1.012345679
fbxo21	1.175257732	1.195121951
lrrc24	1.175	0.625
gpr155b	1.175	0.893617021
cdkl5	1.174603175	0.222222222
myt1b	1.174603175	0.28
scube3	1.174603175	0.880952381
FQ323163.1	1.174603175	1.355932203
ambra1b	1.174418605	0.701492537
trappc2	1.174358974	1.008733624
znhit1	1.174311927	1.486238532
fam110b	1.174242424	0.519083969
inip	1.174193548	1.175324675
ugt1b7	1.173913043	0.529411765
si:ch73-305o9.3	1.173913043	0.647058824
syt14b	1.173913043	0.9375
flt1	1.173913043	1.210526316
si:ch73-266f23.1_3	1.173913043	1.391304348
fbxo32	1.173913043	2.305555556
chmp1a	1.173913043	0.993589744
CABZ01029366.1	1.173913043	1.2
pck1	1.173897952	1.441625829
CABZ01072538.1	1.173469388	0.432835821
itpa	1.173267327	1.638888889
L3MBTL4	1.173076923	0.88
si:ch73-44m9.3_2	1.173076923	2.084745763
slc20a1a	1.172995781	0.959839357
TESK1 (1 of many)_1	1.172932331	0.634408602
tmeff1b	1.172903226	0.638655462
scyl1	1.172839506	0.732919255
fam149b1	1.172839506	0.738461538
uxt	1.172839506	1.609756098
kif5aa	1.172804533	0.332242226
rimkla	1.1728	0.45819398
mtmr6	1.172661871	1.024793388
tmem57a	1.172619048	1.516778523
st8sia5	1.172588832	0.777777778
pgam2	1.172467858	1.402359109
dip2bb	1.172413793	0.488888889
celsr1b	1.172413793	0.56
si:dkey-172k15.4	1.172413793	1.222222222
pms1	1.172413793	1.25
lrrc59	1.172413793	1.318181818
tbc1d31	1.172413793	1.37037037
hax1	1.172297297	1.30952381
tcta	1.17218543	1.392156863
zbtb18	1.172161172	0.828703704
nbeaa	1.172131148	0.293478261
npb	1.172043011	0.908045977
vps72	1.171779141	0.832258065
tbc1d12a	1.171641791	0.664179104

glmp	1.171641791	1.154411765
trappc3	1.171597633	1.152905199
bcorl1	1.171428571	0.518518519
dacha	1.171428571	0.545454545
nyx	1.171428571	0.78125
ncf1	1.171428571	1.323529412
creb5a	1.171428571	1.434782609
vps25	1.171348315	1.282913165
foxk1	1.171296296	0.818584071
rab3c	1.171122995	0.474576271
il17rd	1.170940171	0.82300885
olig2	1.170940171	0.833333333
ankrd13c	1.170798898	0.601092896
lrrc4.1	1.170731707	0.22962963
cadm2b	1.170731707	0.725806452
rabepk	1.170731707	1.081632653
si:ch211-207i20.3	1.170731707	2.333333333
tmem205	1.170731707	1.163636364
azin1a	1.170542636	0.991525424
gabrr1	1.170454545	1.083333333
ercc3	1.17037037	1.098214286
ldlrb	1.170212766	1.1328125
ndufa1	1.170114943	0.964386129
ttbk1a	1.17	0.592105263
dcaf5	1.17	0.784482759
sqstm1	1.169902913	1.079831933
pom121	1.169811321	0.601941748
vezf1a	1.169811321	0.704545455
si:dkey-4c15.11	1.169811321	1
rnf103	1.169811321	1.688073394
emc1	1.169565217	0.798941799
eif4e2	1.169491525	1.178343949
slco5a2	1.169230769	0.45508982
plxna3	1.169230769	0.505050505
alg12	1.169230769	2.172413793
grid2	1.169014085	0.43902439
ccl44	1.169014085	1.03125
si:dkey-221j11.3	1.169014085	2.694915254
si:dkey-112a7.4	1.169014085	1.184313725
adnp2a	1.168831169	0.771929825
si:dkey-210j14.4	1.168831169	1.561643836
ndfip2	1.168582375	0.795744681
actr8	1.168421053	1.021276596
ptpro	1.168181818	0.40201005
cherp	1.168103448	0.611607143
pou2f1b	1.168067227	0.630434783
zgc:193812	1.168067227	1.118644068
mink1	1.168	0.894230769
ms4a17a.11	1.167938931	1.07311828
CABZ01074276.1	1.167938931	1.179104478
mfsd2ab	1.167883212	1.743055556
her4.2_1	1.167664671	1.897435897
tgfb3	1.167464115	1.061281337
otx5	1.167400881	0.386363636
brd3b	1.167213115	0.556701031
zgc:65894	1.166787527	0.4592646
tbr1b	1.166666667	0.296703297
myo9ab	1.166666667	0.49122807
bach2b	1.166666667	0.529411765
GSE1	1.166666667	0.625
amigo3	1.166666667	0.653846154

otx2	1.166666667	0.665271967
MAP3K11	1.166666667	0.666666667
egr2b	1.166666667	0.680851064
SMAD4 (1 of many)	1.166666667	0.686567164
dym	1.166666667	0.723076923
scxb	1.166666667	0.764705882
pdxkb	1.166666667	0.836734694
emp1	1.166666667	0.837837838
ttll11	1.166666667	0.875
si:ch73-111k20.3	1.166666667	0.906976744
itfg2	1.166666667	0.908045977
gipc3	1.166666667	0.942857143
zgc:153151	1.166666667	0.95
taf11	1.166666667	0.954545455
irak1bp1	1.166666667	0.973684211
znf1170	1.166666667	1.023809524
si:ch73-281f12.3p	1.166666667	1.09375
zgc:163098	1.166666667	1.108527132
pomk	1.166666667	1.125
mipep	1.166666667	1.151162791
zgc:171901	1.166666667	1.235294118
tmem184c	1.166666667	1.253968254
hs2st1a	1.166666667	1.307692308
SVIP	1.166666667	1.315789474
klhl11	1.166666667	1.46
irf3	1.166666667	1.5
tmem63a	1.166666667	1.5
znf982	1.166666667	1.6
rbfa	1.166666667	1.652173913
si:ch211-189a15.5	1.166666667	1.714285714
si:dkey-182i3.10	1.166666667	1.823529412
znf1001_2	1.166666667	1.833333333
cbx7a	1.166666667	1.846590909
hpcal4	1.166666667	1.916666667
rcn2	1.166666667	2.066666667
MTCL1	1.166666667	0.34375
slc18a3a	1.166051661	0.862190813
tspan3a	1.165966387	0.881104034
cdc123	1.165605096	1.509090909
rpl35a	1.165479231	0.890327914
march7_1	1.165441176	0.557017544
polr2b	1.165289256	0.635616438
rab3gap2	1.165289256	0.894736842
si:ch211-152p11.8	1.164948454	1.818181818
zgc:92113	1.164859002	0.713256484
cbl	1.164835165	0.56097561
jtb	1.164772727	1.204081633
tpt1	1.164758205	1.105206123
ap3s2	1.164658635	1.00990099
sox10	1.164556962	0.709876543
bnip1b	1.164556962	1.367088608
nptxrb	1.164473684	0.304964539
cx35.4	1.164473684	1.390243902
nup50	1.164444444	0.576419214
rps24	1.164404223	1.013852048
stox2b	1.164383562	0.666666667
epb41l4b	1.164383562	1.276923077
polr2f	1.164351852	1.265306122
nedd9	1.164285714	0.516339869
VPS37B (1 of many)	1.164179104	1.046875
gpr85	1.163934426	0.314606742

mycbp2	1.163934426	0.677852349
tmem222a	1.163934426	0.943548387
ATP5SL	1.163934426	1.5
agfg1b	1.163793103	0.976190476
cyb5d2	1.163636364	0.861538462
cox19	1.163636364	1.191176471
snn	1.163636364	1.204545455
amer2	1.163522013	0.515873016
lactb	1.163461538	0.79047619
zeb2b	1.163265306	0.322222222
b3galt6	1.163265306	0.795454545
thoc5	1.163157895	0.813953488
camta1a	1.163043478	0.349206349
cinp	1.163043478	1.333333333
trim47	1.162962963	1
celf5a	1.162790698	0.282051282
si:dkey-31f5.11	1.162790698	0.351351351
enox1	1.162790698	0.621621622
carkd	1.162650602	0.732484076
cfhl4	1.162522202	1.305752561
kdm4c	1.1625	1.269230769
asap1b	1.162393162	0.505263158
ntrk3a	1.162162162	0.423076923
nr2c2ap	1.162162162	1.621621622
kxd1	1.162162162	1.915254237
mt-co2	1.16207554	1.146930606
fbxo28	1.161931818	1.131578947
slc27a2a	1.161915621	1.002727273
nrxn2a	1.161904762	0.2
fam222ba	1.161904762	0.642857143
pip5k1ca	1.161764706	0.510638298
vps18	1.161676647	1.224137931
WDR83OS	1.161473088	1.116883117
rimbp2_1	1.161290323	0.55
med13b	1.161290323	0.689655172
fam69ab	1.161290323	0.862068966
B3GALT1	1.161290323	1.146341463
si:dkey-182i3.8	1.161290323	1.5625
faxdc2	1.161202186	1.309210526
si:ch73-105m5.1	1.160839161	1.146853147
atp5e	1.160760588	0.982433312
stxbp5a	1.160714286	0.386363636
hoxb2a	1.160714286	0.543859649
zgc:101783	1.160714286	1.036363636
polr1e	1.160714286	1.12195122
rpl37_1	1.160714286	1.476190476
nit2	1.160621762	1.326086957
slc35g2a	1.160427807	0.916666667
csrp2bp	1.160377358	0.791666667
im:6907928	1.160377358	0.86
stard10	1.160377358	1.098901099
fbxo3	1.16	1.220858896
otx1a	1.16	0.424242424
pdpk1b	1.16	0.736401674
si:ch211-241d24.3	1.16	1
otud4	1.16	1.083333333
si:dkey-57k2.7_2	1.16	1.264150943
nae1	1.16	1.267100977
zgc:123217	1.16	1.302325581
hey2	1.16	1.326530612
trappc4	1.16	1.634854772

socs3b	1.159663866	0.518518519
CABZ01090374.1	1.159609121	1.080701754
si:ch1073-82k10.1	1.159574468	0.909090909
crygm2d17	1.159545777	0.881396881
zfyve16	1.15942029	0.984375
rps15a	1.159396789	0.916347669
r3hcc1l	1.159340659	1.391566265
tmx1	1.159235669	1.357615894
ADGRL3 (1 of many)	1.159090909	0.542857143
sdk1a	1.159090909	0.627906977
si:dkey-242h9.3	1.159090909	1.019230769
si:ch211-148l7.4	1.159090909	1.282051282
ankmy2a	1.159090909	1.372093023
zgc:194312	1.159090909	1.5
ccar1	1.158878505	0.755555556
camlg	1.158823529	0.861702128
tomm7	1.158774373	1.14479638
rims1a	1.158730159	0.333333333
agap1	1.158730159	0.605442177
si:ch211-232i5.3	1.158730159	0.890625
RNF31	1.158730159	1.282608696
lhx2b	1.158536585	0.415384615
apba1b	1.158536585	0.538461538
gsk3aa	1.158415842	0.619047619
f7	1.158415842	0.894308943
ncoa6	1.158333333	0.744444444
pigx	1.158333333	1.208633094
si:ch73-12o23.1	1.158273381	0.80625
rfxap	1.158227848	1.121405751
slc43a2b	1.15821501	1.046945531
cry-dash	1.15815486	1.103146853
shank3b	1.157894737	0.548387097
FO681288.1	1.157894737	0.9
CABZ01067151.1	1.157894737	0.923076923
mfsd6l	1.157894737	1.095238095
slc30a1b	1.157894737	1.105263158
serpine3	1.157894737	1.129032258
plrdgb	1.157894737	1.153846154
fam26e.1	1.157894737	1.206896552
nipa1	1.157894737	1.3125
RFX7 (1 of many)	1.157894737	0.458333333
rfx4	1.157894737	0.542857143
dazap2	1.157844991	0.726923077
elp5	1.157303371	1.450704225
si:ch73-70c5.1	1.157142857	1.01369863
slx1b	1.157142857	1.395833333
whsc1l1	1.157024793	0.568181818
tmem50a	1.157014157	1.540372671
bloc1s2	1.156976744	1.358974359
gstm.2	1.156862745	0.76744186
spsb3a	1.156862745	0.930232558
grhpra	1.156862745	1.158878505
rnf207b	1.156862745	1.303571429
si:rp71-1k13.6	1.156862745	1.651162791
tenm1	1.156626506	0.633333333
cnpy2	1.156626506	1.121621622
slc36a4	1.156521739	1.438202247
tnni2a.4	1.156421023	1.026091744
guca1c	1.156398104	1.455958549
CABZ01078858.1	1.156351792	0.926267281
srrm1	1.15625	0.692307692

blzf1	1.15625	0.808743169
DHX30	1.15625	0.9
PEX5L (1 of many)	1.15625	1.058823529
zgc:92518	1.15625	1.111111111
zcchc17	1.156069364	1.202531646
bcor	1.156028369	0.584745763
si:dkey-89b17.4	1.156	0.632850242
dusp6	1.155747837	1.036319613
fam21c	1.155688623	0.775362319
rps25	1.155560783	0.866185366
hnrnpa3	1.155555556	0.372727273
GSK3B (1 of many)	1.155555556	0.653846154
zfyve26	1.155555556	0.926829268
si:ch211-129c21.1	1.155555556	1.241758242
tmem206	1.155555556	1.394736842
parp8	1.155172414	0.454545455
si:ch73-174h16.4	1.155172414	1.145833333
micu3a	1.155172414	1.395833333
gpcpd1	1.155102041	1.311440678
atp6v0b	1.155063291	1.064035088
nipbla	1.15503876	0.765306122
herpud2	1.15503876	0.843065693
cdc16	1.154929577	1
mat2ab	1.154891304	1.450236967
si:ch211-209m20.7	1.154882155	1.266272189
gripap1	1.15483871	1.442477876
kdm4b	1.154791155	0.580976864
robo1	1.154761905	0.493055556
pde6h	1.154661017	1.054563998
dnm1l	1.154639175	0.842406877
RPL37A	1.154599724	0.88146653
arnt2	1.154545455	0.68452381
cwf19l1	1.154471545	0.893939394
psmd5	1.154411765	0.905660377
snap91	1.154205607	0.215189873
trim8b	1.154185022	0.641509434
taco1	1.154185022	0.978494624
ewsr1a	1.154135338	0.650663943
xrcc3	1.153846154	0.790697674
rtn4r	1.153846154	0.793814433
eny2	1.153846154	0.908713693
nfybb	1.153846154	1.123778502
hoxa9b	1.153846154	1.191489362
SRBD1	1.153846154	1.28125
zgc:173575	1.153846154	1.515151515
galnt8b.1	1.153846154	0.555555556
rnaset2l	1.153846154	0.611111111
rasip1	1.153846154	0.666666667
cyp2aa7	1.153846154	0.68
si:ch211-238e22.4	1.153846154	0.769230769
olig4	1.153846154	0.807692308
dram2b	1.153846154	0.868686869
qser1	1.153846154	0.941176471
mkl1a	1.153846154	1
slc8a2a	1.153846154	1
zgc:194659	1.153846154	1.014492754
si:ch73-158p21.3	1.153846154	1.363636364
si:dkey-16p6.1_8	1.153846154	1.363636364
ARAP2	1.153846154	1.666666667
zgc:110249	1.153846154	1.833333333
si:dkey-1h6.8	1.153846154	2.083333333

si:ch211-214j24.9	1.153688525	0.211586902
dpy30	1.153535354	1.184534271
clip3	1.153465347	0.32885906
cntn2	1.153333333	0.314606742
zgc:165653	1.153225806	1.291044776
nrxn1a	1.153153153	0.369318182
tmem167a	1.153125	1.118686869
cyp26a1	1.153061224	1.287671233
ppp1r37	1.153005464	0.817142857
celf3a	1.152882206	0.333333333
hmx4	1.152838428	1.041152263
nudcd3	1.15282392	1.139837398
tmem240a	1.152777778	0.620689655
prdm4	1.152777778	0.686567164
six4b	1.152777778	0.722222222
fbxw2	1.152777778	0.901840491
tmed5	1.152777778	1.347826087
slc25a12	1.152680653	1.035236938
raver2	1.152542373	0.583333333
rev1	1.152542373	0.705882353
supt20	1.152542373	0.816326531
zgc:165656	1.152542373	0.82
serac1	1.152542373	1.174603175
polr2c	1.152542373	1.244604317
tbpl1	1.152542373	1.440677966
smg7	1.15234375	0.723004695
si:ch211-26b3.4	1.152173913	0.264705882
pou2f2a_1	1.152173913	0.294117647
si:ch211-188i4.4	1.152173913	0.638888889
agps	1.152173913	0.739583333
si:ch73-111k22.2	1.152173913	0.873333333
si:ch211-241j12.3	1.152173913	1.113636364
map4k4	1.152091255	0.528455285
tmem256	1.152046784	1.223350254
foxq1a	1.151943463	1.421052632
ssbp3b	1.151869159	0.623529412
ephb4a	1.151785714	0.906976744
sik2b	1.151639344	0.79638009
vma21	1.151558074	1.070038911
ift81	1.151515152	1.894736842
ints8	1.151260504	1.397849462
smoc2	1.151162791	0.849056604
rnf11a	1.151162791	1.338235294
man2b1	1.151041667	1.053892216
si:ch1073-357b18.4	1.150943396	0.636363636
si:dkeyp-104h9.5	1.150943396	1.066666667
si:dkey-108k21.7	1.150943396	1.125
twist3	1.150943396	1.25
si:ch73-109d9.2	1.150943396	1.351648352
cnksr2b	1.150684932	0.261538462
kcnn3	1.150684932	0.734375
zgc:171679	1.150684932	1.425925926
dip2ca	1.150537634	0.604395604
tnfrsf21	1.150537634	0.686746988
iars	1.150306748	0.869565217
magi1b	1.150289017	0.524137931
npdc1a	1.150289017	0.624338624
hlfa	1.150093809	0.874551971
plppr4a	1.15	0.240740741
glipr1b	1.15	0.449275362
exoc7	1.15	0.858156028

tmem184bb	1.15	0.407407407
stxbp5l	1.15	0.511627907
pmepa1	1.15	0.694444444
abca5	1.15	0.953125
si:ch211-253b8.2	1.15	1
ftr24	1.15	1.206896552
mlycd	1.15	1.324324324
znf1059	1.15	1.333333333
atp2a1l	1.14971573	1.097247338
ptdss1a	1.149700599	0.935251799
entpd5b	1.14957265	1.160305344
setd1a	1.149425287	0.75
si:dkey-100n23.3	1.149425287	1.265060241
btaf1	1.149122807	0.760869565
trappc12	1.149122807	0.864197531
sez6b	1.14893617	0.269230769
fastkd3	1.14893617	1.314606742
cc2d1b	1.14893617	1.384615385
eef2b	1.148925112	0.824592443
ppm1aa	1.148760331	0.820224719
glcea	1.148648649	0.759259259
surf1	1.148648649	1.363247863
rab1bb	1.148648649	1.44
katnbl1	1.148648649	1.786885246
smarcc2	1.148514851	0.655913978
sst1.1	1.148514851	0.764705882
pxylp1	1.148514851	0.844444444
jarid2a	1.1484375	0.578431373
lamtor2	1.148255814	1.07635468
FP103009.1	1.148148148	0.346666667
rapgef1b	1.148148148	0.803030303
si:ch211-177d9.1	1.148148148	0.666666667
scaper	1.148148148	0.779069767
CABZ01065103.1	1.148148148	0.788888889
slc4a11	1.148148148	0.814814815
zgc:64022	1.148148148	1.208333333
ogfrl1	1.148148148	1.25
si:dkey-76k16.5	1.148148148	1.636363636
aamdc	1.148148148	1.722222222
si:ch73-252p3.1	1.148148148	4
wdfy3	1.147727273	0.555555556
zgc:112962	1.147727273	1.684931507
gsr	1.147651007	1.345794393
ciapin1	1.147601476	1.50390625
zgc:113142	1.147540984	2.192307692
TPP2	1.147368421	0.90625
gna11b	1.147286822	0.936842105
mgat4a	1.147239264	0.910344828
si:dkey-226k3.4	1.147239264	1.010526316
slc1a4	1.147169811	0.715083799
ssu72	1.147126437	1.137681159
glula	1.147088608	1.199228172
SAMD14	1.147058824	0.229166667
ttbk2a	1.147058824	0.458333333
elk4	1.147058824	0.76
NATD1 (1 of many)	1.147058824	0.775
MGAT3 (1 of many)	1.147058824	1.045454545
si:dkeyp-110g5.4	1.147058824	1.219512195
bmp6	1.147058824	1.34375
scly	1.147058824	1.615384615
gemin8	1.146892655	1.38961039

si:dkey-42i9.4	1.146766169	1.228782288
foxj2	1.146666667	0.777777778
trim23	1.146666667	1.084507042
zfp36l1b	1.146387833	0.842639594
fmn2b	1.146341463	0.309859155
si:dkey-121a11.3	1.146341463	0.697674419
atp8b3	1.146341463	0.735294118
polh	1.146341463	0.801980198
si:ch211-180m24.1	1.146341463	1.421052632
saga	1.146245059	0.752873563
rhoub	1.146131805	1.234482759
ext2	1.146103896	1.09929078
fcf1	1.146031746	1.338762215
rbpjb	1.145833333	1.075
tmem110	1.145833333	1.108695652
sgce	1.145833333	0.619596542
onecut1	1.145631068	0.34939759
ankrd27	1.145454545	0.980769231
snai2	1.145454545	1.056291391
COA4	1.145454545	1.17679558
CR762469.1	1.145454545	1.393939394
fam168a	1.145374449	0.543046358
mcl1a	1.145360825	1.145638629
rbfox3a	1.145299145	0.3
syngap1a	1.14516129	0.277777778
si:ch211-239j9.1	1.14516129	0.830508475
tmem67	1.14516129	0.96
APBA3	1.14516129	1.051724138
XPO5	1.144927536	0.564516129
ubr4	1.144927536	0.84
rtn4ip1	1.144859813	0.940092166
ATP11B	1.144827586	1.083333333
lgals2b	1.144778481	1.10515873
rps17	1.144665948	0.916190101
pnpla8	1.144654088	1.068493151
ulk2	1.144615385	0.679758308
rnf130	1.144578313	0.636363636
neurod4	1.144475921	0.784126984
tubg1	1.144444444	0.664835165
lsm7	1.144385027	0.960960961
stx1b	1.144316731	0.246935201
mt-co3	1.144303778	0.99010461
otub1b	1.144278607	1.15942029
nkx6.2	1.144144144	0.533333333
msx2b	1.144144144	1.702970297
pcdh1a	1.143939394	0.337078652
minos1	1.143742255	1.006681514
atg5	1.143678161	1.291139241
sppl3	1.143646409	0.596590909
g6pca.2	1.143578239	1.628618241
ctsc	1.143564356	1.02
rpl12	1.143542309	1.06791704
ddx47	1.143518519	0.652719665
gapdh	1.143337066	0.990045165
gphnb	1.142857143	0.288461538
plppr3b	1.142857143	0.381443299
e2f7	1.142857143	0.666666667
glg1b	1.142857143	0.694444444
plcg1	1.142857143	0.73964497
sp7	1.142857143	0.793103448
zgc:114041	1.142857143	0.80952381

si:dkey-238k10.2	1.142857143	0.833333333
slc6a14	1.142857143	0.833333333
dhx34	1.142857143	0.875
adamts6	1.142857143	0.909090909
NFATC2 (1 of many)_2	1.142857143	0.909090909
ict1	1.142857143	0.936170213
fsd1	1.142857143	0.966666667
si:cabz01021433.2	1.142857143	1.0625
si:ch211-246m6.5_1	1.142857143	1.103448276
SLC25A5 (1 of many)	1.142857143	1.138888889
galns	1.142857143	1.367346939
AGBL1	1.142857143	1.384615385
chic1	1.142857143	1.426966292
foxd3	1.142857143	1.432432432
abi1b	1.142857143	0.339285714
klhl14	1.142857143	0.42
skor1b	1.142857143	0.488372093
nos1apb	1.142857143	0.5
fam57ba	1.142857143	0.522292994
socs7	1.142857143	0.56043956
trim24	1.142857143	0.58
dnajb4	1.142857143	0.61627907
si:cabz01029535.1	1.142857143	0.75
HECTD4	1.142857143	0.84
sacm1lb	1.142857143	0.842975207
ankib1b	1.142857143	0.845360825
txndc11	1.142857143	0.88372093
cep97	1.142857143	0.891304348
pum2	1.142857143	0.918032787
rdh1	1.142857143	1.040160643
si:dkeyp-122d12.1	1.142857143	1.047619048
smim15	1.142857143	1.146687697
ryroxd1	1.142857143	1.191780822
cnot7	1.142857143	1.304347826
si:dkey-149i7.2	1.142857143	1.375
SLC45A4	1.142857143	1.4
prtfdc1	1.142857143	1.472222222
zdhhc12b	1.142857143	1.84
si:dkey-6o1.2	1.142857143	2.8
crebbpb	1.142857143	0.71875
gnptg	1.142857143	1.472392638
CPLX1 (1 of many)	1.142086331	0.386363636
alg9	1.142045455	0.951871658
mthfd1l	1.141935484	0.787023977
ctsd	1.14180791	1.128996692
rereb	1.141791045	0.512195122
pcdh7a	1.141666667	0.371134021
tspo	1.141509434	0.974889217
mrrf	1.141509434	1.100628931
wbp11	1.141463415	0.693181818
sh3rf1	1.141414141	0.943661972
suco	1.141304348	0.81875
zmym4	1.141176471	0.7625
mms19	1.141176471	0.864197531
ccnb3	1.141176471	1.150537634
btg1	1.141099855	0.758165393
pfdn4	1.140921409	1.233766234
tlx2	1.14084507	0.642857143
tns2b	1.14084507	1.333333333
tspan36	1.140776699	1.1984
ppme1	1.140728477	1.105454545

insm1a	1.140625	0.612149533
tktb	1.140609636	0.727272727
gnal2	1.140495868	0.691176471
glrx5	1.140439932	1.358609795
spry4	1.140410959	0.766990291
elf2b	1.140350877	0.741935484
usp9	1.140350877	0.779264214
dgkh	1.140350877	0.828571429
cdyl	1.140350877	1.222222222
ago4	1.140221402	0.655813953
fnip1	1.140186916	0.825242718
mt-cyb	1.140071908	1.115891109
si:dkey-248g15.3	1.140032949	1.235046589
gabrb4	1.14	0.294117647
lrp11	1.14	0.678571429
CABZ01080370.1	1.14	0.840909091
cdh27	1.14	1.252173913
myoz2a	1.139966273	1.154875717
scocb	1.139841689	0.844086022
CABZ01114051.1	1.139784946	0.779069767
atraid	1.139784946	1.505376344
sec61g	1.139678616	0.871917263
rbm15	1.13963964	0.995327103
ubl7a	1.139534884	0.816666667
ipp	1.139534884	0.825
ftr86	1.139534884	1.131147541
si:ch211-246m6.4	1.139534884	1.238095238
L3HYPDH	1.139534884	1.339285714
ids	1.139534884	1.416666667
hykk.2	1.139534884	1.611111111
SMIM18	1.139344262	1.166666667
patl1	1.139318885	0.761755486
waca	1.139275766	0.758007117
eef1e1	1.139275766	1.283387622
ZNF609 (1 of many)	1.139240506	0.637795276
siae	1.139240506	1.275862069
tm2d3	1.139158576	1.045751634
las1l	1.139130435	1.105882353
mibp2	1.139058761	1.066631016
ginm1	1.138943249	1.567226891
ndufa3	1.138925295	0.965449161
RPL41	1.138918719	0.946874444
rusc2	1.138888889	0.731034483
FO704813.1	1.138888889	0.761904762
atp13a2	1.138888889	1.192307692
rps21	1.138767291	0.925405823
znf292b	1.138613861	0.522727273
arxa	1.138613861	0.736842105
si:ch211-161c3.5	1.138461538	0.4
mapk7	1.138461538	0.75
gosr2	1.13836478	1.047745358
cops5	1.138297872	1.510324484
si:dkey-286j15.1	1.138263666	1.516129032
CABZ01084963.1	1.138240575	0.807874016
rxrbb	1.138095238	1.121212121
pcnxl3	1.137931034	0.80952381
eif2ak3	1.137931034	0.898550725
GIGYF1 (1 of many)	1.137931034	0.953846154
si:ch1073-325m22.2	1.137886598	1.291862285
ddx1	1.137795276	1.055555556
dhrs7	1.137704918	1.067092652

hsbp1a	1.137670197	1.14849921
ptprub	1.137614679	0.516483516
uqcrq	1.13755796	0.990070093
tnksb	1.137546468	0.659292035
ccdc28b	1.1375	1
plxnb3	1.137254902	0.852941176
mecp2	1.137254902	0.882352941
rabep2	1.137254902	1.107843137
uvssa	1.137254902	1.111111111
si:ch211-253b8.5	1.137254902	1.169811321
birc2	1.137142857	0.84180791
parp6a	1.136986301	0.631578947
agap2	1.136842105	0.260869565
cltcb	1.136801541	0.583333333
FAM57A	1.136752137	0.896
kansl1a	1.13671875	0.716216216
cox20	1.136645963	1.267241379
tbk1	1.136563877	0.808219178
zzz3	1.136363636	0.638655462
nrd1b	1.136363636	1.2
c2h1orf27	1.136363636	1.232142857
ube3c	1.136363636	1.490196078
cnpy4	1.136363636	1.588235294
hoxc12b	1.136363636	0.625
pnoca	1.136363636	1.15
ccdc30	1.136363636	1.176470588
nrros	1.136363636	1.307692308
dok1a	1.136363636	2.333333333
caly	1.13618677	0.646153846
rnf175	1.136111111	1.189189189
glyr1	1.136069114	1.120967742
slc44a1a	1.136054422	1.294117647
sorl1	1.136	0.808080808
CR559945.1	1.13592233	1.423529412
gas2l1	1.135802469	0.983870968
sympk	1.135678392	0.760273973
SLITRK3 (1 of many)	1.13559322	0.2
abca2	1.13559322	0.574468085
ror2	1.13559322	0.865671642
smchd1	1.13559322	1.056603774
mettl5	1.13559322	1.380952381
brd4	1.135483871	0.532467532
cldn7b	1.135470527	1.541019956
rnf6	1.135416667	0.80952381
rfwd2	1.135416667	0.9875
casc4	1.135371179	0.741293532
gbf1	1.135135135	0.743902439
rps23	1.135004492	0.922986906
nptx1l	1.134969325	0.42962963
fgfr1op2	1.134969325	1.254901961
snx3	1.134939759	1.3359375
plekha5	1.134751773	0.691666667
slc8a2b	1.134615385	0.52
nfatc1	1.134615385	0.782608696
sclt1	1.134615385	1.295454545
atp8a2	1.134502924	0.587209302
dhrs3a	1.134453782	0.957746479
scmh1	1.134328358	0.615384615
MEGF9 (1 of many)_2	1.134328358	0.673076923
pane1	1.134328358	0.776470588
CABZ01102039.1	1.134328358	1

si:dkey-172k15.5	1.134328358	1.157894737
selk	1.134259259	1.08
nlgn3b	1.134228188	0.254385965
rab40c	1.134199134	0.759433962
taf6l	1.134146341	0.85
C2CD2	1.134146341	1.015873016
ppp1r3cb	1.134146341	1.14619883
ccdc85cb	1.134146341	1.353846154
CRELD1	1.134020619	0.64516129
elovl6l	1.134020619	2.15625
aldh9a1a.1	1.133936081	1.02340234
CABZ01084603.1	1.133928571	0.725274725
bbs2	1.133858268	0.85
ubqln4	1.133580705	0.705769231
CABZ01108998.1	1.133574007	0.833333333
zgc:63587	1.133466135	1.200956938
sall2	1.133333333	0.352941176
lingo2a	1.133333333	0.362068966
lrrc38b	1.133333333	0.486486486
bahd1	1.133333333	0.759259259
accs	1.133333333	0.790697674
si:ch211-127b16.1	1.133333333	0.823529412
fam46d	1.133333333	0.833333333
pi4k2a	1.133333333	0.882352941
hmgxb4a	1.133333333	0.9
rabggta	1.133333333	0.921875
ctdp1	1.133333333	0.927272727
chm	1.133333333	0.935135135
fancd2	1.133333333	0.963636364
smg6	1.133333333	0.966666667
slc7a3a	1.133333333	0.996784566
DIRAS2	1.133333333	1.166666667
hmbox1b	1.133333333	1.2
casp9	1.133333333	1.20212766
slc23a3	1.133333333	1.25
cgrrf1	1.133333333	1.256410256
tti1	1.133333333	1.296296296
si:dkey-153k10.6	1.133333333	1.298245614
si:ch211-161m3.6	1.133333333	1.307692308
si:dkey-3l5.2	1.133333333	1.8125
rad51d	1.133333333	2.133333333
mhc1zba	1.133126935	2.450549451
rps14	1.132964833	0.95214203
si:ch73-367p23.2	1.132883642	1.152045504
tcf7l1b	1.132867133	0.765625
fbxo7	1.1328125	0.763975155
smg9	1.132743363	1.177083333
nrd1a	1.132701422	0.842105263
txndc17	1.132653061	1.775167785
otud6b	1.132653061	1.461538462
ikbkap	1.13253012	0.927536232
nrxn3b	1.132352941	0.375
slc6a13l	1.132352941	1.207920792
znf1156	1.132352941	1.613636364
kdm6a	1.132231405	0.618644068
diabloa	1.132231405	2.414893617
papola	1.132113821	0.866050808
tmem119a	1.132075472	1.730769231
trip6	1.131868132	0.586538462
bmp1a	1.131805158	0.993355482
si:ch211-25g7.5	1.131782946	2.08

rps7	1.131722591	1.029883729
map3k9	1.131578947	0.35483871
steap2	1.131578947	0.608695652
polr2m	1.131578947	0.7
fam131ba	1.131578947	0.769230769
kaznb	1.131578947	0.866666667
rnmtl1a	1.131578947	0.884615385
zwilch	1.131578947	1
si:dkey-262k9.4	1.131578947	1.212121212
si:dkey-93n13.2	1.131578947	1.3125
map4k6	1.131578947	1.46875
foxp2	1.131386861	0.290598291
adgrb1a	1.131313131	0.308823529
smarcb1b	1.131313131	0.981818182
prps1b	1.131313131	1.31443299
rx3	1.131147541	0.711111111
si:dkey-16m19.1_1	1.131147541	0.820224719
vps54	1.131147541	0.826923077
si:ch73-389k6.1	1.131147541	0.833333333
dclre1c	1.131147541	1.267857143
crfb6	1.131147541	1.965986395
mplkip	1.131147541	0.901554404
naa20	1.131121643	1.054775281
nrsn1	1.131054131	0.570552147
znf385b	1.130952381	0.772727273
gpank1	1.130952381	0.894736842
si:dkey-19b23.8	1.130952381	1.032967033
nfkbie	1.130841121	0.859504132
rps20	1.13077808	1.013517394
rps18	1.130615851	1.020662413
abca3b	1.130597015	0.959016393
TMEM14A	1.130555556	1.070588235
brat1	1.130434783	0.755555556
zgc:113423	1.130434783	0.846153846
phf21ab	1.130434783	0.966666667
si:zfos-464b6.2	1.130434783	1.196078431
si:dkey-16p19.5_1	1.130434783	1.470588235
ankdd1a	1.130434783	1.5
crp2	1.130434783	1.515151515
fbxl16	1.130434783	0.493975904
clasp2	1.130434783	0.55186722
ppp2r5cb	1.130434783	0.926380368
alkbh4	1.130434783	1.253968254
prr12a	1.13	0.569620253
zgc:193541	1.129971876	0.988237329
chrnb2b	1.12987013	0.5
lpar2a	1.12987013	0.886363636
thrb	1.12987013	1.459016393
zgc:123105	1.129798903	0.768292683
usp2b	1.129770992	1.08
adgrl3.1	1.12972973	0.315068493
atp5ib	1.129665536	1.065622989
mfsd12a	1.12962963	1.285714286
ap5b1	1.12962963	1.692307692
rpl34	1.129538763	0.99940423
dclk1b	1.129411765	0.304347826
l2hgdh	1.129411765	0.921787709
cln6a	1.129411765	1.255102041
chmp7	1.129337539	1.126182965
smad7	1.129251701	0.773972603
polr3b	1.129032258	1.053435115

KCNAB3	1.129032258	0.315789474
syne1a	1.129032258	0.538461538
gprc5bb	1.129032258	0.689655172
l3mbtl1b	1.129032258	0.8
ubr3	1.129032258	0.872727273
znf384l	1.129032258	0.895833333
CABZ01021450.3	1.129032258	0.966666667
wu:fj64h06	1.129032258	0.966666667
sdhaf1	1.129032258	1.052631579
pkmyt1	1.129032258	1.193548387
irak1	1.129032258	1.318181818
wnt9b	1.129032258	1.333333333
dcbld2	1.129032258	1.421686747
TOM1L2	1.129032258	1.666666667
znf1168	1.129032258	1.833333333
zrsr2	1.129032258	1.857142857
ptprk	1.128834356	0.713333333
zbtb14	1.128787879	0.909774436
c8a	1.128742515	0.829321663
timm10	1.128676471	0.924561404
adrm1	1.12863706	0.787401575
nlgn3a	1.128571429	0.488372093
trmt10c	1.128571429	0.814814815
irg1l	1.128571429	0.971153846
rad17	1.128571429	1.254545455
rplp1	1.128559594	1.030849875
tcnl	1.128397376	1.225749559
anxa3b	1.128378378	1.423529412
DGKK	1.128205128	0.583333333
si:dkey-202p8.1	1.128205128	0.595238095
ACSF3	1.128205128	0.636363636
kcnk1b	1.128205128	1.05
DTX4 (1 of many)	1.128205128	0.490196078
pds5b	1.128205128	0.55186722
mxd1	1.128205128	1.010309278
crygm2d9	1.128001715	0.952554745
shdb	1.128	0.920353982
tapt1b	1.128	1.261904762
hdx	1.127906977	0.53164557
s1pr5a	1.127906977	1.711864407
cntnap3	1.127659574	0.45
man1b1a	1.127659574	0.837209302
trafd1	1.127659574	0.887323944
alpk2	1.127659574	1.341463415
FBXO48 (1 of many)_1	1.12745098	1.428571429
gabarapl2	1.127358491	1.118181818
tcf7l1a	1.127272727	0.78
dlx1a	1.127272727	0.46
osbpl11	1.127272727	0.955555556
amacr	1.127272727	0.968253968
disp1	1.127272727	1.325581395
pggt1b	1.127192982	0.77238806
asphd1	1.127118644	1.035714286
gpr22a	1.126984127	0.972222222
cwc27	1.126984127	1.627659574
wdr45	1.126923077	1.148550725
sub1b	1.126888218	1.039735099
asap2a	1.126760563	0.633333333
ift52	1.126760563	1.274647887
pcnxl2	1.126582278	0.363636364
ppp2r5b	1.126582278	0.446280992

setdb1b	1.126582278	0.585034014
foxc1a	1.126582278	0.769230769
nfatc2ip	1.126582278	1.23943662
tmem106c	1.126436782	1.070588235
rdh10b	1.126436782	1.530612245
gpr75	1.126315789	0.373333333
nt5dc1	1.126315789	1.046153846
abhd13	1.126126126	0.969072165
ube2ka	1.126099707	1.148484848
rab15	1.12605042	1.299145299
ppp3cb	1.12596401	0.453703704
tfg	1.125886525	0.731466227
AC024175.8	1.125842697	1.135231317
cbx2	1.125786164	0.962025316
cant1a	1.125748503	0.877300613
uchl5	1.125531915	1.333333333
sst3	1.125490196	1.035714286
RIMBP2 (1 of many)	1.125	0.466666667
rapgef2	1.125	0.503816794
cacng7a	1.125	0.578947368
samd7	1.125	0.735294118
pde4bb	1.125	0.75
ZBTB26	1.125	0.833333333
lyst	1.125	0.837837838
lzts2b	1.125	0.947368421
fam110c	1.125	1.130434783
nrde2	1.125	1.142857143
si:dkey-117n7.4	1.125	1.352941176
cdh4	1.125	0.323943662
msl2b	1.125	0.585034014
asic1a	1.125	0.727272727
MARK1	1.125	0.805555556
slc45a2	1.125	0.848404255
b4gat1	1.125	0.88
dhrs7ca	1.125	0.904761905
zgc:73226	1.125	0.90625
shrprbck1r	1.125	1
cfap20	1.125	1.035211268
plpp5	1.125	1.040909091
pknox1.1	1.125	1.046153846
vwa9	1.125	1.095238095
znf1149	1.125	1.333333333
CR391916.1	1.125	1.333333333
ccpg1	1.125	1.333333333
tex264a	1.125	1.35483871
churc1	1.125	1.461538462
uba3	1.125	1.527363184
fbxo4	1.125	2.4
fgf23	1.125	2.5
myom2a	1.124735729	0.96884058
tusc3	1.124600639	1.062893082
exoc2	1.124567474	1
fam117ba	1.124497992	0.281437126
scamp3	1.124423963	0.748837209
zgc:55461	1.124413146	0.995708155
zgc:66427	1.124352332	0.945544554
psmd9	1.124352332	1.28125
arl4ab	1.124223602	1.426829268
rpl19	1.124171646	0.936901763
pabpc1b	1.124124124	0.819845857
gpatch1	1.124031008	0.867256637

necab3	1.123966942	1.036363636
cryl1	1.123853211	1.100840336
crygm2d4	1.123823398	0.864788732
nck1a	1.123762376	0.990566038
hgsnat	1.123595506	0.959302326
nol4lb	1.12345679	0.491803279
sec24c	1.123376623	0.801526718
vwa8	1.123287671	1.017857143
aebp1	1.123188406	1.0625
si:dkey-33c12.3	1.123134328	0.836633663
abcc8	1.123076923	0.526315789
ggact.2	1.12295082	1.178217822
tbca	1.12295082	1.296666667
blvrb	1.12295082	1.3203125
arrb2a	1.122905028	1.330985915
nr0b2a	1.122881356	1.264705882
ormdl2	1.122807018	1.462962963
fzd3a	1.122641509	0.532608696
efhd1	1.122615804	1.141732283
desi2	1.122580645	0.911290323
IQSEC1 (1 of many)	1.12244898	0.657142857
glod4	1.122137405	0.91954023
mxd3	1.122137405	1.05511811
mterf3	1.122137405	1.086956522
znf526	1.12195122	0.777777778
shprh	1.12195122	0.928571429
snrnp35	1.12195122	1.1875
BAALC	1.12195122	1.275
CABZ01079258.1	1.12195122	2.485714286
nkain1	1.121848739	0.537190083
kat5a	1.12173913	0.971153846
shisa3	1.121621622	0.659090909
osr2	1.121621622	0.768115942
mtor	1.121621622	0.827272727
ubxn6	1.121546961	1.210355987
pik3ca	1.121495327	1.071428571
snap23.1	1.121468927	1.397887324
rpl36a	1.121426466	0.922086425
si:ch211-195b15.8	1.121330724	0.8
ndufab1a	1.12132089	1.205601093
dscaml1	1.121212121	0.342105263
bicd1a	1.121212121	0.448275862
zgc:100846	1.121212121	0.96
nfia	1.121212121	0.571428571
scpep1	1.121212121	0.965250965
itih3a	1.121212121	1.123966942
parp16	1.121052632	1.093567251
adam23a	1.121019108	0.478813559
si:ch211-66e2.5	1.121019108	0.625
fundc2	1.120996441	1.122905028
LMAN2L (1 of many)	1.120879121	0.971428571
MEPCE (1 of many)	1.120879121	0.983425414
myt1la	1.120689655	0.274647887
trioa	1.120689655	0.514563107
slc25a14	1.120689655	1.010638298
coq10b	1.120541205	1.576523031
htt_1	1.120481928	0.677419355
pelp1	1.120437956	0.739837398
caprin1b	1.120414673	0.994137353
epb41l4a	1.12037037	0.598360656
scg2a	1.12037037	0.855491329

nme2b.1	1.120319813	0.975223309
lrrc8db	1.120300752	1.311111111
tmem59l	1.120291616	0.564917127
dlat	1.120283019	1.060402685
cnppd1	1.120253165	0.931106472
TENM2 (1 of many)_1	1.12	0.25
pcbp4	1.12	0.473684211
wrap53	1.12	0.857142857
siva1	1.12	0.932926829
herpud1	1.12	1.079545455
sp9	1.12	1.14
zbtb46	1.12	0.632653061
phf21b	1.12	0.717948718
LRRC75B (1 of many)	1.12	0.844444444
si:ch211-196h16.4	1.12	0.909090909
fktn	1.12	0.961038961
si:dkey-49n23.1	1.12	0.975308642
tmprss9	1.12	1
tbc1d20	1.12	1.042857143
si:ch211-218d20.15	1.12	1.203703704
ankrd46b	1.119834711	0.892523364
akt2	1.119815668	0.812345679
sema6dl	1.119760479	0.75
TP53INP2	1.11965812	1.042986425
ugt5b2_2	1.11965812	1.807017544
si:ch211-212k18.5	1.119565217	0.272727273
fbxo25	1.119565217	1.167247387
ucp3	1.119354839	1.581717452
mkrn2_2	1.119266055	0.849056604
arrdc1a	1.119266055	1.139784946
prpf18	1.119148936	1.295336788
supt6h	1.119133574	0.862385321
limk1b	1.119047619	1.37804878
eif3m	1.118887824	1.040364583
ascl1b_2	1.118811881	0.942622951
cox6b2	1.118661607	1.07063306
PHACTR1	1.118644068	0.372093023
mb21d2	1.118644068	0.456521739
cdadc1	1.118644068	1.108695652
ndrg1b	1.118421053	0.94017094
nocta	1.11827957	2.658415842
plekhm1	1.118181818	0.752136752
cdr2l	1.11814346	0.620408163
ranbp9	1.118081181	0.875
zgc:193505	1.117886179	1.612689394
plppr3a	1.117647059	0.384615385
cflarb	1.117647059	0.423076923
CABZ01059104.1	1.117647059	0.769230769
setd2	1.117647059	0.802083333
gdnfa	1.117647059	0.80952381
galnt1	1.117647059	0.816666667
si:dkey-100n19.2	1.117647059	0.823529412
armc4	1.117647059	0.9375
pex6	1.117647059	0.964285714
si:dkey-242k1.6	1.117647059	0.967032967
fgf20b	1.117647059	1.105263158
zgc:154006	1.117647059	1.3
cdcp1a	1.117647059	1.391304348
si:ch73-138e16.6	1.117647059	1.672727273
rps28	1.117594456	0.959806797
nrf1	1.117408907	0.952380952

mrpl32	1.117338004	1.177347243
rps26	1.117328799	0.879994844
pcdh10a	1.117241379	0.554744526
slc39a6	1.117241379	0.752293578
wdr45b	1.117216117	1.091803279
prnprs3	1.117147708	0.891050584
yipf5	1.117117117	0.941860465
plxna2	1.117021277	0.461538462
bmpr1ba	1.117021277	0.96875
usp47	1.116883117	0.782258065
srp14	1.116766467	0.891687657
rfx1a	1.116666667	0.466666667
zfyve27	1.116666667	1.182692308
dnajc12	1.116666667	1.24
ddx20	1.116666667	1.416666667
sowah1	1.116666667	1.630434783
pdcd4b	1.116607774	0.647509579
si:ch211-195d17.2	1.116564417	1.14084507
tsc1b	1.116504854	0.842696629
si:dkey-114c15.7	1.116438356	0.358490566
smyd2b	1.11641791	0.959493671
si:ch211-79l20.4	1.116402116	0.652173913
OSCP1	1.11627907	1.035294118
tarsl2	1.11627907	1.042735043
anxa13l	1.11627907	1.377777778
faxcb	1.116071429	0.682352941
CABZ01086759.1	1.116071429	0.75
bmi1a	1.116071429	1.391304348
tcf25	1.115853659	1.711111111
prss16	1.115789474	1.036649215
ibtk	1.115740741	1.049079755
eif4enif1	1.115702479	0.78313253
hunk	1.115671642	0.81496063
galnt9	1.115646259	0.664
apoa1a	1.115545175	0.948346196
CABZ01063167.1	1.115384615	0.5
kcnc2	1.115384615	0.578947368
hbbe1.2	1.115384615	0.680287134
tmem57b	1.115384615	0.738888889
cdc7	1.115384615	0.75
si:ch211-154o6.3	1.115384615	0.836734694
vat1l	1.115384615	1
herc3	1.115384615	1.031746032
RNF14 (1 of many)_1	1.115384615	1.194444444
orc2	1.115384615	1.24137931
trmt5	1.115384615	1.256756757
ilkap	1.115384615	1.4
zgc:161973	1.115384615	1.538461538
zfand5a	1.11533052	1.323572474
lhx5	1.115107914	0.270833333
stk11	1.115107914	0.819548872
UBE2M	1.115107914	0.970037453
tbc1d15	1.115107914	1.220183486
actc1b	1.115071129	0.883636988
atp6v0ca	1.114987864	0.802332719
zfand2a	1.114893617	1.086757991
atp6v1d	1.114801444	1.174780527
hoxc4a	1.114754098	0.595238095
usp12b	1.114754098	1.12037037
dcps	1.114754098	1.151260504
si:ch211-173n18.4	1.114754098	1.636363636

maml3	1.114583333	0.505747126
SLC7A1 (1 of many)	1.114583333	1.047619048
zgc:154077	1.114583333	1.372093023
lrrn3	1.114583333	1.704225352
kidins220b	1.114374034	0.410909091
rpl21	1.114353673	0.783416386
rc3h1b	1.114285714	0.795918367
zdhhc3a	1.114285714	0.8
si:dkeyp-33b5.4	1.114285714	1.0625
si:ch73-144l3.2	1.114285714	1.40625
myocd	1.114285714	1.565217391
rnf180	1.114285714	1.580645161
pygma	1.11406578	1.210116732
ykt6	1.114035088	1.148387097
bod1	1.113970588	1.296460177
si:ch211-79k12.2	1.113924051	1.131147541
rnaseka	1.113772455	1.161616162
nup210	1.113636364	0.766666667
si:ch211-233a24.2	1.113636364	0.866666667
fam173a	1.113636364	1.428571429
CABZ01067153.1	1.113402062	0.7125
nup88	1.113402062	0.816091954
rchy1	1.113268608	1.305555556
EML6	1.113207547	0.333333333
znf385c	1.113207547	0.363636364
rexo1	1.113207547	0.75
cep19_2	1.113207547	0.847826087
zfyve28	1.113207547	1.090909091
mvb12a	1.113207547	1.326530612
zgc:163107	1.113095238	0.868421053
slc25a32a	1.113095238	1.73015873
slc7a14a	1.113043478	0.329268293
ptprdb	1.112903226	0.333333333
th	1.112903226	0.688888889
wdr13	1.112903226	1.025531915
si:cabz01039271.2	1.112676056	0.509090909
jakmip3	1.112676056	0.692307692
FBXO33	1.112676056	0.9375
mrps11	1.112676056	1.080321285
cib1	1.112612613	1.27734375
soga3a_2	1.112582781	0.588709677
spryd7b	1.112582781	0.951219512
ajuba	1.112582781	1.058823529
clstn2	1.1125	0.346153846
LTBP4	1.1125	0.847058824
mrps21	1.1125	1.39375
snrpd3l	1.112244898	1.092989986
bcl9l	1.112068966	0.770114943
snx30	1.112068966	0.952830189
syf2	1.112033195	1.1121673
ube2r2	1.112	1.048543689
noc3l	1.111764706	1.258823529
tvp23b	1.111731844	1.48447205
si:ch211-113d22.2	1.111702128	0.440944882
HIPK1 (1 of many)	1.11147541	0.684027778
rps15	1.111236983	0.927105426
prr12b	1.111111111	0.293650794
jakmip2	1.111111111	0.311688312
si:ch211-248g20.5	1.111111111	0.483146067
mkl2b	1.111111111	0.644444444
cdk6	1.111111111	0.666666667

CDH19	1.111111111	0.770833333
BX539325.1	1.111111111	0.774193548
ophn1	1.111111111	0.8125
zmat4b	1.111111111	0.888888889
card19	1.111111111	0.956521739
avpr2ab	1.111111111	1.071428571
arhgap27	1.111111111	1.136363636
polrmt	1.111111111	1.291666667
zgc:194981	1.111111111	1.347826087
plpp2b	1.111111111	1.455882353
nubpl	1.111111111	1.5
si:ch1073-296d18.1	1.111111111	1.52
si:ch211-87m7.2	1.111111111	1.555555556
znf1152	1.111111111	1.647058824
wnt9a	1.111111111	1.666666667
si:ch211-28p3.4	1.111111111	0.776470588
slc25a36b	1.111111111	0.97029703
NBR1 (1 of many)	1.111111111	1.063583815
si:ch73-266f23.3	1.111111111	1.789473684
smcr8b	1.111111111	1.9
rps11	1.111048239	0.932168662
a2ml	1.110867179	1.217518248
map2k4b	1.110429448	1.090225564
kat5b	1.11023622	1.388349515
dub	1.10989011	0.790697674
atg7	1.109756098	0.711111111
ntan1	1.109756098	0.835164835
zdhhc18a	1.109756098	1.012048193
sirt1	1.109704641	0.903846154
si:ch73-60h1.1	1.109589041	0.784313725
smim4	1.10952381	1.634920635
snx16	1.109489051	0.962406015
ppp1r7	1.109489051	1.325980392
lhx6	1.109375	0.893617021
FQ377903.3	1.109375	0.967741935
mto1	1.109375	1.055555556
flad1	1.109375	1.390909091
cdh2	1.109204368	0.5684803
atp1a1a.3	1.109195402	1.317829457
runx1t1	1.109090909	0.409937888
cdk11b	1.109090909	0.776515152
pard3bb	1.109090909	0.959183673
nudt18	1.109090909	1
kpna6	1.109090909	1.244897959
actr3_2	1.109040718	0.862493013
arl9	1.108910891	0.719047619
hibch	1.10877193	1.540425532
bcl9	1.108695652	0.457831325
PCDH20	1.108695652	0.697674419
PCDH20	1.108695652	0.697674419
fyco1a	1.108695652	1.014084507
trpa1a	1.108695652	1.263157895
osbpl1a	1.108695652	1.805555556
myoz1b	1.108382449	1.256985167
arpc5lb	1.108333333	1.107438017
frg1	1.108280255	1.352201258
apoa2	1.108204029	0.962228257
lsm14ab	1.10816777	0.774611399
FO818659.1	1.108108108	0.454545455
allc	1.108108108	1.384615385
trmt44	1.108108108	1.477272727

gcn1	1.108024691	0.791530945
acvr1l	1.108013937	1.098360656
rpap3	1.107913669	0.865168539
raf1b	1.107913669	0.975806452
smcr8a	1.107843137	1.506329114
kctd10	1.107784431	0.820809249
tmem14ca	1.107784431	1.266961652
tmem35	1.107758621	0.788546256
si:ch211-259g3.4	1.107692308	0.807692308
CR396586.1	1.107692308	1.307692308
pcmt	1.107623318	0.820143885
bhmt	1.107598019	0.978500962
GPT (1 of many)	1.107594937	2.383928571
rnpc3	1.107526882	1.11
rpl23a	1.107492488	0.894024535
rpl30	1.107446952	0.922059037
ep400	1.107438017	0.647058824
tcp11l2	1.107413011	1.163461538
slitrk3b	1.107142857	0.625
zgc:113176	1.107142857	0.6875
actr10	1.107142857	0.797802198
FUZ	1.107142857	0.914285714
zgc:158270	1.107142857	0.975
si:dkey-235d18.5	1.107142857	1.034482759
mthfs	1.107142857	1.08490566
zgc:112023	1.107142857	1.136363636
pms2	1.107142857	2.210526316
ctsz	1.10707457	1.309124767
znf410	1.106918239	0.875621891
gnb3a	1.106918239	0.941860465
glrx	1.106881406	1.200573066
fahd2a	1.106870229	1.127717391
PDE3B	1.106796117	0.764150943
dad1	1.106710671	1.100278552
dusp16	1.106666667	0.816091954
exosc10	1.106666667	1.150793651
si:dkey-153k10.9	1.106557377	0.470899471
cyp2aa1	1.106557377	1.102941176
CABZ01084566.2	1.106557377	1.12
foxo1a	1.106557377	1.237113402
rnf111	1.106508876	0.729166667
nrp2b	1.106463878	0.49
ube4b	1.106463878	0.647826087
gpsm1a	1.106382979	0.333333333
DUSP26	1.106382979	0.56
trappc9	1.106382979	0.811320755
kmt2ba_2	1.106382979	0.845070423
egfra	1.106382979	1.068181818
zgc:77517	1.106314085	0.994404476
caska	1.10625	0.857142857
rbms3	1.10619469	1.260869565
cox14	1.106138107	1.075949367
stk25b	1.106029106	0.65805169
crygm2d10	1.105996263	0.963882619
znf1179	1.10591133	1.216049383
tldc1	1.105882353	0.722222222
si:ch73-211l13.2	1.105882353	0.875
nhlrc2	1.105882353	1.061538462
ubap2a	1.105839416	0.559498956
cnot1	1.105820106	0.65408805
dscama	1.105769231	0.297297297

hoxd3a	1.105769231	0.361702128
coq9	1.105754277	1.125
pfklb	1.105691057	0.510822511
fgfr1op	1.105633803	1.361344538
slc1a5	1.105610561	1.461538462
fus	1.105363985	0.688284519
adra1ab	1.105263158	0.736842105
reep6	1.105263158	0.85
si:cabz01069016.2	1.105263158	0.870967742
kiaa0907	1.105263158	0.963768116
si:ch211-40k21.9	1.105263158	0.976744186
CXCL3	1.105263158	1
GPR180	1.105263158	1.181818182
si:dkey-65b13.9	1.105263158	1.263157895
CR925773.1	1.105263158	2.192307692
pdzd8	1.105263158	0.755102041
yy1b	1.105263158	0.79704797
zdhhc16b	1.105263158	1.194444444
MANEAL	1.105263158	1.772151899
znf593	1.105022831	1.26754386
gpib	1.10501355	1.092028986
syt2a	1.104895105	0.31
ppp1r8b	1.104895105	0.881118881
tbx3a	1.10483871	0.969072165
tmem5	1.104761905	1.115384615
adssl1	1.104720564	1.220922677
smdt1b	1.104718067	0.969392417
notch1b	1.104651163	0.49382716
myog	1.104651163	0.867298578
hoxd10a	1.104651163	1.319444444
usp40	1.104477612	0.981481481
ptena	1.104321908	0.735818476
ces2	1.104195122	1.129296236
c2cd2l	1.104166667	0.64556962
PARG	1.104166667	0.890625
megf10	1.104166667	0.979166667
sat2b	1.104166667	1.153846154
zgc:174928_1	1.104166667	1.476190476
eef1b2	1.104150352	1.048872972
atp5j	1.104135894	1.020903955
ubap2b	1.104052574	0.76953125
zgc:101569	1.104046243	1.252688172
gria1a	1.103896104	0.418181818
atxn7l2b	1.103896104	0.604651163
cramp1	1.103896104	0.644067797
gkup	1.103896104	1.061728395
she	1.103896104	1.31147541
jagn1a	1.103896104	1.430656934
xpr1b	1.103773585	0.804123711
cox15	1.103626943	0.847222222
serpina1	1.103557591	1.061818182
jph3	1.103448276	0.451612903
lmx1al	1.103448276	0.526315789
ZNF608	1.103448276	0.542372881
b4galnt4b	1.103448276	0.571428571
samd1b	1.103448276	0.576923077
cdh8	1.103448276	0.587412587
ubp1	1.103448276	0.620437956
nek7	1.103448276	0.868852459
antxr1a	1.103448276	0.916666667
angptl6	1.103448276	0.931034483

trim45	1.103448276	1
cyp27b1	1.103448276	1.018518519
si:dkey-145p14.5	1.103448276	1.12244898
si:rp71-17i16.6	1.103448276	1.205882353
f3b	1.103448276	1.772151899
rps12	1.103399993	0.821290931
med7	1.103286385	0.987179487
rrp1	1.103092784	0.831746032
btf3l4	1.103076923	1.321875
cry1aa	1.103067485	0.727152318
pald1a	1.103030303	0.754098361
fam188a	1.103030303	1.277372263
tmem151a	1.102941176	0.5
ostm1	1.102941176	1.5
zgc:111986	1.102827763	0.842155919
atp1a3a	1.102817974	0.226840855
ell	1.102702703	1.006410256
MARCHF9	1.102564103	0.468468468
zfpm1	1.102564103	0.595238095
emx3	1.102564103	1.28
wdr34	1.102564103	1.34375
si:dkey-77a20.5	1.102459016	0.4
ewsr1b	1.102409639	0.847317744
dock9a	1.102272727	1.205882353
tnnc1b	1.102220771	1.156827668
fam8a1b	1.102040816	1.357142857
sugp1	1.102040816	1.25
ssx2ipb	1.102040816	1.459016393
gmppaa	1.102040816	1.595375723
zgc:162948	1.102040816	1.736842105
mcl1b	1.101867572	1.427787934
sdhc	1.101843723	1.155416013
im:7140055	1.101694915	0.944444444
hspb3	1.101694915	0.967213115
mhc1zaa_1	1.101694915	1.27027027
polr3c	1.101694915	1.301886792
tp53inp1	1.101626016	1.298210736
ssbp4	1.101610905	0.771631206
rab5c	1.101596517	0.851910828
cicb	1.1015625	0.568627451
cdc42bpab	1.101449275	0.869565217
smarca1	1.101449275	0.909090909
qtrt1	1.101449275	1.036363636
smyhc1	1.101349631	1.187737385
tmem246	1.101265823	1.114285714
st7l	1.101265823	1.265060241
pcmtl	1.101265823	1.311111111
lin54	1.101123596	0.585365854
sult1st4	1.101123596	1.03960396
zfpl1	1.101123596	1.405405405
fkbp2	1.101109741	1.095959596
AKR1B1	1.100977199	1.594684385
tubgcp2	1.100917431	1.017699115
nedd8l	1.100877193	1.48
frs2b	1.100840336	0.705426357
si:ch1073-385i9.2	1.100840336	0.86
CABZ01088933.1	1.100775194	0.277372263
ankhb	1.100775194	0.958333333
ufd1l	1.100737101	1.345323741
sbf1	1.100671141	0.754716981
impact	1.100628931	1.267605634

RNF208	1.100529101	0.460122699
tcea1	1.100378788	0.935817805
prrc2a	1.100204499	0.742021277
pde4a	1.1	0.366666667
lrfn5a	1.1	0.419354839
GGA3 (1 of many)	1.1	0.5
setd1ba	1.1	0.586956522
rasa3	1.1	0.629213483
si:ch73-209l12.1	1.1	0.631578947
wdr53	1.1	0.666666667
sobpb	1.1	0.698924731
jag2b	1.1	0.714285714
pomgnt2	1.1	0.717391304
c2cd3	1.1	0.733333333
zgc:153012	1.1	0.748201439
ngfa	1.1	0.761904762
TMPRSS6	1.1	0.8
lrrfip1b	1.1	0.829787234
si:ch211-153b23.3	1.1	0.84
si:ch73-173p19.1	1.1	0.876033058
jak2a	1.1	0.909836066
plac8l1	1.1	1.3
znf1119	1.1	1.535714286
si:dkey-228a15.6	1.1	1.545454545
lrrc30a	1.1	1.545454545
si:dkeyp-2e4.3_2	1.1	1.684210526
rgs5b	1.1	1.935064935
si:ch211-158e19.2	1.1	2.076923077
STYK1 (1 of many)	1.1	2.538461538
gdpd5a	1.1	0.472222222
MFN1	1.1	0.5
pak7	1.1	0.705882353
ndufaf4	1.1	0.804597701
wdr26b	1.1	0.854625551
chico	1.1	0.859649123
ddx6	1.1	0.885714286
dennd1a	1.1	0.980392157
vps33a	1.1	1.019047619
acot7	1.1	1.163636364
IKZF4	1.1	1.176470588
arih2	1.099808061	0.887189293
farp1	1.099378882	0.720496894
fam49bb	1.099337748	1.432692308
chrnb3a	1.09929078	1.221153846
ACTC1 (1 of many)	1.099284436	0.980471928
reep5	1.099252209	1.183551847
snx27a	1.099099099	0.964566929
jph2	1.09909166	0.966865474
znf143a	1.099056604	1.103448276
pigs	1.098901099	1.059701493
cyhr1	1.098765432	0.877192982
sft2d1	1.098684211	1.207407407
med10	1.098639456	1.205298013
gnb1a	1.098621421	0.616336634
dnmt3aa	1.098591549	0.693877551
nalcn	1.098360656	0.31372549
ssr3	1.09832636	1.009220839
lancl2	1.098214286	0.677419355
si:rp71-1g18.1	1.098214286	0.97029703
si:dkey-102f14.5	1.098214286	1.216216216
ube2d3	1.098206661	1.463702359

myha	1.098181818	1.45323741
trim105	1.098039216	0.949152542
fgd5a	1.098039216	1.073170732
si:ch73-18b11.2	1.098039216	1.595238095
pnkd	1.098039216	1.609090909
cog5	1.097826087	0.864583333
actr6	1.097826087	1.49689441
si:ch211-230g15.5	1.097560976	0.555555556
si:dkey-112m2.1	1.097560976	0.7
glra2	1.097560976	0.791666667
calcr	1.097560976	0.864864865
pebp1	1.097560976	0.995525727
tln2b	1.097560976	1.05
ercc4	1.097560976	1.066666667
pdyn	1.097560976	1.144578313
pex11g	1.097560976	1.333333333
sell	1.097560976	1.343137255
pcdh1gb9	1.097560976	1.35483871
uba52	1.097520881	0.96297864
mgat2	1.097402597	0.966216216
ccdc93	1.097402597	1.464912281
fut8a	1.097345133	0.62406015
leo1	1.097315436	0.743859649
tox3	1.097222222	0.453125
cel.2	1.097222222	0.763265306
dnal4b	1.097222222	1.384615385
grm2b	1.097087379	0.510204082
snx14	1.097087379	0.883495146
necap2	1.097046414	1.035294118
lrp1aa	1.097014925	0.943925234
eef1da	1.096906013	1.09345301
yjefn3	1.096774194	1.073593074
tprn	1.096774194	0.510204082
lrrc73	1.096774194	0.578947368
chd6	1.096774194	0.738095238
zdhhc5a	1.096774194	0.778846154
gtf2h1	1.096774194	0.795454545
CABZ01111454.1	1.096774194	0.838709677
srek1	1.096774194	1.851851852
zgc:77929	1.096618357	1.058201058
KIF3C (1 of many)	1.096590909	0.563106796
mpp1	1.096491228	0.863874346
barhl1b	1.096296296	0.582733813
pvalb6	1.096280088	0.423766816
kif3a	1.096273292	1.004464286
crygm2d2	1.096222193	0.942163977
agr2	1.096214511	1.095582911
agbl5	1.096153846	0.655913978
cdk14	1.096153846	0.87755102
rad1	1.096153846	0.880952381
si:ch73-138e16.2	1.096153846	0.934782609
tob2	1.096153846	0.945054945
qtrtd1	1.096153846	1.087719298
gpr137	1.096153846	1.322580645
ptcd2	1.096153846	1.75
taf13	1.096103896	1.265033408
TMEM267	1.096045198	1.462184874
oaz2a	1.096	1.360587002
creb1a	1.095975232	1.047244094
rabac1	1.095975232	1.830434783
bada	1.095959596	1.68707483

lrrc39	1.095890411	1.540880503
camsap2a	1.095890411	0.442307692
TRHDE (1 of many)_1	1.095890411	0.533333333
SSTR2 (1 of many)_2	1.095890411	1.2
zgc:101851	1.095890411	1.435185185
ubl3b	1.095833333	0.536585366
fam193b	1.095588235	0.595041322
asah1b	1.095588235	1.356060606
tmem9b	1.095588235	1.545454545
exosc1	1.095541401	0.852140078
cutc	1.095541401	0.927152318
hnrnpa0a_2	1.095505618	1
mea1	1.095505618	1.021390374
slc4a2a	1.095238095	0.621212121
ralgapa1	1.095238095	0.731958763
fam222bb	1.095238095	0.814814815
ints2	1.095238095	0.822580645
trps1	1.095238095	0.666666667
si:ch211-124n19.2	1.095238095	0.6875
nrg2a	1.095238095	0.717948718
bmp16	1.095238095	0.957446809
camkmt	1.095238095	1.043478261
dolk	1.095238095	1.076923077
zbtb12.1	1.095238095	1.3
GOLPH3 (1 of many)	1.095011876	1.023498695
TMEM208	1.09486166	1.374429224
pdcd6ip	1.094827586	0.848484848
gopc	1.094827586	1.40625
si:dkey-237i9.1	1.094736842	1.442307692
tbc1d23	1.09469697	1.178010471
stau2	1.094678645	0.704805492
meis3	1.094674556	0.638297872
dclk2b	1.094594595	0.390243902
zswim5	1.094594595	0.469465649
klf12a	1.094594595	0.585858586
pdgfc	1.094594595	0.783783784
sema3c	1.094594595	1
med25	1.094488189	0.727272727
si:dkey-111f13.3	1.094488189	1.168539326
galcb	1.094488189	1.65060241
unc50	1.094339623	1.004366812
ugt5f1	1.094339623	1.395348837
coq6	1.094262295	1.420792079
pcmtd1	1.094202899	0.79389313
brd1b	1.094017094	0.831325301
tmub1	1.093984962	0.831395349
ptpra	1.093877551	0.869565217
ptgdsb.2	1.09383378	2.095238095
pwwp2a	1.09375	0.610169492
slc1a7b	1.09375	0.666666667
ext1a	1.09375	0.890909091
si:dkey-262g12.10	1.09375	1.025
RALGDS	1.09375	1.921875
dirc2	1.093567251	0.868131868
icmt	1.09352518	1.214953271
enc1	1.093457944	0.717171717
keap1b	1.093457944	1
zgc:101000	1.093442623	1.33240612
nme3	1.093385214	0.914529915
snx29	1.093333333	0.84375
rint1	1.093333333	1.017857143

rhcga	1.093189964	1.382887701
PPP2R5C	1.093167702	0.702702703
rpl11	1.093143661	0.87199943
scamp1	1.093023256	0.545454545
tmem39b	1.093023256	1.051282051
tmtc3	1.092857143	0.875
ints4	1.092783505	1.403225806
si:ch211-237b12.4	1.092631579	1.251851852
syt12	1.092592593	0.531914894
SSBP4 (1 of many)	1.092592593	0.608040201
wnk1a	1.092592593	0.783333333
fkrp	1.092592593	1.23255814
zgc:66443	1.092592593	1.688888889
slc38a4	1.092537313	0.893712575
dnmt3ab	1.092485549	0.633540373
CU639469.1	1.092465753	0.631355932
ptpn4b	1.092436975	0.376237624
scn8ab	1.092307692	0.378378378
zgc:136892	1.092307692	1.214285714
TNRC6B (1 of many)	1.092105263	0.826086957
zgc:66447	1.092105263	0.97260274
gid8b	1.092105263	1.279569892
rpl26	1.091959753	0.915938249
ncaph	1.091954023	0.76744186
fbxo18	1.091954023	1.274193548
fem1c	1.091891892	0.578947368
dennd6b	1.091836735	1.376623377
maza	1.091743119	0.517647059
pja2	1.091666667	1.422680412
sp3b	1.091603053	0.577777778
sin3ab	1.091603053	0.785714286
upf1	1.091575092	0.780172414
FBXO46	1.091549296	0.557142857
FOXK2 (1 of many)	1.091503268	0.503448276
si:ch211-208h7.4	1.091503268	0.673611111
si:ch73-366l1.5	1.091476091	0.84270301
map3k4	1.091428571	0.76300578
slc6a11b	1.091295117	0.311111111
zgc:66475	1.09122807	1.99078341
BX322657.1	1.090909091	0.954545455
fam20cb	1.090909091	1.038461538
eapp	1.090909091	1.060606061
tgs1	1.090909091	1.222222222
CR847895.3	1.090909091	1.375
wbp1la	1.090909091	1.431818182
TENM2 (1 of many)_2	1.090909091	0.272727273
dclk1a	1.090909091	0.371134021
wdr11	1.090909091	0.516129032
si:ch211-212k18.7	1.090909091	0.646203554
nkx6.1	1.090909091	0.666666667
smg8	1.090909091	0.843137255
atad2b	1.090909091	0.863636364
tiprl	1.090909091	0.975806452
kcnk9	1.090909091	1.1
viml	1.090909091	1.210526316
acer1	1.090909091	1.214285714
lyrm5b	1.090909091	1.22
polr3k	1.090909091	1.220125786
sgsm3	1.090909091	1.221428571
si:dkey-287g12.6	1.090909091	1.243478261
si:ch73-221f6.1	1.090909091	1.315789474

st3gal5	1.090909091	2.02739726
ubxn2a	1.090909091	2.681818182
lyz	1.090729783	0.658008658
smim13	1.090676884	0.960305344
psma1	1.090668432	1.013472137
si:dkey-29l4.4	1.090666667	1.039426523
dpysl3	1.090651558	0.421052632
klhl31	1.090604027	0.92706334
ctnnb2	1.090604027	0.977639752
erlec1	1.090534979	1.032608696
gpatch11	1.090452261	1.506666667
WDR26 (1 of many)	1.090425532	0.701149425
pafah1b1a	1.090405904	0.705645161
slc25a39	1.090047393	0.772357724
nme2b.2	1.090029499	0.983497733
mrpl41	1.090024331	0.854867257
cbfa2t2	1.09	0.767123288
scg2b	1.089987326	0.562318841
capn7	1.08988764	0.884615385
fhl5	1.08988764	1.646153846
ntrk3b	1.08974359	0.489361702
cuedc2	1.08974359	1.410958904
rbp2b	1.089655172	1.003631961
dopey1	1.089552239	0.642857143
si:rp71-68n21.11	1.089552239	1.474576271
tubb5	1.089529	0.528240553
rsl1d1	1.089445438	1.08774584
rps27.1	1.089418977	0.880388094
aagab	1.089385475	1.321637427
kif1bp	1.089347079	0.9375
si:ch211-102c2.7	1.089285714	1
rtn2a	1.089258699	1.476648352
tet3	1.089219331	0.467391304
derl1	1.089156627	1.423592493
drg1	1.08913649	0.85
atp2b3b	1.089108911	0.293650794
mark3a	1.089108911	0.83125
rpl24	1.089069795	0.945870275
mapk6	1.089068826	0.781563126
nadl1.1	1.089041096	0.25
mzt2b	1.089041096	1.253623188
rad21b	1.089005236	0.573099415
skia	1.088983051	0.695431472
ndufb8	1.088946459	0.901184308
gbp	1.088937093	0.819472617
EML5	1.088888889	0.655172414
hmbox1a	1.088888889	0.935483871
si:cabz01054396.2	1.088888889	1.065217391
ddx31	1.088888889	1.2
si:dkeyp-53d3.5	1.088888889	1.315789474
mars	1.088888889	0.88372093
rnf7	1.088871097	0.974744027
dbpa	1.088832487	1.134228188
cad	1.088709677	0.868085106
lztfl1	1.088709677	1.333333333
rad54l2	1.088607595	0.539473684
sox3	1.088607595	0.996478873
mturn	1.088495575	1.513513514
smarca4a	1.088343558	0.569705094
dhdhl	1.088339223	0.895705521
uqcrh	1.08826991	1.110826628

CLEC3A	1.088235294	0.75
megf8_1	1.088235294	0.791666667
clcn7	1.088235294	0.956521739
intu	1.088235294	1.172413793
SAMD8	1.088235294	1.263157895
fam46bb	1.088235294	1.37254902
cyp46a1.4	1.088235294	1.413793103
olfm2a	1.088122605	0.407079646
ccdc94	1.088050314	1.269230769
ndufa5	1.087982833	0.878997868
gmpr2	1.087947883	0.971530249
ppp3ca	1.087939698	0.618122977
si:ch211-189k9.2	1.087912088	1.223880597
syn1	1.087866109	0.246666667
arfgef2	1.087837838	0.745762712
mau2	1.087719298	0.72972973
mylpfa	1.087641319	0.957398286
mgea5	1.087636933	1.274725275
ing4	1.087599545	1.419607843
psmb2	1.0875	1.041343669
rtcb	1.0875	1.080701754
bola1	1.0875	1.201550388
RPS11 (1 of many)	1.087452471	1.13015873
inppl1a	1.087431694	0.870786517
atg13	1.087420043	1.106818182
bdh1	1.087378641	0.985148515
znf362b	1.087301587	1.538461538
rab3ip	1.087209302	0.770833333
acbd6	1.087209302	0.994186047
apip	1.087025316	1.036390102
psap	1.086969697	0.881582776
slc35f1	1.086956522	0.542857143
CABZ01021450.1	1.086956522	0.785714286
trmt10b	1.086956522	0.962962963
arhgef40	1.086956522	1
vtg7	1.086956522	1
KCNG2	1.086956522	1.05
psme4a	1.086956522	1.058823529
si:dkey-103m22.1	1.086956522	1.285714286
si:dkey-30c15.13	1.086956522	1.310810811
zufsp	1.086956522	1.382352941
zgc:114104	1.086956522	1.444444444
cxcl18b	1.086956522	1.555555556
hsbp1l1	1.086956522	1.56
irf7	1.086956522	3
pou2f2a_2	1.086956522	0.285714286
igsf3	1.086956522	0.337662338
dennd4b	1.086956522	0.603773585
zgc:175214	1.086956522	1.021978022
ninl_2	1.086956522	1.288461538
dhrs13a.3	1.086956522	1.338797814
rnf121	1.086792453	0.855895197
pdhb	1.086781399	0.937960042
rgs17	1.086734694	1.25
sarm1	1.086538462	0.536231884
selo	1.086538462	1.027272727
bcl2l1	1.086538462	0.950381679
bag6	1.08650519	0.794354839
rpl13a	1.086426831	0.985353015
clcn3	1.086419753	0.703703704
herc2	1.086419753	0.871794872

si:ch211-113p18.3	1.086419753	1.580645161
ip6k1	1.086330935	0.684684685
sdr39u1	1.086330935	0.910958904
cldn1	1.086330935	1.245939675
psma2	1.086247086	1.21661055
xkrx	1.086206897	0.710743802
pgk1	1.086127168	0.883969907
rasgef1ba	1.086053412	1.269230769
tnnt3a	1.086042532	1.006810443
neil1	1.086021505	1.608247423
tusc2b	1.085918854	0.925143954
hcfc1b	1.085714286	0.577181208
brd2b	1.085714286	0.586206897
yeats2_1	1.085714286	0.653846154
marveld2a	1.085714286	0.820512821
klhl7	1.085714286	0.88
kcnab2b	1.085714286	1
hspbp1	1.085714286	1.269230769
pfkfb1	1.085714286	1.339285714
COQ8A (1 of many)	1.085714286	1.904761905
syt1a	1.085612366	0.452076677
rnf41	1.085561497	0.88125
cetn2	1.085526316	0.990990991
pfdn6	1.085501859	1.104501608
cox7b	1.085452696	1.012774655
maea	1.085294118	1.401869159
acsl5	1.085185185	1.077625571
plpp6	1.085106383	1.058823529
hook2_1	1.085106383	1.150537634
FO904917.1	1.085106383	1.184210526
eogt	1.085106383	1.210526316
crygm2d8	1.085023123	0.949092995
ccnc	1.085	1.393617021
habp2	1.08490566	0.970238095
araf	1.08490566	1.02020202
klf8	1.084848485	0.723076923
foxi3a	1.084848485	1.236686391
prr13	1.08478803	0.978947368
morn4	1.084745763	0.674418605
ino80db	1.084745763	0.708333333
snrka	1.084656085	0.675862069
desma	1.084563758	1.016839842
camta1b	1.084507042	0.311827957
elavl4	1.084455324	0.27729636
selj	1.084432718	0.946938776
cox7a2a	1.084432718	1.079534433
hsd17b12a	1.08436214	1.626062323
lman2lb	1.084337349	1.439153439
mtus2a	1.084337349	0.319148936
TTC9	1.084337349	0.462686567
tha1	1.084337349	0.833333333
ripk4	1.084337349	1
mpc2	1.084317938	1.191802417
ankrd52a	1.084210526	0.885714286
acta2	1.084128361	0.935040831
ank1b	1.08411215	0.294117647
prrt2	1.08411215	0.362637363
kat6b	1.08411215	0.576086957
NDST1 (1 of many)	1.08411215	0.907894737
gemin6	1.08411215	0.927536232
ptar1	1.083969466	1.298969072

psen1	1.083870968	0.868243243
ptch1_2	1.083798883	0.768707483
mrps16	1.083743842	1.505050505
supt5h	1.083710407	0.76584022
pfdn2	1.083643123	1.108108108
ogt.1	1.083516484	1.009677419
si:ch211-168f7.5	1.083333333	0.630952381
cacng5a	1.083333333	0.692307692
dennd5b	1.083333333	0.703196347
gas6	1.083333333	0.751824818
si:ch211-188p14.2	1.083333333	0.785714286
zbtb8a	1.083333333	0.791666667
frmd3	1.083333333	0.8125
FO704641.1	1.083333333	0.875
wu:fc75a09	1.083333333	0.9
prmt2	1.083333333	1
socs6b	1.083333333	1.024390244
leng9	1.083333333	1.090909091
map3k2	1.083333333	1.142857143
atoh7	1.083333333	1.485714286
polk	1.083333333	1.5625
trim35-39	1.083333333	2.7
atp6v0a2a	1.083333333	0.56
pald1b	1.083333333	0.609375
dennd5a	1.083333333	0.662162162
si:ch211-195b21.5	1.083333333	0.669856459
ube3d	1.083333333	0.755102041
c19h1orf109	1.083333333	0.803921569
brwd3	1.083333333	0.869565217
si:ch211-266d19.4	1.083333333	0.872340426
fra10ac1	1.083333333	1.157142857
jmjd7	1.083333333	1.333333333
zgc:153292	1.083333333	1.774193548
si:ch1073-100e22.1	1.083076923	0.473520249
os9	1.083067093	1.171206226
arid1aa	1.082926829	0.502824859
chd8	1.082840237	0.617647059
mrpl34	1.082802548	1.204968944
tyrp1a	1.082802548	1.26010101
sncaip	1.082644628	0.804123711
ckap5	1.082621083	0.606164384
eif2b2	1.082621083	1.036945813
unc119.1	1.082568807	1.461538462
crygm2d3	1.082567245	0.973385085
phc2b	1.082474227	0.56626506
arf3b	1.082437276	0.58988764
pex5	1.082352941	0.795918367
ssna1	1.082191781	0.732394366
snrpd1	1.082142857	0.87863464
taf9	1.082111437	1.007042254
rfc4	1.082089552	0.771812081
eps8	1.081967213	0.578125
klf13	1.081967213	0.705357143
hivep3b	1.081967213	0.80952381
krt1-c5	1.081967213	0.986842105
rpl37_2	1.081952587	0.87656498
rpl7	1.081841544	0.999235155
ndufa2	1.081818182	0.956326988
ubac2	1.081818182	2.078947368
si:ch211-193l2.5	1.081632653	1.25
itcha	1.081632653	1.5

tdh	1.081556684	1.505042959
cryba2b	1.081471451	1.030506406
ckma	1.081203008	0.864155588
gsk3ab	1.081151832	0.652818991
sulf2b	1.081081081	0.554347826
wu:fj29h11	1.081081081	0.818181818
det1	1.081081081	0.911764706
galnt13	1.081081081	0.93220339
nox1	1.081081081	1.222222222
si:ch211-248e11.2	1.081081081	2.296296296
rpl14	1.081041545	0.889387464
pvalb4	1.081028982	0.999484695
nampta	1.081005587	0.921768707
oxr1a	1.08097166	0.619266055
ubap2l	1.080924855	0.689655172
snrpd2	1.080872914	0.961538462
mfn1b_1	1.080851064	0.894009217
cops7a	1.080851064	1.074309979
vps33b	1.080808081	1.261363636
sf3b6	1.080786026	1.286353468
rpl5a	1.080708905	1.043572327
gpr137bb	1.080645161	0.386363636
ncoa1	1.080645161	0.730769231
si:ch73-380l3.2	1.080645161	1.14516129
polr2h	1.08056872	1.216666667
gatsl2	1.080508475	0.962343096
znf219	1.08045977	0.285714286
pax10	1.08045977	1.175438596
slc20a1b	1.080368906	0.912721893
arhgef18a	1.080357143	1.4
pqbp1	1.080291971	1.080882353
hmgn7	1.080243498	1.010804663
irx1a	1.080213904	0.443181818
mrps10	1.080152672	0.819875776
RIN2 (1 of many)	1.08	0.615384615
slc8a1b	1.08	0.623655914
ndst2a_1	1.08	0.625
smarcal1	1.08	0.783783784
cnot3a	1.08	0.922680412
si:ch211-261d7.6	1.08	1.314285714
pargl	1.08	1.347826087
zgc:63972	1.08	1.510638298
lhfpl4a	1.08	0.895833333
skiv2l	1.08	0.946428571
MRPS12	1.079831933	1.315789474
nkain4	1.079787234	0.780373832
psmd14	1.079761905	1.079903148
tmem198a	1.079710145	0.597826087
fbxl14a	1.079710145	1.053333333
asb2b	1.079545455	0.63559322
blmh	1.079487179	0.867875648
gapvd1	1.079470199	0.737288136
kdm3b	1.079422383	0.756302521
si:ch73-46j18.5	1.079399142	0.762096774
brinp2	1.079365079	0.463414634
ep300b	1.079365079	0.737373737
mfsd8	1.079310345	0.727891156
psmg2	1.079245283	1.088135593
si:cabz01062511.1	1.079207921	0.573170732
si:ch211-180f4.1	1.079207921	0.584615385
tbxas1	1.079207921	0.98989899

rnf115	1.079069767	0.891213389
six3b	1.079037801	0.926739927
lars2	1.078947368	0.756097561
zgc:112083	1.078947368	0.862745098
timeless	1.078947368	0.917197452
FO704821.1	1.078947368	0.933333333
prkdc	1.078947368	0.96
ndufa9a	1.078947368	1.100973236
stard3nl	1.078947368	1.273584906
rbm34	1.078947368	1.76
me2	1.078787879	0.962441315
slc45a4	1.078740157	0.944444444
lamp1	1.078740157	1.028985507
lamp1	1.078740157	1.028985507
ispd	1.078740157	1.051282051
klf11a	1.078651685	0.762452107
xpo4	1.078651685	0.793814433
nfyba	1.078651685	1.093812375
echs1	1.078528827	1.459259259
kcnj10a	1.078431373	0.266666667
tanc1a	1.078431373	0.641975309
abcc10	1.078431373	0.87804878
fbxo42	1.078431373	0.904761905
cnstb	1.078431373	1.025641026
znf628	1.078431373	1.034883721
net1	1.078397213	1.100732601
slc25a33	1.078380706	1.5456942
atp6ap2	1.078327444	1.195223881
sox4a_2	1.078319007	0.612149533
mfap4	1.078125	0.915662651
cdc42bpb	1.078066914	0.769874477
laptm4a	1.077981651	1.251261352
gprc5ba	1.077922078	0.591623037
pcsk6	1.077777778	0.764044944
ankrd13a	1.077777778	1.227272727
ppp2r2ca	1.077586207	0.309859155
gtf3aa	1.077464789	1.225806452
zic1	1.077405858	0.579175705
akt1s1	1.077367206	0.987485779
naca	1.07732572	1.059470377
fkbp8	1.077319588	1.069892473
crygm2d7	1.077264422	0.954986554
lmnb1	1.077253219	0.779582367
sod1	1.077253219	1.163849155
rundc3ab	1.077220077	0.814655172
crygm2d11	1.077185613	0.946433105
psmd6	1.077181208	1.018378378
MPP4 (1 of many)_1	1.076923077	0.710526316
miip	1.076923077	0.88372093
si:cabz01080528.1	1.076923077	0.944444444
zfp64	1.076923077	0.952380952
chmp4c	1.076923077	1.4125
aars2	1.076923077	1.541666667
SRCIN1	1.076923077	0.368421053
ahdc1	1.076923077	0.41509434
si:ch211-199o1.2	1.076923077	0.421052632
tcf20	1.076923077	0.616071429
TRABD2A	1.076923077	0.722222222
SBSPON	1.076923077	0.75
zdhhc7	1.076923077	0.753246753
itln2_2	1.076923077	0.761904762

large	1.076923077	0.826086957
si:dkey-177p2.6	1.076923077	0.920502092
kcnv2b	1.076923077	0.928571429
si:dkey-240n22.6_1	1.076923077	1
ppp5c	1.076923077	1.02
ripk3	1.076923077	1.095238095
lcmt2	1.076923077	1.272727273
CABZ01021450.2	1.076923077	1.363636364
si:ch211-1o7.3	1.076923077	1.363636364
porcn	1.076923077	1.365853659
CU856539.4	1.076923077	1.416666667
dpcd	1.076923077	1.756756757
zgc:171686	1.076923077	1.769230769
gadd45aa	1.076923077	2
uqcrb	1.076861489	1.065887699
yipf2	1.076687117	1.07079646
mtfr1l	1.076628352	0.90212766
armc1	1.076576577	1.144654088
fkbp4	1.076502732	1.032608696
tmem127	1.076502732	1.144927536
rsl24d1	1.07643743	1.176578411
xrcc5	1.076433121	0.98467433
rp9	1.076433121	1.255319149
mef2d	1.076271186	0.795918367
si:zfos-80g12.1	1.076271186	1.557894737
cox7c	1.076261373	1.005640612
clstn3	1.076219512	0.329842932
st8sia1	1.076190476	1.282051282
si:ch73-373m9.1	1.076086957	0.714285714
atf7b	1.076086957	0.803797468
ifngr1	1.076086957	1.277777778
zgc:86609	1.075829384	1.162393162
zzef1	1.075757576	0.859649123
ip6k2b	1.075757576	1.085981308
relb	1.075757576	1.547169811
si:ch1073-190k2.1	1.075539568	1.773305085
ap2b1	1.075517661	0.785947712
NEK9	1.075471698	0.962962963
CTTNBP2NL (1 of many)	1.075471698	1.428571429
tfap2e	1.075342466	0.556650246
asphd2	1.075342466	0.946428571
sox19b	1.075268817	0.396396396
nrcama	1.075268817	0.540983607
ptk7a	1.075268817	0.724324324
oca2	1.075268817	0.946428571
extl3	1.075268817	1.06
gltscr1l	1.075268817	1.14516129
gabbr1b	1.07518797	0.8
isl2a	1.075144509	0.561728395
rps8a	1.075140256	0.910553682
dynll2a	1.075136612	1.299657534
shfm1	1.075117371	1.217532468
ndufb10	1.075028857	1.057648953
dachc	1.075	0.506329114
heatr5b	1.075	0.563380282
park2	1.075	0.659090909
fam19a4b	1.075	0.926829268
si:ch211-204a13.2	1.075	1.024390244
fbxo30b	1.075	1.138888889
pik3c3	1.075	1.144067797
si:ch211-196h16.5	1.075	1.242424242

vat1	1.074921956	0.527989822
tfam	1.074918567	1.43129771
zgc:103697	1.07480315	1.434579439
srpk1b	1.074766355	0.757425743
vps29	1.074666667	1.168660287
tenm4	1.074626866	0.352941176
ube3b	1.074626866	0.739130435
APH1A	1.074626866	0.877192982
mettl6	1.074626866	1.563636364
fyna	1.074468085	1.035714286
atp1a3b	1.074450085	0.372685185
pam	1.074418605	0.688172043
il1rapl1a	1.074380165	0.6125
nt5dc2	1.074349442	0.827140549
bptf	1.074324324	0.758928571
atg4b	1.074324324	1.34
stox2a	1.074285714	0.398496241
tbp	1.074285714	0.932098765
grin1b	1.07424594	0.439490446
akt3b	1.074074074	0.5
ebf1a	1.074074074	0.623376623
tjap1	1.074074074	0.710843373
tctn2	1.074074074	0.787878788
si:ch211-108m2.2	1.074074074	0.850746269
si:ch211-255i3.4	1.074074074	0.882352941
tra2b	1.074074074	0.98467433
hltf	1.074074074	1.159090909
trmt10a	1.074074074	1.533333333
si:dkey-6e2.2	1.074074074	1.64
ccdc28a	1.073891626	1.370165746
csnk2a2b	1.073770492	0.650485437
sepw1	1.073763379	1.205644108
nap1l4a	1.073761855	1.412290503
polr2a	1.073746313	0.78490566
sbno1	1.073732719	1.041420118
arl4cb	1.073684211	1.323333333
zgc:110319	1.073684211	2.178571429
ankfy1	1.073619632	0.843283582
clybl	1.073619632	1.091463415
mrps9	1.073578595	1.125
b3gnt2b	1.073529412	1.166666667
tyw3	1.073529412	1.466666667
ftr67	1.073446328	1.464516129
snx27b	1.073394495	0.904255319
prpf31	1.073359073	0.939393939
med19a	1.073359073	1.114982578
ppiaa	1.073339085	0.911428955
tmem176l.1	1.073333333	1.68707483
sez6l	1.073170732	0.448275862
ankib1a	1.073170732	0.617647059
RUNDC1	1.073170732	0.828571429
rbm48	1.073170732	0.98
gba	1.073170732	1.025
lrrcc1	1.073170732	1.785714286
mt-co1	1.073167919	0.996144327
irx5a	1.073076923	0.556962025
mcts1	1.073059361	1.258517034
mrps14	1.073022312	1.415048544
dnajc10	1.072992701	1.218181818
eif4g3b	1.072916667	0.776315789
nup37	1.072916667	0.971428571

b3gat3	1.072916667	1.276923077
mrpl38	1.072796935	1.214750542
rpl23	1.07277628	0.894714552
foxk2	1.072727273	0.586206897
RABGAP1	1.072727273	0.738372093
zgc:165409	1.072727273	0.866666667
dlx6a	1.072727273	1
PRKAB2	1.072727273	1.005181347
actr1	1.072705602	0.696850394
emx2	1.072625698	0.825581395
bloc1s4	1.072580645	1.739583333
mex3b	1.072519084	0.498007968
rplp0	1.072512369	0.811701985
si:dkey-19b23.12	1.072483221	1.680224404
foxg1a	1.072463768	0.586206897
asxl1	1.07239819	0.622093023
eef1db	1.072334079	1.026563839
psmd4a_2	1.072131148	1.171993912
cox8a	1.072025897	0.895152324
sft2d3	1.072	1.090163934
ndrg3b	1.071875	0.700348432
ddx46	1.071875	0.767361111
dmap1	1.071856287	0.832335329
sp8a	1.071856287	1.650485437
vldlr	1.071748879	0.386597938
actc1a	1.071604331	0.79855905
nt5c3a	1.07150368	1.238430584
wdr17	1.071428571	0.433333333
adgrd2	1.071428571	0.48
gpc5b	1.071428571	0.538461538
FRMD4A	1.071428571	0.548780488
PIGG	1.071428571	0.555555556
ANKRD34A	1.071428571	0.621621622
kalrna	1.071428571	0.65
hivep1	1.071428571	0.6875
ANKRD28 (1 of many)	1.071428571	0.703703704
mpx	1.071428571	0.732673267
slc22a23	1.071428571	0.768421053
si:dkey-148h10.5	1.071428571	0.789473684
srxn1	1.071428571	0.8
cmtr1	1.071428571	0.804511278
znf438	1.071428571	0.810810811
si:dkeyp-113d7.10	1.071428571	0.866666667
AL935300.1	1.071428571	0.933333333
mbip	1.071428571	1
bmpr1bb_1	1.071428571	1.050847458
fdx1l	1.071428571	1.073170732
adamts2	1.071428571	1.090909091
tmem248	1.071428571	1.181818182
wdr54	1.071428571	1.182692308
wu:fc30c06	1.071428571	1.2
pcyt1ab	1.071428571	1.227848101
phospho1	1.071428571	1.268518519
ganc	1.071428571	1.291666667
neu3.1	1.071428571	1.315789474
stx17	1.071428571	1.333333333
slc6a18	1.071428571	1.333333333
znf1159	1.071428571	1.36
si:dkey-261l7.2	1.071428571	1.5
sytl1	1.071428571	1.8
trim35-1	1.071428571	1.9

si:ch211-40k21.5	1.071428571	2.5
mt-nd4	1.071411163	1.426091407
ndufs8a	1.07128868	1.090616046
smarcc1a	1.071264368	0.577777778
srp72	1.071212121	0.733236152
si:dkey-18a10.3	1.071174377	1.386046512
npc1	1.071161049	0.964749536
crygm2d5	1.071111111	0.944982051
polr3glb	1.071038251	1.080246914
si:ch211-153b23.5	1.070895522	1.505952381
zwi	1.070866142	0.734513274
si:ch211-269k10.2	1.070866142	0.796875
EMP3 (1 of many)	1.070866142	1.784313725
UNC13A	1.07079646	0.532258065
kdm2bb_1	1.070707071	0.960526316
dnajc4	1.070707071	1.5
kmt2d	1.070588235	0.767857143
celf4	1.070539419	0.326797386
ss18l2	1.070512821	1.128712871
slu7	1.070351759	0.911694511
bzw1b	1.070205479	1.639175258
bnip3	1.070175439	0.763513514
maml1	1.070175439	0.788461538
pbrm1l	1.070175439	0.872881356
dmrta2	1.070175439	0.977272727
porcnl	1.070175439	1.294117647
bnip3la	1.070175439	1.424242424
mterf2	1.070175439	1.590909091
tefm	1.070175439	1.591836735
hsd17b4	1.070028011	1.03358209
rbm27	1.07	0.717948718
kat6a	1.069892473	0.629370629
rnf220a	1.069868996	0.93373494
grm4	1.069767442	0.4
rhbdf1b	1.069767442	0.666666667
lmf2a	1.069767442	1.153846154
il12rb2l	1.069767442	1.219512195
gnb5a	1.069651741	0.625766871
cand2	1.069620253	1.061728395
pccb	1.069518717	1.05982906
myl1	1.06949862	0.855817941
serf2	1.069398546	1.358835477
cyp2y3	1.069343066	1.305869074
h2afva	1.069339112	1.127388535
si:dkey-184p18.2	1.069306931	1.787878788
sp4	1.069230769	0.556451613
taf2	1.069230769	0.716666667
slc16a6b	1.069230769	0.941747573
si:dkey-24l11.2_2	1.069230769	1.157303371
kansl3	1.069124424	0.790243902
mt-nd1	1.069111662	1.076072851
prkar1aa	1.069007264	0.957583548
hint1	1.06899724	1.084718923
grinaa	1.068965517	0.581967213
soga3b	1.068965517	0.583333333
si:dkey-20i20.11	1.068965517	0.866666667
tet1	1.068965517	1.047619048
BAHD1 (1 of many)	1.068965517	1.096774194
si:ch73-304f21.1	1.068965517	1.157894737
pgm3	1.068965517	1.438356164
zgc:171727_2	1.068965517	1.807692308

si:ch211-10a23.2	1.068965517	1.053571429
zgc:92380	1.068846816	1.093442623
GJD2 (1 of many)	1.068783069	0.391608392
slc4a10a	1.068783069	0.405594406
dync1i1	1.068783069	0.9125
slc32a1	1.06875	0.463709677
flcn	1.06870229	1.153846154
ube2ib	1.068656716	0.985790409
mylpfb	1.06865003	1.124512783
trak2	1.068493151	0.444444444
lgi2b	1.068493151	0.791208791
si:ch211-114n24.6	1.068384539	0.632393085
ndufs3	1.068376068	1.12599906
pgam1b	1.068345324	0.906086957
5_8S_rRNA_8	1.068273092	0.44534413
fmr1	1.068273092	0.844444444
myhz1.1_1	1.068206406	1.230090579
asic4a	1.068181818	0.333333333
bahcc1	1.068181818	0.565217391
phf14	1.068181818	0.567567568
CABZ01085657.1	1.068181818	0.947368421
ryr1b	1.068181818	1.039370079
si:ch211-128m15.3	1.068181818	1.060606061
rad23ab	1.068181818	1.188405797
rasef	1.068181818	1.925925926
BX927081.1	1.068181818	2.807692308
fth1a	1.068119672	0.950806963
wwp1	1.068027211	0.923611111
rbbp5	1.068	0.608856089
akr1b1	1.068	1.522304833
tbc1d12b	1.067961165	1.015384615
gnpda2	1.067961165	1.276595745
bcr	1.067901235	0.552631579
ap5s1	1.067901235	1.042168675
rer1	1.067885117	1.495207668
stmn2b	1.067839196	0.332155477
thoc1	1.06779661	0.948387097
l3mbtl2	1.06779661	0.886363636
s100a10b	1.067743206	1.217376374
otud5b	1.067567568	0.766055046
rab7	1.067567568	0.876854599
arhgap17a	1.067567568	1.164179104
nbn	1.067567568	1.416666667
aimp1	1.067551267	0.750483559
tlk1b	1.067510549	0.838862559
si:ch211-276i12.12	1.067419635	1.198392555
tubb4b	1.067371868	0.759185584
msra	1.067307692	0.982142857
atn1	1.067226891	0.409090909
irf1b	1.067226891	0.9296875
irf2bp2b	1.067213115	0.841827768
rps9	1.067179725	0.960618567
nfu1	1.067164179	1.111821086
cadm3	1.067010309	0.349315068
ptprua	1.066666667	0.322033898
caskin1	1.066666667	0.604651163
ube2e2	1.066666667	0.705555556
adprhl2	1.066666667	0.729166667
n6amt1	1.066666667	0.794444444
bcl6ab	1.066666667	0.8
penka	1.066666667	0.801724138

six2b	1.066666667	0.828571429
stc1	1.066666667	0.875
prkd2	1.066666667	0.904109589
paqr3b	1.066666667	0.906976744
nfkbil1	1.066666667	0.916666667
ccar2	1.066666667	0.925233645
cdk17	1.066666667	0.943548387
zgc:113227	1.066666667	0.96969697
ifih1	1.066666667	1.076923077
efnb2b	1.066666667	1.085714286
si:ch211-222e23.2	1.066666667	1.105263158
klf9	1.066666667	1.242630385
pigv	1.066666667	1.25
lsm10	1.066666667	1.56
fbxo44	1.066666667	1.789808917
anxa5a	1.066666667	1.805084746
etfdh	1.066528067	0.897435897
sumo1	1.066521265	1.328519856
si:ch211-5k11.2_2	1.066503218	0.647094249
adipor1a	1.066473988	0.794871795
atad1b	1.06640625	2.094936709
slc44a2	1.066390041	0.661202186
lim2.1	1.066390041	1.339407745
apba2b	1.066371681	0.296052632
rps3a	1.066363201	0.980365207
mrps26	1.06626506	1.087804878
atp5j2	1.066233239	1.036132813
icn	1.066215678	0.783326661
sepw2b	1.066202091	1.936790924
smfn	1.066176471	1.212765957
fbxo9	1.066137566	1.114441417
lrfn5b	1.066037736	0.46728972
dag1	1.066	0.826603325
NWD2	1.065934066	0.661764706
sema3fa	1.065934066	0.724637681
larp6	1.065934066	0.741935484
nkiras1	1.065934066	1.384615385
cdc5l	1.065868263	1.011286682
taf10	1.065843621	1.109615385
si:dkey-126h10.2	1.065789474	0.666666667
zgc:162200	1.065789474	0.862068966
tma7	1.065774805	0.974332649
ppp2r5d	1.065693431	0.650485437
strip2	1.06557377	0.829787234
tecpr2	1.06557377	1
nfkb1	1.06557377	1.023255814
znf1124_1	1.06557377	1.565217391
crkl	1.06547619	0.736526946
pip5k1ab	1.065420561	1.035714286
anapc16	1.06539075	0.942857143
CPEB2	1.065217391	0.590909091
si:dkey-91i10.2	1.065217391	0.676470588
clcn1a	1.065217391	0.954545455
gemin4	1.065217391	1.127659574
rtfdc1	1.065217391	1.15942029
zgc:86896	1.06514658	1.013931215
brd7	1.065060241	1.058981233
rabgef1	1.065	0.993006993
tshz3b	1.064935065	0.669565217
cars2	1.064935065	1.803921569
crygm2d1	1.064924255	0.9517229

evlb	1.064896755	0.543973941
tomm6	1.064893617	0.984552008
id2b	1.064814815	1.579545455
coro6	1.064814815	1.742857143
cacng2a	1.064748201	0.448598131
rps4x	1.064730364	0.889007383
pygo2	1.064705882	0.611111111
fam20b	1.064676617	0.751412429
huwe1	1.064638783	0.825870647
si:zfos-128g4.1	1.064516129	0.678571429
rab39bb	1.064516129	0.707692308
adra2a	1.064516129	0.727272727
slco5a1	1.064516129	0.8
srgap1a	1.064516129	0.871794872
akap11	1.064516129	0.903225806
si:ch73-89b15.3	1.064516129	0.914285714
ydjc	1.064516129	0.929133858
TMCC1 (1 of many)	1.064516129	1.166666667
apex2	1.064516129	1.170212766
znf1155	1.064516129	1.238095238
tmem223	1.064516129	1.268518519
mif4gdb	1.064516129	1.513513514
ms4a17a.5	1.064516129	1.555555556
zgc:175107	1.064516129	1.565217391
si:dkey-7i4.7	1.064516129	1.818181818
s100a11	1.064395513	1.353565365
ces3	1.064359862	1.286304604
tenm3	1.064220183	0.648809524
ttc4	1.064220183	1.364485981
si:ch211-147k10.6	1.064220183	2.0625
smarcd3a	1.064102564	0.752
lrrk2	1.064102564	0.962264151
ercc8	1.064102564	1.305555556
dnlz	1.064102564	1.407407407
calub	1.06402439	1.376923077
pfkfb4b	1.06401766	1.253753754
HTRA2	1.063829787	0.989361702
sdccag3	1.063829787	1.786885246
aip	1.06379822	1.376859504
ube2s	1.063694268	1.196078431
spata13	1.063559322	1.022813688
ralgapa2	1.063492063	0.646341463
fbxo38	1.063492063	0.854166667
mettl24	1.063492063	0.971830986
sumf1	1.063492063	1.226415094
atp6v1h	1.063356164	1.066075745
zfr	1.063291139	0.708474576
marcksl1b	1.063278309	0.621599741
vdac2	1.063253928	0.919472503
tbc1d9	1.063157895	0.578125
taok1a	1.063157895	0.85915493
si:ch211-7c8.2	1.063157895	0.933054393
fbxl17	1.063157895	1.054054054
tnnt3b	1.063137033	1.060354917
usp44	1.063063063	0.758928571
rgl1	1.062937063	0.932835821
zcrb1	1.062937063	1.130081301
arl6	1.062893082	1.479674797
USMG5	1.062853907	0.846153846
cry1ab	1.062706271	0.955782313
ganab	1.062686567	0.76119403

casq1b	1.062620424	1.162790698
prkar2ab	1.0625	0.541666667
ss18	1.0625	0.563245823
gria1b	1.0625	0.727272727
ror1	1.0625	0.760869565
hira	1.0625	0.765957447
ice2	1.0625	0.842105263
dachb	1.0625	0.901960784
ttc5	1.0625	0.981651376
si:dkey-172k15.11	1.0625	1.333333333
cabp2b	1.0625	1.363636364
adm2a	1.0625	1.594594595
fam210aa	1.0625	1.981132075
tspan9b	1.062200957	1.434210526
si:ch211-200p22.4	1.062146893	0.859259259
ankrd28b	1.062068966	0.796875
fnbp4	1.062052506	0.799410029
galnt16	1.061946903	0.735632184
hsbp1b	1.061868687	1.47754491
tmem237a	1.06185567	0.684444444
daxx	1.06185567	0.714285714
prrg4	1.06185567	1.020725389
rarga	1.061696658	0.787234043
hsp90ab1	1.061662784	0.709427801
cercam	1.061643836	1.325842697
si:ch211-113e8.11	1.061643836	1.341463415
pip5k1cb	1.061538462	0.468085106
cnot4b	1.061538462	0.747747748
sco1	1.061452514	1.025641026
gphna	1.061349693	0.53125
clic5a	1.061320755	1.21182266
mt-nd2	1.061279593	0.993473715
cbarpb	1.06122449	0.354166667
kirrelb	1.06122449	0.518072289
ube2q1	1.06122449	0.864253394
fbxl4	1.06122449	0.977272727
tgfb2	1.06122449	1.087912088
plcd3b	1.06122449	1.297297297
mt-nd3	1.061169693	0.96331906
thoc3	1.061111111	1.085106383
atp2a1	1.061090226	0.876107254
kirrela	1.061068702	0.851851852
si:ch211-13c6.2	1.061068702	0.972222222
gtf2e1	1.061068702	1.152542373
pigu	1.06097561	1.088607595
foxred1	1.06097561	1.205882353
gnb2l1	1.06095673	0.822786028
icn2	1.060921717	0.855089132
vamp4	1.060869565	0.990338164
fam204a	1.060869565	1.160377358
dusp8b	1.060869565	1.333333333
aplp2	1.060830861	0.661691542
clptm1l	1.060728745	1.080769231
srl	1.060624617	0.925248301
USP34	1.060606061	0.661016949
arl8bb	1.060606061	0.745762712
lmtk2	1.060606061	0.833333333
tecpr1b	1.060606061	0.866666667
ticrr	1.060606061	0.875
chid1	1.060606061	1.114503817
stab2	1.060606061	1.137931034

OGDHL	1.060606061	1.153846154
eif2b1	1.060606061	1.257352941
ppp6c	1.060606061	1.43956044
si:cabz01044764.2	1.060606061	1.444444444
lcmt1	1.060606061	1.774647887
ppp4r2b	1.060498221	1
stoml2	1.060465116	0.939086294
nckipsd	1.060301508	0.975845411
ubb	1.060189573	1.302673797
eif1b	1.060185185	0.820677036
nbr1	1.060185185	1
lmbrd2a	1.060150376	1.148148148
u2af2a	1.06013363	0.806748466
ndufb6	1.060077519	1.059614261
pcdh11_1	1.06	0.4
ubxn7	1.06	0.793103448
peak1	1.06	0.973684211
ints12	1.06	1.020618557
SPATA5	1.06	1.043478261
zgc:171435	1.06	1.365853659
aldh4a1	1.059952038	0.808314088
oclna	1.059945504	1.318493151
elac2	1.05982906	1.045454545
smim7	1.05982906	1.666666667
HNRNPUL1 (1 of many)	1.059811123	0.724091521
leng8	1.059800664	0.949771689
ap2m1b	1.05976431	0.708200213
shisa7a	1.059701493	0.431372549
si:dkey-151g10.3	1.059701493	0.704545455
npdc1b	1.059701493	0.859060403
abhd18	1.059701493	0.983471074
zgc:136791	1.059701493	1.130434783
ogdhb	1.059602649	0.930232558
cntnap2b	1.05952381	0.25
akap6	1.05952381	0.462686567
rpl17	1.059465165	1.011176985
slc35c1	1.059405941	0.74796748
scaf4a	1.059322034	0.671428571
snx18a	1.059322034	1.134502924
apoea	1.059146145	0.934164845
crygm2d15	1.059111692	0.98860038
gpbp1l1	1.059016393	0.81124498
atf5a	1.059016393	1.442060086
rps27a	1.058922559	1.015357557
kif1aa	1.058823529	0.45112782
cnnm4b	1.058823529	1.11627907
zbtb1	1.058823529	1.326530612
si:dkey-27k4.4	1.058823529	2.214285714
gbx2	1.058823529	0.797979798
dcaf11	1.058823529	0.89047619
clec16a	1.058823529	0.95
esm1	1.058823529	0.96
mc1r	1.058823529	0.972222222
zgc:174263	1.058823529	1.058823529
dennd1b	1.058823529	1.071428571
si:ch73-62b13.1	1.058823529	1.083333333
toe1	1.058823529	1.102272727
slc2a10	1.058823529	1.142857143
si:dkey-57h18.1	1.058823529	1.142857143
alg3	1.058823529	1.27480916
ftr04_2	1.058823529	1.333333333

sh2b3	1.058823529	1.4
si:cabz01021430.2	1.058823529	1.4
lyrm7	1.058823529	1.459459459
foxg1c	1.058823529	1.461538462
GDPGP1	1.058823529	1.545454545
si:ch211-74m13.1_2	1.058823529	1.727272727
hhla2a.2	1.058823529	1.933333333
serinc2	1.058823529	1.19005848
appb_2	1.058664869	0.571780148
mrpl40	1.058659218	1.216901408
soul5	1.05861244	1.804123711
enoph1	1.05859375	1.18018018
psmd7	1.058504875	1.319018405
hypk	1.058495822	1.247247247
napba	1.058479532	0.326446281
psmc4	1.058401639	1.265402844
tox4a	1.058394161	0.622641509
si:dkey-88p24.11	1.058350101	1.074204947
psmd13	1.058252427	1.301129235
aes	1.058201058	0.75
nf1a	1.058139535	0.714285714
as3mt	1.058139535	0.861386139
fkbp1aa	1.058108108	0.92721519
si:ch211-162e15.3	1.058091286	1.033149171
CABZ01032476.1	1.058064516	0.926829268
tpma	1.05797955	0.952748386
IGLON5	1.057971014	0.342105263
phactr4a	1.057971014	0.823943662
znf800b	1.057971014	1.365079365
zgc:154093	1.05785124	0.821428571
tbc1d17	1.057777778	0.974619289
xpc	1.057777778	1.348148148
tsc22d1	1.057761733	1.165289256
dock4b	1.057692308	0.725
paxip1	1.057692308	0.797101449
rras2	1.057692308	0.919708029
bend3	1.057692308	0.953488372
neurog1	1.057692308	0.983606557
si:dkey-77f5.6	1.057692308	3.117647059
hoxb5b	1.057591623	1.196428571
rrm2b	1.057575758	1.731818182
st6gal2a	1.057507987	0.693965517
exoc6	1.057471264	0.96969697
myom2b	1.057471264	1.044352044
MEX3C	1.057471264	1.189655172
rpl5b	1.057150965	1.008824113
HSD11B1L (1 of many)	1.057142857	0.791666667
klhdc10	1.057142857	0.962616822
lhfp	1.057142857	0.981595092
rgs11	1.057142857	1.051724138
hip1ra	1.057142857	1.128205128
atg101	1.057142857	1.181372549
si:ch211-261n11.9	1.057142857	1.307692308
glb1l	1.057142857	1.859649123
optn	1.056962025	1.548780488
kdm5c	1.056930693	0.77607362
crebbpa	1.056910569	0.62745098
nadl1.2	1.056818182	0.2
ephb2b	1.056818182	0.735849057
arfrp1	1.056818182	0.816091954
zgc:92873	1.056818182	1.463768116

tomm40	1.056795132	0.923245614
CABZ01079721.1	1.056737589	0.651898734
ubr5	1.056666667	0.649805447
camk2g1	1.056640625	0.743209877
mfhas1	1.056603774	0.935897436
mre11a	1.056603774	0.964285714
wdr59	1.056603774	1.021276596
rbm18	1.056603774	1.24137931
si:ch211-221j21.3	1.056603774	1.3
zgc:66472	1.056603774	1.571428571
eif2b3	1.056521739	1.104265403
smg1	1.056497175	0.675862069
mfap1	1.056426332	1.563876652
zgc:77086	1.056384743	1.31827957
RAPGEF4 (1 of many)	1.056338028	0.490909091
si:ch211-241b2.1	1.056338028	1.68627451
kansl2	1.056277056	0.850877193
siah1	1.056277056	0.904255319
madd	1.05625	0.543103448
gtpbp1	1.0562249	0.866666667
tpp1	1.056179775	1.4375
cp	1.056164384	0.638323353
LUC7L2	1.056074766	0.879120879
mmp11b	1.056074766	1.206521739
prnpa	1.056	0.696969697
pnrc2	1.055944056	0.917545542
raraa	1.055944056	0.62987013
ncapd2	1.055900621	0.777777778
scrt1a	1.055865922	0.833333333
chic2	1.055865922	0.962264151
tjp1a	1.055813953	0.648241206
si:dkey-100n23.4	1.055636896	1.011074919
man2a2	1.055555556	0.527027027
zdhhc9	1.055555556	0.681564246
pard6b	1.055555556	0.696969697
si:ch1073-322p19.1	1.055555556	0.717741935
cdan1	1.055555556	0.76
jarid2b	1.055555556	0.777777778
mtmr7b	1.055555556	0.797202797
cdh10a	1.055555556	0.813953488
lrtm2b	1.055555556	0.886363636
smu1b	1.055555556	1.039215686
ppp1r2	1.055555556	1.042857143
lmod1b	1.055555556	1.090909091
zgc:153146	1.055555556	1.136363636
gdpd4b	1.055555556	1.142857143
sptlc2a	1.055555556	1.166666667
CABZ01064859.2	1.055555556	1.1875
tmem150aa	1.055555556	1.297297297
si:dkey-26i13.8	1.055555556	1.363636364
pex11a	1.055555556	1.811320755
mif4gda	1.055555556	1.84
si:ch211-235i11.3	1.055555556	1.9
nod2	1.055555556	2.111111111
cdc73	1.055555556	0.93220339
si:ch211-202a12.4	1.055481283	1.038529935
actn3b	1.055465368	0.972938473
rpl10a	1.055460561	0.964384529
hhatla	1.054978355	1.029867257
rpl7a	1.054817276	0.837655582
six7	1.054794521	0.574468085

GARNL3	1.054794521	0.6
pcsk7	1.054794521	0.957142857
ahi1	1.054794521	1
hspb2	1.054794521	1
fhit	1.054794521	1.428571429
sesn1	1.054775281	1.207381371
rhogc	1.054744526	1.174273859
adar	1.0546875	1.012195122
fsd1l	1.0546875	1.078947368
dhx8	1.054621849	0.770212766
akr1a1a	1.054545455	0.930555556
brsk2b	1.054421769	0.425
psma5	1.054421769	1.240452617
abhd11	1.054421769	1.302083333
fam193a	1.054347826	0.625
ical1_1	1.054347826	1.234567901
map3k7	1.054298643	1.130681818
rufy1	1.054263566	1.118181818
tal1	1.054216867	0.821917808
SERP1	1.054100228	1.234631148
polr2d	1.054093567	0.827807487
nmd3	1.054054054	0.8775
CABZ01066725.1	1.054054054	0.806451613
b4galt3	1.054054054	0.888888889
galnt14	1.054054054	1
cxadr	1.054054054	1.096551724
si:ch73-39o22.3	1.054054054	1.903225806
ctsba	1.054018445	0.905405405
fabp6	1.053903346	1.415220294
atp5b	1.053876761	0.846194336
aprt	1.053846154	1.068376068
tbc1d5	1.053846154	1.17
mpi	1.053763441	0.874125874
endou	1.053763441	1.146788991
ARHGEF25 (1 of many)	1.053691275	1.125984252
sobpa	1.053571429	0.638297872
zgc:110796	1.053571429	0.674418605
stk26	1.053571429	1.02
ercc1	1.053571429	1.113636364
enc3	1.053571429	1.119617225
si:ch211-173p18.3	1.053571429	1.13125
si:dkey-286f11.2	1.053571429	1.3
map7d1b	1.053571429	0.64028777
rbm42	1.053475936	0.802083333
arid2	1.053435115	0.5
atp5h	1.053385186	1.165894665
dclre1b	1.053333333	1.247787611
cab39_2	1.053191489	0.644444444
hn1a	1.053191489	0.72
rassf7b	1.053191489	1.152173913
rnf141	1.053191489	1.848275862
harbi1	1.053097345	1.219047619
myhz1.2	1.053094749	1.35215424
naa10	1.053037608	0.946459413
zgc:63733	1.052980132	1.679245283
vsx1	1.052884615	1.209026128
zgc:110239	1.052816901	1.109022556
hadhaa	1.052631579	0.965156794
grin2ab	1.052631579	0.277777778
top1mt_2	1.052631579	0.748427673
si:ch211-136a13.3	1.052631579	0.769230769

grk7b	1.052631579	0.774193548
si:ch73-278m9.1	1.052631579	0.827586207
CABZ01083166.1	1.052631579	0.92
lygl1	1.052631579	0.979166667
si:ch211-196f2.7	1.052631579	1
adamts5	1.052631579	1.025641026
zgc:158417	1.052631579	1.1
abcg4a	1.052631579	1.1
fam134b	1.052631579	1.136363636
lpcat1	1.052631579	1.238095238
si:dkey-229e3.2	1.052631579	1.428571429
CABZ01086041.1	1.052631579	1.529411765
bspry	1.052631579	1.533333333
zgc:110821	1.052631579	1.678571429
mhc1lia	1.052631579	1.708333333
C14orf28	1.052631579	1.785714286
si:rp71-36a1.2	1.052631579	2
si:ch73-376l24.6	1.052631579	2.318181818
sap18	1.05244123	1.111658456
sgut1	1.052401747	1.457446809
zc3h18	1.052380952	0.626373626
foxred2	1.052287582	1.157142857
cldnd1a	1.052173913	0.583333333
paplna	1.052173913	0.949494949
dbt	1.052123552	1.1069869
nsd1a	1.052083333	0.536585366
arfgap3	1.052083333	0.836734694
si:ch73-211e3.1	1.051948052	0.394366197
cd276	1.051948052	0.939597315
dusp11	1.051948052	1.068965517
ptpdc1a	1.051948052	1.2
cabp5a	1.051948052	1.223684211
endog	1.051948052	1.663716814
susd6	1.051851852	0.807531381
klhl24a	1.051851852	1.340425532
rnf123	1.051724138	0.813953488
wdr81	1.051724138	0.869565217
sin3aa	1.051612903	0.726618705
znf513	1.051546392	0.747252747
smpdl3b	1.051546392	1.7
ptgdsb.1	1.051511358	1.727219965
lsm12a	1.051502146	1.146919431
myl12.2	1.051428571	0.923295455
cnih1	1.051428571	1.160469667
ap2m1a	1.051404346	0.786343612
prps1a	1.05136612	1.061491935
gpx1a	1.051282051	0.935840708
si:ch211-166g5.4	1.051282051	0.609375
plce1	1.051282051	0.722222222
nelfcd	1.051282051	0.93442623
aqp7	1.051282051	0.975
srrd_1	1.051282051	1.072727273
si:dkey-11c5.11	1.051282051	1.161290323
phykpl	1.051282051	1.219512195
si:dkey-153k10.7	1.051282051	1.311111111
prkg1b	1.051282051	1.824773414
ndufb9	1.05126183	1.037717284
ndufb3	1.051220965	0.857848518
apbb2b	1.051094891	0.86
med12	1.051020408	0.761290323
hlcs	1.051020408	0.857142857

ric8b	1.051020408	1.194117647
rab3b	1.051020408	1.512820513
dhdds	1.050955414	1.005376344
ube2g2	1.050898204	1.190140845
arl4d	1.050890585	1.087759815
grin2aa	1.050847458	0.416666667
fgf3	1.050847458	0.84
zer1	1.050847458	0.913043478
epg5	1.050847458	1.095238095
sars2	1.050847458	1.720930233
cnot6a	1.050746269	0.777777778
spcs1	1.050595238	1.254421769
synpo2la	1.050561798	1.02359882
wwc3	1.050505051	0.84
exoc3	1.050505051	0.933774834
notch3	1.050458716	0.641176471
rab39ba	1.050420168	0.825688073
klf6a	1.050420168	1.207575758
zgc:172253	1.050314465	1.846153846
ttyh1	1.050251256	0.31097561
arl8ba	1.05019305	1.382352941
si:ch73-274k23.3	1.05	0.430493274
slc34a1a	1.05	0.603773585
arsia	1.05	0.611111111
si:dkeyp-26a9.2	1.05	0.857142857
slc6a1l	1.05	0.866666667
rtn3	1.05	0.900326797
ap5z1	1.05	1.138888889
fam84b	1.05	1.179487179
FO704710.3	1.05	1.294117647
si:dkey-245p14.4	1.05	1.407407407
klhl22	1.05	1.538461538
moxd1l	1.05	1.880952381
her4.4	1.05	2.023809524
tmem120a	1.05	2.092105263
aars	1.04989154	1.087051143
nosip	1.049792531	1.402298851
cnrip1a	1.049689441	0.881118881
sh3bp5b	1.049689441	0.571428571
si:ch211-79l17.1	1.049586777	0.699186992
bsk146	1.04950495	0.771428571
cica	1.04950495	0.905882353
myo5aa	1.049382716	0.720430108
ipo11	1.049382716	0.887323944
si:ch211-212o1.2	1.049382716	1.036585366
zfand6	1.049382716	1.381909548
ifrd1	1.049363057	0.976510067
csnk1da	1.049358342	0.988123515
chpf2	1.049295775	1.137614679
tiam1b	1.049180328	0.661016949
zgc:195173	1.049180328	0.857142857
si:ch211-103f14.3	1.049180328	1.304347826
asap1a	1.049180328	0.560283688
faf1	1.049180328	1.114285714
glg1a	1.049132948	0.751748252
rpl9	1.049066769	0.847315436
si:dkey-178e17.3	1.049019608	1.5
10-Sep	1.049001815	1.276635514
tuba1c	1.048967459	0.504803164
snx25	1.048951049	0.84057971
ptgr2	1.048951049	1.736111111

chmp1b	1.048895899	1.418402778
ube3a	1.048850575	0.928571429
kif26ba	1.048780488	0.5
kif19	1.048780488	0.588235294
zgc:175066	1.048780488	0.608333333
gal3st4	1.048780488	0.640449438
dvl2	1.048780488	0.730769231
esco1	1.048780488	0.863013699
gpx8	1.048780488	1.119402985
dedd1	1.048780488	1.632978723
si:rp71-36a1.1	1.048780488	1.75
fubp3	1.048717949	0.891812865
DNM2 (1 of many)	1.048648649	1.201298701
paf1	1.048582996	0.826839827
ccdc6b	1.048543689	0.987951807
cspg5b	1.048543689	1.01010101
CPEB4 (1 of many)	1.048442907	0.712195122
ssfa2	1.048387097	0.701754386
brd9	1.048387097	0.869281046
zfyve19	1.048387097	1.185393258
ankra2	1.048387097	1.5234375
zgc:77849	1.048342541	1.035603715
xrcc6	1.048245614	1.333333333
VASH1	1.048192771	0.431034483
yif1b	1.048192771	0.763779528
nkd1	1.048192771	0.935714286
otud7b	1.048192771	0.941176471
rnf150b	1.048192771	1.065217391
si:dkeyp-57f11.2	1.048128342	1.025252525
slc25a5	1.048121253	0.94496185
sel1l	1.048034934	1.102272727
smarca2	1.048	0.946428571
phf13	1.048	1.275
glulb	1.04797048	1.280333069
ppp2r2ab	1.047923323	0.941605839
acvr1ba	1.047887324	0.848484848
jmjd1cb	1.04784689	0.684210526
tshz1	1.047619048	0.53164557
si:ch211-241b2.4	1.047619048	0.545454545
sf3a1	1.047619048	0.604210526
rin3	1.047619048	0.819672131
usp11	1.047619048	0.897435897
mogat3a	1.047619048	1.01910828
znfl2a	1.047619048	1.069767442
esrrd	1.047619048	1.142857143
als2a	1.047619048	1.1875
pdzrn3a	1.047619048	1.4
parn	1.047619048	0.89010989
psmb4	1.047533093	1.105853051
dcun1d2b	1.047445255	1.036363636
sdcbp2	1.047425474	0.862483311
pkmb	1.047415697	1.080697644
yipf4	1.047297297	1.11928934
yipf6	1.047272727	1.257352941
gpat4	1.047244094	0.909574468
tbc1d4	1.047244094	0.974137931
dlst	1.047220562	0.898066784
ncor2	1.0472103	0.607734807
atg9b	1.047169811	1.172413793
stk11ip	1.047169811	1.294117647
MZT1	1.047120419	1.262032086

scinla	1.047090352	1.373303167
map7d2a	1.047058824	0.621621622
itgb5	1.047058824	0.902097902
dph2	1.047058824	1.625
PAXBP1	1.046948357	1.38410596
pphln1	1.046931408	1.051792829
podxl2	1.046875	0.377777778
susd4	1.046875	1.102040816
tubb2b	1.046846518	0.53425154
polr2gl	1.04679803	1.006276151
ak2	1.046788991	0.950511945
ppm1h	1.046728972	0.642857143
si:dkey-51a16.9	1.046728972	1.043478261
mn1a	1.046666667	0.687116564
add1	1.04664723	0.75
megf11	1.046511628	0.277777778
tsen54	1.046511628	0.918367347
nfatc3b	1.046511628	1
MDFI	1.046511628	1.196721311
cdc14b_2	1.046511628	1.320689655
khnyn	1.046511628	1.516129032
scml2	1.046511628	0.627272727
mdkb	1.046505518	0.914979757
pcdh9	1.046296296	0.357142857
syt9a	1.046296296	0.362745098
sos2	1.046296296	0.725274725
gcc1	1.046296296	1.368421053
slit3	1.046263345	0.646341463
tsc22d2	1.046242775	0.896373057
prpf6	1.04622871	0.726315789
si:ch211-133n4.6	1.04622871	1.290598291
pycr1b	1.046218487	0.761133603
med14	1.046153846	0.623853211
nr2c1	1.046153846	0.941666667
asb7	1.046153846	1.1875
rp2	1.046153846	1.259259259
tmprss4a	1.046153846	1.387931034
stx5a	1.046153846	0.985221675
lgals2a	1.046130952	1.217347956
ephb3a	1.046082949	0.545454545
naga	1.046082949	0.871111111
fkbp3	1.046070461	0.950749465
rfx2	1.045977011	0.714285714
NOP9	1.045977011	0.927835052
pmpcb	1.04595186	1.022357724
dldh	1.045885476	1.076709062
snrnp200	1.04587156	0.681318681
atp5a1	1.045781893	0.817819606
alkbh5	1.045714286	0.80952381
lrtm2a	1.045714286	1.0234375
hectd1	1.045563549	0.916905444
nog3	1.045454545	0.954198473
si:dkey-182g1.2	1.045454545	1.490909091
kcnk6	1.045454545	0.810810811
cd151	1.045454545	1.025210084
usp16	1.045454545	1.322580645
ppp1r11	1.045454545	1.548192771
CABZ01032983.1	1.045454545	0.609375
mpped1	1.045454545	0.672413793
ldlrad4a	1.045454545	0.690140845
bbx	1.045454545	0.72972973

zgc:113119	1.045454545	0.730769231
CU693375.1	1.045454545	0.80952381
si:ch211-208k15.1	1.045454545	0.833333333
bub1ba	1.045454545	1.151515152
ciz1b	1.045454545	1.19047619
amn	1.045454545	1.236842105
ccdc142	1.045454545	1.322033898
tmem243b	1.045454545	1.391891892
zmp:0000001082	1.045454545	1.5
si:ch211-210b2.2	1.045454545	1.533333333
itpkcb	1.04526749	0.991416309
slc7a6os	1.045180723	0.965397924
mt-nd4l	1.045081967	1.461356583
eif4g3a	1.045045045	0.489130435
esyt2a	1.045045045	0.845454545
zfp36l2	1.045016077	0.766666667
fbxw7	1.044871795	0.607843137
nploc4	1.044843049	0.858585859
taldo1	1.044808743	0.803418803
amer1	1.044776119	0.806122449
TBC1D8B	1.044776119	0.883333333
map2k2b	1.044776119	1.089285714
aamp	1.044715447	0.841059603
btf3	1.044675281	0.946018323
psmd3	1.044657097	0.889285714
tnni2b.2	1.044608055	1.329485107
ktn1	1.044594595	0.754409769
dopey2	1.044585987	0.9765625
crebl2	1.044554455	1.074235808
tfdp1a	1.044534413	0.90430622
CEP170B (1 of many)	1.044444444	0.454545455
fam43b	1.044444444	0.541666667
vstm4a	1.044444444	0.793103448
ercc5	1.044444444	0.914893617
ppih	1.044444444	1.020833333
gxylt2	1.044444444	1.152777778
znf395b	1.044444444	1.15885947
slc29a2	1.044444444	1.270833333
rsrc2	1.044444444	1.307116105
tmem167b	1.044444444	1.317647059
si:ch211-153f2.7	1.044444444	1.363636364
cox5aa	1.044413708	1.003512424
mrpl30	1.04434907	1.231316726
ehbp1	1.044247788	0.939759036
eef1a1l1	1.044196131	0.830065992
tpst1	1.044176707	0.656387665
camk1da	1.044117647	0.346153846
cblb	1.044117647	0.771929825
usp22	1.044117647	0.813559322
cdc23	1.044117647	0.898305085
col12a1a	1.044117647	1.051805338
edrf1	1.044117647	1.068965517
rps27.2	1.044115574	0.882592786
ucp2	1.043976349	1.298198544
syt3	1.043956044	0.234375
fnbp1b_1	1.043956044	0.933333333
ift88	1.043956044	1.568965517
taok1b	1.043902439	0.994505495
casq2	1.043744532	1.175686275
pvalb2	1.043725908	0.987995309
rtn4a	1.043713384	1.195533499

nars	1.043572985	1.114441417
crybb1l2	1.043569432	0.677153778
mfsd9	1.043478261	0.666666667
si:ch211-244o22.2	1.043478261	0.747368421
usp32	1.043478261	0.828571429
clasrp	1.043478261	0.855172414
socs5b	1.043478261	0.864864865
dna2	1.043478261	0.894736842
comtd1	1.043478261	0.918032787
si:dkey-84k17.2	1.043478261	0.947368421
cpda	1.043478261	1.012195122
slc48a1b	1.043478261	1.124423963
si:dkey-197c15.6	1.043478261	1.19047619
si:dkey-20i20.7	1.043478261	1.277777778
tada2b	1.043478261	1.34375
zgc:163077	1.043478261	1.4
si:ch211-275j6.5	1.043478261	1.45
lin37	1.043478261	1.464285714
si:dkey-264d12.5	1.043478261	1.578947368
SETD9	1.043478261	1.65
ccdc151	1.043478261	1.85
zgc:136908	1.043360434	0.992462312
hoxb5a	1.043333333	0.652719665
aldoab	1.043285372	0.996286711
uprt	1.043227666	1.039660057
ndufa10	1.043214863	1.132549655
rbp4l	1.043213015	1.004567814
myl13	1.043155216	0.877406544
slc37a4b_2	1.043103448	1.146551724
setd8a	1.042990654	0.862608696
dcun1d5	1.042918455	1.410138249
gng7	1.042895442	1.276657061
nucks1b	1.042767296	1.171383648
krt1-19d	1.04271632	1.183502426
UQCR10	1.042687037	1.074985354
sst2	1.042682927	1.26875
ctdspl3	1.042654028	1.168674699
mrps30	1.042643923	1.120805369
myhc4	1.04258614	1.336448598
selt1a	1.042584435	0.951747088
srgap1b	1.042553191	0.256756757
rad54l	1.042553191	0.928571429
tmem150c	1.042553191	1.222222222
ppil2	1.042553191	1.297142857
prepl	1.042553191	1.375
npas2	1.042553191	1.407407407
vrk1_2	1.042553191	1.478991597
DNAJC16 (1 of many)	1.042553191	1.619047619
tcerg1a	1.042424242	0.639705882
atp5o	1.042372881	0.946174142
psma6a	1.04234122	1.109022556
kmt2cb	1.042253521	0.79245283
CDPF1	1.042253521	1.183333333
commd8	1.042253521	1.461538462
rps5	1.042233619	0.965746093
myt1a	1.042207792	0.493333333
tmem189	1.042168675	1.164670659
lamtor5	1.042168675	1.360946746
zgc:162816	1.042105263	0.730769231
zgc:123035_1	1.042016807	0.455882353
specc1la	1.042016807	0.909090909

hoxc5a	1.042016807	1.339805825
EPB41L1	1.041984733	0.635869565
cox5ab	1.041963016	1.089253188
mrpl53	1.041899441	1.437665782
hnrnpr	1.041884817	0.540034072
ppp1caa	1.041884817	0.710332103
dla	1.041884817	0.734177215
cdkn1ca	1.041884817	0.966666667
asb12a	1.041884817	1.242236025
tmed4	1.041811847	1.05862069
cox6a1	1.041754757	0.924963217
wbp4	1.041666667	0.454545455
EPB41L2	1.041666667	0.577358491
usp54b	1.041666667	0.617647059
arhgef11	1.041666667	0.633027523
si:cabz01062511.2	1.041666667	0.648148148
smad2	1.041666667	0.65408805
dennd6aa	1.041666667	0.714285714
nbeal1	1.041666667	0.796296296
ift140	1.041666667	0.820512821
urp1	1.041666667	0.909090909
gnb1l	1.041666667	0.954545455
zgc:158263	1.041666667	0.993150685
slc9a5	1.041666667	1.052631579
map3k3	1.041666667	1.055555556
poli	1.041666667	1.285714286
zgc:101765	1.041666667	1.352941176
sox21a	1.041666667	1.422535211
gart	1.041474654	0.682080925
mrpl23	1.041441441	1.281639929
IFI30 (1 of many)	1.041322314	0.754601227
gga1	1.041322314	0.862745098
cpsf3l	1.041322314	0.893939394
cdipt	1.041125541	1.235988201
ptprt_2	1.04109589	0.391304348
polr3a	1.04109589	0.777777778
zgc:55733	1.04109589	0.853881279
hrasa	1.04109589	1.13740458
serinc1	1.041059603	0.917089679
immt	1.041019956	1.007633588
fezf2	1.040983607	0.791208791
mief1	1.040983607	0.851851852
nadk2	1.040983607	1.235294118
dcaf7	1.040952381	0.761041903
fndc3a	1.040935673	0.715189873
si:rp71-45k5.4	1.040883978	1.175
mrpl17	1.040871935	1.406153846
elmo1	1.040852575	0.96625222
dhrs3b	1.040816327	0.92481203
PRDM16	1.040816327	0.5
rictorb	1.040816327	0.725
zgc:101562	1.040816327	1.233333333
LPIN3	1.040816327	1.384615385
dpm1	1.040816327	1.661971831
tmem245	1.040816327	1.689655172
ryr3	1.040767386	1.176470588
tprb	1.040697674	0.894308943
fip1l1b	1.040598291	0.777542373
necap1	1.040590406	0.757111597
bccip	1.040540541	1.142034549
n6amt2	1.040540541	1.361111111

ponzr4	1.040540541	2.642105263
gys2	1.040462428	0.902621723
nudc	1.040449438	1.337531486
hs6st2	1.04040404	0.963917526
rnf40	1.04040404	1.168141593
neo1a	1.040358744	0.755681818
dnajc7	1.040298507	1.075645756
lnx2a	1.040268456	0.421052632
daglb	1.040201005	1.398496241
riok2	1.040178571	1.45
zgc:162999	1.040160643	1.514492754
cct5	1.040135192	0.778951487
tmcc2	1.04	0.379746835
DGKI	1.04	0.513761468
pcif1	1.04	0.646153846
mbnl3	1.04	0.666666667
bdkrb1	1.04	0.826086957
znf1153	1.04	0.842105263
zgc:154055	1.04	0.909090909
vta1	1.04	0.942176871
paqr5a	1.04	1
slc47a2	1.04	1.333333333
pde4ca	1.04	1.379310345
ctsa	1.04	1.406015038
kctd17	1.04	1.465116279
si:ch73-221f6.3	1.04	1.470588235
gtf2b	1.03988604	1.053072626
rpl18	1.039805074	1.056846389
rab3ab	1.039761431	0.386533666
nup85	1.039735099	0.769736842
vps37c	1.039735099	0.807142857
fzd10	1.039735099	1.162601626
spink4	1.039702233	1.126491647
tmeff2b	1.03968254	0.576923077
FAM184A (1 of many)	1.03968254	0.770114943
lim2.3	1.03968254	0.857142857
uxs1	1.039647577	1.020942408
rps16	1.039623989	0.956378368
lrrc47	1.03960396	1.158415842
rufy2	1.039568345	1.115384615
inpp4aa	1.039548023	0.836065574
tmem101	1.039548023	0.896103896
CABZ01056631.1	1.039473684	1.016666667
cst14b.1	1.039473684	1.718085106
u2af1	1.039452496	1.062211982
reck	1.039370079	1.065155807
mrps15	1.039344262	1.940298507
glrx3	1.039325843	1.012875536
ndufa8	1.03926097	1.146954987
CNDP1	1.039249147	0.92942743
nf1b	1.039215686	0.717948718
itfg1	1.039215686	0.766081871
zgc:113162	1.039215686	0.88
fastk	1.039215686	0.894117647
entpd6	1.039215686	1.338521401
ISCU (1 of many)	1.039130435	1.27245509
kirrel3l	1.039106145	0.575163399
cdc40	1.039106145	0.913978495
arhgap23a	1.0390625	0.824074074
cecr1a	1.0390625	1.336065574
ppat	1.03902439	0.743421053

tom1	1.03902439	1.455621302
gyg1a	1.038961039	1.05
pomt2	1.038961039	1.073529412
gnas	1.038888889	0.671052632
thoc7	1.038869258	1.064724919
ahcyl2	1.038854806	0.795
sps2	1.038854806	1.058536585
phactr3a	1.038834951	0.545454545
rnf185	1.038834951	1.043062201
tnfaip1	1.038834951	1.445652174
rpl8	1.03863055	0.939960689
zfpm2a	1.038461538	0.309090909
pcloa	1.038461538	0.317073171
NCKAP1L	1.038461538	0.891891892
si:ch211-214j24.14	1.038461538	0.894514768
cyp2aa12	1.038461538	0.909090909
CR352265.1	1.038461538	0.923076923
polg	1.038461538	1.04
ntn4	1.038461538	1.1
vps37a	1.038461538	1.128571429
foxj1a	1.038461538	1.2
BX322597.1	1.038461538	1.318181818
adat3	1.038461538	1.322580645
hes2.2	1.038461538	1.333333333
arl13b	1.038461538	1.6
amph	1.038461538	0.395833333
acat1	1.038398693	1.135384615
mylz3	1.038316214	0.923705783
ap1m1	1.038314176	1.522788204
rnf19a	1.038251366	0.926174497
lim2.5	1.038216561	0.48255814
phox2a	1.038167939	0.65
prpsap1	1.038167939	1
cct6a	1.038153377	0.968797565
slc35a1	1.038135593	0.880184332
tppp2	1.038123167	1.108504399
ttc3	1.038095238	0.956989247
h2afy	1.038095238	1.123595506
CABZ01071406.1	1.037974684	1.236363636
lrrc40	1.037974684	1.243902439
nuak1b	1.037914692	0.983425414
farsb	1.037914692	1.005405405
pon3.2_2	1.037878788	1.30141844
slc39a10	1.037837838	0.930107527
casd1	1.037837838	1.225806452
nell2b	1.037777778	0.432642487
si:ch73-112n4.1	1.037735849	0.26035503
nrg2b	1.037735849	0.4
b4galt6	1.037735849	0.817567568
armc1l	1.037735849	1.027272727
snR50	1.037681159	1.128205128
mkrn1	1.037656904	0.935222672
epb41l3b	1.037643208	0.868632708
utp6	1.037634409	1.708955224
eno3	1.037629494	0.833889174
mal2	1.037593985	1.036585366
nnt	1.037423534	0.862495172
fzr1a	1.037406484	1.055882353
syvn1	1.037383178	0.811320755
zgc:194469	1.037383178	1.258426966
IZUMO1R	1.037313433	0.833333333

map4k5	1.037313433	0.953488372
vps51	1.037313433	1.024193548
akap9	1.037267081	0.887218045
fgf12b	1.037267081	0.992424242
actr3_1	1.037127504	0.892387405
ift80	1.037037037	0.766233766
tie1	1.037037037	1.419354839
CU693369.1	1.037037037	0.627118644
glra4b	1.037037037	0.692307692
abcg1	1.037037037	0.833333333
fh	1.037037037	0.837209302
abcc3	1.037037037	1.048780488
stap2b	1.037037037	1.666666667
trmt12	1.037037037	2.411764706
6-Mar	1.036931818	0.824742268
ptgr1	1.036893204	0.912152269
si:dkey-69o16.5	1.036789298	1.041942605
mlip	1.036764706	0.828767123
acta1b	1.036706308	0.900278448
ephb4b	1.036697248	0.807017544
zdhhc24	1.036697248	1.044247788
smim19	1.036697248	1.371681416
rnf146	1.036630037	0.868217054
gpd1a	1.036619718	1.16091954
bin1a	1.036585366	0.6
prdm1a	1.036585366	0.985714286
rest	1.036585366	1.042857143
CHST6	1.036585366	1.269230769
spop	1.03654485	0.976744186
tsr2	1.03652968	1.181451613
hif1ab	1.03652968	0.671568627
ndufb5	1.036403751	1.101505585
eno1a	1.036380173	0.994470438
bmpr1bb_2	1.036363636	0.837209302
ssrp1b	1.036363636	0.950617284
haus4	1.036363636	0.961538462
ascl1a	1.036363636	0.989361702
plekhm2	1.036363636	1.228571429
ttpal	1.036363636	1.719512195
rpl22l1	1.036347251	1.193029491
vps37b	1.03626943	0.653179191
rab3gap1	1.036231884	0.805970149
zmpste24	1.036184211	1.305785124
si:ch211-248k15.2	1.036144578	0.918918919
coa6	1.036111111	1.448
zmiz1a	1.036082474	0.605442177
sox21b	1.036036036	0.45045045
ITGB1BP2	1.036036036	0.872881356
sf3a2	1.036016949	0.599277978
ier3ip1	1.035940803	1.441108545
zgc:112052	1.035928144	1.207692308
wasf3b	1.035842294	0.586805556
si:ch211-198a12.6	1.03575419	1.294964029
BAHCC1 (1 of many)	1.035714286	0.619047619
si:ch73-116f3.3	1.035714286	0.80952381
dcxr	1.035714286	0.866935484
HNRNPUL2	1.035714286	0.925925926
zmiz1b	1.035714286	1.095238095
si:rp71-1h20.3	1.035714286	1.161290323
plxnb2b	1.035714286	1.176470588
znf142	1.035714286	1.2

nmnat2	1.035714286	1.27
iqcb1_1	1.035714286	1.3
SMIM10	1.035714286	1.558823529
bag1	1.035587189	1.847533632
ZNF423	1.035573123	0.621052632
dhrs13l1	1.035514019	1.96037296
taf5	1.035460993	0.711711712
syap1	1.035422343	1.016286645
cirbpa	1.035405517	0.998063356
psmb5	1.035346097	0.893159108
mtmr2	1.035294118	1.165517241
crygm2d19	1.035233668	0.962472406
tspan4a	1.035211268	0.763358779
sec22a	1.035211268	1.544
zgc:162944	1.035190616	1.177022274
dcaf12	1.035175879	0.70984456
ptbp2b	1.03515625	0.513157895
ociad1	1.035149385	1.398039216
lims2	1.035135135	1.450980392
fzd3b	1.035087719	0.488888889
rims2b	1.035087719	0.494117647
znf740a_1	1.035087719	0.731481481
creb3l1	1.035087719	0.824561404
ubald2	1.035087719	1.159574468
hmg20a	1.035087719	0.792207792
mybpha	1.035051546	0.883575884
ak4	1.035	1.455128205
mmp15a	1.034965035	0.950819672
trim35-40	1.034883721	1.564516129
per2	1.034696406	0.827470687
bsg	1.034694882	1.466965286
emc2	1.03468781	0.821888412
rims2a	1.034682081	0.897106109
grpel2	1.034542314	1.396678967
CNNM1	1.034482759	0.55
sipa1l2	1.034482759	0.611702128
gpr101	1.034482759	0.722222222
tmed8	1.034482759	0.745762712
ptk2ab	1.034482759	0.757894737
FNDC10	1.034482759	0.76344086
kcnab1b	1.034482759	0.808510638
gpatch3	1.034482759	0.875
itga10	1.034482759	0.961538462
timp2a	1.034482759	0.998347107
d2hgdh	1.034482759	1.052631579
si:ch73-120g24.4	1.034482759	1.117647059
foxj1b	1.034482759	1.25
flnb	1.034482759	1.408163265
FASTKD5	1.034482759	1.428571429
si:cabz01071912.2	1.034482759	2.111111111
sart1	1.034416826	1.070175439
dda1	1.034375	1.396946565
nrbp1	1.034334764	0.978835979
elovl1a	1.034313725	1.214285714
gtf2a1	1.034246575	1.075471698
gna13b	1.034246575	1.083969466
trpc1	1.034188034	0.740740741
MEGF9 (1 of many)_1	1.034188034	0.775193798
chn1	1.034090909	0.621827411
st3gal2	1.034090909	0.628571429
socs5a	1.034090909	0.668965517

colq	1.034090909	1.326086957
badb	1.034090909	1.569892473
anxa6	1.034031414	1.008849558
usf1	1.034013605	1.189300412
jak1	1.033962264	0.827160494
slc43a2a	1.033923304	1.126721763
derl3	1.033898305	1.11627907
dse	1.033898305	0.84496124
acaca	1.033898305	0.87804878
si:dkey-23p11.2	1.033898305	1.333333333
CABZ01041962.1	1.033898305	1.375
slc50a1	1.033898305	1.5
sgcb	1.033826638	1.433908046
cldn7a	1.033755274	0.692883895
uqcc3	1.033755274	1.412037037
si:ch211-117k10.3	1.033707865	0.823943662
ndufs4	1.033691756	1.032653061
rerea	1.033613445	0.534883721
scaf4b	1.033613445	0.669354839
camk2b1	1.033519553	0.776523702
sec62	1.033492823	1.313069909
ube2f	1.033472803	1.18556701
ppp1r13ba	1.033333333	0.776923077
zgc:63470	1.033333333	1.037037037
mkl2a	1.033333333	1.090909091
kctd13	1.033333333	1.099173554
si:dkey-71l1.1	1.033333333	1.117647059
oaz2b	1.033333333	1.188888889
tti2	1.033333333	1.19047619
senp8	1.033333333	1.28
tigarb	1.033333333	1.357142857
si:rp71-1h20.5	1.033333333	1.5
nfkbiz	1.033333333	1.933333333
nfil3	1.033210332	1.040880503
tuba8l3	1.033182504	0.68385346
letm2	1.033149171	0.955621302
wdr21	1.033112583	1.416666667
qrich1	1.033057851	0.673366834
eef1a1a	1.033039648	1.050847458
plxnb1a	1.032967033	0.632911392
cmc2	1.032967033	1.574324324
spcs3	1.032921811	1.253947368
mia3	1.032894737	1
CABZ01089963.1	1.032786885	0.496350365
iqsec3a	1.032786885	0.58974359
ly75	1.032786885	0.854545455
cnot8	1.032786885	0.976430976
si:dkey-86e18.1	1.032786885	1.5
atp6v1b2	1.032786885	1.307236062
mrps5	1.032727273	0.890410959
ube2e3	1.032648126	0.72027972
plch2a	1.032608696	0.492537313
snrpe	1.032581454	1.015384615
cox10	1.03257329	0.985559567
gtf2f1	1.03255814	1.189873418
si:dkey-205h13.2	1.032527106	1.114806017
ube4a	1.032520325	0.87628866
cbx4	1.032520325	1.4375
kdelr2b	1.03250478	0.824662813
SLC41A3	1.032414911	0.923794712
ehd3	1.032407407	1.310077519

sema5bb	1.032258065	0.631578947
sap130b	1.032258065	0.681818182
hs3st3b1a	1.032258065	0.6875
wrnip1	1.032258065	0.793103448
zgc:110329	1.032258065	1.185483871
zbtb3	1.032258065	1.204545455
tph1b	1.032258065	1.28
sptlc1	1.032258065	1.333333333
mtmr8	1.032258065	1.75
ctbp1	1.032200358	0.519038076
aldh18a1	1.032110092	0.812865497
iqgap1	1.032110092	0.989583333
atp6v1g1	1.032094058	1.18677792
sfpq	1.032051282	0.664540816
si:ch211-261d7.3	1.032051282	0.866666667
snrpb2	1.032	1.130769231
commd2	1.032	1.2760181
h3f3a	1.031946508	0.992596332
col1a1b	1.031937735	0.841454545
ptpn23b	1.031914894	1.163636364
nagk	1.031914894	1.5
cyp2n13	1.031914894	2.520467836
smad6a	1.031847134	1.043478261
hes6	1.031818182	0.53256705
cryaa	1.031818182	1.243697479
lamtor1	1.031818182	2.186915888
chrna6	1.031746032	0.772727273
id1_1	1.031652989	1.301863354
cdk7	1.03164557	1.273255814
ei24	1.031531532	1.212121212
zfand3	1.031456954	0.760655738
zgc:194578	1.03125	0.513761468
ldhd	1.03125	0.714285714
rnf168	1.03125	0.921568627
gmcl1	1.03125	1.084745763
ppm1nb	1.03125	1.143884892
wipi2	1.03125	1.208955224
vax1	1.03125	0.535714286
rhbdl1	1.03125	0.764705882
zgc:174160	1.03125	0.805555556
proca1	1.03125	0.85
si:ch73-144d13.8	1.03125	1.142857143
tmem104	1.03125	1.346938776
amfr	1.031007752	0.841860465
actb2	1.030988015	0.723517637
gpx4b	1.030953218	0.97826087
MTFP1	1.030927835	1.026717557
nol11	1.030927835	1.144278607
itsn1	1.030864198	0.704
papd4	1.030864198	1.684684685
ppm1bb	1.030852995	0.926724138
cyc1	1.030826141	0.894280079
tnpo3	1.030769231	0.89380531
hnrnpa0l_1	1.030766526	0.745361166
atl1	1.030701754	0.43315508
erp44	1.030651341	1.00407332
lrp6	1.030612245	0.730337079
TRAPPC9	1.030612245	0.875
ctdnep1a	1.030612245	1.09
ccnd2b	1.030612245	1.506329114
ASPH	1.030612245	1.605633803

ssh2a	1.030534351	0.957983193
si:ch211-225b11.1	1.030534351	1.631578947
mdka	1.030525721	0.722531646
aldoaa	1.030505483	1.382276423
pabpc1a	1.030465212	0.762200892
ciarta	1.03043022	1.370121131
ap2a1	1.030349014	0.641666667
shisa9a	1.03030303	0.797752809
zgc:101559	1.03030303	0.911764706
METRNL (1 of many)	1.03030303	0.928571429
smad3b	1.03030303	1.023809524
deaf1	1.03030303	1.166666667
ufsp1	1.03030303	1.633333333
rpp21	1.03030303	2.133333333
ndp	1.03030303	2.769230769
tnnc2	1.030271139	1.044249542
bnip4	1.030150754	2.12
cul4b	1.030136986	1.171641791
CABZ01052431.1	1.030120482	1.039215686
si:ch211-5k11.12	1.030018762	0.608281771
rprd2a	1.03	0.582278481
slit1a	1.03	0.609467456
spopla	1.03	0.760869565
tnnt2a	1.03	1.77852349
sec11a	1.029968454	1.431407942
sec24b	1.02994012	0.688741722
myom1b	1.02992278	1.442708333
dbi	1.029871977	1.065689468
ints9	1.029850746	0.716666667
rpap1	1.029850746	0.862068966
prim2	1.029850746	0.984496124
mrpl37	1.029850746	1.019402985
atxn1a	1.029850746	1.288461538
stk25a	1.029850746	1.558823529
wdr33	1.029850746	0.73964497
snrkb	1.029850746	1.335365854
gdi1	1.029783394	0.904109589
bdh2	1.029761905	1.698529412
trappc10	1.02970297	0.863013699
fam126a	1.02970297	0.987654321
pcdh1a3	1.029411765	0.571428571
sorcs2	1.029411765	0.638297872
avpr1aa	1.029411765	0.895833333
tmem30ab	1.029411765	0.928961749
adcy9	1.029411765	1.06
tbc1d22a	1.029411765	1.169811321
nr1h5	1.029411765	1.236842105
MSANTD2	1.029411765	1.272727273
si:ch73-380l3.1	1.029411765	1.333333333
hoxd4a	1.029411765	1.829787234
rpl4	1.029285905	0.968968096
arf3a	1.029239766	1.167938931
uba2	1.029227557	0.80861244
ccdc124	1.029207232	1.099518459
vgll2b	1.02919708	0.752747253
hacd3	1.029166667	1.196153846
gnai2b	1.029126214	0.862903226
si:ch211-285f17.1	1.029126214	0.896907216
col1a2	1.029119442	0.839434578
ergic2	1.029090909	1.313084112
psmc1b	1.029007634	0.927357032

prkx	1.028985507	1.326086957
usf2	1.028901734	1.280898876
opn9	1.028846154	0.794392523
phf20b	1.028846154	0.89010989
lnpa	1.028846154	0.915384615
gdap2	1.028846154	1.180851064
csnk1db	1.028820961	0.909185804
rab5aa	1.0288	0.756052142
edem3	1.028776978	1.095022624
ndufs5	1.028773585	0.955008489
hgd	1.028753994	1.084931507
pcdh17	1.028653295	0.643478261
myhz1.3	1.028603022	1.103462874
pear1	1.028571429	0.736111111
si:ch211-163l21.4	1.028571429	0.833333333
slc30a6	1.028571429	0.892857143
brinp3a.1	1.028571429	0.952941176
si:ch211-150o23.3	1.028571429	1.058139535
nfkb2	1.028571429	1.086956522
mul1a	1.028571429	1.142857143
pycr1a	1.028571429	1.166666667
CABZ01054391.1	1.028571429	1.185185185
kif1b	1.028571429	0.457943925
rps13	1.028566882	0.843529975
cds2	1.028446389	0.927170868
ndc1	1.028368794	1.136690647
dap1b_1	1.028340081	0.860247284
atp6v0e1	1.028301887	1.21038961
gnpda1	1.028225806	1.231316726
FTL	1.028208745	1.136176066
arf2b	1.028195489	0.838974359
acvr2b	1.028169014	0.525641026
fbxo11a_2	1.028169014	0.756476684
crls1	1.028169014	1.088235294
bida	1.028169014	1.1875
fam50a	1.028169014	1.600694444
pbx1a	1.028089888	0.653225806
pde7a	1.028089888	0.8
entpd4	1.028037383	0.719512195
slc33a1	1.028037383	1.084210526
si:dkey-56m19.6	1.028011204	1.215805471
mon2	1.027932961	0.641975309
rnf14	1.027932961	1.067708333
apoc2	1.027860327	1.106195702
irx6a	1.027777778	0.52
notch1a	1.027777778	0.575757576
znf236	1.027777778	0.638888889
si:dkey-46i9.6	1.027777778	0.790697674
mettl3	1.027777778	0.875
dzip1	1.027777778	0.883116883
PNPLA2	1.027777778	1.322580645
zgc:162936	1.027777778	1.827586207
cdkl1	1.027777778	0.798076923
polr2i	1.027777778	1.684684685
gfer	1.027522936	1.176470588
sepp1b	1.027444254	1.211356467
afg3l2	1.027439024	1.174418605
adarb1b	1.02739726	0.673913043
fam171a1	1.02739726	0.784482759
cabp1a	1.02739726	0.985294118
gabpb2a	1.02739726	1.14953271

tuba8l4	1.027390312	0.655381944
g6pd	1.02734375	1.435
cryba4	1.027315754	0.995565749
zgc:86598	1.027310924	0.792237443
mfn2	1.027237354	1.030456853
elof1	1.027155465	1.160201149
arhgap21b	1.027027027	0.776397516
kcnc3a	1.027027027	0.375
ensaa	1.027027027	0.616666667
wnk1b	1.027027027	0.952
acsl1b	1.027027027	0.969924812
si:dkey-147f3.8	1.027027027	1.019607843
atp13a3	1.027027027	1.107142857
si:dkey-15h8.15	1.027027027	1.692307692
ndufaf5	1.026819923	1.010989011
acadsb	1.026690391	0.741721854
tmem131	1.026666667	0.960893855
nup58	1.026666667	0.768
mtx1a	1.026666667	0.852941176
rpl18a	1.026626407	0.941793124
tp53rk	1.026595745	1.093264249
cep170aa	1.026548673	0.421052632
dhx36	1.026548673	1.085365854
gorasp2	1.026530612	0.627615063
srsf1b	1.026506024	0.816384181
tpra	1.026490066	0.85046729
myom3	1.026490066	1.058441558
lonp1	1.026490066	1.076335878
ptp4a2b	1.026455026	1.001412429
prdx5	1.026349614	0.980275229
gtf2a1l_2	1.026315789	0.656565657
rmi2	1.026315789	0.695652174
si:ch211-284e13.4	1.026315789	0.714285714
apobb.2	1.026315789	0.761904762
bnip3lb	1.026315789	0.975609756
vapal	1.026315789	1.003355705
cept1b	1.026315789	1.081967213
ubxn8	1.026315789	1.230769231
si:dkeyp-87e7.4	1.026315789	1.231707317
si:ch73-182a11.2	1.026315789	1.23255814
pnocb	1.026315789	1.305555556
si:ch73-199k24.2	1.026315789	1.636363636
arid1ab	1.026315789	0.534759358
mrpl16	1.026272578	1.03268945
klhl43	1.026234568	0.909365559
zgc:161969	1.02617801	1.27672956
mob2b	1.026086957	0.720930233
ctc1	1.025974026	0.722222222
pcbd1	1.025974026	1.183970856
psmc3	1.025925926	1.206735267
edc3	1.025862069	1.657534247
sulf2a	1.025806452	0.521367521
ubc	1.025800085	1.090661939
rhoac	1.025695931	1.041189931
flrt1a	1.025641026	0.379310345
ptprga	1.025641026	0.615384615
hcfc2	1.025641026	0.731707317
cylda	1.025641026	0.773195876
jmjd6	1.025641026	0.863013699
prdm9	1.025641026	0.909090909
mtx3	1.025641026	0.946428571

ftr97_1	1.025641026	1.409090909
si:dkey-121n8.4	1.025641026	1.7
spred1	1.025568182	0.704697987
rnf128a	1.025559105	1.134020619
crygm2d21	1.025528914	0.929501085
scn8aa	1.025423729	0.555555556
triob	1.025423729	0.5875
tecpr1a	1.025423729	0.765432099
si:dkey-16p21.8	1.025415677	1.106288032
asna1	1.025404157	1.056886228
klf17	1.025316456	0.857142857
si:ch1073-396h14.1	1.025316456	1.271428571
gtf2h2	1.025316456	1.472727273
si:ch211-142h2.1	1.025316456	2.15625
CABZ01052815.1	1.025252525	0.928205128
chchd3a	1.025242718	0.991902834
proza	1.025236593	0.492163009
hnrnpub	1.025220681	0.739329268
pan3	1.025210084	0.670212766
npepl1	1.025174825	0.794973545
eps15l1a	1.025	0.740740741
afap1l2	1.025	0.884955752
hmbsa	1.025	0.93495935
si:dkey-31g6.6	1.025	1.082096934
hecw2b	1.025	0.637681159
si:ch73-181d5.4	1.025	0.678571429
pacs2	1.025	0.964285714
xpo1b	1.025	0.969162996
tbc1d10ab	1.025	1.04
map9	1.025	1.342857143
kcnj1a.1	1.025	1.566666667
si:ch211-117m20.5	1.024988844	0.92842395
ctdsplb	1.024943311	0.862660944
pabpc4	1.02490217	0.963041503
psmc6	1.024896266	1.322617681
zgc:77262	1.024861878	0.530054645
cln3	1.024793388	0.973333333
armt1	1.024793388	1.397959184
ttc32	1.024793388	2.010526316
kdm6al	1.024691358	0.68
mboat1	1.024691358	1.178082192
im:7138535	1.024691358	1.852459016
rnf13	1.024691358	1.339393939
si:dkey-7j14.5	1.024640657	1.271551724
otx1b	1.024590164	0.479166667
casc3	1.024489796	0.686567164
mrps7	1.024464832	1.080597015
txndc12	1.024456522	1.132686084
zgc:114130	1.024390244	0.545454545
zgc:77375	1.024390244	0.619469027
si:dkey-75a21.2	1.024390244	1.105263158
poc1b	1.024390244	1.111111111
hdac4	1.024390244	1.28
mrpl47	1.024390244	1.339041096
uckl1b	1.024390244	1.376237624
cishb_2	1.024390244	1.458333333
ptgesl	1.024324324	1.401215805
ik	1.024320458	0.838016529
uqcrc2a	1.024217962	0.942727273
eif5a	1.024145171	0.842657846
ap4b1	1.024096386	0.833333333

cog4	1.024096386	1.151515152
psmd12	1.024048096	1.6167979
fntb	1.024038462	0.90776699
gucy1a3	1.024	0.857142857
sec31b	1.024	0.901960784
aco2	1.023960067	0.976359338
rplp2l	1.023952417	0.919855312
kdm5bb	1.023941068	0.599078341
si:dkey-78l4.14	1.023941068	1.392927308
cuedc1a	1.023809524	0.462264151
pkig	1.023809524	0.958677686
anapc7	1.023809524	1.094339623
bnip2	1.023809524	1.243243243
uncx	1.023809524	1.252747253
hmg20b	1.023809524	1.41509434
si:dkey-175m17.7	1.023809524	0.5
guca1b	1.023809524	0.767123288
CELA1 (1 of many)_7	1.023809524	1.15
kcnj3a	1.023809524	1.184210526
tmem107l	1.023809524	1.34375
tardbpl	1.023706897	0.787037037
gna11a	1.023648649	0.846889952
ccdc102a	1.023584906	0.743589744
znf319	1.023584906	0.846153846
dab1a	1.023529412	0.333333333
foxj3	1.023529412	0.666666667
dkk3a	1.023529412	0.703125
CT573476.1	1.023529412	0.818181818
ufl1	1.023529412	0.944785276
si:ch211-122f10.4	1.023529412	1.090909091
rad21a	1.02351314	0.803610108
cd99l2	1.023469388	0.907202216
rxrba	1.023460411	0.952991453
uri1	1.0234375	1.113360324
slc16a3	1.023323615	0.934640523
lrrc75ba	1.023255814	0.442857143
jupb	1.023255814	0.472222222
mapkbp1	1.023255814	0.575
zbtb21	1.023255814	0.898550725
si:ch73-111m19.2	1.023255814	0.944444444
tdrkh	1.023255814	1.297297297
kdm5ba	1.023188406	0.638888889
nsfl1c	1.023109244	0.844243792
smg5	1.023041475	0.773684211
gpr182	1.022988506	0.9140625
gata3	1.022988506	0.476190476
samm50	1.022988506	1.074468085
sf1	1.02295082	0.63028169
sgip1a	1.022727273	0.453125
si:ch1073-55a19.2	1.022727273	0.734693878
cwf19l2	1.022727273	0.77027027
tnksa	1.022727273	0.849315068
baiap2l1b	1.022727273	0.857142857
CC2D1A	1.022727273	0.880597015
brms1lb	1.022727273	0.921052632
pdxp	1.022727273	0.957746479
ankrd6b	1.022727273	1.078947368
fkbp10b	1.022727273	1.5
ppdpfa	1.022682446	0.666086957
agla	1.022675737	0.834975369
evla	1.022624434	0.829015544

cog3	1.022556391	0.946902655
dpysl4	1.022522523	0.620481928
nrp1a	1.022508039	0.61752988
wdr5	1.0225	0.956873315
elk1	1.02247191	1.047619048
rgs7a	1.02247191	1.150793651
tcea2	1.02238806	1.105263158
senp3b	1.022346369	1.377777778
apoa1bp	1.022321429	1.165938865
napab	1.022271715	1.324390244
fbxl18	1.022222222	0.608695652
numbl	1.022222222	0.666666667
si:dkey-65j6.2	1.022222222	0.94
gcat	1.022222222	0.972972973
rpp25l	1.022222222	1
prdx3	1.022163121	1.044846577
axin1	1.022123894	0.822335025
lmbrd1	1.022099448	1.062893082
si:dkey-95j14.1	1.022058824	0.782608696
ARID5B	1.021978022	0.626666667
SLC19A1	1.021978022	1.457142857
xpo1a	1.021978022	0.859296482
sdhaf2	1.021978022	0.917647059
atp2c1	1.02189781	0.728070175
hn1l	1.021857923	0.800271739
rdh12	1.021857923	0.926829268
rgs7bpa	1.02173913	0.623188406
slc23a2	1.02173913	1.222222222
FAM72B	1.02173913	1.424242424
si:dkey-229b18.3	1.02173913	1.430769231
pak1	1.021645022	0.47027027
cyth3b	1.021582734	0.771929825
zc3hc1	1.021582734	0.985815603
smim14	1.021582734	1.576576577
atf1	1.021563342	1.291666667
si:ch211-161h7.5	1.021563342	2.666666667
PSMG4	1.021551724	1.387900356
hoxb4a	1.021505376	0.628205128
prdm13	1.021505376	0.793103448
rcbtb2	1.021505376	0.830985915
SLC27A2 (1 of many)	1.021505376	1.014814815
tra2a	1.021466905	1.038745387
crot	1.021390374	0.971428571
tmem126a	1.021333333	1.128
ANKRD9 (1 of many)	1.021276596	0.515151515
lbx1a	1.021276596	0.677419355
pcyox1l	1.021276596	0.958333333
si:ch211-93g23.2	1.021276596	0.966666667
cecr5	1.021276596	1.40625
enthd2	1.021276596	1.730769231
rpl13	1.021234051	0.909255005
hey1	1.021164021	1.282258065
si:ch73-199e17.1	1.021126761	0.908163265
mtif3	1.021126761	0.983870968
kctd6a	1.021126761	1.545454545
emc10	1.021077283	0.797066015
lfng	1.021077283	0.90821256
anks1b	1.021052632	0.552631579
npsn	1.020979021	1.154589372
tle3b	1.020942408	0.711656442
appbp2	1.020887728	1.088652482

mnx1	1.020833333	0.533333333
mfsd5	1.020833333	0.976744186
trim109	1.020833333	1.197530864
dgcr6	1.020833333	1.4
rpl3	1.020813932	0.863781003
ccdc12	1.020725389	1.433862434
scaf1	1.020689655	0.631355932
adck3	1.020661157	1.164882227
gpr107	1.020661157	1.380530973
nek6	1.020618557	1.072727273
cox6c	1.020606301	1.155021067
tefa	1.020425902	2.041070317
si:dkey-243i1.1	1.020408163	0.658536585
atp1a1a.5	1.020408163	0.846153846
taf5l	1.020408163	0.931818182
nudt22	1.020408163	1.107142857
pax1a	1.020408163	1.161290323
eif5	1.020392157	0.881196581
rtn4b	1.020325203	1.029885057
rbm14a	1.020304569	0.662857143
dhps	1.02027027	0.97810219
enah	1.02020202	1.035242291
ptrhd1	1.02020202	1.112244898
agmo	1.02020202	1.600917431
chaf1b	1.02	0.666666667
cd9a	1.02	0.799435028
bap1	1.02	0.809917355
abhd3	1.02	0.833333333
lsm14aa	1.02	0.888349515
zgc:172271	1.02	0.949152542
slc2a8	1.02	1.294117647
s100t	1.019946809	1.481415929
kdsr	1.01986755	1.558139535
chkb	1.01980198	1.229885057
lmo4a	1.01980198	1.38410596
fastkd2	1.019736842	1.384615385
ost4	1.019672131	0.93608074
zgc:77784	1.019607843	0.435897436
pax7a	1.019607843	0.5
asnsd1	1.019607843	1.575342466
sox2	1.01953125	0.635
zswim8	1.019512195	0.76875
fbxo22	1.019480519	1.021428571
cmbl	1.019455253	1.342723005
serpini1	1.019417476	0.566473988
mfsd13a	1.019417476	0.75257732
sf3b2	1.019298246	0.614982578
tollip	1.019283747	1.109181141
atp6v1c1a	1.019230769	1.408980583
sema4c	1.019230769	0.61
ek1	1.019230769	0.756756757
nav2b	1.019230769	0.836734694
AREL1	1.019230769	0.986666667
si:cabz01090193.1	1.019230769	1.05
styx	1.019230769	1.162601626
dtx4	1.019230769	1.235294118
phyhd1	1.019230769	1.315589354
slc30a8	1.019230769	1.358974359
il15	1.019230769	1.372881356
ppp1r15a	1.019230769	1.653846154
tor1	1.019138756	1.275280899

anxa1a	1.019110926	1.244181883
chd1	1.019047619	0.627906977
CABZ01100338.1	1.019047619	0.913461538
wasf1	1.018987342	0.46961326
tp63	1.018957346	0.87826087
yipf3	1.018957346	1.095238095
hmgb2b	1.018933732	0.799032035
pbx4	1.018932874	0.581105169
si:dkey-33i11.9	1.018918919	1.133540373
cenpe	1.018867925	0.701298701
ablim1a	1.018867925	0.842105263
ssh2b	1.018867925	0.880597015
ecsit	1.018867925	1.093333333
gabarapb	1.018838305	1.252396166
hbbe1.1	1.018736383	0.644408029
mtx1b	1.018691589	1.02247191
gpat3	1.018666667	1.882352941
hiat1b	1.01863354	0.785276074
mpped2a	1.018518519	0.530201342
znf576.2	1.018518519	0.880184332
dgcr8	1.018518519	1.105590062
MTERF1	1.018518519	1.142857143
otulinb	1.018518519	1.523809524
FO082779.1	1.018518519	1.575757576
adam28	1.018518519	1.588235294
kpna1	1.018469657	0.970099668
zgc:171566	1.018461538	1.598425197
rps2	1.018419453	0.795083804
si:ch211-195b13.1	1.01836394	1.758364312
ism1	1.018292683	0.993975904
dctn2	1.018276762	0.724637681
prrx1b	1.018181818	0.982142857
CABZ01118758.1	1.018181818	0.875
TNNC2 (1 of many)	1.01814059	0.879795396
rbm22	1.018115942	0.733624454
neurod6b	1.018050542	0.701149425
rfc5	1.018050542	1.024054983
gabarapa	1.018036072	1.287762906
si:ch73-375g18.1	1.018018018	1.02020202
mlf2	1.017969452	0.803314917
gnl1	1.01793722	0.773662551
si:ch1073-456m8.1	1.01793722	0.964646465
STX3 (1 of many)	1.017857143	0.858585859
alkbh3	1.017857143	1.034482759
ttll12	1.017857143	1.084745763
noxo1a	1.017857143	1.574468085
tuft1a	1.017751479	2.568965517
si:ch73-386h18.1	1.017699115	0.453333333
abhd10a	1.017699115	1.231707317
coasy	1.017647059	0.910828025
ca15a	1.01754386	1.518218623
si:dkey-40c11.1	1.01754386	0.790697674
piga	1.01754386	1.219512195
wu:fe05a04	1.01754386	1.307692308
si:dkey-11e23.4	1.01754386	1.470588235
ncoa5	1.017509728	0.699316629
isca2	1.017492711	1.431372549
cox8b	1.017467249	0.947530864
ogfod1	1.017391304	0.857142857
smox	1.017391304	1.00408998
vps50_2	1.017391304	1.163043478

ckmb	1.017383694	0.911786754
pcm1	1.01734104	0.857142857
smek1	1.017326733	0.726744186
mrps34	1.017268446	1.278350515
nhej1	1.017241379	0.753623188
kng1	1.017241379	0.8
dtwd2	1.017241379	0.891304348
nek4	1.017241379	1.27027027
asb8	1.017142857	0.942446043
eif2b4	1.017142857	1.108108108
id3	1.017123288	1.330291971
ndel1b	1.017045455	1.224637681
ppiab	1.01704173	0.786117913
si:ch211-129p13.1	1.016949153	0.333333333
cdc25b	1.016949153	0.642857143
vps13c	1.016949153	0.976190476
cep63	1.016949153	1.06779661
snx17	1.016949153	1.336842105
eif2s3	1.016937435	0.944535609
si:dkey-183j2.10	1.016877637	0.956756757
arhgef7b	1.016806723	0.978947368
zgc:153867	1.016764839	0.942672414
mef2cb	1.016759777	0.78
znf687b	1.016666667	0.495145631
NAV1 (1 of many)	1.016666667	0.676470588
pcdh2aa15	1.016666667	0.777777778
bmpr2b	1.016666667	0.985915493
CABZ01077218.1	1.016666667	1.588235294
slc25a11	1.016625104	0.973275862
pkp3b	1.016528926	0.714285714
coq4	1.016528926	1.324074074
miox	1.016460905	1.111764706
svopa	1.016393443	0.35483871
dock6	1.016393443	0.87755102
tmod1	1.016393443	1.409090909
cluap1	1.016393443	1.875
rpl15	1.016331116	0.910259172
FRMD5 (1 of many)	1.016304348	1.182389937
rnf145b	1.016304348	1.669291339
zgc:175088	1.016282225	1.402183803
med23	1.016260163	0.622641509
mapk8ip1	1.016260163	0.6375
mapk8ip1	1.016260163	0.6375
rspry1	1.016260163	0.803738318
vti1b	1.016260163	1.051724138
si:ch211-81a5.8	1.016216216	0.732217573
rmdn3	1.016216216	0.98974359
acadl	1.016194332	1.085054678
prr18	1.016129032	0.642140468
rptor	1.016129032	0.843137255
aldh1l2	1.016129032	1.078651685
idi1	1.016129032	1.087145969
pycrl	1.016129032	1.119402985
mapkapk3	1.016129032	1.269230769
zgc:91849	1.016129032	2.076923077
aff4	1.016129032	0.670886076
si:ch1073-291c23.2	1.016129032	1.023696682
xab2	1.016129032	1.04494382
ppp1r14ba	1.01594533	0.849056604
eif3f	1.015876565	0.995144725
prdm2b	1.015873016	0.778947368

tmc6b	1.015873016	0.869230769
txnl4b	1.015873016	0.875
tmed1b	1.015873016	1
slc38a10	1.015873016	1.060606061
ggt1b	1.015873016	1.139534884
mysm1	1.015873016	1.509803922
si:ch73-367f21.4	1.015873016	1.8
meaf6	1.015734266	1.150976909
map4k3a	1.015625	0.882352941
pacsin1b	1.015625	0.93902439
dia1a	1.015625	1.05
znf1134_1	1.015625	1.325581395
tmem177	1.015625	1.409090909
zfr2	1.015544041	0.767567568
ndfip1	1.015517241	0.982107356
cldn19	1.015463918	0.62345679
ptprn2	1.015444015	0.700440529
preb	1.015384615	0.841025641
rrp15	1.015384615	1.439814815
caprin1a	1.01532567	0.821596244
prkab1a	1.01532567	1.119617225
hspa8	1.015275551	1.114485685
angel2	1.015267176	1.113043478
arpc4l	1.015209125	1.23500612
inhbaa	1.015151515	0.796875
smc3	1.015082956	0.697959184
sh3bp5a	1.015037594	0.817460317
b3gat2	1.015037594	0.903508772
si:ch73-71c20.5	1.015037594	1.821052632
diablob	1.015	1.407821229
arpp19a	1.014989293	1.09439528
uqcrc1	1.014935742	1.153042852
tfap2b_2	1.014925373	0.666666667
ssbp3a	1.014925373	0.894736842
hipk3a	1.014925373	0.917910448
unc5c	1.014925373	1.130434783
mon1bb	1.014925373	1.155172414
zgc:91909_1	1.014925373	1.222222222
adck2	1.014925373	1.224489796
mt-nd6	1.01490335	0.988952149
mrps2	1.014888337	0.880487805
znf827	1.014814815	0.616161616
ddhd2	1.014814815	1.31
tax1bp1b	1.014772727	1.208510638
klhl41b	1.014771049	0.920233463
unc5da	1.014705882	0.461538462
inpp4b	1.014705882	0.88
nfkbib	1.014705882	1.050847458
mrpl14	1.014705882	1.347252747
yap1	1.014583333	0.826180258
plgrkt	1.014563107	0.931034483
usp36	1.014545455	0.752988048
spna2	1.014505119	0.683544304
tulp4b	1.014492754	0.326086957
phf12b	1.014492754	0.816
ndst1a	1.014492754	0.894736842
orc5	1.014492754	1.123076923
BX571715.1	1.014492754	1.416666667
GTPBP2 (1 of many)	1.014423077	1.068181818
calm1a	1.014415942	1.223668058
dnajc18	1.014388489	0.829959514

fam133b	1.014367816	1.293680297
eef2l2	1.014306569	1.139382601
prox1b	1.014285714	0.622222222
zgc:113337	1.014285714	0.829545455
rab11ba	1.014256619	0.819105691
mtdhb	1.014251781	0.997361478
stag1b	1.014218009	0.700534759
fam84a	1.014150943	0.590909091
ilf2	1.014092141	0.789983845
CABZ01102528.1	1.014084507	0.888888889
insrb	1.014084507	0.945652174
crygn1	1.014084507	1.044776119
rbm10	1.014084507	1.068965517
papolg	1.014044944	0.940140845
sec24a	1.014005602	0.778089888
march5l	1.013986014	1.052980132
pitpnbl	1.013941698	0.97008547
znf622	1.013937282	0.982332155
aldoca	1.013888889	0.822857143
garem	1.013888889	0.920634921
ptpn4a	1.013888889	0.963963964
grhprb	1.013888889	1.078175896
fam160b2	1.013888889	1.256756757
cd109	1.013888889	1.689655172
mob1ba	1.013793103	1.831050228
rbm4.3	1.013731826	0.671489362
psmb7	1.01369863	1.044474394
slc35d1b	1.013636364	1.398148148
psmc2	1.013579049	1.242926829
hk1	1.013559322	0.640096618
dnaja2l	1.013554217	0.859491779
col2a1a	1.013544018	0.686936937
gltscr1	1.013513514	0.636363636
MAF1	1.013513514	0.87732342
MAF1	1.013513514	0.87732342
tmem130	1.013513514	1.12244898
arhgap29a_1	1.013513514	1.2
carhsp1	1.013468013	0.733333333
ash2l	1.013468013	0.750902527
hspb1	1.013392857	0.872964169
slc30a5	1.013333333	1.179347826
crsp7	1.013333333	0.953846154
gtf3c3	1.013333333	1.25862069
spg20b	1.013333333	1.333333333
mtfr1	1.013333333	1.609375
cyt1l	1.013311148	0.709156669
ecrg4a	1.013303769	0.922242315
slc2a3a	1.013303769	0.923976608
boc	1.013192612	1.130136986
pfkpb	1.013157895	0.803921569
tm7sf3	1.013157895	1.067484663
smad9	1.013157895	1.068965517
vps39	1.013157895	1.153846154
zgc:194285	1.013157895	1.818181818
foxp1a	1.013157895	1.068965517
txn2	1.013157895	1.139059305
gramd1a	1.013071895	0.905982906
snrpc	1.013029316	0.739130435
cenpi	1.012987013	1
CABZ01087514.1	1.012987013	2.108695652
scrib	1.012931034	0.770334928

zgc:153521	1.012820513	0.753846154
limch1a	1.012820513	0.816666667
trim44	1.012820513	1
qrsl1	1.012820513	1.182926829
prpf40a	1.012738854	0.665441176
SPEN (1 of many)	1.012738854	0.775862069
rnf165a	1.012658228	1.076923077
tipin	1.012658228	1.442857143
ifi30	1.012531328	0.911816578
gpc4	1.012526096	0.970454545
nid2a	1.0125	0.910852713
atf7a	1.0125	1.093333333
pop5	1.01242236	1.5546875
vbp1	1.012376238	1.079069767
ift172	1.012345679	0.753623188
tubgcp5	1.012345679	1.016393443
fstb	1.012345679	1.572519084
ndel1a	1.012307692	1.365187713
pcnp	1.012254902	1.444141689
rspo2	1.012195122	1.098360656
dnajc28	1.012195122	1.296296296
rpain	1.012195122	1.378787879
cryba1l1	1.012163892	0.520415473
dusp8a	1.012158055	0.97309417
naa35	1.012158055	1.003546099
gng2	1.012145749	0.689213894
zyx	1.012145749	0.957142857
rabep1	1.012121212	1.026548673
sfxn4	1.012121212	1.147435897
CAMSAP3 (1 of many)_2	1.012048193	0.486486486
ndst2a_2	1.012048193	0.892156863
fgf13b	1.012048193	0.923529412
daam1a	1.012048193	1.042253521
blcap	1.011990408	1.051359517
bsdc1	1.011976048	1.044117647
GLDC	1.011976048	0.717821782
adgrl2b.1	1.011904762	0.585714286
smug1	1.011904762	1.096774194
gtpbp1l	1.011764706	0.950617284
foxl1	1.011764706	1.382352941
cacna1sb	1.01171875	1.094170404
wtap	1.01171875	1.25
nfs1	1.01171875	1.639810427
si:ch211-255p10.3	1.011673152	1.26590106
b3galt2	1.011627907	0.747440273
chst2a	1.011583012	0.960227273
snrnp70	1.011577424	0.795373665
maff	1.011547344	1.729452055
impad1	1.011494253	0.892523364
tanc2a	1.011363636	0.623188406
si:cabz01007794.1	1.011299435	1.078350515
gsk3b	1.011267606	0.652173913
abi1a	1.011235955	0.807228916
aar2	1.011235955	1.0625
mrpl51	1.011235955	1.302884615
hmgcrb	1.011173184	1.057324841
gc	1.011160714	0.780141844
pdcl	1.011111111	0.932748538
vdrb	1.011111111	1.080246914
stard13b	1.011111111	1.083333333
mnat1	1.011111111	1.439393939

gnb2	1.010989011	0.657738095
slc25a36a	1.010928962	1.012820513
herc1	1.010869565	0.796875
rassf10a	1.010869565	0.965909091
BX470189.1	1.010869565	1.462686567
basp1	1.010770059	0.377622378
ccnk	1.010638298	0.613636364
papss1	1.010638298	1.107692308
cdk5	1.010638298	1.703703704
atp6v1aa	1.010598032	0.854870775
nup214	1.010526316	0.648351648
jph1b	1.010526316	0.854942234
si:ch211-278j3.3	1.010526316	0.873563218
eif2ak1	1.010526316	1.385542169
si:ch73-193c12.2	1.010526316	1.5
rnaseh2b	1.010471204	1.565217391
unc45a	1.010416667	0.686046512
arhgap35a	1.010416667	0.707142857
lemd3	1.010416667	0.78313253
usp33	1.010380623	0.948051948
mxi1	1.010344828	0.696581197
oxa1l	1.010282776	0.975903614
tmx2b	1.010273973	1.105485232
hs6st1a	1.010204082	0.62962963
znf865	1.010204082	0.657894737
si:ch211-274j7.3	1.010152284	0.322033898
ubn2b	1.01010101	0.683544304
IDH2	1.009969325	1.014348981
suv420h1	1.009966777	0.87398374
apex1	1.009925558	0.843023256
gpsm2	1.00990099	0.825581395
klhl15	1.00990099	0.846153846
pithd1	1.009803922	0.760330579
kptn	1.009803922	1.361344538
ZNF319 (1 of many)	1.009615385	1.079710145
pvalb1	1.009609506	1.044362999
filip1l	1.009569378	0.816753927
cited4a	1.009557945	1.576757532
rps6	1.009484333	0.908150852
spcs2	1.009417808	1.388185654
ggcx	1.009389671	1.273333333
dyrk1b	1.009375	0.671641791
ndufaf1	1.009345794	1.008
fez1	1.009326425	0.672897196
nptnb	1.00931677	0.592307692
actn3a	1.009294147	1.120087072
atp2a2a	1.00921659	1.102040816
hnrnpaba	1.009146341	0.521432173
mtpn	1.009081736	1.06124498
mrps23	1.009049774	1.303501946
PCP4L1 (1 of many)	1.009039548	0.584397163
nfya	1.008955224	0.93125
scamp5a	1.008948546	0.862745098
mettl9	1.008928571	0.944444444
rab12	1.008928571	1.197802198
baz2ba	1.008888889	0.541237113
gtf2h3	1.008849558	1.543478261
copz1	1.008830022	1.191827469
usp12a	1.00877193	0.618811881
hyi	1.00877193	0.861751152
chst10	1.00877193	1.65

phf3	1.008695652	0.741935484
trim71	1.008695652	0.880434783
triobpb	1.008695652	1.176
itga8	1.008695652	1.209302326
dusp22a	1.008695652	1.745283019
gngt2b	1.008645533	0.556652387
ints3	1.00862069	0.882978723
cwc25	1.00862069	1.025862069
lmo4b	1.008591065	1.029906542
hug	1.008561644	1.170081967
cacna1aa	1.008547009	0.337349398
kat7b	1.008547009	0.977272727
edem2	1.008474576	0.942446043
foxg1b	1.008460237	0.884615385
ctdsp2	1.008426966	1.399364407
llgl1	1.008403361	0.756097561
ppcs	1.008403361	1.094488189
gale	1.008403361	1.16097561
ppp2r3a	1.008368201	1.367149758
grk5l	1.008333333	1.036585366
luc7l3	1.008316008	0.807860262
cry5	1.008264463	1.765734266
ncl1	1.008219178	0.812297735
lix1l	1.008196721	1.069306931
phlda1	1.008196721	1.231707317
lyrm5a	1.008196721	2.309859155
ube2l3a	1.008143322	1.143911439
si:ch211-113e8.10	1.008130081	0.720720721
ing1	1.008130081	1.666666667
cbx1a	1.008012821	1.138317757
klc4	1.008	0.74
zgc:112285	1.007905138	1.104651163
dlg5b.1	1.007874016	0.922413793
crlf3	1.007874016	1.026143791
crygm2d13	1.007873414	1.057867083
vdac3	1.007865414	1.059589495
dnajc6	1.0078125	0.435294118
bud31	1.007751938	1.078947368
dnajb6a	1.00764526	1.271062271
PARL (1 of many)	1.007633588	0.828571429
reep2	1.007604563	1.404040404
gyg1b	1.007580979	1.156515581
tex261	1.007575758	1.46728972
mtdha	1.007541478	0.791814947
col1a1a	1.007478133	1.023668205
rasa1a	1.007462687	0.869565217
capza1b	1.007443245	0.996197719
rsrp1	1.007352941	1.374015748
birc6	1.00729927	0.716814159
eif2s1b	1.007285974	1.042950985
ndufs7	1.007258515	1.09077527
cpm	1.007246377	1.212121212
kdm2ab	1.007194245	0.681415929
si:ch211-212c13.8	1.007194245	1.048117155
marcksa	1.007092199	0.788
ndufab1b	1.007067138	1.035522067
schip1	1.007042254	0.85
cdh6	1.006993007	0.772727273
fbxw4	1.006993007	1.0234375
ptbp2a	1.006944444	0.962962963
smap1	1.006932409	0.784067086

mybpc1	1.006920415	1.046610169
myoz1a	1.006920415	1.206197855
rap2b	1.006756757	0.856164384
mrpl45	1.006756757	1.261613692
mrpl46	1.006711409	1.146666667
rpl10	1.006679294	0.997718698
shmt2	1.00660793	0.784552846
got1	1.006587615	1.374847375
si:dkey-46a10.3	1.006493506	1.663793103
cyfip2	1.006493506	0.485530547
angptl4	1.006443299	1.601340034
si:dkey-7j14.6	1.006369427	1.018413598
pnp5a	1.006361323	1.7618683
sf3b1	1.006334842	0.910493827
nlk2	1.006329114	0.609090909
fbxo45	1.006329114	0.927007299
jak2b	1.006329114	0.943262411
tusc2a	1.00625	0.988304094
rbb4l	1.00625	1.005813953
zgc:193593_1	1.006237006	1.4106814
mafba	1.00617284	0.568627451
vmp1	1.006116208	1.119444444
tsfm	1.006097561	1.243243243
srp68	1.006072874	1.126794258
vtna	1.006024096	0.980769231
dact1	1.005988024	0.986754967
adgrb3	1.005952381	0.383928571
NDUFC1	1.005930319	1.183157895
atp6v1f	1.005928854	1.223753977
creb1b	1.00591716	0.846153846
ubl7b	1.00591716	1.020725389
hccsa.1	1.00591716	1.307086614
si:ch211-253h3.1	1.005882353	0.725925926
si:ch211-166e11.5	1.005882353	1.016129032
nipal3	1.005882353	1.642335766
zfhx4	1.005847953	0.478632479
camk2n1a	1.005763689	1.317554241
rps26l	1.005673481	0.834615385
nup153	1.005649718	0.652777778
tmem106ba	1.005639098	1.5
eif3ba	1.005565863	0.845416187
rbm26	1.005555556	0.543046358
sox6	1.005555556	0.905109489
srprb	1.005494505	1.029929577
atp1b2a	1.005494505	0.63913471
slmapa	1.005464481	0.905325444
psmb1	1.005451242	1.084394904
ccng1	1.005421184	1.134903213
vsg1	1.005395683	1.383592018
mogs	1.005376344	0.949275362
ncbp3	1.005376344	1.292857143
cdon	1.005263158	0.942465753
epha4a	1.005235602	0.6
trappc13	1.005128205	1.191358025
serbp1b	1.005116959	0.859235151
ckbb	1.005113413	0.547429399
abhd10b	1.005025126	0.90952381
vps41	1.004950495	1.251612903
gps2	1.004807692	1.272108844
dnajc9	1.004807692	1.493902439
smndc1	1.004790419	1.322623829

cirh1a	1.004672897	1.048309179
kifc1	1.004651163	0.82
pgam5	1.00462963	0.892156863
PEPD	1.004608295	0.802631579
ube2a	1.004552352	1.398972603
kpnb3	1.004470939	0.743315508
ppm1da	1.004464286	0.813131313
psen2	1.004424779	0.817307692
ncam2	1.004405286	0.527472527
myhz2	1.004365859	1.141215715
sypb	1.004359673	0.694035631
slc27a1a	1.004347826	1.069444444
ufsp2	1.004347826	1.46961326
cachd1	1.004329004	0.669950739
npnt	1.004329004	0.820652174
eif1axb	1.004329004	1.275045537
timm8a	1.004291845	1.523584906
pkma	1.004240555	0.693528184
zgc:112056	1.004201681	1.227906977
TPM1	1.004166667	1.030044843
zgc:123103	1.004057971	1.216915265
klhl21	1.004	1.16751269
pde6d	1.004	1.334975369
rbfox2	1.003976143	0.720403023
chrac1	1.003937008	1.188940092
epb41a	1.003875969	0.427807487
tardbp	1.003809524	0.764488286
picalmb	1.003787879	0.685990338
srsf10b	1.003787879	0.711815562
tmem184ba	1.003773585	0.957894737
gngt1	1.003740648	0.799176955
cdk5r2a	1.003710575	0.279317697
me3	1.003708282	1.114671164
mgst3a	1.003616637	1.048120301
usp5	1.003603604	0.890625
RPS17	1.003589283	0.860230874
pdzd11	1.003484321	1.3125
tmsb	1.003472222	0.952095808
chuk	1.003424658	1.711790393
pno1	1.003404255	1.159813809
stam2	1.003401361	0.94488189
got2a	1.003355705	1.344339623
ivns1abpb	1.003289474	1.171532847
ctnnb1	1.003225806	0.666502463
gria2a	1.003184713	0.904761905
rtn1a	1.003122212	0.762291169
ubr7	1.003012048	0.951140065
crygm2d18	1.002969877	0.896820186
crygm2d16	1.002952927	0.959777632
eif4e3	1.002949853	1.452631579
eef1g	1.00283209	0.955438476
cycsb	1.002779064	1.468912416
kpna5	1.002762431	0.966777409
cpne3	1.002724796	1.475609756
cltca	1.002717391	0.653169014
ETFA	1.002710027	1.231613611
psat1	1.002680965	1.013888889
xpnpep1	1.002469136	0.921875
hm13	1.002427184	0.725623583
tmem165	1.002386635	1.061994609
hagh	1.00234192	2.125

ptn	1.002298851	0.636075949
psma8	1.002188184	1.09249859
rlbp1a	1.002178649	0.914285714
tspan31	1.002159827	1.11627907
pin1	1.002061856	0.943866944
mybpc3	1.001897533	1.156126482
dct	1.001862197	1.118644068
cops2	1.001694915	1.374485597
ldb3a	1.001675042	0.964227642
arhgdia	1.001537279	0.807660283
selt2	1.001351351	1.344165436
tmod4	1.001279318	1.06174886
srsf5b	1.001248439	1.004189944
krtt1c19e	1.001213945	0.931056428
sh3bgr	1.001146789	1.182539683
etfb	1.000843882	1.119288314
mdh2	1.000746826	1.173398731
ppp2r1b	1.00063012	1.077857143
anxa2a	1.00062422	1.455653351
ywhabb	1.000506586	0.960762943
kcnd3	1	0.276595745
FQ312026.2	1	0.314285714
ptprnb	1	0.349206349
slc1a6	1	0.354166667
si:ch211-87j1.4	1	0.384615385
CIT	1	0.393442623
whsc1	1	0.401869159
CABZ01075131.1	1	0.411764706
cacna1g	1	0.433333333
BICD1 (1 of many)	1	0.444444444
cadpsa	1	0.448275862
arl6ip6	1	0.476190476
clcn4	1	0.47826087
epha8	1	0.484848485
si:dkey-253d23.1	1	0.5
znf710b	1	0.516556291
pvrl1b	1	0.519553073
grm5a	1	0.526315789
CABZ01038499.1	1	0.527272727
zgc:153395	1	0.527777778
usp53b_1	1	0.534883721
gabbr2	1	0.541666667
si:dkey-15k2.5	1	0.55
dus1l	1	0.551020408
pde3a	1	0.553571429
CT583723.1	1	0.555555556
samsn1b	1	0.56097561
tmem63c	1	0.566666667
si:dkey-106g10.7	1	0.571428571
sin3b	1	0.577319588
angel1	1	0.578947368
sdk2b	1	0.578947368
rfx3	1	0.578947368
fmnl1a	1	0.580645161
tmem125b	1	0.583333333
dchs1b	1	0.583333333
mustn1a	1	0.58974359
sema5a	1	0.593333333
tbc1d10b	1	0.595041322
AC024175.23	1	0.6
csnk1g1	1	0.606557377

tek	1	0.607142857
tulp4a	1	0.612612613
acd	1	0.612903226
chst7	1	0.613636364
impdh1a	1	0.629032258
camk1ga	1	0.631578947
CABZ01112317.1	1	0.633333333
cpsf2	1	0.641025641
FAM13C	1	0.641509434
casz1	1	0.641975309
grna	1	0.642857143
pkn1b	1	0.652173913
uhrf1bp1l	1	0.653846154
rarab	1	0.656934307
si:ch211-222f23.7	1	0.666666667
si:cabz01076231.1	1	0.666666667
si:dkey-29p9.3	1	0.666666667
arvcfb	1	0.666666667
mertka	1	0.675
fbrs	1	0.679389313
si:ch73-344o19.1	1	0.68
si:ch211-173b16.3	1	0.68
tmem100b	1	0.685714286
tbck	1	0.686046512
fstl5	1	0.68618267
haus5	1	0.68627451
skor1a	1	0.689655172
gli1	1	0.697674419
pbx3a	1	0.7
cdca5	1	0.701298701
dlg5a	1	0.705882353
tmppe	1	0.706896552
BRPF1	1	0.711864407
e2f4	1	0.713114754
ppp1r9ba	1	0.713114754
FAM124B	1	0.714285714
kmt2bb	1	0.721518987
trim2b	1	0.722891566
n4bp1	1	0.731343284
cwc22	1	0.739130435
slc17a8	1	0.740740741
clasp1a	1	0.742268041
sp3a	1	0.742857143
cep72	1	0.75
slc5a12	1	0.76
CABZ01055522.1	1	0.766666667
tm7sf2	1	0.767123288
si:ch1073-235m4.3	1	0.772727273
dpyda.3	1	0.772727273
nfrkb	1	0.772727273
cables2a	1	0.775510204
FO818704.1	1	0.777777778
b2m	1	0.777777778
msx1a	1	0.78125
anapc1	1	0.783783784
asb16	1	0.785714286
si:ch211-208h1.1	1	0.785714286
si:dkey-54f18.2	1	0.785714286
tmtopsb	1	0.785714286
pthlha	1	0.787234043
ucn3l	1	0.787878788

si:dkey-166k12.1	1	0.787878788
rnasen	1	0.79245283
map2k6	1	0.794117647
tcf21	1	0.796296296
adat1	1	0.8
dre-mir-132-2	1	0.8
si:ch211-214g1.4	1	0.8
zgc:162339	1	0.806451613
hnrnpk	1	0.806788512
kifc3	1	0.807017544
epob	1	0.80952381
vwa7	1	0.814814815
GAS2	1	0.815789474
rabl6b	1	0.816568047
neurl4	1	0.818181818
CABZ01065679.1	1	0.823529412
FO082031.1	1	0.823529412
unm_sa1614	1	0.826086957
dhx32a	1	0.827586207
hnrnph1	1	0.827586207
zgc:77287	1	0.829268293
asb3	1	0.829787234
kremen1	1	0.833333333
si:dkey-164n2.1	1	0.833333333
ascc3	1	0.833333333
plekhm3	1	0.837209302
kmt2ca	1	0.837209302
zgc:158412	1	0.84
si:ch211-163l21.7	1	0.842105263
si:ch1073-416d2.4	1	0.848484848
c2cd5	1	0.849056604
si:ch211-139d20.3	1	0.857142857
has2	1	0.857142857
tbce	1	0.861111111
plp1b	1	0.861702128
tcirg1b	1	0.863309353
snphb	1	0.864864865
pnp5b	1	0.865853659
dtnba	1	0.867716535
sox1a	1	0.870748299
PGS1	1	0.872727273
agtpbp1_1	1	0.873563218
rpz3	1	0.875
pik3r4	1	0.877358491
tyro3	1	0.88
fkbp16	1	0.886524823
vps13a	1	0.888
slc4a1ap	1	0.893333333
FO834898.1	1	0.894736842
smpd3	1	0.894736842
klhl12	1	0.896551724
pagr1	1	0.897727273
si:dkeyp-73d8.13	1	0.9
gnrh2	1	0.903225806
si:dkey-22h13.5	1	0.909090909
lats1	1	0.913043478
tnpo1	1	0.9140625
etnk2	1	0.916666667
mmd2b	1	0.923076923
si:ch211-202h22.8	1	0.928571429
morn2	1	0.928571429

WIPI1 (1 of many)	1	0.928571429
CR352258.1	1	0.9375
atxn7l1	1	0.9375
sstr1a	1	0.939393939
pnpla7a	1	0.939849624
mylk2	1	0.941176471
asb13a.1	1	0.943396226
CU571310.1	1	0.944444444
rassf3	1	0.946428571
csgalnact2	1	0.948275862
ttc21b	1	0.95
homezb	1	0.950413223
STXBP6	1	0.953974895
slc25a38b	1	0.95890411
klc1a	1	0.95951417
HPS6	1	0.96
CAAP1	1	0.961373391
CABZ01064859.1	1	0.962962963
hnf1a	1	0.962962963
GXYLT1 (1 of many)	1	0.964285714
sufu	1	0.964497041
kif7	1	0.96875
slc25a32b	1	0.972727273
msto1	1	0.97979798
stim1a	1	0.984848485
dync1li1	1	0.988593156
st3gal3a	1	0.99047619
si:ch73-233k15.2	1	1
si:ch211-68a17.5	1	1
znf994	1	1
si:dkey-26c10.5	1	1
hps5	1	1
ches1	1	1
snrpf	1	1.003616637
atrxl	1	1.01
fam120a	1	1.010526316
si:ch211-183d21.1	1	1.011904762
rfx1b	1	1.01754386
nxnl2	1	1.020618557
ggnbp2	1	1.026086957
PLCL2	1	1.027777778
cyb5r1	1	1.0330033
cpvl	1	1.033707865
ncapg	1	1.034013605
tmco3	1	1.038461538
lmnl3	1	1.04
nck1b	1	1.040462428
DUSP21	1	1.048780488
dffa	1	1.052173913
arhgef6	1	1.052631579
med1	1	1.053333333
tbccd1	1	1.057142857
sav1	1	1.057522124
uvrag	1	1.0625
arpp21	1	1.063829787
ahsa1b	1	1.066433566
wasa	1	1.066666667
bmp8a	1	1.066666667
megf6a	1	1.066666667
gsto1	1	1.06779661
stambpb	1	1.068965517

txlna	1	1.068965517
slc4a4b	1	1.074074074
lin52	1	1.075471698
ccdc88b	1	1.076923077
rangrf	1	1.092105263
strn4	1	1.098684211
aldh5a1	1	1.100917431
si:ch1073-303d10.1	1	1.107142857
ddhd1a	1	1.108695652
napepld	1	1.112903226
raf1a_1	1	1.12
fmn2a	1	1.130434783
ipcef1	1	1.131578947
cep162	1	1.133333333
PKD1	1	1.133333333
trap1	1	1.136200717
serpinb14	1	1.139455782
FO834877.1	1	1.142857143
gsg1l2a	1	1.142857143
fbxo34	1	1.147335423
chrnb3b	1	1.15
sertad2b	1	1.150537634
AKT1	1	1.152173913
cyp27a1.4	1	1.153846154
mrps36	1	1.154146341
spast	1	1.157894737
si:ch73-112l6.1	1	1.173913043
NNMT	1	1.175
dock1	1	1.175
im:6911600	1	1.180360721
si:ch211-262i1.2	1	1.181818182
xrcc6bp1	1	1.186206897
zgc:174574	1	1.1875
tctex1d1	1	1.1875
nit1	1	1.1875
tram2	1	1.1875
atg4a	1	1.192307692
adora2ab	1	1.2
si:ch211-271d10.2	1	1.2
si:dkey-31f5.8	1	1.2
pank1a	1	1.2
si:dkey-33c12.4	1	1.202185792
arsk	1	1.208333333
traf3	1	1.212765957
itchb	1	1.225
setx	1	1.225806452
alg1	1	1.225806452
acss2l	1	1.227272727
romo1	1	1.228013029
si:ch211-276c2.4	1	1.229166667
tspan3b	1	1.230769231
RB1CC1	1	1.23125
dcun1d4	1	1.231578947
fam13b	1	1.234090909
tmem129	1	1.238095238
znf148	1	1.24
galk2	1	1.24137931
rhobtb2b	1	1.24137931
panx3	1	1.243243243
GUCA1A (1 of many)	1	1.25
nudt5	1	1.259574468

si:dkey-20i20.4	1	1.266666667
prdm11	1	1.272727273
mlh1	1	1.282608696
tox4b	1	1.290322581
si:dkey-76k16.6	1	1.294117647
si:ch211-73m21.3	1	1.3
si:dkeyp-2e4.8	1	1.3
fibpb	1	1.3125
trim62	1	1.314285714
zgc:163061_2	1	1.315789474
zhx2a	1	1.31884058
cenpo	1	1.333333333
adck1	1	1.333333333
ccne1	1	1.333333333
stx5al	1	1.335443038
tmem238b	1	1.342857143
ZFYVE1	1	1.34375
BX908782.3	1	1.355555556
lrrc29	1	1.355555556
grb10b	1	1.357142857
si:ch211-215p11.1	1	1.363636364
si:dkey-207m2.4_2	1	1.363636364
flr	1	1.365384615
pir	1	1.38028169
si:dkeyp-1a11.3	1	1.391304348
pex19	1	1.398190045
si:dkeyp-97a10.3	1	1.4
si:ch211-117l17.6	1	1.40625
ASTE1	1	1.40625
tmprss5	1	1.416666667
si:ch73-105b23.1	1	1.416666667
si:dkey-239h2.3	1	1.421875
pex10	1	1.433333333
znf1181	1	1.434782609
si:ch211-198c1.10	1	1.4375
rundc3aa	1	1.448275862
olah	1	1.45
ypel3	1	1.450617284
ftr01	1	1.454545455
si:cabz01073308.1	1	1.454545455
pot1	1	1.454545455
stard3	1	1.458333333
txndc9	1	1.459510358
zgc:113336	1	1.466666667
nsun4	1	1.466666667
armc10	1	1.472727273
si:dkey-171o17.8	1	1.476190476
cyp2k18	1	1.484848485
si:ch73-144d13.4	1	1.486486486
si:ch211-51h9.6	1	1.488721805
rassf6	1	1.494949495
wdr76	1	1.495934959
si:dkey-78a14.4	1	1.5
cdkn2c	1	1.5
si:dkey-172k15.3	1	1.5
eva1bb	1	1.5
si:dkey-93n13.1	1	1.5
b9d2	1	1.5
thrap3a	1	1.507692308
si:ch211-163b2.4	1	1.511111111
crema	1	1.554545455

ninl_1	1	1.56
si:dkey-222b8.4	1	1.5625
si:ch211-199b20.3	1	1.611111111
BX004873.5	1	1.625
AL929119.1	1	1.636363636
lypla1	1	1.638888889
enkur	1	1.64
slc28a1	1	1.641509434
zgc:171599	1	1.645833333
si:dkey-121a9.3	1	1.65
slc2a11b	1	1.650485437
znf1113	1	1.666666667
sgtb	1	1.671875
chmp3	1	1.68852459
nsmaf	1	1.698113208
znf1020	1	1.7
si:ch211-213a13.1	1	1.7
si:ch73-78o10.1	1	1.705882353
zgc:153722	1	1.727272727
ccdc90b	1	1.730769231
ggact.1	1	1.761904762
zgc:171220	1	1.764705882
slf1	1	1.769230769
tceanc2	1	1.793103448
abcg4b	1	1.8
si:dkey-78p8.1	1	1.80952381
si:dkey-274m17.3	1	1.818181818
orai1a	1	1.833333333
si:dkey-77f5.8	1	1.833333333
si:dkey-38l22.2	1	1.846153846
si:ch211-274p24.2	1	1.85
pcgf1	1	1.857142857
waif2	1	1.857142857
si:ch211-209l18.4	1	1.9
si:dkey-262g12.9	1	1.947368421
zgc:198241	1	2
NAT6	1	2
tmem175	1	2
ppa2	1	2.065217391
chac2	1	2.153846154
si:dkey-163m14.2	1	2.176470588
si:dkey-23k10.3	1	2.333333333
si:dkey-111k8.3	1	2.333333333
lrrc42	1	2.346153846
si:ch73-124g13.5	1	2.5
si:dkey-106l3.7	1	2.571428571
zgc:110353	1	2.6
si:ch211-243c14.5	1	2.666666667
ptgis	1	2.775
si:dkey-186j8.2	1	3.272727273
SNORA26_2	1	4
cldni	0.999666778	1.436639605
sdhb	0.99930265	1.055782313
rpl6	0.999232428	0.966653174
pfkma	0.999082569	1.53042328
aldh2.2	0.998846597	0.895061728
hnrnph1l	0.998786408	0.788352273
mdh1aa	0.998734498	0.880070994
ivd	0.998559078	1.518211921
si:dkeyp-44a8.2	0.998527246	1.005681818
slc6a1b	0.998482549	0.227368421

COX5B (1 of many)_2	0.998415805	0.931830192
sdc4	0.998367347	1.492522433
slc25a3b	0.998321866	0.810337145
psmd4b	0.998091603	1.384449244
pgm1	0.998073218	0.877170418
paip2b	0.998029557	0.903533906
acadm	0.998025016	1.303867403
mt-atp8	0.997984862	1.266072709
trappc1	0.997975709	0.921875
rbpms2a	0.997975709	1.100628931
rps19	0.997778248	0.930730142
ggctb	0.997737557	1.013592233
vdac1	0.997703261	0.85480859
3-Sep	0.9975	0.985380117
prdx2	0.997324099	0.950737054
rab10	0.997273347	1.06764961
cacnb4b	0.997118156	0.603305785
dsg2.1	0.99704142	1.018115942
arl1	0.996875	1.071304348
eif3c	0.996818182	0.804070043
pam16	0.996815287	1.039772727
phb2a	0.996784566	0.97995283
pcp4b	0.996740016	0.967741935
zgc:92313	0.996721311	1.238970588
myca	0.996677741	0.871473354
hgs	0.996655518	0.821011673
si:dkey-106l3.6	0.996598639	0.535885167
stom	0.996559633	1.34097035
zgc:66101	0.996527778	0.655063291
xrn2	0.996466431	0.744274809
atp6v0a1b	0.996453901	0.742366412
slc22a18	0.996415771	1.230434783
fam3a	0.996389892	1.294354839
ndufs6	0.996186845	1.235177866
tcf12	0.996183206	0.853211009
pfn1	0.996162224	0.839160839
rcor1	0.996138996	1.211055276
slc11a2	0.996078431	0.813114754
tp53bp2a	0.996078431	0.911290323
hpdb	0.996068152	1.608414239
gcdha	0.995978552	1.183448276
dr1	0.995951417	0.868421053
agrn	0.995934959	0.74251497
ctdspl2a	0.995934959	0.765
trappc6b	0.995918367	1.570621469
trpc4apa	0.995901639	0.838383838
acvr2aa	0.995884774	0.717105263
zgc:153426	0.995879121	0.409090909
rnf25	0.995867769	0.986607143
tm9sf2	0.995793901	1.088235294
atf7ip	0.995762712	0.623152709
gngt2a	0.995732575	0.715827338
pdlim5b	0.995726496	0.904942966
si:ch211-235o23.1	0.995708155	1.266990291
rraga	0.995652174	1.402116402
psmd11a	0.995555556	0.927083333
r3hdm4	0.995535714	0.948186528
rnft1	0.995454545	1.104477612
ctsf	0.995363215	1.026188167
HPF1	0.995327103	1.198830409
dcun1d2a	0.995249406	1.129175947

crygn2	0.995201456	1.053891387
tbl1xr1a	0.995098039	0.707006369
mkl1b	0.995	0.807486631
atf4a	0.994997726	1.006052142
apeh	0.994974874	1.063157895
snw1	0.994940978	0.783636364
ufc1	0.994923858	1.741721854
zc3h7b	0.99488491	0.953177258
usp14	0.994858612	1.160427807
magt1	0.994858612	1.509493671
rusc1	0.994845361	0.341666667
amd1	0.994834711	1.114772727
tcp1	0.994676131	0.721832439
rps10	0.994665749	0.942893762
tmem132e	0.994594595	0.606666667
ehmt1b	0.994565217	0.608695652
nrarpa	0.994520548	0.88
ppp1r9bb	0.994505495	0.630630631
fgfr1a	0.994505495	0.822485207
CABZ01102047.1	0.994475138	0.465277778
tial1	0.994444444	1.51
atp5g3b	0.994230769	0.971630709
eif3d	0.994199536	0.861589772
dpp6b	0.994117647	0.64516129
angptl3	0.994117647	1.96031746
zgc:110843	0.994100295	1.095890411
atp5g1	0.994088513	1.127372097
kmt2e	0.99408284	0.408805031
ccdc25	0.99408284	1.443298969
nt5c2a	0.994029851	0.977011494
si:ch211-255g12.6	0.993965651	0.969796215
mthfd1a	0.993957704	0.818604651
fam63a	0.993939394	0.911392405
fuca2	0.993874426	0.871010638
nell2a	0.99378882	0.290076336
ttc19	0.99378882	1.582608696
hmox2a	0.99376947	1.305263158
faua	0.993760937	0.91563377
zgc:91999	0.993730408	0.924281984
lysmd2	0.993730408	0.956228956
ptch2	0.993710692	0.536764706
cbx6a	0.993710692	0.945454545
tpd52l2a	0.993690852	1.252559727
arhgap1	0.993660856	1.217472119
ddx21	0.993630573	0.841172461
F8A2	0.993589744	1.09352518
slc26a5	0.993589744	1.359375
gstt1b	0.993548387	1.25862069
mmaa	0.993506494	0.884057971
acy1	0.993506494	1.160583942
med27	0.993421053	1.162962963
atp1b3b	0.993411765	0.930531232
ppil4	0.993377483	1.178571429
fam171a2	0.993288591	0.785714286
fam135a	0.993288591	0.798165138
napga	0.993243243	1.090909091
trnt1	0.993243243	1.441441441
CABZ01114898.1	0.993197279	0.887850467
dync1i2a	0.993197279	1.036312849
xpot	0.993174061	0.788888889
ptp4a1	0.993154947	1.273264402

itpk1b	0.993150685	0.852713178
tmem251	0.993103448	1.532374101
fryl	0.993055556	0.739495798
tat	0.992982456	2.365384615
abcb7	0.992957746	1.147859922
eif4g2b	0.992926614	0.822906641
pygl	0.992916175	0.75174216
si:ch73-265d7.2	0.992907801	0.489583333
kalrnb	0.992907801	0.680851064
pspc1	0.992831541	1.385714286
znf131	0.992805755	0.909090909
tuba1a	0.992795677	0.565172591
aass	0.992753623	0.932835821
lypla2	0.992753623	0.956896552
TRAPPC8	0.992592593	0.718181818
NOP14	0.992366412	0.991803279
nrip1b	0.992248062	0.64516129
ubald1a	0.992248062	0.914691943
pnpla7b	0.992248062	0.918032787
mtm1	0.992248062	1.111111111
setd3	0.992217899	1.041860465
clk2a	0.992207792	1.34751773
efnb1	0.9921875	0.712765957
AC024175.21	0.9921875	0.799212598
tubgcp4	0.9921875	1.009708738
tmem79b	0.9921875	1.419753086
siah2l	0.992125984	0.833333333
nedd1	0.992063492	1.011904762
galnt10	0.992063492	1.048543689
ptprfa	0.992	0.585585586
uck2b	0.992	0.922764228
agr1	0.991916859	1.439675174
phf24	0.991869919	0.623931624
hnrnpm	0.991831972	0.670299728
rpl28	0.991733639	0.943010252
strap	0.991614256	1.195544554
psmb6	0.991578947	1.077630235
si:ch211-269e2.1	0.991452991	1.242424242
atp6v1c1b	0.991452991	1.254854369
zbtb16a	0.99137931	0.622222222
smurf1	0.99137931	0.641304348
plod1a	0.99137931	0.965346535
zgc:91940	0.99137931	1.376344086
tefb	0.991354467	0.966122961
sema3fb	0.991304348	0.765957447
asf1ba	0.991304348	0.828828829
golga2	0.991304348	1.313953488
ptp4a2a	0.991258741	1.358649789
adrbk2	0.99122807	0.557894737
ogt.2	0.991150442	1.101265823
p4ha2	0.991071429	0.765765766
polb	0.991071429	1.354166667
trmt112	0.991071429	1.497461929
rassf8b	0.991071429	1.748427673
hic1l	0.991071429	1.848684211
ift20	0.991071429	1.911392405
smu1a	0.990963855	1.103571429
mapk8a	0.990909091	0.548076923
kdm7ab	0.990909091	0.65
si:ch211-283h6.4	0.990909091	0.842696629
murca	0.990899899	0.967472119

dyrk1ab	0.990825688	0.586206897
hs6st1b	0.990825688	0.745283019
parp6b	0.990825688	1.146666667
reep3b	0.990825688	1.47382199
gsc	0.990740741	1.494505495
rbx1	0.990727532	1.050916497
slc35g2b	0.990654206	0.656565657
onecutl	0.990654206	0.662921348
CABZ01037298.1	0.990654206	0.795031056
cacna2d2b	0.990654206	1.089285714
GMEB2	0.990566038	0.972972973
cct7	0.990541052	1.014992504
abcg2c	0.99047619	0.721153846
sap130a	0.990384615	0.698795181
actl6a	0.990384615	1.193548387
rbbp6	0.990338164	0.918604651
metap1	0.990338164	0.970501475
si:ch211-133n4.4	0.990300679	1.487113402
AC024175.22	0.990291262	3.197530864
si:ch211-260e23.9	0.990243902	2.016548463
CABZ01085923.1	0.990147783	1.22826087
si:ch211-252f13.6	0.99009901	0.321428571
uggt2	0.99	0.974025974
nudt8	0.99	1.110091743
AC024175.20	0.989974937	1.434343434
cstf2	0.989959839	0.926289926
ndufv2	0.989954338	1.059104737
fkbp14	0.989949749	1.37012987
trip12	0.989932886	0.702811245
sco2	0.98989899	1.008333333
scarb1	0.98989899	1.086538462
cbln18	0.98989899	1.93814433
uroc1	0.989873418	1.055674518
si:ch211-215a10.4	0.989847716	0.94011976
crygmxl2	0.989795918	0.755388941
kctd7	0.989795918	0.586206897
brpf3a	0.989795918	0.842105263
fam212ab	0.989795918	1
rpa3	0.989726027	0.815789474
higd2a	0.989708405	1.226190476
abat	0.989698891	1.040867389
slc8a4a	0.989690722	0.302325581
tdrd3	0.989690722	0.930555556
gspt1	0.989690722	1.0609319
tagln	0.989633174	0.966415353
fgfr4	0.989619377	1.004273504
rpe	0.989619377	1.242957746
nkrf	0.989583333	0.840490798
rcl1	0.989583333	0.901408451
pbldp	0.989583333	1
slc41a2a	0.989473684	0.935779817
CABZ01071903.1	0.989473684	1.072289157
ube2d2l	0.989473684	1.24137931
cited2	0.98943662	1.110599078
sox7	0.989361702	0.824324324
zdhhc8b	0.989361702	0.827160494
atad1a	0.989361702	1.158730159
si: ch211-223l2.4	0.989361702	1.204545455
tnrc6b	0.989304813	0.899224806
retsat	0.989247312	1.244897959
xpo7	0.989247312	0.571428571

ints7	0.989247312	0.641025641
pum1	0.989228007	0.61423221
gtpbp4	0.989205103	1.01344809
grb2b	0.989197531	1.178294574
si:dkey-176g1.6	0.989189189	2.022058824
eif4h	0.989164087	0.8026534
h2afy2	0.989145183	0.596139569
extl2	0.989130435	1.011494253
galm	0.989071038	1.259493671
mrpl43	0.988970588	1.650442478
si:dkey-16m19.4	0.988950276	0.936305732
tm2d1	0.988950276	1.141975309
ubtd2	0.988888889	0.630208333
tdg.2	0.988888889	0.87755102
CDK18	0.988888889	1.037037037
si:dkey-77f5.3	0.988888889	1.065789474
rac3a	0.988857939	0.855029586
atp5l	0.988768447	0.934297964
myl7	0.988764045	1.529616725
si:dkeyp-75h12.5	0.988668555	0.871621622
prkacaa	0.988636364	0.789308176
arsa	0.988636364	1
mdm4	0.988593156	1.02020202
eif3g	0.98857868	0.766486486
ncor1	0.988505747	0.71942446
nxpe3	0.988505747	1
rc3h1a	0.988505747	1.032786885
senp2	0.988439306	1.175675676
btbd2a	0.988416988	0.838709677
tm2d2	0.988416988	1.621495327
cry2	0.988391376	1.176056338
pcmtd2	0.988372093	0.882352941
si:cabz01102082.1	0.988372093	0.927272727
cyb5r4	0.988372093	1
mfap2	0.988341969	1.022624434
degs1	0.988317757	0.772256729
magi1a	0.988235294	0.338028169
ift57	0.988235294	1.234375
man2b2	0.988235294	1.35483871
ankhd1	0.988235294	0.702702703
cfl2	0.988215488	0.871206514
drg2	0.988188976	1.185950413
rcan1a	0.98816568	1.323770492
si:dkey-21e2.4	0.988095238	0.85625
mrgbp	0.988095238	1.442028986
gmpr	0.988066826	1.022113022
dicer1	0.988023952	0.752212389
snrpg	0.988011988	0.826677316
RASGRF1	0.987951807	0.381818182
rhbdf1a	0.987951807	0.720238095
c17h14orf159	0.987951807	0.802631579
filip1b	0.987951807	1.054794521
cux1b	0.987878788	1.447761194
tprkb	0.987878788	1.510344828
lmbr1	0.987804878	1.614754098
si:dkey-74k8.8p	0.987730061	1
psmc1a	0.987730061	1.062283737
mesdc2	0.987704918	0.868913858
sun1	0.987692308	0.86746988
ddx5	0.98766328	1.374556738
tegt	0.987642116	0.920324427

sepw2a	0.987548638	1.335863378
smyd2a	0.9875	1.324137931
mapre3b	0.987421384	0.96
hlx1	0.987421384	1.041666667
cntnap2a	0.987341772	0.371428571
znf503	0.987315011	0.572706935
syngr2b	0.987234043	0.916
lhx4	0.987179487	0.476190476
amot	0.987179487	0.948275862
pcyt1bb	0.987179487	0.983957219
mtfmt	0.987179487	1.284090909
psme1	0.987179487	2.566666667
si:ch73-390p7.2	0.987096774	2.422018349
fam76b	0.987055016	1.164
dtnbb	0.987012987	0.469387755
pou3f3a	0.987012987	0.496124031
arhgap29b	0.987012987	0.828125
stambpa	0.987012987	0.898089172
prdm2a	0.987012987	0.909090909
cyp1c2	0.987012987	1.109090909
cax2	0.987012987	1.173333333
BMT2	0.987012987	1.25
hmbsb	0.987012987	1.529411765
chordc1a	0.987012987	1.604166667
ran	0.986932172	0.965163183
vsx2	0.986842105	0.642857143
setd5	0.986842105	0.644067797
mlec	0.986842105	0.892086331
dld	0.986842105	0.944444444
med20	0.986842105	1.086956522
ddx27	0.986842105	1.385214008
alad	0.986842105	1.473214286
si:ch211-175m2.5	0.986842105	2.142857143
ndufv1	0.98682284	1.032135453
vps16	0.986784141	0.8125
cd2ap	0.986784141	0.933649289
mtmr9	0.986784141	1.028735632
prkci	0.986772487	1.244582043
gcsha	0.986737401	0.997584541
fabp7a	0.986714801	0.840136538
mtmr3_1	0.986666667	0.65625
lctlb	0.986666667	0.680851064
ddx39aa	0.986588433	0.831031682
foxa3	0.986486486	0.728813559
sbk3	0.986486486	0.89010989
TMEM179 (1 of many)	0.986486486	1.494736842
im:7154473	0.986486486	2.173913043
abcb8	0.986394558	1.027210884
gyltl1b	0.98630137	0.666666667
spata5l1	0.98630137	0.835616438
chchd4b	0.98630137	1.288135593
purba	0.986282579	0.786259542
atp5c1	0.986250358	1.037716872
csnk2a2a	0.986206897	1.051094891
notch2	0.986175115	0.857142857
tmco1	0.986111111	1.549342105
si:ch211-238n5.4	0.986111111	0.714285714
srsf2b	0.986111111	0.744
lmx1bb	0.986111111	1
ptpn9b	0.986111111	1.06
aldh7a1	0.986064392	1.005860416

nr3c1	0.986062718	0.99078341
ubxn1	0.986062718	1.220394737
pgd	0.986040609	1.065371025
mrpl54	0.986027944	1.264637002
MCRIP2	0.985948478	1.168859649
skp1	0.98592	1.042518128
CABZ01063757.1	0.985915493	0.69047619
tjp2b	0.985915493	0.695945946
crcp	0.985915493	1.147540984
opa1	0.985915493	1.304054054
ak1	0.98589939	0.94042383
mapta	0.985835694	0.683246073
atp6v0d1	0.985735736	1.284062758
tmed7	0.985731272	0.637969095
usp38	0.985714286	0.985074627
pogzb	0.985714286	1.009433962
prr15la	0.985714286	1.017241379
prrg2	0.985714286	1.124260355
meis1b	0.985685072	0.827218935
fam214a	0.985576923	0.937853107
slc35e1	0.985507246	1.057142857
crygm2d20	0.985436893	0.965593975
zgc:85789	0.98540146	1.142857143
prep	0.985365854	1.251798561
lrrtm1	0.985294118	0.603773585
abhd2b	0.985294118	0.855072464
tmem68_2	0.985294118	1.491803279
trim63b	0.985250737	1.058536585
bre	0.985221675	1.042801556
ddx3b	0.985203453	0.663500678
metrn	0.985185185	1.068965517
ABHD12B (1 of many)_1	0.985185185	1.178343949
zgc:158846	0.985180412	1.237322515
tmem214	0.985163205	0.914373089
bhlhe22	0.985163205	0.917391304
rragca	0.985148515	1.357142857
cbx1b	0.985130112	0.955555556
lrit3a	0.985074627	0.527027027
CABZ01078098.1	0.985074627	0.860465116
si:zfos-1011f11.1	0.985074627	0.964912281
slc38a6	0.985074627	1.131147541
nelfe	0.985	1.016393443
clic4	0.984984985	0.939655172
rdh14b	0.984962406	0.786885246
ercc6	0.984962406	1.265822785
bcl7bb	0.984962406	1.623931624
mt-nd5	0.984917886	1.131601839
tox	0.98488121	0.295964126
zgc:123035_2	0.984848485	0.518072289
e2f8	0.984848485	0.518518519
cyp2aa3	0.984848485	0.548387097
acat2	0.984848485	1.048387097
cul5a	0.984848485	1.1
pdcd2	0.984848485	1.373134328
DNAJA4	0.984848485	1.440366972
si:ch211-210c8.6	0.984848485	1.552763819
btr02	0.984848485	1.896551724
c23h20orf24	0.98482933	0.899061033
sod2	0.984821429	1.109555189
eif2b5	0.984790875	0.917073171
rrp7a	0.984732824	0.931271478

cse1l	0.98470948	0.996138996
USP31	0.984615385	0.875
engase	0.984615385	1
smpx	0.984615385	1.170833333
CABZ01117131.1	0.984615385	0.693989071
supt4h1	0.984562607	1.086206897
usp8	0.984536082	0.763736264
zgc:154075	0.984496124	1.274193548
sde2	0.984455959	0.786666667
CRIP2	0.984455959	1.275974026
nup98	0.984375	0.814814815
cog2	0.984375	1.18852459
dnal1	0.984375	2.357142857
thrap3b	0.984334204	0.691616766
timmdc1	0.984326019	1.409836066
cnn1b	0.984293194	1.49068323
ufm1	0.984269663	1.239892183
tmem38a	0.984266667	1.063610178
csnk2a1	0.984149856	0.746626687
usp13	0.984126984	1.034883721
ctr9	0.984126984	0.719457014
fam217b	0.984126984	1.075
cnot10	0.984126984	1.095744681
her2	0.984126984	1.121212121
alg14	0.984126984	1.121621622
prrc2c	0.984042553	0.666666667
akt2l	0.984018265	0.766730402
wdtc1	0.984	0.930693069
atad3b	0.983974359	0.852813853
ube2v2	0.983930779	1.035658915
ano10a	0.98392283	1.235714286
tnrc6c1	0.983870968	0.691729323
fat1a	0.983870968	0.877862595
kcmf1	0.983870968	0.901315789
ttc17	0.983870968	0.866666667
fhod3a	0.983870968	0.946902655
5S_rRNA_291	0.983870968	1.032520325
bag5	0.983870968	1.068181818
trmt13_1	0.983870968	2.054054054
SNORD99	0.983870968	4.05
G3BP2 (1 of many)	0.983823529	0.661861075
rnf10	0.983805668	0.804087385
tmed9	0.983758701	1.507607192
lonp2	0.983695652	0.832432432
tasp1	0.983606557	1.133333333
dpysl5a	0.983539095	0.441396509
igfn1.1	0.983516484	1.098786828
zic4	0.983471074	0.730337079
phf12a	0.983471074	0.768421053
crmp1	0.983425414	0.417322835
vgll4b	0.983425414	0.881081081
pvalb7	0.98340249	1.1
lrrc20	0.983372922	0.896253602
dhx16	0.983333333	0.738738739
arhgef18b	0.983333333	1.025641026
camsap2b	0.983333333	1.048780488
dcp1b	0.983333333	1.333333333
ugt2a6	0.983333333	1.411764706
si:ch73-234i15.6	0.983333333	1.935483871
zgc:85858	0.983333333	1.156028369
parp3	0.983193277	1.346938776

sh3glb1b	0.983146067	1.129943503
ddx54	0.983050847	0.814102564
gnsb	0.983050847	1.166666667
rplp2	0.983011511	0.893137848
nrp2a	0.982978723	0.656862745
serbp1a	0.982912105	0.802447721
dctn1b	0.982905983	0.462686567
eya3	0.982905983	0.775510204
tfdp2	0.982905983	0.798319328
dynll2b	0.982905983	1.010050251
taf12	0.982798165	1.455182073
diras1a	0.982785603	0.91087344
klf11b	0.982758621	0.796875
prdm10	0.982758621	0.630434783
wdr20a	0.982758621	0.785714286
ankha	0.982758621	1.878787879
cpa5	0.982734614	1.206059023
ogdha	0.982706002	0.949700599
si:ch73-21k16.6	0.98265896	0.737288136
nckap1	0.982547993	0.797235023
ahr2	0.982517483	0.995652174
psma6b	0.982507289	0.934911243
wasla	0.98245614	0.728643216
tcirg1a	0.98245614	0.918367347
zgc:162472	0.98245614	1.1875
ftsj1	0.98245614	1.24742268
sh3gl3a	0.98245614	1.384615385
vps9d1	0.98245614	1.444444444
dapk2a	0.98245614	1.69047619
mccc1	0.982417582	0.812345679
aclya	0.982404692	0.785211268
cyp2aa4	0.982300885	0.725
barhl1a	0.982300885	0.785714286
FKBP15	0.982300885	0.93
homer1b	0.982222222	0.707423581
rabgap1l	0.982206406	0.928571429
tamm41	0.982142857	0.607142857
si:dkey-7l6.3	0.982142857	0.791666667
nr2e1	0.982142857	0.871794872
ankrd24	0.982142857	0.921568627
zfyve9a	0.982142857	1.217948718
sharpin	0.982142857	1.297297297
si:ch211-127m7.2	0.982142857	1.677419355
zgc:136864	0.982142857	1.030973451
ldhba	0.982077273	0.882828542
si:ch211-146m13.3	0.981981982	0.613333333
insra	0.981927711	0.75
pdlim1	0.981927711	1.391585761
slc51a	0.981900452	1.265957447
ptx3a	0.981873112	1.165467626
ctsla	0.981859698	1.490046163
cs	0.981825213	1.030013049
si:ch211-235f12.2	0.981818182	0.520467836
gemin5	0.981818182	0.822222222
elnb	0.981818182	0.836363636
capn10	0.981818182	0.872340426
fam83hb	0.981818182	1.223529412
fbxo8	0.981818182	1.594936709
mcat_2	0.981818182	1.645833333
spoplb	0.981818182	0.985714286
tnnt1	0.981818182	1.15483871

cpsf1	0.98173516	0.860606061
smyd1a	0.981692232	1.017748479
cnih2	0.981651376	0.552631579
pikfyve	0.981651376	0.870588235
slc35a4	0.981651376	1.241758242
sept5a	0.981617647	1.381165919
zgc:113201	0.981595092	0.904761905
sept8a	0.981595092	0.91218638
ddx59	0.981481481	0.707692308
dbnlb	0.981481481	0.808823529
slc25a1a	0.981481481	1.095652174
phlda3	0.981481481	1.12244898
mycbp	0.981481481	1.287671233
n4bp2	0.981481481	1.921052632
gnai1	0.981395349	1.055445545
pfkla	0.98136646	0.922413793
slc40a1	0.981333333	0.851002865
gse1	0.981308411	0.593023256
sema3bl	0.981308411	0.741176471
phf8	0.981308411	0.834482759
fgf13a	0.98125	0.641891892
dcaf6	0.98125	0.870229008
ptges3a	0.981150794	1.490243902
si:ch1073-155h21.2	0.981132075	0.444444444
gng10	0.981132075	0.538461538
zgc:153169	0.981132075	1.018518519
mnd1	0.981132075	1.533333333
tpm2	0.981075269	1.099500416
tgfbr2	0.981012658	1
SIKE1	0.981012658	1.222222222
wfs1a	0.980952381	0.900990099
dennd4c	0.980952381	1.010869565
mkrn2_1	0.980952381	1.4375
eftud2	0.98089172	0.623762376
stx10	0.98089172	1.3046875
sgta	0.980874317	1.131539611
hmgcl	0.980830671	1.142857143
fam43a	0.980821918	0.923512748
supt16h	0.980815348	0.752089136
nono	0.980769231	0.565084227
crabp2b	0.980769231	0.623115578
tubgcp3	0.980769231	0.700854701
snip1	0.980769231	0.73006135
dpp7	0.980769231	1.021943574
CU633479.1	0.980769231	1.524193548
gria3a	0.980769231	0.506024096
SPRTN	0.980769231	0.842105263
si:dkey-57k2.7_1	0.980769231	1.142857143
acad8	0.980769231	1.359756098
plekha3	0.980769231	1.382716049
FAM188B	0.980769231	1.43902439
tnnt2e	0.980683507	1.075889328
stxbp3	0.980620155	1.200956938
rps3	0.980594187	0.900410637
swap70b	0.980582524	0.743589744
senp3a	0.980582524	1.289855072
gars	0.980563654	1.022335025
ap3d1	0.980544747	0.868571429
atox1	0.980417755	1.138107417
onecut3a	0.980392157	0.576923077
prkacbb_1	0.980392157	1.291139241

hexdc	0.980392157	1.540540541
si:dkey-16l2.20	0.980392157	2.0625
hat1	0.980306346	1.050699301
exosc7	0.980263158	0.984126984
ncoa2	0.98019802	0.746835443
narfl	0.98019802	1.585714286
rpa2	0.980148883	0.963752665
zgc:86709	0.98013245	0.550684932
nr2f2	0.980072464	0.497395833
clstn1	0.980016653	0.422446406
frmpd1b	0.98	0.459459459
cdc6	0.98	0.86407767
cmtr2	0.98	1.195121951
tmco6	0.98	1.396226415
BX005085.1	0.98	1.510638298
agpat5	0.979919679	1.079069767
aqp11	0.979919679	1.353658537
mta3	0.979899497	0.714285714
lin7b	0.979879276	1.134803922
psma3	0.979877515	1.380213385
hspa4b	0.979818365	1.153846154
git1	0.97979798	0.802631579
piezo2a.2	0.97979798	0.868131868
bricd5	0.97979798	1.5
fth1b	0.979674797	1.015936255
stag2b	0.979643766	0.84
igsf9b	0.979591837	0.526315789
ret	0.979591837	0.550724638
ago1	0.979591837	0.942857143
si: dkey-225f5.4	0.979591837	1.388888889
pip5k1ba	0.979591837	1.428571429
si:dkey-253d23.3	0.979591837	1.509803922
zgc:136930	0.979564165	1.079263264
eci1	0.979559748	1.17961165
dctn4	0.979452055	0.873873874
chmp6b	0.979439252	1.037974684
chchd1	0.979423868	1.631284916
arpc4	0.979400749	1.151515152
arpc3	0.979397781	1.093831001
sept5b	0.979381443	0.357142857
eif3k	0.979302832	1.048152296
traf3ip2b	0.979166667	1.260869565
mon1a	0.979166667	1.733333333
si:dkey-19a16.7	0.979166667	1.739130435
zic5	0.979166667	0.792
irf2bp1	0.979079498	0.700787402
gdi2	0.979046836	0.903949293
taf1	0.979020979	0.757894737
CU856539.1	0.978947368	0.718061674
tmem145	0.978947368	0.982758621
aqr	0.978873239	0.697183099
grin1a	0.978858351	0.581218274
cops4	0.978846154	1.208955224
cox17	0.978841871	1.344626168
scyl3	0.978723404	1.290598291
ubap1lb	0.978723404	0.488888889
lrwd1	0.978723404	0.861111111
mhc1laa	0.978723404	2.821428571
si:dkey-193e13.8	0.978672986	1.229398664
eif4ebp2	0.978636826	1.111789859
glod5	0.978632479	1.160305344

si:ch211-79k12.1	0.978632479	1.404255319
pde6g_1	0.978586724	1.079726651
epb41l3a	0.978571429	0.365217391
sirt5	0.978571429	0.932773109
hdgfrp2	0.978494624	0.651639344
ahcy	0.97849162	0.952177693
ddi2	0.978448276	0.834224599
si:ch211-284f22.3	0.978417266	1.227848101
myom1a	0.978381375	1.276257161
mov10a	0.978378378	1.01754386
tnni1c	0.978328173	0.990875912
epcam	0.978291317	1.510152921
waslb	0.97826087	0.691428571
pcolceb	0.97826087	0.724637681
fam65c	0.97826087	0.98
CABZ01069006.1	0.97826087	1.078431373
il6r	0.97826087	1.166666667
trdmt1	0.97826087	1.71875
mtmr7a	0.97826087	1.878378378
psmd10	0.97826087	1.427419355
syt4	0.978125	0.768181818
mut	0.978090767	0.831223629
rbpms2b	0.978021978	0.733766234
them4	0.978021978	1.385542169
myef2	0.978021978	0.716627635
fubp1	0.977992958	0.86440678
ppib	0.977961433	0.942397138
myl12.1	0.977883781	1.044023773
tmem59	0.977883096	1.154093098
tub	0.977777778	0.35483871
si:dkey-22o22.2	0.977777778	0.676470588
BCR (1 of many)	0.977777778	0.75
zgc:91860	0.977777778	0.795620438
dlx2b	0.977777778	1.25
zbtb34	0.977777778	1.28125
CABZ01058650.1	0.977777778	0.932
fam199x	0.977777778	1.295918367
mrps28	0.977678571	0.911439114
robo3	0.977653631	0.670886076
chordc1b	0.977635783	1.746887967
zgc:100906	0.97761194	1.414141414
rab14	0.97759434	1.106182796
slc25a4	0.977580517	1.175442503
psmg1	0.977578475	1.097457627
crip1	0.97755102	1.298119964
ampd2b	0.97752809	0.554054054
c8g	0.977477477	0.846689895
myclb	0.977477477	1.171597633
csnk2b	0.977378576	0.8979442
c3a.6	0.977272727	0.808290155
ppp1r12c	0.977272727	1.087378641
chchd6b	0.977272727	1.091133005
cacnb2b	0.977272727	0.551724138
tmem161b	0.977272727	0.75
puf60b	0.977272727	0.825581395
mrpl9	0.977272727	1.270114943
stox1	0.977272727	1.35483871
slc16a5a	0.977272727	1.62962963
si:cabz01007807.1	0.977272727	1.8
dynll1	0.977127859	1.220007971
atic	0.977099237	0.733480176

tmtc4	0.977099237	0.974358974
gpaa1	0.977099237	0.990566038
snf8	0.977099237	1.50862069
ccdc174	0.977099237	1.540540541
arglu1a	0.977040816	1.42804428
wipf1b	0.977011494	0.85106383
ankrd46a	0.977011494	0.867816092
mrpl11	0.976982097	0.725225225
mmadhc	0.976982097	1.277227723
bysl	0.976945245	0.994993742
znf646	0.976923077	0.894736842
dnmt1	0.97689769	0.647302905
fbn2b_1	0.976863753	0.960784314
CT826376.1	0.976744186	0.487804878
pkn2	0.976744186	1.070422535
crb3a	0.976744186	1.129032258
znf740b	0.976744186	1.370967742
p4ha1a	0.976608187	1.112903226
uspl1	0.9765625	0.911564626
oser1	0.976525822	1.016853933
kpna4	0.976491863	0.772925764
nxt2	0.976470588	1.5875
glo1	0.976419214	1.155204461
lancl1	0.976377953	0.920634921
slc25a44b	0.976190476	1.219298246
cav3	0.976190476	0.9546875
si:dkey-217d24.6	0.976190476	0.76
rgs9b	0.976190476	0.857142857
nubp1	0.976190476	1.104477612
srpk1a	0.976119403	0.868131868
acsl3b	0.976109215	0.847826087
mllt10	0.975903614	0.652777778
gpr143	0.975903614	0.77173913
rnf26	0.975903614	1.934782609
rhebl1	0.975877193	1.568627451
fstl1a	0.975830816	0.907303371
psmb3	0.975819033	1.149277689
si:ch73-52f15.5	0.975806452	1.220689655
metrnl	0.975806452	0.923267327
atpif1a	0.975784753	1.06504065
atp5f1	0.97569935	1.019179542
si:ch211-191j22.3	0.9756691	1.109048724
lrp2a	0.975609756	0.588235294
slc43a3a	0.975609756	0.785714286
lrch1	0.975609756	0.819444444
dnajc17	0.975609756	0.838709677
met	0.975609756	0.861111111
prdm5	0.975609756	0.870967742
atp7a	0.975609756	0.888888889
atxn7	0.975609756	0.96
nccrp1	0.975609756	0.979899497
parvb	0.975609756	1.005681818
tnrc5	0.975524476	1.40201005
cpe	0.975520196	0.492537313
atcaya	0.975460123	0.487951807
lgsn	0.975460123	1.262222222
brk1	0.975425331	0.970204842
si:ch211-231f6.6	0.975409836	1.265151515
pfdn5	0.97535668	1.17769131
wbp2	0.975308642	0.590163934
casp2	0.975308642	1.866666667

cyp2k16	0.975225225	1.263546798
spock2	0.975216853	0.691525424
ranbp3b	0.975206612	0.849673203
arl2	0.975206612	1.568047337
gata6	0.97515528	0.80239521
ranbp10	0.97515528	1.125
yif1a	0.975124378	1.25
fabp3	0.975050123	0.955156951
fat3a	0.975	0.461538462
sb:cb81	0.975	0.598360656
phactr4b	0.975	0.676190476
ambra1a	0.975	0.967741935
sucla2	0.975	0.975991649
rab33ba	0.975	1.153846154
myf5	0.975	1.166666667
wdr60	0.975	1.230769231
si:ch211-132d3.4	0.975	1.446494465
guca1a	0.975	1.833333333
BX530067.1	0.975	2.465116279
itm2cb	0.974943052	1.347457627
si:dkey-42i9.6	0.974916388	0.986863711
tceb1b	0.974852071	1.469945355
aftpha	0.974842767	1.040322581
mthfr	0.974842767	1.296875
mycn	0.974811083	0.651810585
hnrnpa1a	0.974789916	0.562457338
spsb1	0.974789916	1.059405941
mapk4	0.974683544	0.853658537
si:ch211-191a24.4	0.974683544	1.328767123
LSM3	0.974630021	1.103921569
efna1a	0.974619289	1.590361446
mrpl24	0.974595843	1.095555556
eif3i	0.974582173	0.929258455
samd11	0.974576271	0.575471698
trim33	0.974489796	0.583850932
usp25	0.974468085	0.811715481
dlgap5	0.974358974	0.697916667
galca	0.974358974	0.87962963
tab2	0.974358974	0.972477064
limk1a	0.974358974	0.414634146
pvrl3b	0.974358974	0.90625
aadac	0.974358974	1.181818182
fgl2a	0.974358974	1.375
bin2b	0.974358974	1.85
psma4	0.974327628	1.136856369
myh10	0.974304069	0.873994638
slc30a1a	0.974276527	0.881136951
MRPL49	0.974248927	1.16194332
mrps6	0.974238876	0.862197393
aebp2	0.974193548	0.58974359
rnf8	0.974193548	0.862857143
pole4	0.974137931	1.133333333
dmtn	0.974137931	1.298850575
avl9	0.974137931	1.5
tmem56b	0.974074074	1.099099099
anxa4	0.973947896	0.945673077
rpsa	0.973923273	0.892844322
hip1	0.973913043	0.765306122
PLEKHH3	0.973913043	0.814814815
CABZ01044611.1	0.973913043	0.842696629
tjp3	0.973913043	0.919191919

dnase1l3	0.973913043	1.440366972
maf1	0.973799127	2.152317881
maf1	0.973799127	2.152317881
pcca	0.973747017	1.359281437
zgc:114045	0.973684211	0.625
znf516	0.973684211	0.681818182
pskh1	0.973684211	0.810810811
lrp1ab	0.973684211	0.816513761
map3k1	0.973684211	0.890909091
chek2	0.973684211	0.951219512
puraa	0.973684211	1
rab5ab	0.973684211	1.017094017
stab1	0.973684211	1.220338983
vipr1a	0.973684211	1.92
DDX17	0.973614776	0.841359773
tnrc6a	0.973607038	0.767716535
faf2	0.97359736	0.740350877
slc30a7	0.973568282	0.857142857
ptdss2	0.973544974	0.881944444
dnm1a	0.973509934	0.459770115
tmem182a	0.973493976	1.191904048
mapre1b	0.973478939	1.28975265
col9a2	0.973473684	0.664939551
si:ch211-282j22.3	0.973451327	1.333333333
inhbb	0.973451327	1.457446809
ampd1	0.97343296	1.033325672
bop1	0.973404255	0.768292683
stk24b	0.973404255	0.959731544
gbas	0.973342003	1.122869318
ADCY3 (1 of many)	0.973333333	0.520833333
SLC52A2	0.973333333	1.107692308
ccbl1	0.973262032	1.728915663
calm3b	0.97317314	1.222158658
rps6kal	0.973154362	1.101851852
ruvbl2	0.97311828	1.050314465
sdhaf3	0.97311828	1.348993289
rnps1	0.973076923	0.678137652
cnot6l	0.973063973	1.022284123
eif4a1b	0.973031284	0.992628087
stag2a	0.972972973	0.820359281
wbscr16	0.972972973	0.958974359
si:ch211-196c10.13	0.972972973	0.620689655
ipo9	0.972972973	0.764227642
cntfr	0.972972973	0.820717131
zdhhc16a	0.972972973	0.883116883
pkdccb	0.972972973	1.255813953
zgc:162971	0.972972973	1.523809524
naa38	0.972826087	1.286516854
aven	0.972789116	1.136
CU639413.1	0.972727273	0.639097744
SCYL2	0.972727273	0.835616438
cplx2l	0.97271487	0.465838509
pdcd10a	0.972677596	1.013888889
unc119b	0.97260274	0.965686275
pdcd7	0.97260274	0.910714286
polr2l	0.97260274	0.939393939
twsg1a	0.972477064	0.771428571
aldh2.1	0.972375691	0.977987421
GABARAPL1	0.972328244	1.36440678
grwd1	0.972318339	1.061151079
pxna	0.972307692	0.95

fam136a	0.972270364	1.189380531
gpr176	0.972222222	0.482758621
trim3a	0.972222222	0.5875
kctd8	0.972222222	0.722222222
b9d1	0.972222222	0.99047619
dnm2a	0.972222222	1.043103448
sacm1la	0.972222222	1.080645161
kdf1a	0.972222222	1.129032258
zgc:162952	0.972222222	1.193548387
ncs1b	0.972222222	1.608695652
amn1	0.972222222	1.862068966
f10	0.972144847	0.701123596
si:ch211-166a6.5	0.972121212	1.027874564
AK6	0.972111554	1.083601286
dynlrb1	0.972088547	1.632707775
rac1a	0.972039474	1.036121673
si:dkeyp-72h1.1	0.972027972	1.08496732
cpa4	0.971976401	1.661347518
fam214b	0.971962617	0.866666667
scn3b	0.971962617	1.301369863
cap1	0.97188755	0.723905724
pias1b	0.971830986	0.78125
fam134c	0.971830986	1.161616162
gpn1	0.971830986	1.6
mfap3l	0.971830986	1.238095238
tufm	0.971751412	1
sh3gl3b	0.971698113	1.297297297
edc4	0.971631206	0.716981132
gatad1	0.971590909	1.432432432
sostdc1a	0.971590909	1.644970414
ebag9	0.971576227	1.075566751
uqcrfs1	0.971563981	0.985282093
prpf39	0.97148289	0.794055202
SATB1	0.971428571	0.288888889
map2	0.971428571	0.371134021
thbs3a	0.971428571	0.6
nol4la	0.971428571	0.625
cnr1	0.971428571	0.714285714
si:dkey-184a18.5	0.971428571	0.755555556
SMC6	0.971428571	1.037037037
msh3	0.971428571	1.095238095
slc22a15	0.971428571	1.163934426
FO704661.1	0.971428571	1.25
dguok	0.971428571	1.369230769
malt3	0.971428571	2.066666667
ntn1b	0.971428571	0.388059701
kbtbd12	0.971428571	1.157068063
fbxl6	0.971428571	1.823529412
LINC00998	0.971346705	1.137333333
gigfy2	0.971311475	0.879581152
mib1	0.971291866	0.788819876
mgrn1a	0.971291866	1
flot2b	0.971264368	0.993103448
rbfox1l	0.971264368	1.211678832
fam213aa	0.9712313	1.184576485
prex1	0.971153846	1.28125
mmgt1	0.971134021	1.034739454
gps1	0.971052632	1.334328358
hp1bp3	0.971014493	0.801104972
bcat1	0.971014493	1.097826087
slc4a4a	0.970930233	0.351648352

ambp	0.970862471	1.056277056
rgs3a	0.970833333	1.088888889
mier3a	0.970760234	0.848684211
egfl6_1	0.970684039	0.971297359
slc6a8	0.970684039	1.224806202
camkvl	0.970588235	0.607142857
foxf2a	0.970588235	0.765306122
isl2b	0.970588235	0.9
dock9b	0.970588235	1.040816327
CR753886.1	0.970588235	1.245901639
mab21l2	0.970588235	0.612939841
crata	0.970588235	0.820069204
ybx1	0.970552667	0.787950639
bcl2l10	0.970521542	1.348958333
slc25a28	0.970501475	0.975935829
atp6v1ab	0.970479705	0.787671233
ugdh	0.970414201	1.458333333
tox2	0.97037037	0.473684211
pcid2	0.97037037	1.030075188
smx5	0.970338983	1.055776892
CABZ01112656.1	0.97027972	0.790251108
cdk5r1b	0.970247934	0.970223325
arpc1a_1	0.970238095	1.094927536
arhgap12b	0.970149254	0.601851852
nova1	0.970149254	0.612903226
baiap2b	0.970149254	0.760869565
ppp2r3b	0.970149254	1.192982456
pcdh1g14	0.970149254	1.204545455
ap3m2	0.970099668	0.830039526
hsp90aa1.2	0.970099668	0.961038961
khsrp	0.970074813	0.707865169
rap1aa	0.970031546	1.143859649
sf3b4	0.97	0.517751479
mapk3	0.969945355	0.858131488
jam3a	0.969849246	1.694656489
gabpb1	0.96969697	0.516129032
tmem265	0.96969697	0.516129032
rapgef1a	0.96969697	0.652173913
qki2	0.96969697	0.847826087
zcchc14	0.96969697	0.857142857
samd4a	0.96969697	0.983333333
mif	0.96969697	1.075901328
OLFM4 (1 of many)_2	0.96969697	1.534188034
fbxl3b	0.96969697	0.858695652
fhl3a	0.96969697	1.615384615
mt-atp6	0.96953872	1.183042008
trappc6bl	0.969512195	1.7109375
cdc42l	0.969494757	1.206736353
ncam1b	0.969465649	0.431578947
pddc1	0.969387755	1.3375
si:dkey-29p10.4	0.969387755	1.732142857
frem2b_2	0.969325153	1.082474227
uso1	0.969325153	1.359223301
dnaja2	0.969311377	0.989031079
rnf2	0.969278034	0.998381877
lrit1b	0.969230769	0.299145299
mamdc1	0.969230769	0.37037037
wscd1b	0.969230769	0.468085106
C9orf172 (1 of many)_1	0.969230769	0.727272727
bcar1	0.969230769	0.752380952
fbn1	0.969230769	0.808823529

bhlhe23	0.969230769	1.080808081
dqx1	0.969230769	1.083333333
ist1	0.969230769	1.183937824
faimb	0.969135802	1.02247191
si:dkey-245p14.7	0.969135802	1.094017094
cdk9	0.969094923	0.95631068
kpnb1	0.969084423	0.740740741
soga1	0.969072165	0.550724638
FBXO48 (1 of many)_2	0.969072165	0.752688172
efna1b	0.969072165	1.453125
ttc27	0.968992248	0.894736842
prkar2aa_1	0.968970381	0.880484115
scara5	0.96875	1.397058824
crb1	0.96875	0.454545455
apcdd1l	0.96875	0.701298701
phip	0.96875	0.833333333
si:zfos-905g2.1	0.96875	1
si:ch73-217n20.1	0.96875	1.166666667
recql	0.96875	1.288135593
mras	0.96875	1.421052632
myl9a	0.968712395	0.944146079
aspg	0.968553459	1.176
fgfrl1b	0.968503937	0.75
ndufa4l	0.968449198	1.092821967
si:ch211-195b11.3	0.968424705	1.470510563
prrx1a	0.968421053	1.177606178
CASKIN1 (1 of many)	0.968421053	0.434343434
gramd4b	0.968421053	0.949152542
ctnnbip1	0.968390805	1.074468085
st8sia6	0.968379447	1.759162304
slc12a10.2	0.96835443	1.641509434
si:ch211-222l21.1	0.968352467	0.793443618
capzb	0.968312757	1.023387401
pdcb	0.968270215	0.995150339
fam69aa	0.968253968	1.19047619
snrnp48	0.968253968	1.833333333
tpte	0.968181818	0.996389892
15-Sep	0.968134958	1.107407407
eif3ha	0.968091895	0.923351159
pygmb	0.968055556	0.750421585
ube2nb	0.96804261	1.180851064
slirp	0.968028419	1.03539823
si:dkey-40m6.8	0.968	0.525641026
farsa	0.967959528	0.966796875
IWS1	0.967938931	1.25
sccpdha	0.967914439	0.908396947
gusb	0.967914439	1.155555556
MARK4 (1 of many)	0.967889908	0.683060109
nedd8	0.967824968	1.247222222
gng3	0.967805519	0.860813704
elavl3	0.967741935	0.368983957
vangl2	0.967741935	0.677419355
gpkow	0.967741935	1.18579235
zeb1b	0.967741935	0.780821918
dre-let-7g-1	0.967741935	0.8
si:dkey-159a18.1	0.967741935	0.928571429
larsa_1	0.967741935	0.987804878
RSPH1	0.967741935	1.038461538
slc25a19	0.967741935	1.136986301
adgrf3b	0.967741935	1.391304348
myo19	0.967741935	1.588235294

zgc:162025	0.967741935	1.764705882
wdr1	0.96759676	0.844793713
pmela	0.967485549	0.973811165
flvcr1	0.967479675	0.526785714
trim3b	0.967479675	0.757425743
si:busm1-57f23.1	0.967479675	1.173728814
ndufb2	0.967464115	0.98053528
otub1a	0.96742671	1.096345515
mynn	0.967391304	0.981595092
med22	0.967391304	1.613636364
tnrc6c2	0.967320261	0.7
kcnj12b	0.967320261	1.294964029
eif4ebp1	0.967268623	0.882040383
etv5b	0.967261905	0.845394737
cpb1	0.967220683	1.198473282
hmgb3b	0.967168263	1.612736661
bag6l	0.967105263	0.695652174
si:ch211-235m3.6p	0.967032967	1.555555556
exoc6b	0.966942149	1.041666667
pip5k1bb	0.966942149	1.5
khdrbs1a	0.966926407	0.970780469
rqcd1	0.966911765	0.953405018
paqr7b	0.966887417	1.034883721
srsf5a	0.966883822	1.234001293
msh2	0.966824645	1.122807018
stt3b	0.966814159	0.681233933
atp1a1a.1	0.966731898	0.694145759
mettl16	0.966666667	0.580645161
zgc:172302	0.966666667	0.631578947
arhgap5	0.966666667	0.643678161
eed	0.966666667	0.696629213
FO681287.1	0.966666667	0.707317073
six5	0.966666667	1.069767442
heatr3	0.966666667	1.194968553
zgc:153073	0.966666667	1.869565217
serhl	0.966666667	1.975903614
ndrg4	0.966611018	0.41745283
atp6v1ba	0.966601179	1.025641026
ube2ia	0.966580977	0.965240642
adcyap1b	0.966457023	1.321336761
mtss1	0.966442953	1.024
nsun2	0.966442953	1.297029703
smyd1b	0.966386555	0.9329806
lnpep	0.966386555	1.818181818
rev3l	0.966292135	1.015384615
flna	0.966277562	0.937861272
zgc:91910	0.966261808	1.237241379
yy1a	0.966257669	0.801470588
cpne2	0.966216216	0.674418605
cul1b	0.966101695	0.867132867
opn4.1	0.966101695	0.333333333
eva1a_1	0.966101695	0.919354839
b3glcta	0.966101695	1.131313131
grhl2b	0.966101695	1.315789474
ddx49	0.966101695	1.78313253
bbip1	0.966037736	1.440528634
dnpep	0.96593674	1.136690647
ZSWIM6	0.965909091	0.415384615
srgap3	0.965909091	0.527272727
vps45	0.965909091	1.628099174
plxna1a	0.965811966	0.630769231

lrrn2	0.965753425	0.840425532
pank4	0.965714286	1.517857143
ube2al	0.965656566	1.815533981
apc	0.965608466	0.553571429
tuba8l2	0.965584101	0.974080268
nipal4	0.965517241	1.24
fam219aa	0.965517241	1.341463415
mettl2a	0.965517241	1.24375
kif21b	0.965517241	0.608695652
plekha7b	0.965517241	0.76
fam160a1b	0.965517241	0.791666667
shisa4	0.965517241	0.863636364
slc29a1a	0.965517241	0.896988906
hmgn3	0.965443913	1.288612565
arl8a	0.965432099	1.394822006
map2k2a	0.965417867	0.919308357
dachd	0.965384615	0.671957672
si:ch211-193k19.1	0.965277778	0.722627737
ergic3	0.965277778	1.5
tspan7	0.965260546	1.297029703
tsr3	0.965217391	0.975609756
med15	0.965217391	1.068965517
calm2a	0.965158606	1.378459687
appa	0.965141612	0.752508361
dhx32b	0.965116279	0.846153846
si:dkey-34e4.1	0.964912281	0.571428571
rbpja	0.964912281	1.1375
si:dkey-71b5.7	0.964912281	1.170731707
ugt2a5	0.964912281	1.325581395
pigc	0.964912281	1.458333333
stat2	0.964912281	2
rragcb	0.964912281	2.0625
nr2f6a	0.964912281	0.701298701
cpt1aa	0.964912281	0.957746479
dcaf8	0.964912281	1.053846154
mapk9	0.964788732	0.988095238
tpbgb	0.964788732	1.564814815
wbscr22	0.964757709	0.896713615
cul4a	0.964705882	0.926267281
gon4l	0.964705882	0.85
plpp7	0.964705882	0.935064935
si:ch211-132g1.3	0.964705882	1.483870968
myl6	0.964673913	1.177464789
ipo7	0.964611872	0.868974042
yod1	0.964566929	1.227979275
ITIH4 (1 of many)	0.964566929	1.431619787
copg2	0.964556962	0.895366218
lgmn	0.964478764	1.413877551
zgc:162396	0.964444444	1.353982301
ccnl1a	0.964412811	1.219178082
dctn3	0.964365256	1.544117647
taf15	0.964320154	0.839766082
tmem244	0.964285714	0.549019608
mllt3	0.964285714	1
MCOLN3 (1 of many)	0.964285714	1.261538462
apol1	0.964285714	1.326086957
rtkn2a	0.964285714	1.833333333
rgs1	0.964285714	3.157894737
nol6	0.964285714	1.045112782
plekha1a	0.964285714	1.052083333
qkia	0.964285714	1.054545455

ak5	0.964285714	0.597402597
hnf4a	0.964285714	0.873417722
zgc:172139	0.964285714	1.082191781
cops8	0.964285714	1.083844581
si:dkey-16m19.1_2	0.964285714	1.413333333
metap2b	0.964224872	1.166666667
nup93	0.964157706	0.639215686
atl2	0.964125561	1.165
tex2	0.964071856	0.932773109
pus1	0.963963964	0.868686869
npepps	0.963946869	0.798226164
dnajc25	0.963768116	1.489795918
ppid	0.96369637	1.1724846
ulk1b	0.963636364	0.929411765
pcdh1g9	0.963636364	0.944444444
arhgef33	0.963636364	2.466666667
eif2s1a	0.963553531	1.510510511
adam17a	0.963541667	1.477876106
wdr37	0.96350365	1.077253219
kcnc1a	0.963414634	0.509803922
zbtb12.2	0.963414634	0.777777778
kat7a	0.963414634	0.96969697
foxp1b	0.963302752	0.522727273
uba1	0.963265306	0.835850957
hook1	0.963235294	1.18556701
sb:cb1058	0.963172805	1.761154856
zgc:101744	0.963125549	0.957734573
zyg11	0.963114754	0.995098039
hadh	0.963110668	1.19558011
slc39a9	0.963099631	1.327102804
timm17a	0.963084495	1.437826541
lamb1a	0.963039014	0.898963731
tuba8l	0.96302051	1.030733546
sccpdhb	0.962962963	0.841945289
exoc4	0.962962963	1.038461538
znf280d	0.962962963	1.525862069
zgc:153929	0.962962963	2
her8a	0.962962963	0.5
dlg1	0.962962963	0.933333333
golim4a	0.962962963	1.014150943
pidd1	0.962962963	1.095238095
serpinb1l4	0.962962963	1.142857143
dync2li1	0.962962963	1.523809524
eda	0.962962963	1.8
rhoaa	0.962930274	1.079520697
atp1b1b	0.962855062	1.521448999
tmem255a	0.962790698	1.009661836
eif1ad	0.962790698	1.406976744
sema3b	0.962765957	0.740458015
rmnd5b	0.962750716	1.13028169
sh3pxd2b	0.962732919	0.699346405
fbxl14b	0.962732919	0.78125
slc25a26	0.962732919	0.886904762
fam96b	0.962732919	1.527131783
rtf1	0.962719298	1.105849582
col10a1a	0.962658701	1.0403317
cmlc1	0.962593516	1.054404145
dlg3	0.962585034	0.659836066
mrps24	0.962555066	0.948275862
atp13a1	0.9625	0.7
csdc2a	0.9625	0.815789474

zbed4	0.9625	0.833333333
lrrc45	0.9625	1.327586207
erf	0.962406015	0.766917293
ccnb1	0.962365591	0.821428571
prkab1b	0.962356792	1.155143339
mapre2	0.962292609	0.926691729
nhsb	0.962264151	0.375
rab43	0.962264151	1.153846154
ccnl1b	0.962264151	1.47826087
lmf2b	0.962085308	0.609375
rab18b	0.962049336	1.153692615
glmna	0.962025316	1.351351351
dnajb12a	0.961977186	1.619791667
ndufa11	0.961975309	0.912937347
cox4i1	0.961904762	1.21920215
thap11	0.961904762	0.761904762
aplp1	0.961818182	0.615275813
map1lc3b	0.961811723	1.65917603
gosr1	0.961748634	0.948571429
loxl2a	0.961734694	1.620567376
fam102bb	0.961722488	1.337931034
chtopa	0.961711712	1.049132948
kdm1a	0.961586121	0.979750779
igdcc3	0.961538462	0.627659574
ptpn13	0.961538462	0.91588785
rybpb	0.961538462	1.073170732
tfcp2	0.961538462	1.220779221
RSBN1	0.961538462	0.75
acoxl	0.961538462	0.820512821
neurl1ab	0.961538462	0.826086957
kctd3	0.961538462	0.835616438
ppp1r1c	0.961538462	0.977777778
wdr62	0.961538462	1
rsph4a	0.961538462	1.047619048
ryr1a	0.961538462	1.047619048
tysnd1	0.961538462	1.148148148
si:ch73-266f23.1_1	0.961538462	1.35
hpse	0.961538462	1.372093023
tmprss4b	0.961538462	2
h3f3d	0.961474037	0.870913428
capns1b	0.961439589	1.204633205
rac2	0.961352657	1.297450425
trim13	0.961290323	0.474025974
atp2b4	0.961218837	0.700704225
tmsb4x	0.961215449	1.167087113
msl2a	0.961165049	0.6
cog7	0.961165049	0.817679558
TMEM87A	0.961089494	1.294392523
pdcd4a	0.961060481	1.028481013
cxxc5a	0.961038961	0.972899729
ino80	0.961038961	1.196078431
cct3	0.960960961	0.889498102
prox1a	0.9609375	0.694805195
golph3	0.9609375	1
capn2l	0.9609375	1.11023622
pafah1b1b	0.960916442	0.916030534
mical3a_1	0.960893855	0.75
vps26b	0.960869565	1.298076923
arl3	0.960824742	1.208430913
hsd17b10	0.960812772	1.068640646
cdkn2aip	0.960784314	1.068181818

tmem237b	0.960784314	1.1375
rergla	0.960784314	1.424657534
rnmt	0.960784314	1.166666667
gak	0.960784314	0.811320755
elmod1	0.960725076	1.444664032
lclat1	0.960674157	0.923566879
ddb1	0.960662526	0.683486239
f3a	0.960629921	1.827586207
nudt9	0.960606061	1.039285714
ssuh2rs1	0.960569551	0.994342291
abl2	0.960526316	0.737704918
sash1a	0.960526316	0.827586207
WIPF3	0.960526316	1.072463768
alg11	0.960526316	1.172413793
usp10	0.960526316	0.632432432
canx	0.960506706	0.785091965
usf1l	0.960431655	1.398148148
eif3ea	0.960412601	0.988573021
si:ch211-165e15.1	0.96039604	1.759259259
mafk	0.96039604	2.245614035
prkacbb_2	0.960352423	1.254237288
ckmt2b	0.96031981	1.090778098
nxf1	0.96031746	1.6
usp28	0.960227273	0.797029703
coq3	0.960199005	0.940909091
cox11	0.960176991	1.478527607
sdha	0.960142068	0.956675508
efnb2a	0.96013289	0.944162437
ildr2	0.96	0.881355932
prc1a	0.96	0.648351648
pex16	0.96	0.85046729
si:ch211-218g4.2	0.96	0.97826087
cenpl	0.96	1
lrrc8ab	0.96	1
hexa	0.96	1.035971223
bcl10	0.96	1.043478261
olig1	0.96	1.069767442
nif3l1	0.96	1.226666667
ormdl3	0.96	1.368421053
ppm1f	0.96	1.4
si:ch211-235m3.5	0.96	1.523809524
nars2	0.96	1.8
kdf1b	0.96	1.9375
fgfr1bl_1	0.96	2.375
atp5d	0.959950556	0.81141046
ankrd54	0.959839357	0.886462882
hmga1b	0.959821429	0.6
pafah1b3_2	0.959821429	1.292428198
casq1a	0.959808612	1.164225941
rnd3a	0.959798995	1.268041237
slc17a6b	0.959770115	0.566115702
arhgef7a	0.959731544	0.605504587
spata2	0.959731544	0.720930233
zgc:113208	0.959677419	1.293333333
mpzl1l	0.95959596	1.168
ncanb	0.95959596	0.444444444
hsf1	0.95959596	0.683168317
zgc:113019	0.95959596	1.4625
CABZ01088345.1	0.959537572	0.940217391
tln1	0.959493671	0.926900585
mapk10	0.959459459	0.505882353

nav3	0.959459459	0.851851852
akap17a	0.959459459	1.036363636
arpc5b	0.959440559	1.500907441
abracl	0.959427208	1.142512077
rab1ba	0.95942029	1.023312883
tsn	0.959302326	1.1875
ube2d2	0.959272727	0.856646394
dpp3	0.959247649	0.935185185
mab21l1	0.959229898	0.802403204
cox16	0.959183673	0.909774436
fblim1	0.959183673	0.679012346
gramd1ba	0.959183673	0.774193548
atp10d	0.959183673	0.955555556
cog8	0.959183673	1.084507042
wacb	0.959183673	1.142857143
sh3bp5la	0.959183673	1.152777778
arr3a	0.959172758	1.089649381
eomesa	0.959016393	0.397058824
cstf1	0.958974359	0.635897436
magoh	0.958947368	1.351001178
lnpb	0.95890411	0.948717949
CABZ01030038.1	0.95890411	0.949152542
dis3l2	0.95890411	1.051724138
sys1	0.95890411	1.324324324
cdc42	0.958885942	1.016793893
scaf11	0.958847737	0.852216749
lsm12b	0.958805355	0.955080214
anxa2b	0.958795563	1.265306122
ampd2a	0.958762887	0.542168675
rps6ka4	0.958762887	1.183333333
zgc:158291	0.958677686	0.436170213
clvs2	0.958677686	0.538461538
fgfr3	0.958677686	0.886010363
pecr	0.958677686	1.0703125
flot1a	0.958598726	1.021466905
ctnnbl1	0.958563536	1.087542088
ap1g1	0.958525346	0.75
agk	0.958525346	1.036649215
gpd1c	0.958506224	1.427807487
prp19	0.958424508	0.74248927
nutf2	0.958402662	0.82717873
hoxc12a	0.958333333	0.973913043
eif4g2a	0.958333333	1.139784946
golga5	0.958333333	1.236453202
rxfp3.3b	0.958333333	0.578947368
sez6a	0.958333333	0.705882353
adam19b	0.958333333	0.805555556
fjx1	0.958333333	1.023809524
si:ch73-138e16.3	0.958333333	1.176470588
trim59	0.958333333	1.1875
hsc70	0.958188153	1.10058309
RAMP1	0.958158996	0.986175115
lnx1	0.958115183	0.560509554
rnf20	0.958115183	0.638888889
akr1a1b	0.958083832	0.989189189
wbp2nl	0.958041958	0.756838906
rabl2	0.958041958	1.452991453
rhcgb	0.958041958	1.671641791
tmem230b	0.958015267	1.323404255
gfm1	0.957943925	1.024539877
crtac1a	0.957928803	0.850993377

slkb	0.957894737	0.789473684
il6st	0.957894737	0.822222222
CU856510.1	0.957894737	0.968421053
pld1a	0.957894737	1
SPTBN4	0.957746479	0.466666667
acap3b	0.957746479	0.829787234
sema3aa	0.957746479	0.71197411
creb3l3l	0.957725948	1.272873194
POLD4	0.957597173	1.459143969
slc17a6a	0.957446809	0.28030303
b4galnt4a	0.957446809	0.392857143
LRRC75A	0.957446809	0.75
sbno2a	0.957446809	0.769230769
si:dkeyp-121d2.4	0.957446809	0.794117647
8-Mar	0.957446809	0.85
hsdl1	0.957446809	0.860465116
si:ch211-125a15.1	0.957446809	0.967032967
mob3c	0.957446809	1.333333333
mrpl21	0.957295374	1.404651163
prdx4	0.957274827	1.015300546
lzic	0.957264957	1.5
rgcc	0.957163959	1.771276596
chd4a	0.957142857	0.46749226
telo2	0.957142857	0.639344262
agap3	0.957142857	0.688888889
acap2	0.957142857	1
ccdc61	0.957142857	1.035087719
cbr1	0.957142857	1.20952381
RAB31	0.957142857	1.4
iqce	0.957142857	1.80952381
col5a1	0.957007259	0.985
oprl1	0.956989247	0.578125
dapk1	0.956989247	0.670886076
vamp2	0.956981664	0.580524345
eprs	0.956947162	0.962059621
smarcd1	0.956912029	0.750988142
slain2	0.956834532	0.825396825
apopt1	0.956790123	1.831775701
camk2d2	0.956749672	0.654513889
cables1	0.956521739	0.5
si:dkey-117n7.5	0.956521739	0.684210526
pdha1b	0.956521739	0.8
nr2f1b	0.956521739	0.826666667
hmox2b	0.956521739	1
si:ch211-74f19.2	0.956521739	1
CU681841.1	0.956521739	1.058823529
entpd5a	0.956521739	1.058823529
si:dkey-32e6.6	0.956521739	1.133333333
cnih4	0.956521739	1.154639175
evi5b	0.956521739	1.222222222
zgc:113426	0.956521739	1.227272727
si:dkeyp-4f2.3	0.956521739	1.285714286
slc25a40	0.956521739	1.453125
dusp28	0.956521739	1.846153846
tgfb1a	0.956521739	1.368421053
CFAP70	0.956521739	0.674418605
si:ch73-49k18.1	0.956521739	1.642857143
tnni2a.1	0.956521739	1.050578915
psmc5	0.95648855	1.170573969
dstyk	0.956349206	0.726666667
sestd1	0.956349206	1.046153846

nfil3-6	0.956331878	1.790322581
sept7b	0.956318253	0.957249071
dhx15	0.956276446	0.711145997
VSTM2B	0.95620438	0.460869565
oaz1b	0.956199877	1.024424285
sult6b1	0.956181534	1.485074627
ubald1b	0.956140351	0.899159664
vgf	0.956097561	0.669291339
klf2b	0.956097561	1.309677419
erlin1	0.956090652	1.28021978
usp7	0.956043956	0.651960784
slc16a10	0.956018519	1.044444444
ppp3r1b	0.95599393	0.897703549
gna13a	0.955947137	1.214634146
glis2b	0.955882353	0.651515152
si:dkey-183i3.5	0.955836777	1.01855018
hif1an	0.955801105	1.039473684
capn1b	0.955696203	0.971962617
sema6e	0.955555556	0.721649485
syngr3b	0.955555556	0.857142857
pdss2	0.955555556	1.459459459
fut9a	0.955555556	0.423076923
atxn7l2a	0.955555556	0.688888889
dnajc15	0.955555556	1.118343195
si:dkey-118j18.2	0.955555556	1.375
zgc:113184	0.955555556	1.4
glrbb	0.955508475	1.07310705
pin4	0.955489614	1.24137931
klf3	0.955445545	0.967889908
gnai2a	0.955445545	1.252124646
hddc3	0.955414013	1.263157895
casp3a	0.955403087	1.269807281
cebpg	0.955399061	1.418338109
hectd3	0.955357143	0.755208333
chp2	0.955307263	1.57037037
cct8	0.955236486	0.92222677
si:ch211-278f21.5	0.955223881	0.762711864
ldb3b	0.955223881	1.22407045
trpm4b.2	0.955223881	1.387755102
rab41	0.955182073	1.259581882
cript	0.955128205	1.215909091
h2afx	0.955112219	0.865580448
barhl2	0.955102041	1.145695364
nipblb	0.95505618	0.752212389
fibpa	0.955017301	1.671794872
bmp2k	0.954954955	0.755555556
nudcd1	0.954954955	1.033333333
smpd1	0.954954955	1.258241758
pon2	0.954954955	1.425641026
apoc1	0.954895779	1.058716507
mgat4b	0.95480226	0.947368421
nubp2	0.95480226	0.969230769
krt15	0.954787234	0.936142485
otomp	0.954666667	1.292974589
snap25b	0.954603581	0.618982118
mob4	0.954584404	1.000821693
mipb	0.954545455	0.699324324
ANO2 (1 of many)	0.954545455	0.375
si:ch73-233f7.1	0.954545455	0.431818182
kcnma1a	0.954545455	0.647058824
fam160a2	0.954545455	0.776315789

lrp8	0.954545455	0.553191489
fam234b	0.954545455	0.772727273
CNGB1	0.954545455	0.8
tfdp1b	0.954545455	0.8
synpr	0.954545455	0.925925926
rnf34a	0.954545455	0.933333333
rab1aa	0.954545455	1.212121212
spata2l	0.954545455	1.432432432
tpgs2	0.954545455	1.451612903
fancc	0.954545455	1.529411765
gfral	0.954545455	1.785714286
rbm25b	0.954407295	0.731404959
ncbp2	0.954268293	1.02173913
camk4	0.954248366	0.969924812
txn	0.954225352	1.464864865
chd4b	0.954212454	0.693467337
rb1	0.954198473	0.8125
ppifa	0.954198473	1.53271028
cdc34a	0.954177898	1.151975684
zc3h13	0.954166667	0.696629213
ube2z	0.954166667	1.428571429
cnn3b	0.95412844	0.834061135
crybb1	0.954077593	0.424836601
ttk	0.954022989	0.543209877
trh	0.954022989	0.675324675
tfip11	0.954022989	0.743902439
src	0.954022989	0.847457627
lpar2b	0.954022989	0.915662651
mospd1	0.954022989	1.040372671
cyp20a1	0.953974895	1.11790393
ctrb1	0.953956985	1.269497493
si:ch211-207l14.1	0.953947368	0.864864865
gch2	0.953900709	1.998550725
tpx2	0.953846154	0.701680672
grm6b	0.953846154	0.808510638
ddr2a	0.953846154	1.019607843
tmem2	0.953846154	1.048387097
si:dkey-11f12.2	0.953846154	1.063829787
trpm1b	0.953846154	1.25
tpi1b	0.953817405	1.274413811
rab14l	0.953810624	1.220963173
esrp1	0.953757225	1.261437908
dars	0.953670706	0.966909332
ethe1	0.953667954	1.023809524
her9	0.953658537	1.253333333
cdkn2aipnl	0.953642384	0.923344948
riok3	0.953535354	1.026634383
ypel1	0.953488372	1
tead1a	0.953488372	0.666666667
wdr32	0.953488372	0.7
herc7	0.953488372	0.911764706
dll4	0.953488372	1.028571429
rbks	0.953488372	1.202898551
plekho2	0.953488372	1.25
si:dkey-19d21.2	0.953488372	1.548780488
ccni	0.953340799	0.798190401
nudcd2	0.953333333	1.101010101
ppp2r4	0.953307393	0.843049327
znrf1	0.953271028	0.544554455
ppp4cb	0.953271028	0.928712871
pgrmc2	0.953271028	1.029962547

get4	0.953271028	1.258461538
ilf3a	0.953177258	0.726495726
mmp23bb	0.953125	0.656716418
FAM163A	0.953125	0.973684211
mhc1ula	0.953125	2.209302326
copb2	0.953072626	1.083847102
rad23b	0.953041622	0.843551797
erc1a	0.953020134	0.494736842
nusap1	0.952991453	1.164021164
foxo4	0.952991453	1.209944751
pop7	0.952941176	0.941704036
smarcb1a	0.952914798	1.229110512
lingo1b	0.952830189	0.555555556
rffl	0.952755906	1.261261261
micu1	0.952736318	0.848039216
pla2g6	0.952702703	0.877192982
tspan5a	0.952702703	0.93
dlx3b	0.952681388	1.079847909
zgc:194665	0.952662722	0.6
zgc:136564	0.952662722	0.769911504
syngr2a	0.952662722	1.278911565
brd8	0.95256917	0.746835443
tpi1a	0.952554745	0.771428571
larp1	0.952531646	0.717054264
col9a1a	0.952512861	0.825866051
znf367	0.952380952	0.612903226
si:ch211-146g19.1	0.952380952	0.861111111
tbcela	0.952380952	1.357142857
efcab7	0.952380952	1.490196078
st6galnac5b	0.952380952	1.8125
LTN1	0.952380952	0.9125
lamc2	0.952380952	0.956521739
gtdc1	0.952380952	1.137931034
pttg1	0.952380952	1.179487179
si:ch1073-342h5.2	0.952380952	1.195121951
pde9a	0.952380952	1.277777778
PLEKHA2	0.952380952	1.4
auh	0.952380952	1.418032787
fam167ab	0.952380952	1.6
pgap3	0.952380952	1.7
ponzr3	0.952380952	1.902017291
try	0.952242015	1.287656584
vars	0.952205882	0.844919786
nkap	0.952182952	1.354545455
nr2f5	0.95212766	0.815642458
pafah1b2	0.95212766	1.269565217
znf414	0.952095808	0.992248062
atg9a	0.952054795	0.915384615
pbx2	0.952054795	1.090517241
esd	0.95202952	0.855345912
gadd45gip1	0.95194508	1.00511509
mbtps1	0.951851852	0.99078341
slc6a16a	0.951807229	1.179487179
nol9	0.951724138	0.985294118
spred2b	0.951690821	0.890243902
zgc:153924	0.951612903	0.485294118
btbd11a	0.951612903	0.6875
gclc	0.951612903	0.828054299
mrpl39	0.951612903	0.831967213
PDZD4 (1 of many)_1	0.951612903	0.891891892
usp45	0.951612903	0.931034483

stc1l	0.951612903	1.06122449
si:ch73-160h15.3	0.951612903	1.510204082
matr3l1.1_2	0.95157385	1.235059761
poldip2	0.951530612	1.224376731
dgcr14	0.951515152	1.338983051
celf2	0.95148248	1.008888889
bdnf	0.951456311	0.925
dnajc11a	0.951428571	0.933333333
nedd4l	0.951388889	0.679245283
ef1	0.951351351	1.046357616
dmtf1	0.951219512	0.833333333
CABZ01047424.1	0.951219512	0.971830986
tmem169b	0.951219512	1.28
zgc:113314	0.951219512	2.375
stap2a	0.951219512	0.413793103
tnfaip8l3	0.951219512	1.465346535
dlgap4a	0.951048951	0.554744526
vps35	0.951030928	0.900332226
MYO9B (1 of many)	0.950980392	0.873239437
si:ch211-210c8.7	0.950980392	1.303030303
tmem259	0.950920245	1.144144144
irs2a	0.950892857	1.854961832
lrrc7	0.950819672	0.425
bivm	0.950819672	0.538461538
CABZ01100870.1	0.950819672	1.46875
emc6	0.950790861	0.747399703
eif2s2	0.950786608	1.028365601
znf706	0.950780312	1.144295302
prodha	0.950777202	1.522033898
f11r.1	0.950715421	1.244147157
endou2	0.950657895	1.353146853
zgc:153293	0.950617284	0.804597701
SPOUT1	0.950617284	1.230769231
nfixb	0.95049505	0.376811594
grk4	0.950413223	0.877777778
bloc1s6	0.950413223	1.152654867
BX572619.1	0.950413223	1.593023256
slc39a7	0.950298211	1.311627907
ank1a	0.950286807	1.187845304
col14a1b	0.950276243	0.801470588
shoc2	0.950248756	0.902777778
zmynd11	0.950226244	0.986486486
ube2v1	0.950131234	0.772423025
enox2	0.95	0.615384615
naalad2	0.95	0.736842105
sgk494b	0.95	0.75
zfhx3	0.95	0.796610169
SASH1 (1 of many)	0.95	0.851851852
sdr16c5a	0.95	0.927672956
hapln1b	0.95	0.973404255
si:ch211-191d2.2	0.95	1.194444444
sh3gl1b	0.95	1.219387755
bmp1b	0.95	0.611111111
zak_2	0.95	0.666666667
rgs7b	0.95	0.740740741
dpy19l3	0.95	0.823529412
si:dkey-51e6.1	0.95	0.89375
chrna9	0.95	0.904761905
chtf8	0.95	0.910714286
si:ch211-136a13.1	0.95	0.916666667
palld	0.95	0.931972789

lingo3a	0.95	0.954545455
mgme1	0.95	1.054054054
OGFOD3	0.95	1.074074074
osbpl2b	0.95	1.185185185
znf1009_3	0.95	1.2
si:dkey-207l24.2	0.95	1.210526316
si:dkey-98f17.5	0.95	1.307692308
si:ch211-134m17.9	0.95	1.315789474
sec22bb	0.95	1.349090909
eef2kmt	0.95	1.736842105
si:ch211-288g17.3	0.949846469	0.63925883
adipor2	0.94984326	0.971342383
lmo1	0.949820789	0.668161435
sept9a	0.949820789	1.061776062
fam60al_1	0.949790795	0.985981308
erh	0.949754902	1.333333333
dip2a	0.949640288	0.666666667
gmfb	0.949604743	1.455062571
cryba2a	0.949499444	1.067198178
hoxc8a	0.949494949	1.306122449
anxa11b	0.949453552	0.882436261
h2afvb	0.949420183	0.875357483
scdb	0.949416342	0.900943396
rbm7	0.949367089	0.761316872
ciz1a	0.949367089	0.779661017
purab	0.949367089	0.781818182
tmem198b	0.949367089	0.826666667
stx6	0.949367089	1.22
si:ch73-238c9.1	0.949367089	1.853448276
dlg1l	0.949275362	0.890909091
pter	0.949275362	1.078431373
efna5a	0.949275362	1.160377358
pomp	0.949268293	0.926306769
vamp1	0.949211909	0.443594646
ppp2r2d	0.949152542	0.602230483
pcyt1aa	0.949152542	1.359223301
CABZ01080009.1	0.949152542	0.963636364
si:ch211-173n18.3	0.949152542	0.971698113
PSMB10	0.949152542	1.022900763
galnt11	0.949152542	1.121827411
megf6b	0.949152542	1.142857143
cbx8b	0.949152542	1.545454545
ptpmt1	0.949152542	1.770833333
stmn4	0.949074074	1.171428571
si:dkeyp-117h8.2	0.949044586	0.546391753
alg2	0.948979592	0.932038835
ehmt1a	0.948979592	0.972972973
pa2g4a	0.94887684	1.040444092
apoa4b.1	0.94881853	1.260975187
si:ch211-134n22.1	0.948717949	0.634146341
tp53bp1	0.948717949	0.669491525
slc35b2	0.948717949	0.76056338
mllt1b	0.948717949	1.098039216
si:dkeyp-67f1.1	0.948717949	2.057142857
si:ch211-194k22.8	0.948717949	1.275555556
fga	0.948646773	0.905859118
syncrip	0.948620362	0.588582677
NFX1	0.948571429	0.772727273
ncoa3	0.948571429	0.940740741
rerg	0.948529412	1.047058824
rabif	0.948529412	1.253846154

gatad2b	0.948453608	0.579710145
tmem201	0.948453608	0.764044944
cdc42ep3	0.948453608	0.888324873
utp20	0.948453608	1.067567568
ino80e_1	0.948387097	1.243055556
kcnj3b	0.948275862	0.367346939
adcy2b	0.948275862	0.711111111
elk3	0.948275862	0.877358491
KCNJ15	0.948275862	0.949152542
slc9a8	0.948275862	1.042105263
abhd5a	0.948275862	1.333333333
sdf4	0.948235294	2.024911032
igbp1	0.948207171	1.124352332
zgc:109965	0.948148148	0.577586207
fam60a	0.948148148	0.874015748
fbxo11a_1	0.948113208	0.848275862
coa3a	0.948076923	1.399082569
git2a	0.948051948	0.673076923
ablim3	0.948051948	0.823529412
sema4bb	0.948051948	1.491525424
ppp2r1a	0.947928994	1.053733426
ssr1	0.947925142	0.823529412
SLC29A4 (1 of many)	0.947916667	1.058823529
kifap3b	0.947791165	1.0625
sec61a1	0.947753396	0.966906026
dus3l	0.947712418	0.982608696
traf7	0.947712418	1.132075472
mphosph8	0.947686117	1.164456233
ebf3a	0.947651007	0.689440994
lasp1	0.947648625	0.945020747
chd9	0.947643979	0.61971831
vamp3	0.947483589	1.371313673
tnni1al	0.947468959	1.032913843
gpd1b	0.94745098	1.023930384
rnf220b	0.947368421	0.526315789
zc3h12b	0.947368421	0.541666667
cep152	0.947368421	0.6
si:ch211-147j13.3	0.947368421	0.695652174
vipr1b	0.947368421	0.733333333
zgc:152977	0.947368421	0.833333333
slc25a35	0.947368421	0.846153846
picalma	0.947368421	0.882352941
tm4sf18	0.947368421	0.923076923
si:ch211-274p24.4	0.947368421	1
git2b	0.947368421	1
CABZ01115396.1	0.947368421	1
si:ch73-138e16.1	0.947368421	1
si:ch73-144d13.5	0.947368421	1.066666667
kcne4	0.947368421	1.25
si:dkey-77f5.13	0.947368421	1.454545455
zgc:56556	0.947368421	1.517241379
si:dkey-22a1.3	0.947368421	1.529411765
dhx40	0.947368421	1.642857143
si:dkey-111k8.5	0.947368421	2.555555556
pkn1a	0.947368421	0.6875
mcm3ap	0.947368421	1
mapkapk5	0.947368421	1.184210526
mrpl3	0.947368421	1.218978102
trappc11	0.947368421	1.267015707
minpp1a	0.947368421	1.59375
pip4k2aa	0.947368421	1.096491228

g3bp2	0.947089947	1.159468439
arhgef9b	0.947019868	0.677419355
zcchc11	0.946969697	0.902912621
igfbp5a	0.946969697	1.845360825
snrpa	0.946954813	1.033797217
dixdc1a	0.946902655	0.937172775
mccc2	0.946859903	1.287709497
trim63a	0.946808511	1.615686275
ccnt2b	0.946708464	1.026923077
tlk2	0.946666667	0.700854701
TLE2	0.946666667	0.72
timm44	0.946666667	1
pomgnt1	0.946564885	1.265306122
PKLR	0.946551724	0.600961538
plg	0.946502058	1.135518158
ela2	0.946474284	1.165929204
cct2	0.946466809	0.726887193
ttc38	0.946428571	0.675324675
kdm7aa	0.946428571	0.905405405
lpcat2	0.946428571	0.931818182
ndufa6	0.94638404	0.925637025
rab6bb	0.946351931	1.114379085
pou3f1	0.946261682	0.716981132
arhgap21a	0.946236559	0.865671642
SNX2	0.946236559	0.872093023
syne2b	0.946153846	1.054347826
adck4	0.946107784	1.620155039
adarb1a	0.945945946	0.452830189
tmem39a	0.945945946	0.71559633
dbf4	0.945945946	0.71875
trim65	0.945945946	0.730769231
kank3	0.945945946	0.984375
si:ch211-126c2.4	0.945945946	1
map6d1	0.945945946	1.208333333
CABZ01084570.1	0.945945946	1.266666667
CLINT1 (1 of many)	0.945945946	1.338235294
pcdh1gb2	0.945945946	1.565217391
si:dkey-261j15.2	0.945945946	1.727272727
exoc3l4	0.945945946	2.625
atp1b3a	0.945921986	1.082331175
cyt1	0.945850455	0.975040047
lig3	0.945812808	1.026845638
slc10a3	0.945783133	0.679856115
syt11a	0.945736434	0.720797721
LZTS3 (1 of many)	0.945652174	0.37804878
meis1a	0.945652174	0.867816092
dtx2	0.945652174	1
ino80b	0.945544554	1.224489796
itga6a_2	0.945454545	0.5
raly	0.945454545	0.714285714
zgc:64201	0.945454545	0.962264151
scfd2	0.945454545	0.977272727
ccdc84	0.945454545	1.930232558
pnisr	0.9453125	0.965156794
ocrl	0.9453125	0.967391304
c8b	0.945255474	0.812080537
ddb2	0.945244957	1.464968153
pdha1a	0.945211581	1.105039788
mfsd1	0.945205479	0.784090909
ptpn3	0.945205479	1.024390244
elmsan1a	0.945205479	1.03030303

utrn	0.945205479	0.869109948
cyp2ad3	0.945188794	1.494652406
ppp1cab	0.945165945	1.041450777
wasf2	0.94516129	1.019169329
igf2b	0.945121951	1.585271318
scg5_2	0.945121951	1
ndufs2	0.945103206	1.129701061
si:dkey-103g5.3	0.945098039	1.663316583
gnao1b	0.945080092	0.643500644
prpf4	0.945054945	0.801932367
chrna5	0.945054945	0.806818182
acy3.2	0.945054945	0.866666667
fam46c	0.945054945	1.263513514
cers2b	0.945054945	1.3
impa1	0.945017182	1.277310924
cdh11	0.944990177	0.767981439
ddx23	0.944976077	0.894736842
abhd17c	0.944954128	0.637254902
gpr1	0.944954128	0.910891089
si:dkeyp-118b1.2	0.944954128	1.14556962
FAM83G	0.944954128	1.157303371
gnal	0.944954128	1.5
ppp2r5ea	0.944927536	1.067164179
ndnf	0.944897959	0.888382688
foxf1	0.94488189	1.518518519
lrrtm2	0.944827586	0.735294118
si:ch73-95l15.5	0.944827586	1.228813559
ppwd1	0.944827586	1.231481481
srsf3b	0.944792974	0.784163474
smyhc2	0.944751381	1.193877551
spag7	0.944751381	1.827868852
RNF157	0.944700461	1.042553191
mprip	0.944664032	0.678217822
ier5l	0.944656489	0.781007752
camk2g2	0.944578313	0.776315789
fem1a	0.944444444	0.624309392
cldn15a	0.944444444	1.061728395
cyp3a65	0.944444444	1.466216216
cntnap5l	0.944444444	0.387096774
znf385d	0.944444444	0.409090909
mybl2b	0.944444444	0.6
fbxl7	0.944444444	0.682926829
lgr4	0.944444444	0.72173913
si:ch211-80j13.1	0.944444444	0.777777778
hid1b	0.944444444	0.793103448
faap24	0.944444444	0.823529412
exo1	0.944444444	0.882352941
znf76	0.944444444	0.9
SELENOI	0.944444444	0.931818182
arl16	0.944444444	1.083333333
vetz	0.944444444	1.107142857
DHX35	0.944444444	1.15625
sirt3	0.944444444	1.216666667
si:ch211-227m13.1	0.944444444	1.3125
bbs7	0.944444444	1.432835821
ikbke	0.944444444	2.130434783
raph1a	0.944444444	0.592592593
xkr6b	0.944444444	0.705882353
ranbp3a	0.944444444	1.266666667
si:dkeyp-100a1.6	0.944444444	1.279069767
grapa	0.944444444	1.8

mrpl4	0.944353519	0.968847352
hnrnpl2	0.944300518	0.723684211
prpsap2	0.944250871	1.306451613
lsm8	0.944206009	0.889084507
polr3h	0.944206009	1.131004367
ppp1cbl	0.944190302	0.828003457
seta	0.944145635	1.057319588
ssr4	0.944104803	1.021360759
cdh13	0.944	0.385542169
PDE4DIP	0.944	1.152542373
usp48	0.943925234	1.180722892
tmem199	0.943925234	1.2
anapc2	0.943820225	0.829268293
tnfsf10	0.943820225	0.901287554
polr2j	0.943734015	1.355029586
tiam1a	0.943661972	0.836206897
trmt2a	0.943661972	1.064516129
ormdl1	0.943661972	1.44973545
sgca	0.943573668	1.060402685
CU928117.1	0.943548387	0.831325301
pnpla6	0.943548387	1.328767123
atxn2l	0.943478261	0.643617021
CABZ01073167.1	0.943396226	0.56
sephs1	0.943396226	0.783185841
tmem169a	0.943396226	0.8
sh3pxd2ab	0.943396226	0.977272727
mmp28	0.943396226	1.24
MFF (1 of many)	0.943396226	1.313868613
ift22	0.943396226	1.733333333
ammecr1	0.943333333	0.896860987
ppp1cb	0.943313953	1.037891269
fah	0.943283582	1.54
dvl3a	0.943262411	0.823529412
ntng2a	0.943181818	0.928571429
mios	0.943181818	0.931506849
slc12a2_2	0.943143813	1.004366812
fgf14	0.943089431	0.392156863
adgrl2a	0.943089431	0.49137931
neflb	0.943089431	0.641975309
s100s	0.943089431	1.054298643
olfm1a	0.943060498	0.797546012
tspy	0.943060498	1.115044248
pi4kb	0.943037975	0.73015873
hs3st3b1b	0.943037975	1.096491228
chl1b	0.942965779	0.588235294
tmem91	0.942857143	0.6
chtf18	0.942857143	0.612903226
galnt18b	0.942857143	0.8
slc7a8a	0.942857143	0.823404255
rab38c	0.942857143	0.88
tmem179	0.942857143	0.912162162
unc5b	0.942857143	1.090909091
FEZ2 (1 of many)_1	0.942857143	1.176470588
cox5b2	0.942857143	1.304123711
arhgef19	0.942857143	1.375
VMAC	0.942857143	1.470588235
zgc:113090	0.942857143	1.65
chchd7	0.942731278	1.634020619
dedd	0.942675159	0.900826446
si:dkey-111e8.1	0.942622951	0.776699029
sorbs1	0.942622951	1.206030151

TMEM27	0.942622951	1.989795918
wdr61	0.942583732	0.916666667
pfdn1	0.942567568	1.29588015
atrx	0.942528736	1.017766497
nup205	0.942408377	0.592178771
cdc42ep4b	0.942386831	1.031674208
sumo3a	0.942340792	0.889344262
calm1b	0.942315971	1.028248588
itpr1b	0.942307692	0.5
usp54a	0.942307692	0.588235294
gli2b	0.942307692	0.616666667
ints5	0.942307692	0.770833333
psmd11b	0.942307692	1.09178744
adamts15a	0.942307692	1.631578947
znf1014	0.942307692	1.739130435
cdkn1bb	0.942279942	1.035775128
agpat2	0.942196532	1.111111111
ubfd1	0.942196532	1.291390728
ppig	0.942176871	0.871428571
cd151l	0.94214876	1.31152648
cadm4	0.942115768	0.521857923
ctbp2a	0.942028986	0.505747126
ssbp1	0.942028986	1.36
zfand4	0.942028986	0.53968254
lin9	0.942028986	1.480769231
urod	0.942028986	1.53125
KRT86	0.942028986	2.180392157
znf536	0.941935484	0.358333333
ccdc92	0.941935484	0.548148148
wdfy2	0.941908714	1.154285714
kif5c	0.941860465	0.615384615
sort1b	0.941860465	0.856
si:ch211-67e16.11	0.941860465	1.104477612
rhot1a	0.941828255	1.220883534
mycb	0.941818182	0.547058824
rae1	0.941747573	1.25
atp6v1e1b	0.941730934	1.245614035
si:dkeyp-116a7.2	0.941724942	1.546712803
psd3l	0.941704036	0.771604938
cdc42ep4a	0.941666667	1.244680851
slc35b3	0.941666667	1.351648352
krtcap2	0.941666667	1.032258065
pros1	0.941605839	0.820143885
lsm1	0.941605839	1.336206897
si:ch1073-44g3.1	0.941580756	0.477178423
rif1	0.941558442	0.736363636
phax	0.941489362	0.932960894
pitx2	0.941441441	1.258883249
adh5	0.941390041	0.774193548
csk	0.941358025	0.724458204
emc7	0.941267388	1.501923077
PLPPR2 (1 of many)	0.941176471	0.416666667
si:dkeyp-113d7.1	0.941176471	0.664
CABZ01072157.1	0.941176471	0.722222222
tbc1d32	0.941176471	0.791666667
si:ch211-178n15.1	0.941176471	0.941176471
rnf41l	0.941176471	1
GABRG1	0.941176471	1.058823529
CR450780.5	0.941176471	1.071428571
atoh1a	0.941176471	1.136363636
snapc1b	0.941176471	1.320754717

hsh2d	0.941176471	1.4
tubd1	0.941176471	1.5
si:dkey-21c1.8	0.941176471	1.823529412
wbp1	0.941176471	0.818181818
ksr1b	0.941176471	0.933333333
vps13d	0.941176471	1
CELA1 (1 of many)_5	0.941176471	1.061320755
UBA6	0.941176471	1.072727273
LRRN3 (1 of many)	0.941176471	1.186666667
GAN	0.941176471	1.194444444
med4	0.941176471	1.232876712
sgf29	0.941176471	1.450704225
PAOX	0.941176471	1.704545455
lgals8a	0.941176471	1.9375
msrb1b	0.94100295	1.420849421
ndufs1	0.940990099	0.952960941
gkap1	0.940972222	0.866197183
max	0.940972222	0.951417004
naa15b	0.94095941	0.847222222
gpm6aa	0.940919037	0.633928571
nr2f1a	0.940850277	0.611111111
aida	0.940828402	1.641025641
tmem203	0.940789474	0.993939394
uqcrc2b	0.940789474	1.110497238
cfb	0.940754039	0.951040392
nisch	0.940740741	0.941747573
tmem147	0.940700809	1.048780488
pigq	0.940677966	1.063291139
cipcb	0.940639269	1.033057851
diaph2	0.940594059	0.647727273
adcy6a	0.940594059	0.841269841
capn8	0.940594059	0.965517241
slc4a7	0.940594059	0.989361702
SHKBP1	0.940594059	1.016393443
zfyve21	0.940540541	1.175141243
zgc:56493	0.940524781	1.043103448
prmt1	0.940504808	1.091715976
abhd16a	0.940397351	0.943319838
TULP3	0.940397351	1.13253012
rogdi	0.940334129	1.495702006
stard13a	0.940298507	0.627118644
wdhd1_1	0.940298507	0.633333333
lrch4	0.940298507	0.731707317
fgf10a	0.940298507	0.968253968
CREB3L3 (1 of many)	0.940298507	1.242424242
mark3b	0.940298507	0.803797468
memo1	0.940298507	0.963855422
tomm20b	0.940229885	0.963855422
nptxra	0.940217391	0.584745763
fuca1.2	0.94017094	1.310344828
serpine2	0.940149626	0.959349593
abhd14b	0.94011976	0.990291262
mboat7	0.94011976	1.375
nmt2	0.940074906	1.106598985
cfl1l	0.940069822	1.111173603
tmed3	0.940063091	1.642156863
zgc:153704	0.940029985	0.915134371
zgc:85936	0.94	0.755102041
CABZ01079490.1	0.94	1.105263158
ern2	0.94	1.205882353
pmchl	0.94	1.442307692

cdk13	0.94	0.612676056
ptpn11a	0.94	0.926605505
aifm1	0.939958592	1.128865979
got2b	0.93986637	1.162531646
ubiad1	0.939849624	0.905349794
si:ch211-196l7.4	0.939849624	1.376271186
map7d1a	0.939814815	0.970760234
ints10	0.939759036	0.771428571
HDAC7	0.939759036	0.888888889
noc4l	0.939759036	1.661764706
setd6	0.939759036	1.724137931
mapk8b	0.939655172	0.658227848
camkk1b	0.939655172	0.888888889
myzap	0.939655172	1.533980583
vimp	0.939655172	1.605797101
ndnfl	0.939597315	0.786407767
BORCS6	0.939597315	0.876106195
myoz3a	0.93956044	0.836653386
rab11fip4a	0.939516129	0.7875
mast3b	0.939393939	0.678571429
myo10l3	0.939393939	0.729166667
cbln14	0.939393939	0.825
si:ch73-138e16.4	0.939393939	1
znf507	0.939393939	1.130434783
ankrd1a	0.939393939	1.258064516
mitd1	0.939393939	1.291666667
iffo2a	0.939393939	1.823529412
si:dkey-16m19.6	0.939393939	2
tomm20a_1	0.939341421	1.160958904
mtx2	0.939334638	1.031434185
pvalb5	0.939334638	1.150793651
kars	0.939277479	0.951048951
ptpn1	0.939252336	0.956756757
nudt4b	0.939226519	0.647959184
ttll4	0.939226519	0.84
ccnyl1	0.939226519	1.057971014
ccdc9	0.939226519	1.069767442
erbb2ip	0.939189189	0.775700935
ppap2d	0.939153439	1.329824561
cmss1	0.939130435	1.466666667
cldnh	0.939058172	1.511187608
hepacama	0.93902439	0.550387597
msh6	0.93902439	0.642384106
ampd3b	0.93902439	0.895238095
ANKRD50	0.93902439	1.02173913
gtf2h5	0.93902439	1.7125
crybgx	0.938983051	0.741097923
ap1s1	0.938931298	1.460606061
chd7	0.938864629	0.557894737
scfd1	0.93877551	1.23297491
slc22a2	0.93877551	1.303448276
si:dkey-206f10.1	0.93877551	0.441176471
znf804a	0.93877551	0.473684211
sh2d3ca	0.93877551	0.74137931
fam63b	0.93877551	0.8
zgc:114046	0.93877551	0.901098901
dclre1a	0.93877551	1.342857143
fgf20a	0.93877551	1.68
kcnk1a	0.93877551	2.461538462
stx12	0.938730853	1.069364162
nid1b	0.938718663	0.807228916

st14a	0.938718663	1.12455516
dnajc5ga	0.938697318	1.123287671
eml1	0.938650307	0.68852459
thop1	0.938596491	0.825396825
sgms1	0.938596491	0.98
tmem106bb	0.938596491	1.838565022
tex10	0.938547486	0.850931677
phkb	0.938496583	1.07523511
sms	0.938492063	0.859090909
igdcc4	0.938461538	0.580645161
map3k14b	0.938461538	1.657894737
gapdhs	0.938402008	0.730331809
gria4b	0.938356164	0.536842105
znf346	0.938333333	1.066513761
itgb6	0.938271605	0.95
nudt21	0.938271605	1.148083624
stmn4l	0.938235294	0.758308157
ppip5k1b	0.93814433	0.989130435
hoxa3a	0.93814433	1.142857143
cers2a	0.93814433	1.175675676
acbd3	0.938095238	0.86
zgc:162608	0.938095238	1.561904762
ilvbl	0.938053097	0.780612245
sp8b	0.938053097	0.810810811
fam65a	0.937984496	0.669172932
col2a1b	0.937832311	0.838078292
trnau1apb	0.937788018	1.042440318
atpv0e2	0.937759336	1.357976654
zmat2	0.937704918	1.761083744
eif3s6ip	0.937562438	0.977044476
proca	0.9375	0.816176471
oclnb	0.9375	1.666666667
myrip	0.9375	0.43902439
si:ch211-210c8.5	0.9375	0.54
rcor3	0.9375	0.6
ebf1b	0.9375	0.64
kctd16b	0.9375	0.666666667
stil	0.9375	0.714285714
fam155b	0.9375	0.720930233
pcolce2a	0.9375	0.736842105
gpr52	0.9375	0.828571429
evx1_1	0.9375	0.84
tns1a	0.9375	0.87037037
cacnb1	0.9375	0.919642857
bora	0.9375	1
anapc5	0.9375	1
wipf2a	0.9375	1
ints6	0.9375	1.048780488
zgc:193681	0.9375	1.071428571
zgc:152938	0.9375	1.096774194
slc16a4	0.9375	1.131782946
map1sa	0.9375	1.163934426
si:ch211-147a11.3	0.9375	1.266666667
klhl41a	0.9375	1.338983051
si:dkey-4c15.14	0.9375	1.357142857
si:ch211-245n8.4	0.9375	1.36
zbtb5	0.9375	1.425
si:ch73-352p4.5	0.9375	1.64
si:dkey-269o24.1	0.9375	1.666666667
stk3	0.9375	1.832369942
sbds	0.9375	2.25974026

gpia	0.9373297	0.76317249
si:ch73-269m14.2	0.93728223	1.172093023
dhrs12	0.93728223	1.861244019
hiat1a	0.937106918	1.299145299
gabpa	0.937062937	0.681528662
btbd10a	0.937062937	0.824034335
wdr18	0.937062937	1.37007874
lrba	0.937007874	0.987804878
ctdsp1	0.936936937	1.292682927
hnrpl	0.936914428	1.012746973
ndufv3	0.936883629	1.073741007
sdcbp	0.936842105	1.648854962
abcc2	0.936794582	1.155063291
si:dkey-276j7.1	0.936788875	1.082142857
gpc1b	0.936781609	1.060606061
bckdk	0.936758893	0.989154013
egln1b_1	0.936758893	1.050925926
ntng1a	0.936708861	0.25
iqsec2	0.936708861	0.584615385
chchd2	0.936708861	1.042649728
fam107b	0.936666667	1.37109375
rps6kb1a	0.936651584	0.987124464
aimp2	0.936627282	0.831384016
lrp5	0.936619718	0.852459016
CABZ01050249.1	0.936619718	1.034482759
dclk2a	0.936507937	0.563636364
im:7160594	0.936507937	0.71942446
BAZ1A	0.936507937	0.782178218
RHOBTB3	0.936507937	0.906976744
zgc:162613	0.936507937	0.911764706
rbl1	0.936507937	0.923076923
tmem54a	0.936507937	1.209677419
dpf2l	0.936454849	0.696498054
lin7c	0.936454849	1.551219512
neurod1	0.936408978	1.014678899
coq2	0.936363636	0.901639344
rexo4	0.936363636	1.516483516
capn1a	0.936363636	1.039735099
ttc39c	0.936305732	0.963636364
si:dkey-6n6.2	0.936305732	1.126984127
dpysl2b	0.936283186	0.36
zgc:85975	0.936189608	1.03205699
slc25a23b	0.936170213	0.566666667
ZBTB39	0.936170213	0.575757576
phf2	0.936170213	0.697674419
si:ch211-152c8.5	0.936170213	1.230769231
abt1	0.936170213	1.68852459
acbd5a	0.936170213	1.846153846
si:ch211-132b12.7	0.936046512	1.294605809
rftn2	0.936	0.637931034
pkib	0.935960591	1.593548387
macrod2	0.93594306	1.020491803
her15.2	0.935897436	0.915789474
cdc34b	0.935897436	1.154727794
nadka_1	0.935897436	1.843137255
u2surp	0.935866983	1.037974684
srgap2	0.935828877	0.764285714
sh3bp4a	0.935779817	0.693069307
exosc2	0.935779817	0.898648649
dmd	0.935779817	0.922330097
cldn12	0.935779817	1.288461538

eif2a	0.935691318	0.779850746
pole3	0.935691318	1.142857143
prr33	0.935534591	1.283858998
AC024175.16	0.935526316	0.92626132
crygm2d12	0.935488677	1.029615278
mrpl22	0.935483871	1.017910448
ccnjl	0.935483871	0.573770492
lrp3	0.935483871	0.72
zgc:162595	0.935483871	0.769230769
si:dkey-57i11.4p	0.935483871	0.80952381
purg	0.935483871	0.906976744
si:ch1073-104i17.1	0.935483871	0.962962963
slc8a3	0.935483871	1.083333333
dchs1a	0.935483871	1.095238095
zbtb25	0.935483871	1.166666667
adam9	0.935483871	1.177083333
lyrm1	0.935483871	1.5
si:dkey-11f4.16	0.935483871	1.545454545
CABZ01040556.1	0.935483871	1.722222222
si:ch211-202f5.3	0.935483871	1.782608696
dctn1a	0.935483871	1.033333333
naa15a	0.935281837	0.829326923
zgc:162967	0.935251799	1.333333333
hip1rb	0.935185185	0.965909091
si:ch73-103b11.2	0.935185185	1
bri3	0.935064935	0.954154728
gmppb	0.935028249	0.96835443
ilf3b	0.935007386	0.611211573
arid4a_1	0.93495935	0.73
usp24	0.93495935	0.866071429
colgalt2	0.93495935	0.941176471
PDPR	0.93495935	1.004926108
TSHZ2	0.934911243	0.736842105
eef1a1b	0.934911243	1.37414966
srsf4	0.93487699	0.982078853
tmem63ba	0.9348659	1.079439252
ndufb4	0.934809348	0.927232635
arl6ip1	0.934794829	1.358882521
zgc:77816	0.934782609	0.893617021
fgl1	0.934782609	1.053571429
tnfrsfa	0.934782609	1.292517007
actn2b	0.934782609	1.456140351
brap	0.934782609	0.884892086
irbp	0.934715026	0.563535912
cyb5r3	0.934615385	1.375
slc30a9	0.934545455	1.188073394
myo6a	0.934482759	0.878504673
pitpnm3	0.93442623	0.612244898
heatr1	0.93442623	1.111111111
vcpip1	0.93442623	1.133333333
cryba1b	0.934389974	0.646890636
phc2a	0.934306569	0.730769231
ccdc82	0.934306569	0.837209302
prkcsh	0.934306569	1.166666667
rybpa	0.9342723	1.047337278
vcp	0.934242572	0.886533666
wwp2	0.934240363	1.065625
racgap1	0.934210526	0.951612903
zgc:162964	0.934210526	1.206349206
and1	0.934179894	1.016618657
ank3a	0.934131737	0.606557377

sae1	0.934097421	0.997076023
lgi2a	0.934065934	0.661290323
ppm1ab	0.934065934	1.118644068
aacs	0.934065934	1.628865979
pvalb9	0.934054054	0.799822064
snrpb	0.934	0.704162367
eea1	0.933962264	0.970149254
lsg1	0.933920705	1.184210526
znf106a	0.933908046	1.156862745
si:dkey-235h8.1	0.933884298	0.76969697
vac14	0.933884298	1.081395349
bbs5	0.933884298	1.175438596
ngrn	0.933884298	1.459459459
tfb2m	0.933884298	1.252808989
lpar6a	0.933823529	1.020833333
apoda.2	0.933781513	1.211275626
dlb	0.93373494	0.890510949
cpne1	0.933712121	1.777777778
mtss1la	0.933673469	0.75147929
rab6a	0.933649289	1.447058824
chga	0.933554817	1.122047244
myl9b	0.933402706	1.073149742
mta1	0.933333333	0.531531532
mettl14	0.933333333	0.539325843
si:ch211-247j9.1	0.933333333	0.6
nup155	0.933333333	0.738461538
OXR1 (1 of many)	0.933333333	0.781690141
snai1a	0.933333333	0.864583333
nol10	0.933333333	0.871212121
bicd2	0.933333333	0.897959184
TNC (1 of many)	0.933333333	0.908127208
tmem107	0.933333333	0.929824561
c3a.3	0.933333333	0.947712418
lamb1b	0.933333333	1.007692308
rasa1b	0.933333333	1.102564103
rgp1	0.933333333	1.153846154
tmem231	0.933333333	1.244897959
sap30bp	0.933333333	1.275555556
ugt2a4	0.933333333	1.365671642
hprt1l	0.933333333	1.396226415
grm2a	0.933333333	0.448979592
dab2ipa	0.933333333	0.510638298
DMWD	0.933333333	0.638888889
si:dkey-10f21.4	0.933333333	0.666666667
si:dkey-24p1.6_2	0.933333333	0.842105263
myo5c	0.933333333	0.846153846
rin2	0.933333333	0.925
eme1	0.933333333	0.966666667
grem2a	0.933333333	1.0625
hoxb8b	0.933333333	1.098039216
zgc:154061	0.933333333	1.142857143
stim2b	0.933333333	1.19047619
si:dkey-182g1.10	0.933333333	1.266666667
si:dkey-18j18.3	0.933333333	1.384615385
zgc:162150	0.933333333	1.545454545
pcgf5b	0.933333333	1.7
ccdc15	0.933333333	2
si:ch211-212i14.2	0.933333333	2.666666667
c9	0.93324857	0.717424242
cd63	0.933169533	0.967714528
si:ch211-114c12.2	0.933130699	0.760135135

prpf38b	0.933070866	0.830985915
bzw1a	0.933057281	1.179093005
cbln1	0.932989691	0.808695652
cpt1b	0.932960894	1.269461078
eif4bb	0.932907348	0.831325301
myo18ab_1	0.932885906	0.941176471
pqlc2	0.932835821	1.192307692
c3a.1	0.932773109	1.089425124
pla2g12b	0.932719954	1.199138344
apc2	0.932692308	0.358490566
dis3	0.932692308	1.576271186
syt5b	0.932678822	0.933333333
pold1	0.932642487	0.69005848
smc4	0.93258427	0.616766467
nt5c2l1	0.93258427	0.969072165
ncapd3	0.93258427	0.986666667
zgc:174888	0.93258427	1.382352941
si:ch211-196g2.4	0.93258427	1.645833333
senp6a	0.932515337	0.7
sptssa	0.932489451	1.139013453
glrba	0.932432432	0.897058824
SPAG9 (1 of many)	0.932432432	0.975806452
ehd4	0.932432432	1.136986301
clptm1	0.932384342	0.876957494
aldh1a2	0.932352941	0.894160584
trim8a	0.932330827	0.653225806
bcs1l	0.932330827	0.952380952
hrasb	0.932330827	1.06779661
adssl	0.93231441	0.625
rs1a	0.932288401	1.014164306
nlgn2b	0.93220339	0.315789474
sos1	0.93220339	0.703703704
kctd12.1	0.93220339	0.734375
FAM171A2 (1 of many)	0.93220339	0.868852459
cbr4	0.93220339	0.894736842
atoh8	0.93220339	1.080645161
keap1a	0.93220339	1.395348837
ccna2	0.932	0.737254902
EIF1B	0.931997571	1.102016607
exoc1	0.931924883	1.36
gpr153	0.931818182	0.361111111
npas1	0.931818182	0.517241379
nrn1la	0.931818182	0.6
mms22l	0.931818182	0.625
arhgef10lb	0.931818182	0.666666667
slc25a25b	0.931818182	0.848484848
znf668	0.931818182	0.871794872
ezh1	0.931818182	0.914285714
arl13a	0.931818182	0.918918919
cers5	0.931818182	0.93220339
p2rx4a	0.931818182	1.243243243
exo5	0.931818182	1.527777778
tceb2_3	0.931740614	1.271818787
ip6k2a	0.931736527	0.967611336
KIF2A	0.931726908	1.051428571
kctd6b	0.931726908	1.149038462
zmym2	0.931677019	0.595744681
sra1	0.931677019	1.177514793
bcas2	0.931649331	1.391382406
mast2	0.931623932	0.521276596
anxa13	0.931558935	1.327906977

arpc2	0.931529542	1.0625
mrpl18	0.931506849	1.067610063
map4k2	0.931506849	1.12
zgc:173593	0.931506849	1.229508197
prkrirb	0.931506849	1.621212121
ddx42	0.931451613	0.963541667
FO904869.1	0.931372549	0.192307692
zbtb8b	0.931372549	0.974358974
pef1	0.931372549	1.25
ptprz1b	0.931330472	0.488095238
baz2a	0.931330472	0.63803681
spg7	0.931318681	1.496
cdnf	0.93129771	0.834319527
cttnbp2nlb	0.93129771	1.538461538
atp6v0cb	0.931288635	0.770591487
sarnp	0.931283906	1.418972332
suclg1	0.931242461	0.898709315
emc3	0.931232092	0.987261146
si:ch211-151p13.8	0.931216931	1.441471572
cand1	0.931098696	1.010416667
ctnna1	0.931076179	0.936231884
rims1b	0.931034483	0.52173913
trpm2_2	0.931034483	0.523809524
ppp2r2cb	0.931034483	0.641791045
sipa1l1	0.931034483	0.65625
zgc:172270	0.931034483	0.830769231
PHLPP2	0.931034483	0.923076923
si:ch211-155e24.3	0.931034483	0.97752809
sema4ba	0.931034483	1
nr2c2	0.931034483	1.010638298
si:ch211-67e16.4	0.931034483	1.020408163
vps26bl	0.931034483	1.154929577
shq1	0.931034483	1.25
tmem243a	0.931034483	1.257142857
prdm8	0.931034483	1.272727273
mtrr	0.931034483	1.476190476
zgc:153372	0.931034483	1.533333333
tmem154	0.931034483	1.744680851
mfsd11	0.931034483	1.80952381
si:ch211-140b10.6	0.931034483	1.862068966
klhl13_2	0.931034483	0.571428571
snx7	0.931034483	1.183673469
grinab	0.930902111	1.053164557
si:dkey-21e2.16	0.930851064	0.753012048
si:ch211-197h24.6	0.930851064	1.005882353
rps6kc1	0.930735931	1.161073826
crebzf	0.930735931	1.218604651
polr2eb	0.930715935	0.969171484
rnf44	0.930693069	0.615384615
fam120c	0.930693069	0.81097561
lamb2	0.930693069	0.88358209
CT027611.1	0.930693069	1.203703704
cox7a3	0.930588235	0.941110488
CAPN15	0.930555556	0.529411765
wipi1	0.930555556	0.943005181
acap3a	0.930555556	0.958333333
ube2na	0.930530165	0.931924883
med31	0.930463576	1.116207951
edf1	0.930448223	0.978671837
naa25	0.930348259	1.087209302
col5a3b	0.930337079	0.996721311

ubtfl	0.930313589	1.009174312
nfyal	0.930232558	0.953488372
mfn1b_2	0.930232558	1.192118227
cep68	0.930232558	1.321428571
si:dkey-54n8.2	0.930232558	1.473684211
si:dkey-165n16.1	0.930232558	1.5125
idh3g	0.930188679	1.412470024
mrps35	0.930184805	1.014953271
srsf7a	0.930167598	0.626388889
kat8	0.930107527	1.47761194
sdprb	0.930087391	1.166666667
tmcc1b	0.93006993	1.007692308
alyref	0.930009588	0.590866729
rxrgb	0.93	0.833333333
cldnk	0.929936306	0.725925926
slc4a8	0.929936306	0.945652174
ddost	0.929906542	1.013011152
tpd52l2b	0.929899857	1.220458554
asb13b	0.929824561	1.022727273
si:ch211-232m10.6	0.929824561	1.068965517
mfsd6b	0.929824561	1.163265306
mpeg1.1	0.929824561	1.412698413
bcl11ab	0.92972973	0.796296296
apoa1b	0.929717933	1.141411971
pou3f2a	0.9296875	0.455445545
ugt1b1	0.9296875	1.217391304
cpsf3	0.929663609	1.095081967
alg5	0.929663609	1.228873239
myoz2b	0.92962963	1.916201117
DVL3 (1 of many)	0.929577465	0.860465116
cry1ba	0.929539295	1.029182879
gng13b	0.929496403	0.892592593
macf1a	0.929487179	0.761061947
rrad	0.929487179	2.084745763
lman2	0.929467085	1.177263969
setb	0.929451764	0.810737034
fam118b	0.929411765	1.098591549
banf1	0.929404145	1.476153245
uqcc2	0.929310345	1.433839479
pvrl2l	0.929292929	1.771428571
eif4a3	0.929282869	0.75347544
col4a5	0.929270947	0.914511494
pmpca	0.92926045	1.09561753
tgfbi	0.929251138	0.906196213
rbp4	0.929211783	1.39523212
alox12	0.92920354	0.85840708
si:dkey-70p6.1	0.929133858	0.633928571
usp37	0.929133858	0.722222222
hdlbpa	0.929133858	0.979492188
zgpat	0.929133858	1.366336634
cpeb4	0.929032258	0.75
si:ch211-212d10.2	0.929032258	1.398550725
nenf	0.928994083	1.593984962
rnaseh2a	0.928870293	0.701438849
noc2l	0.928870293	1.425438596
adipor1b	0.928853755	0.931914894
itm2ba	0.92881588	1.275483304
ppp2r5eb	0.928753181	1.326241135
mid1ip1l	0.928730512	1.226012793
ARF5	0.928665786	1.063540091
si:ch211-132b12.2	0.928571429	0.323529412

pafah1b3_1	0.928571429	0.416666667
gpr19	0.928571429	0.564102564
pdgfbb	0.928571429	0.685714286
stx11a	0.928571429	0.708333333
mlxip	0.928571429	1.06122449
zgc:154125	0.928571429	1.153846154
zbtb47a	0.928571429	1.2
si:ch1073-186o8.3	0.928571429	1.55
tbc1d22b	0.928571429	1.6875
il15l_2	0.928571429	1.916666667
rnf215	0.928571429	2.36
slc25a20	0.928571429	0.7997558
traf2a	0.928571429	0.909090909
galnt2	0.928571429	0.965
ywhae1	0.928571429	0.988372093
calb2a	0.928571429	0.737005431
ppp1r3db	0.928571429	0.75
eepd1	0.928571429	0.773195876
dbr1	0.928571429	0.843971631
tbx20	0.928571429	0.971428571
il16	0.928571429	1
usf3	0.928571429	1.038461538
cep135	0.928571429	1.2
si:rp71-1c23.3	0.928571429	1.27753304
gnl3l	0.928571429	1.376033058
tomm34	0.928571429	1.380434783
fgf4	0.928571429	1.583333333
plekhb2	0.928571429	1.687022901
znf592	0.928571429	1.9
tnni2b.1	0.928525016	1.153691275
c1qbp	0.928515318	0.860058309
atp1a2a	0.928446771	1.379591837
gmps	0.928327645	0.890243902
clpxb	0.928327645	1.103942652
vps4b	0.928301887	1.511056511
golga7	0.928294574	1.122641509
epdl1	0.928286853	1.282142857
clip2	0.928229665	0.752
ywhah	0.928191489	0.511111111
acss2	0.928191489	1.034985423
mob3a	0.928143713	1.106666667
tpst1l	0.928104575	0.736842105
pdia6	0.928015564	1.112903226
ptprfb	0.927977839	0.583333333
psd2	0.927835052	0.625
khdrbs2	0.927835052	0.953216374
azin1b	0.927835052	1.084915341
drap1	0.927741935	0.818300654
si:ch211-160j14.3	0.927710843	1.112676056
her12	0.927710843	1.392405063
si:ch1073-440b2.1	0.927710843	1.743083004
mat2aa	0.927694407	0.968796434
slit2	0.927631579	0.690909091
txnrd1	0.927607362	0.93990755
tpcn1	0.927536232	0.836065574
jade1	0.927536232	0.990740741
hhatlb	0.927536232	1.214814815
zgc:193742	0.927536232	1.309859155
gdf6a	0.927536232	1.382978723
atl3	0.927419355	1.289340102
ext1c	0.927374302	0.648351648

dyrk1aa	0.927374302	0.717948718
wdr36	0.927374302	1.100719424
pip4k2ca	0.927360775	0.840659341
sh3kbp1	0.927272727	0.822695035
enpp5	0.927272727	1.317460317
ppt2	0.927272727	1.43902439
pias4a	0.927272727	0.695402299
eva1ba	0.927272727	0.855555556
si:ch211-156j22.4	0.927272727	0.888888889
men1	0.927272727	0.924731183
tcaim	0.927272727	1.175438596
hsd3b7	0.927272727	1.293172691
si:dkey-119m7.4	0.927272727	1.543478261
tpm3	0.927217332	1.046011255
thbs1b	0.927152318	1.137809187
pdia5	0.927125506	0.854748603
ttyh2l	0.927083333	0.603960396
mpp3a	0.927083333	0.718309859
smc5	0.927083333	1.323076923
arl2bp	0.927083333	1.836065574
ndufa12	0.927007299	1.093461538
klf5l	0.926966292	0.734104046
ola1	0.926931106	1.06300578
bet1	0.926910299	1.308108108
zgc:172246	0.926879506	0.822831727
col15a1b	0.926829268	0.594594595
kctd15a	0.926829268	0.623376623
snrnp40	0.926829268	0.685185185
atp5g3a	0.926829268	1.028532142
tagln3a	0.926829268	1.176470588
syt8	0.926829268	1.236842105
fut11	0.926829268	1.675675676
doc2b	0.926829268	0.516129032
bmpr2a	0.926829268	0.696629213
stx3a	0.926829268	1.115384615
pdap1a	0.926780341	1.33490566
c3b.1	0.926760563	0.936599424
dhx9	0.926760563	0.979757085
nr1h3	0.926666667	1.23655914
nap1l1	0.926647327	0.906741004
afp4_1	0.926605505	0.935064935
mapk14b	0.926605505	1.166666667
PPP2CA	0.926573427	0.815068493
ywhaqa	0.926535528	0.994967978
hnrpkl	0.926499033	0.847727273
gabrr2a	0.926470588	0.697674419
echdc2	0.926470588	0.74137931
llgl2	0.926470588	0.884848485
wu:fj39g12	0.926470588	0.9
si:dkey-181i3.8	0.926470588	0.93220339
si:dkey-21c1.1	0.926470588	1.4
bod1l1	0.926315789	0.710526316
pgls	0.926315789	1.050632911
dapk3	0.926289926	1.394495413
eif5b	0.926267281	0.873529412
gskip	0.926267281	1.060538117
khdrbs1b	0.926251097	0.892553191
myo9aa	0.926229508	0.887640449
ca8	0.926229508	1.06185567
mrpl12	0.926216641	1.114726027
BX649250.1	0.926174497	0.893939394

rock2a	0.926174497	0.933333333
pdcd10b	0.926153846	1.865612648
slc35b1	0.926086957	0.906403941
acadvl	0.926083866	1.096088435
fxr1	0.926065163	0.891381346
tnrc18	0.925925926	0.754716981
si:ch211-284e20.8	0.925925926	0.757834758
carf	0.925925926	0.805970149
ttc7b	0.925925926	0.874015748
nlk1	0.925925926	0.880952381
decr2	0.925925926	1.024390244
brcc3	0.925925926	1.071428571
rab40b	0.925925926	1.484126984
dtd2	0.925925926	1.703703704
arsg	0.925925926	0.673913043
pias1a	0.925925926	0.714285714
tert	0.925925926	0.776119403
si:ch211-214b16.4	0.925925926	0.964912281
si:ch211-218c6.9	0.925925926	1.129032258
nudt6	0.925925926	1.227272727
nrm	0.925925926	1.361111111
havcr1	0.925925926	1.387096774
rtkn2b_1	0.925925926	1.428571429
ano9a_1	0.925925926	1.619047619
bckdhb	0.925764192	1.027149321
clta	0.925684485	1.079008521
dnaja1	0.925675676	1.007874016
stk17a	0.925619835	0.745098039
npm3	0.925581395	1.099056604
frmd8	0.925581395	1.373333333
sybu	0.925531915	0.8
MMP15 (1 of many)	0.925531915	0.867647059
utp18	0.925531915	0.875
gtf2ird1	0.925531915	1.089285714
yrdc	0.925531915	1.146067416
blvra	0.925531915	1.4375
zdhhc20b	0.925531915	1.571428571
hexb	0.925507901	0.975935829
spon1b	0.925480769	0.614084507
rad23aa	0.925373134	1.038793103
enosf1	0.925373134	1.547619048
si:ch1073-340i21.3	0.925311203	1.122302158
btbd6b	0.925287356	1.01179941
soul2	0.925287356	2.042801556
bcap29	0.925233645	1.177570093
lgals3a	0.925219941	0.846270928
lpp	0.925170068	1.027027027
mlst8	0.925170068	1.2421875
acin1b	0.92513369	0.958041958
eif3s10	0.925104022	0.976190476
zbtb40	0.925	0.947368421
si:dkey-154p10.3	0.925	1.34375
cep70	0.925	1.433333333
uacab	0.924836601	1.452173913
si:dkey-6n21.12	0.92481203	1.341176471
adam10a	0.924778761	1.320150659
usp39	0.924778761	0.822510823
krt18	0.924747174	0.893078759
usp6nl	0.924731183	0.658227848
spc24	0.924731183	0.699029126
abhd17ab	0.924731183	1.140625

hnrnph3	0.924731183	1.835820896
impdh2	0.924705882	1.203196347
akirin2	0.924675325	0.792682927
epha4b	0.924657534	0.413043478
vopp1	0.924657534	0.615384615
zranb1b	0.924657534	0.903930131
hn1b	0.92454955	0.92450879
usp49	0.924528302	0.520833333
si:ch211-3o3.9	0.924528302	0.897435897
cdt1	0.924528302	0.904761905
epha3	0.924528302	1.058823529
necab1	0.924528302	1.882352941
krt4	0.924426106	0.940428827
bri3bp	0.924398625	0.977653631
phf6	0.924369748	1.132231405
calcoco1a	0.924369748	1.247252747
ttc9c	0.924242424	1.056603774
ssr2	0.924223074	0.994503171
angptl1a	0.924137931	1.025210084
sdc2	0.924092409	1.116480793
ttll1	0.924050633	0.93902439
nfe2l1b	0.924050633	1.14893617
zgc:136439	0.924050633	1.150684932
TIMM29	0.924050633	1.262443439
npc2	0.924034869	1.055664063
adcyap1r1a	0.923976608	0.390804598
dpp6a	0.923913043	0.367346939
plekha6	0.923913043	1.015151515
hsd17b7	0.923913043	1.275862069
ube2q2	0.923913043	0.910447761
CABZ01067945.1	0.923809524	0.415584416
cnot3b	0.923809524	0.846153846
cldn11b	0.923728814	0.855855856
per3	0.923664122	1.072625698
phkg1a	0.923664122	1.909090909
15-Sep	0.923625255	1.002347418
hnrnpa1b	0.923529412	0.915812592
mcfd2	0.923423423	1.597402597
si:ch211-51e12.7	0.923354373	0.655430712
fermt2	0.923287671	0.958333333
lamc1	0.923283984	0.926870748
marcksl1a	0.923099851	0.550662061
adam19a	0.923076923	0.645454545
ect2	0.923076923	0.454545455
trpm3	0.923076923	0.5
ano2	0.923076923	0.612903226
abi3b	0.923076923	0.803030303
smco4	0.923076923	0.996031746
cep290	0.923076923	1
chodl	0.923076923	1.015151515
si:dkey-100n10.2	0.923076923	1.041666667
rhot1b	0.923076923	1.192307692
tmbim4	0.923076923	1.354779412
coro2a	0.923076923	1.441176471
rpp30	0.923076923	1.516129032
HERC4	0.923076923	1.625
kcnd1	0.923076923	0.441176471
ELFN1 (1 of many)	0.923076923	0.621212121
kynu	0.923076923	0.698795181
ipo13	0.923076923	0.739726027
wtip	0.923076923	0.84

adnpa_1	0.923076923	0.918604651
si:ch1073-235i16.2	0.923076923	1
si:dkey-30c15.17	0.923076923	1
zgc:64065_1	0.923076923	1
map3k7cl	0.923076923	1.052631579
si:dkey-60a16.1	0.923076923	1.096153846
pimr65	0.923076923	1.7
si:ch211-171h4.3	0.923076923	1.75
osgepl1	0.923076923	1.789473684
ndrg3a	0.923026316	0.782020684
acsbg2	0.922907489	0.581333333
rbm4.1	0.922839506	0.889679715
larp4b	0.922651934	0.964788732
prosc	0.922606925	1.496062992
mboat2b	0.922535211	0.787037037
morc2	0.92248062	0.616666667
lonrf1	0.922465209	1.560655738
cflara	0.922413793	1.663636364
atxn2	0.922330097	0.776315789
tnpo2	0.922330097	1.069767442
gys1	0.922272048	1.182124789
pcdh11_2	0.922222222	0.650485437
gmds	0.922222222	0.988372093
smim8	0.922222222	1.121019108
napaa	0.922222222	1.644859813
vcla	0.922178988	0.953974895
scamp2	0.922178988	1
ptk2bb	0.922077922	0.901515152
waplb	0.922077922	0.920353982
wnt7aa	0.921985816	0.933884298
ch25hl1.1	0.921985816	1.020689655
strbp	0.92195122	0.738562092
znf598	0.921933086	1.044334975
zgc:113054	0.921914358	1.567099567
sp5a	0.921875	0.393939394
trip4	0.921875	0.968253968
patz1	0.921875	1.019230769
obfc1	0.921875	1.739130435
zgc:154054	0.921686747	1.22754491
mat2b	0.921658986	0.855072464
slc25a6	0.921658986	1.173076923
ccdc50	0.921568627	1
ripk1l	0.921568627	1.024390244
tmem51a	0.921568627	1.065217391
cacna1sa	0.921568627	1.240506329
trpc4apb	0.921568627	1.134228188
UBL4B	0.921568627	0.93956044
ube2ql1	0.921511628	0.386266094
TPGS1	0.921428571	0.705128205
ubtd1a	0.921428571	0.776223776
six3a	0.921428571	0.812182741
rxraa	0.921259843	0.690909091
hoxb6a	0.921259843	0.952830189
traf4a	0.921182266	0.794326241
timm50	0.921182266	1.058536585
soul4	0.921146953	0.678947368
CU914487.1	0.921052632	0.789473684
relt	0.921052632	0.933333333
cdk2	0.921052632	1.111111111
si:ch211-169p10.1	0.921052632	1.217391304
taf1b	0.921052632	1.235294118

f7i	0.921052632	0.763779528
fmo5	0.921052632	1.051502146
cnot2	0.920918367	1.135714286
decr1	0.920903955	1.145510836
antxr1c	0.920863309	1.065420561
col11a1a	0.920846128	0.712643678
tmem47	0.920833333	0.961111111
si:dkey-12h9.6	0.920792079	0.848484848
igf1ra	0.920792079	0.921875
hhip	0.920754717	0.960199005
dlx4a	0.920731707	0.96031746
nlgn4a	0.920634921	0.632911392
plekha7a	0.920634921	0.847457627
zgc:174877	0.920634921	0.936170213
ZNF384 (1 of many)	0.920634921	0.940594059
stxbp2	0.920634921	1.17
TULP2	0.920634921	1.2
arcn1b	0.920634921	1.324200913
slc38a7	0.920634921	1.625
copb1	0.920592194	1.142123288
oaz1a	0.920578683	1.186104998
dyrk2	0.920577617	1
rps6kb1b	0.920560748	0.944751381
ppifb	0.920454545	0.993071594
vav2	0.920454545	0.65625
CABZ01011067.1	0.920454545	0.838709677
si:dkey-11k2.7	0.920454545	1.661157025
zfp36l1a	0.920410783	1.073243647
spry2	0.920374707	0.81042654
disp2	0.920353982	1.029411765
socs2	0.920353982	1.033333333
jkamp	0.920289855	0.867768595
krt8	0.9201817	0.878095975
si:ch211-276i12.9	0.920127796	1.11641791
GALNTL6	0.92	0.447619048
chst11	0.92	0.666666667
sec23a	0.92	0.797546012
2-Mar	0.92	0.891304348
psenen	0.92	0.984269663
rab5b	0.92	1.58677686
zgc:110045	0.92	0.789473684
btbd7	0.92	0.837837838
nup188	0.92	0.845238095
myg1	0.92	0.903030303
cep350	0.92	0.929577465
wash1	0.92	0.973684211
tyk2	0.92	1.117647059
SLC9A3R2	0.92	1.121212121
si:ch211-137a8.2	0.92	1.235294118
prlrb	0.92	1.411764706
zgc:195023	0.92	1.479452055
rbm24a	0.919831224	1.333333333
btbd10b	0.919799499	0.914772727
lims1	0.919753086	0.846689895
lhfpl2b	0.919753086	1.067010309
s1pr1	0.919736842	0.7568
phkg1b	0.919708029	1.939759036
spock3	0.91967509	1.237513873
GABRA2 (1 of many)_2	0.919642857	0.289855072
yme1l1a	0.91959799	0.928104575
ralaa	0.91954023	0.774647887

ctdnep1b	0.91954023	0.974358974
fam19a5a	0.91954023	1.411764706
nom1	0.91954023	1.739130435
hdac1	0.91951952	1.039539899
Metazoa_SRP_1	0.919495635	0.812738854
clockb	0.919491525	1.109489051
adh8b	0.919477693	0.875232775
rnf139	0.919463087	0.677419355
axin2	0.919413919	0.970731707
si:ch211-95j8.2	0.919413919	1.602941176
mfsd10	0.919354839	1.58974359
panx1a	0.919254658	1.47972973
srrm2	0.919230769	0.68018018
celsr1a	0.919191919	0.928571429
krt5	0.919159164	1.022283908
ano5b	0.919117647	1.078947368
cib2	0.919117647	1.626373626
si:ch73-1a9.3	0.919089191	1.126821862
dscr3	0.918918919	0.894578313
si:dkey-228b2.5	0.918918919	1.181069959
angpt1	0.918918919	0.540540541
zbtb4	0.918918919	0.87037037
baiap2a	0.918918919	0.953846154
top3b	0.918918919	1
plcd3a	0.918918919	1
tgfbr1a	0.918918919	1.081081081
elfn2a	0.918918919	1.083333333
c6_2	0.918918919	1.096774194
snapc2	0.918918919	1.344827586
cryba1l2	0.918918919	1.35483871
rbmx2	0.918918919	1.396226415
mocs2	0.918918919	1.452830189
srp54	0.918803419	1.022727273
gpr37l1b	0.918699187	0.795180723
ruvbl1	0.918699187	1.19873817
atp11c	0.918604651	0.633333333
dohh	0.918604651	1.092814371
u2af2b	0.91858679	0.941729323
clpxa_2	0.918406072	0.894009217
ddx18	0.918367347	1.147058824
mtmr11	0.918367347	0.6
mipol1	0.918367347	0.930232558
galt	0.918367347	1.240740741
si:ch211-250o15.5	0.918367347	1.583333333
si:ch211-71k24.8	0.918224299	1.019323671
clip1a	0.918181818	1.25974026
dph5	0.918181818	1.447852761
snx19b	0.918181818	1.682539683
cd9b	0.918169209	0.982367758
pacsin2	0.918149466	1.028340081
alas1	0.918133803	0.731781377
si:ch211-5k11.2_1	0.918069948	0.555829228
obsl1a	0.918032787	1.372093023
ccl39.3_1	0.918032787	2.175
si:ch1073-174d20.2	0.917874396	1.083870968
abhd4	0.917857143	1.050420168
KCNJ6	0.917808219	0.464285714
efr3ba	0.917808219	0.479166667
rgma	0.917808219	0.651315789
atad2	0.917808219	0.71641791
chfr	0.917808219	1.06557377

rhot2	0.917808219	1.467391304
add2	0.917647059	0.486725664
hdac8_1	0.917647059	0.612903226
fynb	0.917647059	0.686567164
fndc7b	0.917647059	1.46
sypa	0.917549168	0.443037975
spns1	0.917547569	0.982570806
tspan7b	0.917525773	1.011703511
strn	0.917525773	1.076923077
ctnnal1	0.917525773	1.323943662
cirbpb	0.91744509	0.919572858
CU861477.1	0.917431193	0.463768116
MNT (1 of many)	0.917431193	0.75
daam1b	0.917431193	0.908571429
slc37a1	0.917431193	1.674698795
rpf2	0.917391304	1.254658385
strumpellin	0.917355372	0.953488372
pitx3	0.917355372	1
ehd1a	0.917257683	0.860655738
ccdc22	0.917241379	0.884297521
ybey	0.917159763	0.721518987
si:ch211-217k17.7	0.917061611	1.274271845
phb	0.917030568	1.098010317
aif1l	0.916876574	1.185714286
rpn2	0.91687448	1.145146089
grip2a	0.916666667	0.545454545
pth2r	0.916666667	0.733333333
mllt4a	0.916666667	0.838709677
f13a1b	0.916666667	0.86
rasal3	0.916666667	0.923076923
CABZ01077370.1	0.916666667	0.928571429
kcnj11l	0.916666667	0.933333333
si:ch211-232h12.2	0.916666667	0.95
fam98a	0.916666667	0.966666667
heyl	0.916666667	1
zbtb48	0.916666667	1
tomm5	0.916666667	1.041926851
smyd4	0.916666667	1.090909091
baxa_2	0.916666667	1.178217822
inpp4ab	0.916666667	1.242424242
prkag2b	0.916666667	1.294117647
FO904953.1	0.916666667	1.294695481
thumpd3	0.916666667	1.333333333
si:dkeyp-69b9.4	0.916666667	1.647058824
fam189a2	0.916666667	0.660714286
tmem266	0.916666667	0.666666667
dpp9	0.916666667	0.793650794
st13	0.916666667	0.847547974
arl11	0.916666667	1
ndufa4l2a	0.916666667	1
csf1b	0.916666667	1.036363636
kif14	0.916666667	1.090909091
znrd1	0.916666667	1.23880597
pptc7a	0.916666667	1.251748252
ushbp1	0.916666667	1.266666667
alg6	0.916666667	1.266666667
si:ch73-14h1.2	0.916666667	1.288461538
si:dkeyp-117b11.3	0.916666667	1.391304348
trim101	0.91643454	1.079710145
rab3aa	0.91643454	1.47107438
si:ch211-11c15.3	0.91641791	0.943298969

pbx3b	0.916323731	0.601659751
zc3h14	0.916256158	1.094594595
syn2a	0.916208791	0.786245353
fpgs	0.916201117	0.813253012
alcamb	0.916201117	1.235042735
lamp2	0.916099773	1.180055402
slc4a2b	0.91588785	1.159090909
n4bp2l2	0.91588785	1.272727273
ceacam1	0.915873016	1.417310665
ift74	0.915841584	0.87804878
lmbr1l	0.915841584	1.233695652
ptprz1a	0.915789474	0.475409836
ncaph2	0.915789474	0.987804878
phgdh	0.915766739	0.709016393
cacng1a	0.915750916	1.173228346
sepp1a	0.915728867	1.28698095
sox5	0.915708812	0.693121693
ppp3ccb	0.915662651	0.849878935
hpcal1	0.915662651	0.933333333
rbm5	0.915662651	1.154867257
atp1a1b	0.915656299	0.688405797
cited4b	0.915567282	0.685015291
gnat2	0.915545992	0.840871022
F7 (1 of many)	0.915492958	0.739130435
si:ch211-243j20.2	0.915492958	0.939759036
nufip2	0.915492958	1.201923077
si:ch211-241e1.3	0.915492958	1.238095238
slc39a13	0.915492958	1.283018868
trit1	0.915492958	1.296296296
timm10b	0.915492958	1.448275862
mbpa	0.915413534	0.924406048
gtf2f2a	0.915360502	1.425702811
sh3glb2b	0.915322581	1.130208333
mrpl15	0.915294118	1.193820225
dennd2c	0.915254237	0.733333333
igf2r	0.915254237	0.780487805
im:7151449	0.915254237	0.884615385
csrp1a	0.915254237	1.108247423
traf3ip1	0.915254237	1.2
polr1c	0.915254237	1.25862069
rpz2	0.915254237	1.585635359
si:ch211-80h18.1	0.915141431	0.933206107
MAST3 (1 of many)	0.91509434	0.580645161
brwd1	0.91509434	0.876712329
CABZ01083501.2	0.91509434	0.914634146
zgc:110269	0.91509434	1.118181818
zgc:153981	0.91509434	2.058139535
mst1	0.914965986	0.60326087
si:ch211-173d10.1	0.914933837	1.770949721
crygmx	0.914901961	1.815737705
peli2	0.914893617	0.941666667
stmn3	0.914893617	0.785714286
rbms1a	0.914893617	0.841040462
plat	0.914893617	1.093023256
katna1	0.914893617	1.307692308
si:dkey-27p18.5	0.914634146	0.307692308
pex11b	0.914634146	0.855421687
slc3a2a	0.914581536	0.912667191
arfip2a	0.914572864	1.113333333
atxn7l3	0.914529915	1.48
prkrip1	0.914473684	1.547169811

COLQ (1 of many)	0.914414414	1.265306122
hspa5	0.914366596	0.632365145
arap3	0.914285714	0.828571429
irf5	0.914285714	0.882352941
tll1	0.914285714	1.047619048
ubash3bb	0.914285714	1.096774194
pemt	0.914285714	1.454545455
hrasls	0.914285714	1.760869565
kcnk5a	0.914285714	1.842105263
si:ch211-241d21.5_1	0.914285714	1.904761905
sec22c	0.914285714	2.25
gltscr2	0.914176732	1.455259027
snx9b	0.914141414	1.010989011
palm1b	0.9140625	0.932135729
bckdha	0.91403287	1.154458599
emid1	0.913978495	1.071684588
pdgfra	0.913907285	1.004166667
hcn1	0.913793103	0.472222222
gli2a	0.913793103	0.833333333
crebrf	0.913793103	0.86
pacsin3	0.913793103	0.947368421
ssx2ipa	0.913793103	0.982758621
ern1	0.913793103	1
abch1	0.913793103	1.159090909
cdc27	0.913705584	1.025
rela	0.913580247	1.075757576
zgc:91890	0.913580247	1.266666667
fadd	0.913580247	2.196721311
bzw2	0.913580247	1.27394636
prrt1	0.913513514	0.391891892
pkp4	0.913461538	0.530120482
otofb	0.913461538	1.252873563
slc6a17	0.913385827	0.4375
flot2a	0.913308913	0.88896648
gnat1	0.913242009	0.923161361
matn4	0.913180516	1.21399177
ppt1	0.913173653	1.411347518
id4	0.913140312	1.294403893
hnrnpc	0.913112164	1.055846422
mcm10	0.913043478	0.590909091
fam101a	0.913043478	0.702702703
rspo1	0.913043478	0.736842105
lrp1ba_1	0.913043478	0.769230769
b3galt1a	0.913043478	0.842105263
mtnr1bb	0.913043478	0.888888889
plcd4a	0.913043478	0.941176471
gig2n	0.913043478	1
twist1a	0.913043478	1.040650407
cntrl	0.913043478	1.071428571
si:ch211-195m9.3	0.913043478	1.086956522
cep83	0.913043478	1.162790698
znf1136	0.913043478	1.166666667
si:ch211-229n2.7	0.913043478	1.31372549
ABCF2 (1 of many)	0.913043478	1.333333333
iqcc	0.913043478	1.36
si:dkey-4i23.7	0.913043478	1.45
psme2	0.913043478	2.8
ssh1b	0.913043478	0.490196078
ptprb	0.913043478	0.898305085
mier1a	0.913043478	1.395348837
mtmr14	0.913043478	1.532608696

tspan37	0.913043478	1.586956522
flot1b	0.913017751	0.793851718
anxa1c	0.912969283	0.918067227
usp2a_2	0.912966252	0.948364888
col4a3bpb	0.912921348	1.396078431
osbpl2a	0.912891986	1.606451613
krt94	0.912873862	0.892086331
crygm2d14	0.912865388	1.04516129
pitrm1	0.912844037	1.142857143
fasn	0.912790698	0.657407407
urm1	0.912790698	0.983695652
pla2g7	0.912790698	1.837606838
ada	0.912708601	1.255681818
gigyf1	0.912698413	1.291925466
elovl1b	0.912681913	1.022988506
ell2	0.912663755	1.023584906
ywhabl	0.912623398	0.856178258
cdca8	0.9125	0.917647059
mbtd1	0.912442396	1.218045113
ndufa4	0.912431108	0.981997677
bola3	0.912408759	1.383333333
epc2	0.912371134	0.753246753
rcvrn2	0.912337662	1.114130435
cbsb	0.912317328	1.076502732
osbp	0.912280702	1.1171875
THEM6 (1 of many)_2	0.912280702	1.183006536
oxnad1	0.912280702	1.3125
tmf1	0.912195122	1.224637681
syt5a	0.912181303	1.04
atg16l1	0.912162162	0.884297521
med6	0.912133891	1.113924051
ppa1b	0.912073491	1.355305466
SLC3A2 (1 of many)	0.912052117	1.155405405
nrp1b	0.912	0.842696629
cks1b	0.911949686	1.245454545
sptbn2	0.911904762	0.739669421
phkg2	0.911894273	1.340659341
si:dkey-33o22.1	0.911764706	0.52
gdf5	0.911764706	0.892857143
osgep	0.911764706	0.916083916
cep76	0.911764706	0.916666667
tbx1_1	0.911764706	0.92173913
chdh	0.911764706	0.923913043
CU929259.1	0.911764706	0.956521739
si:cabz01063885.1	0.911764706	1.121212121
usp3	0.911764706	1.125
atg12	0.911764706	1.281818182
pgm2l1	0.911764706	1.366666667
trub2	0.911764706	1.3828125
si:ch211-235i11.4	0.911764706	1.545454545
zgc:174935	0.911764706	1.6125
grpel1	0.911764706	1.650909091
fbxl20	0.911764706	1.9
cd81b	0.911671924	1.083870968
mbd3a	0.911637931	0.981283422
tlk1a	0.911627907	0.738636364
zgc:153031	0.911564626	1.439189189
elmod2	0.911564626	1.726415094
copa	0.911542611	0.819225251
rx1	0.911504425	0.445783133
arl6ip5b	0.911504425	1.15437788

c10h21orf59	0.911504425	1.38317757
kbtbd2	0.911458333	1.193939394
znf281b	0.911392405	1
gbp3	0.911392405	1.133333333
ampd3a	0.911392405	1.509803922
zgc:112255	0.911314985	1.698039216
fam101b	0.911290323	0.972222222
xbp1	0.911280846	0.837688442
fzd7a	0.911251981	0.792880259
abhd2a	0.911242604	1.172932331
hmgb3a	0.911147903	1.2234537
scg3	0.911140584	1.128467153
hsdl2	0.911138924	1.126564673
hoxa2b	0.911111111	0.659574468
chd1l	0.911111111	0.878787879
kif16ba	0.911111111	1.071428571
jazf1a	0.911111111	1.076923077
btr01	0.911111111	1.315789474
arv1	0.911111111	1.409638554
htra4	0.911111111	1.527777778
ahcyl1	0.911025145	0.8321513
mob1bb	0.911016949	1.683615819
rnf144ab	0.910958904	0.864
arhgap32b	0.910891089	0.5
atg4da	0.910891089	1.084507042
fgfr2	0.910852713	0.773913043
usp4	0.910852713	0.945652174
znf207a	0.910810811	0.619335347
MMADHC (1 of many)	0.910780669	1.169230769
si:ch73-250a16.5	0.910714286	0.592592593
znhit3	0.910714286	0.613333333
mtif2	0.910714286	1
rps19bp1	0.910714286	1.074074074
mier1b	0.910714286	1.285714286
pisd	0.910638298	0.873737374
COQ10A	0.910569106	1.680851064
mtss1lb	0.910447761	0.756756757
abi2a_1	0.910447761	1.157303371
myb	0.910447761	1.30952381
sort1a	0.910377358	1.35915493
rpa1	0.910355487	1.096121417
dip2ba	0.910344828	0.81372549
arfgap2	0.910321489	0.870833333
pdia2	0.910271547	1.662068966
bcl11aa	0.91025641	0.559322034
lmcd1	0.91025641	1.381578947
hsf2	0.91023622	1.343313373
mak	0.910179641	1.192307692
si:ch211-271b14.1	0.91011236	1.069306931
lipg	0.91011236	1.231343284
mpp2b	0.91005291	0.636363636
edem1	0.91005291	1.511450382
pcp4a	0.910047847	0.810408922
zgc:112175	0.910034602	0.819277108
tnip1	0.910025707	1.021582734
clpxa_1	0.910016978	0.864097363
eef2k	0.91	1.048076923
jagn1b	0.91	1.534591195
serp1	0.90990991	1.243339254
tle2	0.90990991	0.543010753
ACAP2 (1 of many)	0.90990991	0.777777778

si:dkey-119m7.8	0.90990991	1.174418605
gchfr	0.909871245	1.25
hcfc1a	0.909836066	0.525345622
pa2g4b	0.909811695	1.232767233
fliii	0.909722222	1.12173913
snu13b	0.909695817	1.104557641
tom1l2	0.909677419	1.233766234
tom1l2	0.909677419	1.233766234
msx1b	0.909677419	1.607594937
zgc:110333	0.909677419	1.697841727
mthfd2	0.909560724	1.086567164
dtnbp1a	0.909547739	1.182320442
GCA	0.909454061	1.035322777
chaf1a	0.909433962	0.628440367
hnrnpa0b	0.909431138	0.535877863
paip1	0.909395973	1.095833333
cdh1	0.909395973	1.481132075
sox19a	0.909365559	0.565916399
ppm1g	0.909333333	0.623493976
KCNN2	0.909090909	0.62962963
tsen15	0.909090909	0.717948718
si:ch211-215d8.2	0.909090909	0.844444444
rfc1	0.909090909	0.860215054
fignl1	0.909090909	0.935483871
tusc5b	0.909090909	0.958333333
rhobtb2a	0.909090909	1.162162162
CABZ01118267.1	0.909090909	1.233333333
skap2	0.909090909	1.444444444
pde10a	0.909090909	2.166666667
gfra3	0.909090909	0.388888889
adcy5	0.909090909	0.823529412
hivep2a	0.909090909	0.844155844
rab27b	0.909090909	0.846153846
pkd2l1	0.909090909	0.875
pbld	0.909090909	0.924137931
shroom4	0.909090909	0.926829268
anapc4	0.909090909	0.946666667
znf970	0.909090909	1
DTX3	0.909090909	1.02
mfsd6a	0.909090909	1.052631579
tspan2a	0.909090909	1.090047393
sh3rf2	0.909090909	1.142857143
CR388132.1	0.909090909	1.235294118
trmt11	0.909090909	1.243902439
znf1105	0.909090909	1.357142857
si:dkey-20i20.2	0.909090909	1.5
mavs	0.909090909	1.514285714
zgc:113443	0.909090909	2
arf2a	0.908926417	0.987218045
tmem11	0.908910891	0.856521739
sfxn2	0.908902692	1.02513465
HACD1	0.90881459	1.906605923
rtn1b	0.908805031	0.595744681
lap3	0.908730159	0.97188755
hspb6	0.908730159	1.712121212
crygm2d6	0.908720733	0.864997948
nras	0.908675799	1.084656085
qsox1	0.908554572	0.651709402
mef2ca	0.908554572	1.03
rap1ab	0.908536585	1.197080292
pcdh1b	0.908496732	0.683168317

fbxo31	0.908450704	1.539823009
elovl5	0.908396947	1.191304348
si:ch211-116o3.5	0.908396947	1.223214286
tns3.2	0.908333333	0.901785714
nf2b	0.908333333	1.471910112
dtnbp1b	0.908256881	0.864
pxmp2	0.908256881	1.694915254
gab1	0.908163265	0.784946237
armc8	0.908163265	0.975903614
sh3bgrl3	0.908139535	1.358823529
endouc	0.908088235	0.844479005
zgc:91944	0.908045977	0.714285714
mrps33	0.908045977	1.362903226
opn1sw2	0.908015501	0.755651661
fam177a1	0.90797546	1.521929825
bms1	0.907834101	0.910447761
mrpl36	0.907834101	1.09047619
stk35	0.907730673	1.097402597
stmn2a	0.907702984	0.963878327
ythdf2	0.907630522	0.712195122
upf3b	0.907630522	1.912408759
CABZ01038517.1	0.907563025	0.567567568
fbp1a	0.907496013	1.151351351
cobl	0.907407407	0.727272727
guf1	0.907407407	1.086956522
zbtb7b	0.907407407	1.105263158
cby1	0.907407407	2.923076923
dido1	0.907284768	0.871559633
ccdc115	0.907284768	1.625
sdr16c5b	0.907251265	1.352430556
pdia3	0.907239819	0.916376307
asrgl1	0.907216495	1
stat3	0.907216495	1.215384615
tceb1a	0.907216495	1.420118343
ythdf1	0.907216495	0.903846154
im:7147486	0.907142857	0.752941176
fnta	0.907079646	0.981900452
mgrn1b	0.907063197	1.791139241
si:dkey-19e4.5	0.907017544	1.381773399
sat1a.1	0.907014682	1.491902834
zdhhc18b	0.906976744	0.580645161
arhgap12a	0.906976744	0.5875
bub1	0.906976744	0.644067797
ankrd44	0.906976744	0.74
fam19a5b	0.906976744	0.771428571
ep300a	0.906976744	0.881355932
tyw5	0.906976744	0.91025641
si:ch73-248e21.1	0.906976744	1.05
dhrs7b	0.906976744	1.256410256
tph1a	0.906976744	1.260869565
si:ch1073-340i21.2	0.906976744	1.267175573
si:zfos-411a11.2	0.906976744	1.568627451
sssca1	0.906882591	0.69005848
nqo1	0.906821963	1.631163708
si:ch211-286b5.5	0.906810036	1.339055794
si:ch211-212k18.4	0.906705539	0.790322581
rfx7	0.906666667	0.788461538
rabggtb	0.906666667	1.27014218
QSOX2	0.906666667	1.288888889
btbd3a	0.906666667	1.371428571
ano10b	0.906666667	1.593220339

si:dkeyp-72g9.4	0.906633907	0.671732523
atp6v0a1a	0.906605923	1.053731343
cx43.4	0.906565657	0.827868852
srpr	0.906565657	0.940789474
rcan3	0.906542056	0.927272727
fsta	0.90647482	1.028776978
timm13	0.906464924	1.037283622
lhx1a	0.906451613	0.508928571
xrn1	0.906432749	0.807407407
lrig2	0.906432749	1.083333333
yme1l1b	0.906403941	1.207692308
mrps18a	0.906403941	1.242105263
ccbl2_1	0.906403941	1.666666667
smad1	0.906367041	0.932489451
mylipb	0.906336088	1.351032448
pmp22b	0.906315059	1.262282092
lsm4	0.906293706	1.280071813
kdm2aa	0.90625	0.910828025
dhrsx	0.90625	0.871428571
bicc1b	0.90625	0.903614458
slc25a51b	0.90625	0.91011236
si:ch211-212g7.6	0.90625	1.044776119
si:ch211-265m20.8	0.90625	1.310810811
cntn4_2	0.90625	0.709677419
plagl2	0.90625	0.823529412
ehbp1l1b	0.90625	0.896551724
her8.2	0.90625	0.897435897
inppl1b	0.90625	0.966666667
klhl23	0.90625	1
cenpp	0.90625	1.028985507
zgc:110006	0.90625	1.216216216
rapsn	0.90625	1.346153846
nos1apa_2	0.90625	1.5
usp30	0.90625	1.761904762
fgg	0.906230722	1.102108768
ubl3a	0.906113537	0.917142857
itgbl1	0.906103286	0.946745562
si:ch211-106h4.12	0.906103286	1.115207373
terfa	0.906077348	0.852941176
slc12a2_1	0.906015038	0.829787234
znf281a	0.905982906	0.869047619
ARHGEF12 (1 of many)	0.905982906	0.968421053
rln3a	0.905982906	1.323232323
pdk1	0.905940594	1.419753086
pnpo	0.905940594	1.933823529
ccdc88c	0.905882353	0.827586207
si:ch73-119p20.1	0.905797101	1.153039832
limk2	0.905797101	0.972093023
sagb	0.905777778	0.891689373
ezrb	0.905718702	1.017786561
pyyb	0.905660377	0.388349515
rapgefl1	0.905660377	0.696969697
tmem181	0.905660377	0.824561404
pcnt	0.905660377	0.967741935
mad1l1	0.905660377	1.086956522
si:dkey-222f2.1	0.905660377	1.328729282
ca10a	0.905579399	1.240223464
cotl1	0.905532359	0.717992656
si:dkey-5i3.5	0.905511811	1.714285714
arhgap32a	0.905405405	0.882352941
olfm3a	0.905405405	1.069444444

dnase2	0.905405405	1.103448276
ugt2a7	0.905405405	1.277777778
nthl1	0.905405405	1.37704918
dnase1l3l	0.905405405	2.151898734
pcsk5b	0.905325444	1.007874016
cfl1	0.905236107	1.10380561
ndfip1l	0.905222437	1.247282609
gpt2	0.905109489	0.916666667
ptp4a3	0.905077263	1.276649746
tm9sf4	0.904988124	1.128939828
oxct1a	0.904967603	1.11898017
asap2b	0.904761905	1.445544554
slc22a21	0.904761905	0.756097561
gpr146	0.904761905	0.790441176
wfikkn2a	0.904761905	0.85106383
uggt1	0.904761905	1.13559322
si:ch1073-398f15.1	0.904761905	1.222222222
ndufaf2	0.904761905	1.770833333
zgc:172282	0.904761905	0.413793103
fam78ba	0.904761905	0.518518519
zmat4a	0.904761905	0.519230769
si:dkey-225f23.5	0.904761905	0.583333333
serinc4	0.904761905	0.647058824
spag1a	0.904761905	0.95631068
cxcr4a	0.904761905	0.977777778
zdhhc22	0.904761905	1
tmem229b	0.904761905	1.12037037
n4bp3	0.904761905	1.125
zgc:154058	0.904761905	1.2
zgc:193690	0.904761905	1.25
si:dkey-238i5.2	0.904761905	1.607142857
cygb2	0.904761905	1.690909091
ctsk	0.904661017	1.004048583
arglu1b	0.904509284	1.328413284
coro1cb	0.904471545	0.827195467
mrps25	0.904458599	1.307017544
si:ch211-194m7.3	0.904411765	1.082251082
ins	0.904285714	0.837387964
rnft2	0.904255319	1.206349206
CRYGB	0.904173107	1.25994695
ldb1a	0.904132231	0.8891258
si:ch211-214c7.4	0.904109589	0.464285714
sipa1l3	0.904109589	0.844827586
trip11	0.904109589	1.22826087
ap1s2	0.904109589	1.243944637
cwc15	0.904040404	0.987632509
myhz1.1_2	0.904020101	1.238709677
aaas	0.904	0.955752212
PXDN	0.904	1.053571429
fras1	0.904	1.072916667
tyr	0.903903904	0.882352941
vav3b_1	0.903846154	0.578947368
psme4b	0.903846154	0.847133758
pparab	0.903846154	1.19047619
st5	0.903703704	0.626760563
polr3e	0.903669725	1.188679245
maptb	0.903614458	0.527363184
cdkal1	0.903614458	0.933823529
SUN2	0.903614458	1.029850746
flvcr2b	0.903614458	1.62
lrrc58a	0.903614458	1.684210526

cab39l	0.903571429	1.441176471
tp53bp2b	0.903448276	0.680851064
gfap	0.903414634	0.657189721
mrpl52	0.903225806	1.557471264
lpgat1	0.903225806	0.660869565
znf318	0.903225806	0.776859504
dync1i2b	0.903225806	0.848484848
jag1b	0.903225806	0.865853659
si:ch211-235e15.1	0.903225806	0.8671875
dner	0.903225806	0.310344828
pappa2	0.903225806	0.612903226
nfil3-2	0.903225806	0.9125
ecscr	0.903225806	0.96875
chn2	0.903225806	1.08
zgc:136971	0.903225806	1.217391304
si:dkey-5i16.5_1	0.903225806	1.75
si:ch211-89o9.4	0.903225806	2.75
tm9sf3	0.903092784	1.470588235
hspa4l	0.903030303	1.304347826
NAT8	0.903030303	2.136842105
fzd2	0.902946274	0.866666667
pfkpa	0.902927581	0.522954092
ywhaqb	0.902923977	1.036999748
fam184a	0.902912621	0.875
tmem53	0.902912621	1.493333333
cdk2ap2	0.902821317	0.908011869
irx7	0.902777778	0.836363636
osr1	0.902777778	0.976
ackr3b	0.902777778	1.112831858
nxph2a	0.902777778	1.8
csnk1g2a	0.902723735	0.875706215
si:ch211-194c3.5	0.902621723	0.854054054
use1	0.902597403	1.323308271
b3gnt2l	0.902564103	1.391608392
atp2b1a	0.902527076	0.878306878
ano11	0.902439024	0.676470588
pias2	0.902439024	0.954887218
pex26	0.902439024	0.957446809
ccm2	0.902439024	1.196428571
pcdh2aa1	0.902439024	1.235294118
stx2a	0.902439024	1.411764706
BX530097.1	0.902439024	1.538461538
si:dkeyp-69c1.7	0.902439024	1.615384615
zgc:101583	0.902439024	2.347826087
api5	0.902439024	1.096590909
hsp90aa1.1	0.90237099	1.368243243
tdh2	0.902255639	1.423076923
sec23ip	0.902173913	0.695945946
skib	0.902173913	0.845637584
si:ch1073-435i13.1	0.902173913	0.9375
lama4	0.902116402	0.944444444
si:dkey-12j5.1	0.902061856	1.620689655
cacnb2a	0.901960784	0.866666667
wdr6	0.901960784	0.923076923
CABZ01052573.1	0.901960784	1
mrps27	0.901960784	1.047169811
rtel1	0.901960784	1.131578947
si:dkey-266m15.5	0.901960784	1.131578947
pop4	0.901960784	1.830985915
anxa5b	0.901864573	1.303383897
adam22	0.901840491	0.588888889

pdcd5	0.901812689	1.550669216
BICD2 (1 of many)	0.901785714	1.071005917
mmp14a	0.901734104	0.889370933
si:ch211-157c3.4	0.901724138	1.705627706
prkcdb	0.901709402	1.11627907
mag	0.901694915	0.72361809
CBFA2T3	0.901639344	0.472868217
aaed1	0.901639344	1.097222222
nat15	0.901639344	1.306358382
suds3	0.901639344	1.345238095
itga6b	0.901639344	1.57827476
larp7	0.901639344	1.795454545
efs	0.901515152	0.691489362
peli1b	0.901515152	0.89010989
fhl1a	0.901477833	0.921465969
suz12b	0.901477833	1.019354839
ndst3	0.901408451	0.352941176
si:ch73-132f6.5	0.901408451	1.174757282
ngdn	0.901408451	1.293103448
aggf1	0.901408451	1.454545455
zgc:55558	0.901408451	1.081272085
gpd1l	0.901408451	1.354651163
sec14l1	0.901345291	0.835616438
actb1	0.901287764	0.707992093
si:ch211-167m1.5	0.901234568	1.283018868
glt8d1	0.901234568	1.592592593
lxn	0.901162791	0.92276964
gnb1b	0.901030928	0.710914454
rit1	0.900990099	1.247524752
qars	0.900928793	0.978181818
ssb	0.900917431	1.116981132
plpp3	0.900914634	0.949324324
anp32a	0.900833699	1.038869258
ptgdsa	0.900826446	0.927835052
mcm7	0.900735294	0.563380282
ftr82	0.900684932	1.196850394
rufy3	0.900647948	1.377708978
ppp2r2aa	0.900621118	0.845360825
utp23	0.900621118	1.272727273
zgc:66448	0.900621118	1.34
hpca	0.900530504	0.921248143
wfdc2	0.900398406	0.711864407
zgc:163030	0.900321543	2.019920319
fkbp1b	0.900302115	1.31433506
nup107	0.900277008	0.962585034
zgc:56525	0.900184843	1.32038835
tbkbp1	0.9	0.409836066
erfl3	0.9	0.568421053
si:ch211-149b19.2	0.9	0.705882353
hoxd9a	0.9	0.781954887
helq	0.9	0.8
STRADB	0.9	0.8
si:ch211-14g13.5p	0.9	0.803278689
raph1b	0.9	0.8125
swi5	0.9	0.82
prkg1a	0.9	0.836734694
mafgb	0.9	0.851851852
ggact.3	0.9	0.875
znf704	0.9	0.891891892
plxnb2a_1	0.9	0.897590361
zgc:152830_1	0.9	0.902097902

fam73b	0.9	0.942857143
slc16a13	0.9	0.947368421
otog	0.9	0.952380952
rfc2	0.9	0.975308642
si:dkey-125i20.2	0.9	0.981132075
lpcat4	0.9	0.983870968
mcm9	0.9	1
myct1b	0.9	1
shroom3_1	0.9	1
tcf7	0.9	1
loxl4	0.9	1.083333333
serpinh2	0.9	1.090909091
gadd45gb.1	0.9	1.126262626
ddr2l	0.9	1.204545455
b4galt4	0.9	1.213675214
olfml2bb	0.9	1.24
PHTF1	0.9	1.27027027
scnm1	0.9	1.393939394
glis2a	0.9	1.4
F2RL2 (1 of many)	0.9	1.4
SLC22A3	0.9	1.421052632
zgc:113298	0.9	1.434782609
fitm2	0.9	1.559210526
bend5	0.9	1.714285714
si:rp71-1g18.13	0.9	2.076923077
si:ch211-238e22.2_2	0.9	2.25
CABZ01060373.1	0.9	2.388888889
si:ch211-266k2.1	0.9	2.428571429
pknox1.2	0.9	1.015873016
pgam1a	0.9	1.059192201
prelid3a	0.9	1.573033708
pla2g12a	0.9	1.6
AIFM2	0.899782135	1.764478764
prss59.2	0.899740709	1.242246145
mrto4	0.899728997	1.807174888
amy2a	0.899628253	0.699029126
rbm39a	0.899628253	1.036363636
rab2a	0.899620184	1.076335878
ube2l3b	0.899575672	0.974137931
si:dkey-260j18.2	0.899470899	0.848484848
rrm2_2	0.899465241	0.794392523
ngly1	0.899408284	1.319148936
abl1	0.899371069	0.963235294
zfx	0.899371069	1.00862069
lrig1	0.899328859	0.806451613
diexf	0.899224806	1.240384615
vmhc	0.899193548	1.605263158
anxa11a	0.899185336	0.99044586
tbcc	0.899159664	1.202020202
cmasa	0.898907104	0.960212202
flnca	0.898876404	1.031746032
elmsan1b	0.898876404	1.105263158
ube2c_1	0.898832685	1.142201835
fdps	0.898832685	1.759036145
mcf2la	0.898809524	0.560606061
calm3a	0.898758981	1.43373494
si:cabz01101003.1	0.898734177	0.676056338
ccl34b.1	0.898734177	0.9
btbd17b	0.898734177	0.902097902
dnajc3b	0.898734177	0.940740741
rnf145a	0.898734177	1.133333333

zgc:162184	0.898734177	2.440677966
tmem115	0.898648649	1.225
bmi1b	0.898648649	1.333333333
lama5	0.898550725	1
CABZ01072096.1	0.898550725	0.756756757
slc6a16b	0.898550725	0.918918919
fhdc4	0.898550725	1.071428571
msi1	0.898496241	0.905027933
add3a	0.898488121	0.988795518
chchd6a	0.898477157	0.97008547
pi4k2b	0.8984375	0.87804878
nfyc	0.898419865	0.706030151
tmem134	0.898395722	1.858333333
si:ch211-246m6.5_2	0.898305085	0.791044776
mark4a	0.898305085	1.053571429
srfa	0.898305085	1.090909091
slc12a7a	0.898305085	1.097560976
dhrs7cb	0.898251192	1.246998285
cry4	0.898230088	0.708154506
ldhbb	0.898148148	0.791208791
ap1b1	0.898089172	0.944700461
map7d3	0.898089172	1.081300813
atg3	0.898089172	1.554913295
stip1	0.898021309	1.175889328
fgb	0.897986577	0.9625512
kif13ba	0.897959184	0.868852459
eefsec	0.897959184	1.086956522
hoxa13b	0.897959184	1.103448276
si:ch73-308b14.1	0.897849462	1.37992126
slc27a2b	0.897727273	0.748538012
znrf3	0.897727273	1.157142857
mospd2	0.897727273	1.160714286
timm23a	0.897657213	1.221902017
sart3	0.897637795	0.794392523
srcap	0.897637795	0.863013699
atp6v1e1a	0.897590361	1.715384615
amdhd2	0.897560976	0.797235023
si:dkeyp-116h7.2	0.897435897	0.555555556
mllt1a	0.897435897	0.68627451
myo7aa	0.897435897	0.838709677
efna5b	0.897435897	0.842105263
dnajc30b	0.897435897	1.142857143
trim25	0.897435897	1.4
ccdc149b	0.897435897	1.561403509
cptp	0.897435897	2.419354839
apom	0.897394137	1.073805751
tbx2b	0.897379913	0.921511628
pcdh18a	0.897196262	0.606741573
acanb	0.897196262	0.895522388
msl1b	0.897196262	1.475903614
csnk1g2b	0.897058824	0.934911243
tgm2a	0.897058824	1.078369906
npy	0.897058824	1.014492754
taf8_1	0.896907216	0.95
si:ch73-42p12.2	0.896797153	1.63546798
lect1	0.896782842	0.658640227
cdca7a	0.896774194	0.682857143
cux1a	0.896774194	0.724770642
gna12a	0.896774194	1.324561404
fscn1a	0.89673913	0.655286344
tmem30aa	0.896703297	1.203448276

pcf11	0.896678967	1.351851852
opn1mw1	0.896671061	0.722432905
lhx9	0.896666667	0.815899582
3-Mar	0.896551724	0.695652174
cnnm2a	0.896551724	0.733333333
poc1a	0.896551724	1.041666667
atpaf2	0.896551724	1.058823529
bet1l	0.896551724	1.22
dhx33	0.896551724	1.25
prr15lb	0.896551724	1.275862069
mov10b.1	0.896551724	1.411764706
nr1d2b	0.896551724	1.436654367
BX323087.1	0.896551724	1.5
ublcp1	0.896551724	1.605633803
aftphb	0.896551724	1.630434783
rap1b	0.896507115	1.446337308
zw10	0.896341463	1.1015625
tmbim1	0.896341463	1.452554745
aldh6a1	0.896250455	1.084169884
mbpb	0.896238651	1.034124629
aph1b	0.896193772	1.27804878
foxn3	0.896103896	0.86440678
sec13	0.896045198	1.103942652
nasp	0.896	0.92
rbm47	0.896	0.849740933
zgc:110540	0.896	1.013043478
asun	0.896	1.16
srsf11	0.895939086	0.814479638
eya1	0.895910781	0.9342723
htatsf1	0.895890411	1.183098592
prickle3	0.895833333	0.75
PXMP4	0.895833333	0.865168539
zgc:123068	0.895833333	0.955223881
mettl20	0.895833333	2
ddx39ab	0.895821727	0.658536585
nsfa_1	0.895738204	0.808302808
zgc:153993	0.895652174	1.597765363
tia1	0.895604396	0.909090909
col11a2	0.895578551	0.549755302
tmem136b	0.895522388	0.723076923
dysf	0.895522388	1.125
CEP120	0.895522388	1.125
cart4	0.895522388	1.44
samd13	0.895522388	1.48
flrt3	0.895384615	1.17167382
PDE8A	0.895348837	0.567164179
ccser2a	0.895348837	0.814814815
znf207b	0.895348837	1.18699187
tead1b	0.895348837	1.259259259
tmem204	0.895238095	1.060606061
prdm1b_2	0.895238095	1.308641975
itgav	0.895225464	1.170873786
prss23	0.89516129	0.881818182
nktr	0.89516129	1.842424242
rab11a	0.894941634	1.069473684
fam46ba	0.894878706	1.859259259
sec61b	0.894784995	0.80794702
tbx4	0.894736842	0.476190476
fzd8b	0.894736842	0.545454545
pex5la	0.894736842	0.65
wdr43	0.894736842	0.756147541

arfgef1	0.894736842	0.764705882
prr11	0.894736842	0.8
si:ch73-376g11.3	0.894736842	0.820512821
si:dkey-262g12.14	0.894736842	0.952380952
depdc1a	0.894736842	0.962962963
sdhda	0.894736842	1
ildr1a	0.894736842	1.037735849
akip1	0.894736842	1.052631579
RNF224	0.894736842	1.083333333
txndc16	0.894736842	1.208333333
nfe2l2b	0.894736842	1.394366197
FO704783.1	0.894736842	1.444444444
prmt9	0.894736842	1.46875
ska2	0.894736842	1.590517241
tex9	0.894736842	3.142857143
FAM126B	0.894736842	0.590163934
rfwd3	0.894736842	0.803921569
stx18	0.894736842	0.834862385
si:ch1073-303k11.2	0.894736842	0.965909091
pds5a	0.894736842	0.973684211
ankle2	0.894736842	1.078947368
alkbh8	0.894736842	1.125
si:rp71-19m20.1	0.894736842	1.341176471
cldn5b	0.894736842	1.379679144
ano9b	0.894736842	1.43902439
eif4eb	0.894736842	1.8125
CELA1 (1 of many)_1	0.894626124	0.883878241
epn2	0.894514768	0.908629442
si:ch73-162j3.5	0.894366197	1.010869565
flj11011l	0.894366197	2.53125
shox	0.894308943	0.918699187
gtpbp10	0.894308943	1.4375
lypc	0.894273128	1.243967828
sox12	0.894230769	1.214285714
oxsr1a	0.894230769	1.393442623
si:ch211-214j24.10	0.894117647	0.832758621
tsnax	0.894117647	0.885245902
mcee	0.894117647	1.234482759
gpalpp1	0.894039735	1.134453782
SIL1	0.894039735	1.789473684
si:ch211-207c15.7	0.893939394	0.717391304
slc38a9	0.893939394	1.056603774
ghra	0.893939394	1.216666667
gcfc2	0.893939394	1.551020408
vsig8b	0.893939394	1.663461538
inpp5f	0.89380531	0.774647887
zdhhc6	0.893700787	1.295698925
si:ch211-196c10.15	0.893617021	0.595744681
zcchc8	0.893617021	0.88
brf1b	0.893617021	0.903225806
slc38a3a	0.893617021	0.924528302
acsm3	0.893617021	1.071428571
samd10a	0.893617021	1.602941176
slc10a2	0.893617021	1.84375
apoa4b.2	0.893600617	0.959037378
ezh2	0.893491124	0.881889764
slc38a2	0.893442623	0.737967914
gypc	0.893442623	1.454545455
agpat3	0.893421053	0.855400697
tax1bp3	0.893382353	2.052356021
cbx5	0.893371758	1.052009456

naa30	0.893333333	0.766423358
pknox2	0.893333333	0.811320755
btr30	0.893333333	1.272727273
eci2	0.893271462	0.829326923
cd81a	0.893263992	1.235200509
ccnd1	0.893258427	1.073883162
ctnnd1	0.893203883	0.771317829
arcn1a	0.893203883	1.197640118
METTL26	0.893203883	2.068181818
si:ch73-52f24.4	0.89316026	1.405494505
calm2b	0.893144939	1.220149254
srsf1a	0.89311408	0.834132311
si:dkey-17e16.15	0.893081761	0.610526316
elf2a	0.893081761	0.96969697
pik3r3b	0.893048128	0.822429907
si:dkeyp-69b9.6	0.893048128	1.007633588
rbm12b	0.893048128	1.064516129
zgc:112160	0.893010423	2.024757282
si:ch211-113g11.6	0.892857143	0.434782609
tagapb	0.892857143	0.851851852
znf1009_2	0.892857143	0.974358974
adcyap1a	0.892857143	1.196428571
CU633908.1	0.892857143	1.387755102
si:dkeyp-20g2.1	0.892857143	1.434782609
timd4	0.892857143	1.8
sumo2b	0.892857143	0.74601227
rreb1a	0.892857143	0.803030303
parp1	0.892857143	0.95035461
slc22a7a	0.892857143	1.011764706
KRT23	0.892857143	1.762295082
rpia	0.892744479	1.011235955
emp2	0.892733564	1.109704641
mta2	0.892682927	0.602446483
prrc1	0.892655367	0.873417722
nrbp2a	0.892655367	1.572649573
lamtor3	0.89261745	1.483870968
tmcc3	0.892523364	0.956756757
cd2bp2	0.892523364	1.385714286
itgb4	0.892508143	1.208737864
aplnrb	0.892473118	0.392156863
znf800a	0.892473118	0.803030303
gfra2b	0.892473118	0.944444444
cyp27c1	0.892473118	1.23880597
tecra	0.892473118	1.267605634
ascc2	0.892473118	1.416666667
gjd2b	0.892473118	1.672413793
ube2h	0.892268695	0.880718954
aatkb	0.892156863	0.918032787
b4galt7	0.892156863	1.957446809
stx16	0.892086331	1.397959184
snd1	0.892078071	0.81375
marcksb	0.892062538	0.859065156
zgc:101810	0.892030848	1.222570533
eif3ja	0.89194499	1.556994819
map3k10	0.891891892	0.666666667
ephb1	0.891891892	0.692307692
ppp4r4	0.891891892	0.708333333
banp	0.891891892	0.714285714
pigo	0.891891892	0.769230769
pik3c2a	0.891891892	0.920289855
borcs7	0.891891892	1.037037037

pxdc1b	0.891891892	1.096774194
si:ch211-225g23.1	0.891891892	1.2
zgc:174310	0.891891892	1.346153846
p4htm	0.891891892	1.473684211
snrnp27	0.89184953	0.961360124
ddx61	0.89184953	1.64254386
man1a1	0.891666667	1.038461538
nus1	0.891666667	1.091891892
asah1a	0.891666667	2.054054054
twist2	0.891625616	1.310344828
secisbp2	0.891566265	0.933333333
slc9a6a	0.891566265	0.983333333
fem1b	0.891566265	1.127272727
amotl2a	0.891495601	1.003344482
CU856539.2	0.891472868	1.101851852
ptges3b	0.891472868	1.421499293
bc2	0.89133627	1.480392157
skp2	0.891304348	0.837837838
pbld2	0.891304348	1.0859375
rnf24	0.891304348	1.192592593
wu:fc21g02	0.891262136	0.847345133
tspan12	0.89119171	1.25
napbb	0.891176471	0.940540541
cbfb	0.891156463	0.948717949
rab3da	0.891089109	0.945652174
rcn1	0.891050584	1.154228856
foxo3b	0.891041162	0.982026144
sall3b	0.890909091	0.325
mical3b	0.890909091	0.555555556
lmo2	0.890909091	0.770491803
dars2	0.890909091	0.934782609
gclm	0.890909091	1.423728814
hao1	0.890814558	0.83943662
CABZ01065131.1	0.890756303	0.757281553
vmhcl	0.890756303	1.847457627
mrpl2	0.890660592	1.00234192
mef2aa	0.890625	1.037593985
slc16a1b	0.890625	1.22327044
pik3c2b	0.890625	0.741935484
fgf12a	0.890625	0.770642202
tmtc2b	0.890625	0.86
mogat3b	0.890625	1
si:ch73-389b16.1	0.890625	1.669902913
fstl1b	0.890566038	1.340290381
pkd2	0.890510949	1.114035088
aldh9a1b	0.89047619	1.029411765
phex	0.890459364	1.006711409
srsf2a	0.890410959	0.691520468
atp2a3	0.890410959	0.869918699
mgmt	0.890410959	1.5
si:dkey-193c22.1	0.890410959	1.659574468
pabpn1	0.890374332	0.763337893
aqp12	0.890343699	0.690434783
stk38b	0.890243902	1.052631579
pinx1	0.890243902	2.260273973
FQ311894.1	0.89017341	0.724137931
slc43a1b	0.89017341	1.16
bves	0.89017341	1.190184049
mecom	0.89010989	0.75
si:ch211-284e13.6	0.89010989	0.930232558
ndufb11	0.89010989	1.021519543

nucb1	0.89010989	1.727699531
capza1a	0.890093999	0.932216299
zdhhc13	0.890052356	0.720588235
si:ch211-202e12.3	0.889908257	0.977272727
ncl	0.889892324	0.889788054
si:dkey-21a6.5	0.88976378	0.804195804
ctrl	0.889724311	1.43490701
mtch2	0.889721627	1.068720379
csrp1b	0.889705882	0.932989691
kdm6bb	0.88961039	0.647727273
tbcb	0.889534884	1.552567237
synpo2b	0.889502762	1.316455696
chrna1	0.889487871	1.177142857
zgc:153615	0.889312977	0.528497409
dnajc1	0.889212828	1.274305556
pygb	0.889182058	0.987138264
hadhb	0.889161053	1.129411765
atf4b	0.889158576	1.150339476
aktip	0.889140271	1.378947368
si:zfos-1011f11.2	0.888888889	0.409090909
rapgef6	0.888888889	0.642857143
prpf8	0.888888889	0.693415638
kmt2a	0.888888889	0.753623188
ska1	0.888888889	0.868421053
top1mt_1	0.888888889	0.936170213
fgf6b	0.888888889	0.95
cpt2	0.888888889	0.97515528
mcama	0.888888889	1.011111111
lrtm1	0.888888889	1.052631579
tbx5a	0.888888889	1.133333333
nkx2.2b	0.888888889	1.166666667
gstk1	0.888888889	1.285714286
myh7ba	0.888888889	1.35
ccne2	0.888888889	1.372881356
znf1012	0.888888889	1.454545455
commd4	0.888888889	1.531914894
nt5c1ba	0.888888889	1.666666667
spty2d1	0.888888889	1.688073394
slc35f4	0.888888889	0.454545455
CU861453.1	0.888888889	0.761904762
mid2	0.888888889	0.785714286
pdik1l	0.888888889	0.825
adamts12	0.888888889	0.863636364
zmynd19	0.888888889	0.91011236
lace1b	0.888888889	0.952380952
LPIN1 (1 of many)	0.888888889	0.956521739
bmper	0.888888889	0.984615385
helb	0.888888889	1
thada	0.888888889	1
dgkza	0.888888889	1
CRISPLD2	0.888888889	1
exosc4	0.888888889	1.022222222
cemip	0.888888889	1.031914894
mapk11	0.888888889	1.052631579
col19a1_2	0.888888889	1.058823529
znrf2a	0.888888889	1.146341463
dlk2	0.888888889	1.1875
CABZ01009157.1	0.888888889	1.198412698
rag1	0.888888889	1.24
cep55l	0.888888889	1.263157895
rpz	0.888888889	1.396396396

prpf4bb	0.888888889	1.4140625
spryd3	0.888888889	1.579710145
zgc:110366	0.888888889	1.698412698
sh3bgrl2	0.888446215	1.153488372
adnp2b	0.888429752	1.435582822
plpp1a	0.888361045	1.035545024
arl8	0.888217523	1.026845638
umps	0.888157895	0.52
lrrc57	0.888157895	1.550847458
DHDH (1 of many)	0.888143177	1.052631579
kif22	0.888111888	0.71641791
si:dkey-261h17.1	0.888082902	0.959235669
eya2	0.888059701	1.042016807
cdc14ab	0.888	1.103092784
and2	0.887862606	1.244221106
SHROOM2	0.887850467	0.739130435
zgc:110789	0.887850467	0.807017544
slc6a13	0.887850467	1.202247191
tmed10	0.887824897	0.908610272
pitpnb	0.887804878	1.248
scrt1b	0.887755102	0.6
afmid	0.887755102	1.227848101
arl6ip5a	0.887755102	1.691176471
insig1	0.887700535	1.77734375
hspa14	0.887640449	1.078740157
and3	0.887618297	1.094899666
mob2a	0.887596899	1.028708134
scp2a	0.887581699	1.155647383
grtp1a	0.887573964	0.930481283
adpgk	0.8875	0.929824561
sub1a	0.887473461	1.092578424
pbdc1	0.887473461	1.404761905
rbm38	0.887323944	0.828358209
zgc:194281	0.887323944	0.9
loxl3b	0.887218045	1
cdkn3	0.887096774	0.853333333
ppp6r2b	0.887096774	0.891891892
nup35	0.887096774	0.956140351
hacl1	0.887096774	1.241758242
msx3	0.887096774	1.241758242
islr2	0.887045814	1.048351648
ppp1r21	0.88700565	1.060150376
ppp6r2a	0.886877828	1.161616162
bmpr1aa	0.886861314	1.246231156
plxna1b	0.886792453	0.527272727
drd1b	0.886792453	0.705882353
urb1	0.886792453	1.052631579
lrrc28	0.886792453	1.076923077
spryd7a	0.886792453	1.299270073
zgc:92242	0.886731392	1.611111111
2-Sep	0.886652542	1.141943734
nop10	0.886649874	0.973333333
plcd4b	0.886597938	1.472222222
slc1a3a	0.88647343	1.036827195
six1a	0.886363636	0.661654135
RAPGEF2 (1 of many)	0.886363636	0.924731183
PTP4A3 (1 of many)	0.886363636	1.262345679
p2ry1	0.886363636	0.523809524
sgms2	0.886363636	1.074074074
trip10a	0.886363636	1.279411765
si:rp71-26b20.1	0.886363636	1.424242424

toporsa	0.886363636	2.111111111
tns1b	0.886227545	0.913669065
luc7l	0.886198547	0.855524079
spred3	0.886178862	0.522222222
rbm8a	0.886153846	1.346209913
ccdc85b	0.886138614	1.295454545
zgc:123238	0.886138614	1.326923077
pdgfab	0.886075949	1.101449275
amotl2b	0.886075949	1.1875
sdad1	0.886039886	1.253378378
rab20	0.885964912	0.85
tcp11l1	0.885964912	1.025
sfmbt1	0.885964912	1.146067416
sspn	0.885964912	1.278538813
rnf170	0.885844749	0.920454545
tubb2	0.885826772	0.795212766
ech1	0.885813149	1.100775194
gdf10b	0.885714286	0.618421053
si:ch211-165g14.1	0.885714286	0.916666667
pde12	0.885714286	1.372881356
fam195b	0.885714286	1.454545455
casp8l2	0.885714286	1.52173913
kbtbd7	0.885714286	1.576923077
KYAT3 (1 of many)_1	0.885714286	2.416666667
pdcd11	0.88559322	0.907317073
zgc:153675	0.88559322	1.058558559
igfn1.3	0.885585586	1.85733882
zgc:153018	0.885542169	1.309659091
mbd3b	0.885509839	0.899590164
ppfibp1b	0.885496183	1.119565217
elovl4a	0.885416667	0.68503937
dnajb1b	0.885416667	1.320512821
cul3a	0.885375494	1.182825485
HIKESHI	0.885350318	0.825503356
eng1b	0.885350318	1.166666667
epn1	0.885304659	0.947867299
fuca1.1	0.885271318	1.093397746
sepsecs	0.885245902	0.97826087
rnls	0.885245902	1.04587156
CABZ01066926.1	0.885245902	1.129032258
zdhhc15b	0.885245902	1.179310345
rhocb	0.88516129	0.937681159
epb41l5	0.885135135	0.734375
gas7a	0.885057471	0.840909091
hfe2	0.885057471	1.126582278
cmtm7	0.885	1.222772277
gstm.1	0.884984026	1.562171629
ywhaba	0.884980116	1.086206897
pou6f1	0.884955752	0.8125
CABZ01051572.1	0.884955752	0.923809524
sox9b	0.884892086	0.782945736
ddx52	0.884816754	1.172839506
eno2	0.884656085	0.674782609
golt1bb	0.884615385	1.184615385
si:dkeyp-97a10.1	0.884615385	1.424242424
uncx4.1	0.884615385	0.8
col16a1	0.884615385	0.940438871
FQ377654.1	0.884615385	1.038834951
cbwd	0.884615385	1.666666667
mybl1	0.884615385	0.708333333
TBC1D8	0.884615385	0.851851852

akna	0.884615385	0.916666667
acsl3a	0.884615385	0.971428571
si:rp71-62i8.1	0.884615385	1
cox18	0.884615385	1.011111111
zgc:172128	0.884615385	1.043478261
grap2b	0.884615385	1.055555556
irf2	0.884615385	1.127659574
ankrd10a	0.884615385	1.404761905
pvrl4	0.884615385	1.813953488
zgc:153733	0.884615385	2.058823529
dgkaa	0.884476534	1.098130841
dlx5a	0.884462151	0.85198556
slbp	0.884462151	1.685185185
si:ch73-389b16.2	0.884393064	0.956140351
si:dkey-22o12.2	0.884297521	0.870588235
lin7a	0.884297521	1.364145658
cth	0.884267631	1.397894737
olfm1b	0.884231537	0.619158879
dffb	0.884210526	1.791044776
kctd5a	0.884169884	0.898617512
anos1a	0.884120172	0.558139535
rho	0.884115029	0.328411887
TMSB15A	0.88410804	1.281935259
opn1lw2	0.884079392	0.60677406
glb1	0.884057971	1.130434783
gnptab	0.884057971	1.132653061
si:ch73-126m5.4	0.884057971	1.244444444
slc25a16	0.884057971	1.352678571
si:dkey-48p11.3	0.884057971	1.456140351
asxl2	0.884057971	1.619047619
plagx	0.884	0.87755102
ppargc1a	0.883928571	0.680555556
elf3	0.883928571	1.115789474
gstt1a	0.883886256	0.854435831
tars	0.883783784	1.282051282
tfa	0.883783146	0.814274943
CABP7 (1 of many)	0.88372093	0.424242424
oxct1b	0.88372093	0.902912621
MMP23B	0.88372093	0.941176471
SLC2A4RG	0.88372093	0.947368421
si:ch211-257p13.3	0.88372093	1.291666667
casp7	0.88372093	1.495238095
zgc:113279	0.88372093	2.214285714
ap2s1	0.883682008	1.722067039
psmd8	0.883614089	1.537284895
ccdc58	0.883597884	1.333333333
pptc7b	0.883597884	1.491071429
nucb2a	0.883575884	1.287292818
hacd2	0.883445946	0.777163904
znf710a	0.883333333	0.700934579
tspan33a	0.883333333	1.848101266
perp	0.883268482	1.617407072
itm2ca	0.883211679	0.599514563
pdlim3b	0.883211679	1.204724409
zgc:56585	0.883211679	1.560344828
fam53b	0.88317757	0.852803738
prss12	0.883116883	1.096153846
phf10	0.883116883	1.168067227
skiv2l2	0.883116883	0.840206186
hipk3b	0.882978723	1.194029851
ostf1	0.882978723	1.265151515

med24	0.882882883	0.901098901
cyth3a	0.882882883	1.794520548
tmed2	0.882847896	1.11577424
gipc1	0.8828125	1.754098361
TMEM30B	0.882758621	1.358333333
si:dkey-102c8.3	0.882758621	1.43902439
mxra8b	0.882736156	0.770440252
ache	0.882722513	1.036666667
elavl1	0.882701422	1.15942029
ugp2b	0.882539683	1.211111111
ccdc32	0.88253012	1.137123746
abcb9	0.882352941	1.024390244
sytl4	0.882352941	1.119047619
si:ch211-136k14.3	0.882352941	1.121212121
hibadha	0.882352941	1.231958763
tarbp2	0.882352941	1.395833333
ARHGAP22 (1 of many)	0.882352941	0.233333333
cldnj	0.882352941	0.648648649
bxdc2	0.882352941	0.739463602
gdpd5b	0.882352941	0.805970149
trim35-7	0.882352941	0.833333333
si:dkey-262g12.11	0.882352941	0.882352941
nin	0.882352941	0.916666667
pask	0.882352941	0.923076923
CR774179.1	0.882352941	1
cntln	0.882352941	1
syt10	0.882352941	1
mgat1a	0.882352941	1.037383178
ngfb	0.882352941	1.096774194
cybb	0.882352941	1.238095238
aipl1	0.882352941	1.25
btr21	0.882352941	1.264705882
mchr2	0.882352941	1.333333333
si:ch211-165d12.4	0.882352941	1.384615385
bmp2b	0.882352941	1.421568627
slc19a2	0.882352941	1.541666667
gbp1	0.882352941	1.833333333
cul5b	0.882352941	1.846153846
tmem41b	0.882352941	1.882352941
si:dkey-253d23.9	0.882352941	2
MFAP4 (1 of many)_9	0.882352941	2.357142857
si:ch211-32p8.1	0.882242991	0.94017094
sap30l	0.882239382	0.907368421
gspt1l	0.882202305	0.942430704
slc1a3b	0.882051282	0.410344828
ap3m1	0.882051282	1.439490446
CU915738.1	0.882022472	1.123966942
gpr173	0.881889764	0.985915493
acp1	0.88185654	1.128078818
igf2a	0.881818182	1.563829787
cluha	0.881789137	0.706122449
hspe1	0.881761006	1.046134663
rcvrn3	0.881726159	0.806150467
GANAB (1 of many)	0.88172043	1.067164179
RNF166	0.88172043	1.603773585
si:ch211-268b13.6	0.881656805	0.715789474
fbp2	0.881630864	0.893442623
rnf38	0.881578947	0.808333333
LRRC2	0.881578947	1.065789474
tctex1d2	0.881578947	1.446808511
rfc3	0.881443299	0.95505618

hmgb1b	0.881421027	1.230623819
rasl11a	0.881355932	0.712121212
yars	0.881355932	1.614754098
ftr15	0.881355932	1.8
dcaf13	0.881278539	0.919642857
c3b.2	0.881081081	0.956331878
si:ch211-188f17.1	0.880952381	0.5
CABZ01089777.1	0.880952381	0.548387097
si:dkey-191g9.7	0.880952381	0.625
raver1	0.880952381	0.828571429
bmpr1ab	0.880952381	0.889705882
atg2b	0.880952381	0.958333333
prkag2a	0.880952381	1.013513514
arpp19b	0.880952381	1.033980583
CR847847.1	0.880952381	1.12195122
uqcc1	0.880952381	1.241610738
lmo7a	0.880851064	1.043243243
pawr	0.880794702	1.310810811
cntn1b	0.880733945	0.404761905
seph_1	0.880733945	0.824267782
ptpn22	0.880597015	0.825
fcho2	0.880597015	0.861788618
mcm3	0.880487805	0.827763496
apobec2a	0.880487805	1.030732861
spns2	0.880434783	0.890625
ildr1b	0.880434783	1.166666667
RAB35 (1 of many)	0.880434783	1.333333333
si:ch73-61d6.3	0.88034188	0.649484536
fdft1	0.88034188	0.685714286
tada3l	0.880149813	1.37012987
rpn1	0.880145278	0.573369565
kif23	0.88	0.914634146
golga3	0.88	0.971428571
aox6	0.88	1
ADAMTS7	0.88	1.157894737
znf511	0.88	1.157894737
thap3	0.88	1.235294118
brf1a	0.88	1.277777778
PIK3C2G	0.88	1.461538462
dnmt3bb.3	0.88	1.55
adal	0.88	1.5625
GTPBP8	0.88	1.608695652
si:dkey-23f9.4	0.88	1.647058824
ust	0.88	1.657142857
arhgap4b	0.88	0.702380952
qpctla	0.88	0.797101449
si:dkeyp-106c3.1	0.88	0.875
NCSTN	0.88	0.916666667
atad5a	0.88	0.947368421
gatb	0.88	1.350877193
si:ch211-157b11.12	0.88	2.175
irx3a	0.87987988	0.77394636
aup1	0.879858657	1.05511811
adoa	0.879781421	1.106918239
si:ch73-22o12.1	0.879746835	1
si:ch211-251b21.1	0.879745447	1.097303371
myl4	0.879736409	1.295795796
rorab	0.879712747	1.00486618
styk1	0.879699248	1.885416667
rn7sk	0.879518072	1.012195122
tmem108	0.879518072	1.015384615

ppm1ba	0.879518072	1.016949153
arih1	0.879464286	1.072847682
plxnb1b	0.879432624	0.69
smarcd3b	0.879396985	0.68125
rorca	0.879396985	0.834285714
zic3	0.879365079	1.218009479
plk4	0.879310345	0.591836735
grem2b	0.879310345	0.659574468
dot1l	0.879310345	0.780487805
MINOS1-NBL1	0.879310345	0.817391304
ubtf	0.879310345	0.7578125
si:ch211-262i1.6	0.879227053	1.516556291
cd248a	0.879194631	0.79109589
snx1a	0.879166667	0.963010204
zgc:113436	0.879120879	1.194029851
gna15.1	0.879120879	1.542372881
si:dkeyp-73b11.8	0.878875969	1.117179742
timm8b	0.878824969	0.939172749
GFOD1	0.878787879	0.595238095
wdfy4	0.878787879	0.783783784
dnm3a	0.878787879	0.851851852
ctps1b	0.878787879	0.96656535
pnp6	0.878787879	1.102564103
zgc:162939	0.878787879	1.115384615
CABZ01054394.1	0.878787879	1.259259259
atxn10	0.878787879	1.32
tnnt2c	0.878787879	1.40776699
zgc:174164	0.878787879	1.535714286
si:dkeyp-67f1.2	0.878787879	2.45
apba2a	0.878787879	0.52238806
iffo1b	0.878787879	0.746268657
pard3	0.878787879	0.922077922
tmtc1	0.878787879	1.275862069
pak2a	0.878706199	0.8802589
map7d2b	0.878698225	0.832669323
srsf9	0.878640777	0.751256281
surf2	0.878612717	1.379032258
smtnb	0.878571429	0.87
dcun1d3	0.878504673	0.816091954
si:ch211-160d20.3	0.878504673	0.954545455
idh3a	0.878365831	1.415509259
pnn	0.878277154	0.940149626
serpinb1l3	0.87826087	0.720194647
gem	0.87826087	0.760330579
gpm6bb	0.87826087	0.773480663
shc1	0.87826087	0.888297872
quo	0.87826087	0.903846154
luzp2	0.878205128	1.242990654
ela3l	0.878201777	1.195589645
MPC2 (1 of many)	0.878186969	0.938718663
ywhaz	0.878064727	1.141391941
ric3b	0.87804878	0.333333333
parpbp	0.87804878	0.75862069
meig1	0.87804878	1.075471698
cratb	0.87804878	1.078947368
HSPB11	0.87804878	1.576923077
stt3a	0.87804878	0.739930955
ankrd10b	0.87804878	0.877192982
akap7	0.87804878	1.129032258
GPATCH2	0.87804878	1.202702703
bcap31	0.878003697	1.27926078

AC024175.11	0.877996229	0.842896517
si:dkey-56f14.7	0.877984085	1.494505495
col11a1b	0.877973113	0.854666667
tusc5a	0.877952756	0.352331606
phyh	0.877934272	1.315789474
nap1l4b	0.877922078	1.828451883
isl1	0.877894737	0.809815951
asf1bb	0.877828054	1.038709677
clns1a	0.877813505	0.813953488
MDP1	0.877777778	0.908163265
scn1bb	0.877729258	0.607407407
si:ch211-93f2.1_2	0.877679697	0.947761194
itgb1b.1	0.877629063	1.178010471
zgc:193790	0.87755102	0.507936508
tesk2	0.87755102	0.95890411
abcd4	0.87755102	1.348837209
TSTD2	0.87755102	1.4375
si:ch211-264f5.6	0.87755102	1.498007968
cxxc1a	0.87745098	1.288732394
lix1	0.877419355	0.858895706
katnb1	0.877419355	0.965517241
aldh3a2a	0.877419355	1.495726496
zgc:113149	0.877358491	0.547368421
kif4	0.877358491	0.724137931
supt3h	0.877358491	0.991596639
CU695232.1	0.877358491	1.22
alcama	0.877358491	0.922131148
ltbp1_1	0.877300613	0.927419355
exoc8	0.877300613	1.270491803
CABZ01079024.1	0.877192982	0.595238095
lcor	0.877192982	0.65625
soat2	0.877192982	0.825396825
gla	0.877192982	1.074074074
mtr	0.877192982	1.2125
pank1b	0.877192982	1.280952381
chmp5b	0.87716263	1.714611872
arf6b	0.877138414	1.144761905
cct4	0.877107531	0.765098185
si:ch211-209p16.4	0.877083333	1.611528822
ikbkg	0.87704918	1.401234568
celf1	0.877005348	1.217391304
plppr1	0.876957494	0.941644562
sf3b5	0.87694704	0.93133583
lrfn4b	0.876923077	0.975903614
rasgrp4	0.876923077	1.422222222
eral1	0.876923077	1.465753425
ptpreb	0.876811594	1.155555556
smarca5	0.876785714	0.686230248
sv2bb	0.876767677	0.861736334
kiaa0101	0.876712329	0.596491228
rangap1a	0.876712329	0.748329621
crabp1a	0.876595745	0.778947368
lhfpl3	0.876569038	1.544585987
kif2c	0.87654321	0.983050847
si:dkey-23i12.5	0.87654321	1.68
csnk1a1	0.876536611	1.207332491
mgat5	0.876470588	0.826446281
her15.1	0.876470588	1.596774194
park7	0.876456876	1.181425486
hibadhb	0.876404494	1.075971731
tfap2d	0.876404494	0.56

spsb4b	0.876404494	0.861111111
bcl7ba	0.876404494	0.95890411
si:dkey-31c13.1	0.876404494	1.168831169
nol7	0.876404494	1.386861314
rgmb	0.876344086	0.656976744
coq5	0.876344086	1.473282443
rock1	0.87628866	0.969465649
polr3f	0.87628866	1.233333333
zgc:113263	0.87628866	1.428571429
ddx3a	0.876258993	1.041431262
narf	0.876146789	1.316939891
coro1b	0.876080692	1.071428571
pdlim2	0.876	1.126582278
ift46	0.875968992	1.15
hbs1l	0.875862069	1.273584906
si:dkey-74k8.3	0.875789474	1.148148148
adnpb	0.875706215	1.12195122
srsf10a	0.875647668	1.328244275
si:ch211-156b7.4	0.875527426	0.917021277
syngr3a	0.875432526	1.041935484
plscr3b	0.875406284	1.058263971
mmp2	0.875083056	1.03917221
si:ch73-127m5.1	0.875	0.386792453
cdh18a	0.875	0.48
f2r	0.875	0.608695652
cga	0.875	0.631578947
E2F1	0.875	0.72
rsg1	0.875	0.897435897
znf1040	0.875	0.928571429
msantd4	0.875	0.9375
MIPEP (1 of many)	0.875	0.963963964
parp4	0.875	1.023255814
arl15b	0.875	1.075471698
si:ch211-69l10.2	0.875	1.1
phactr2	0.875	1.183098592
ACVR2B (1 of many)	0.875	1.244680851
adgrl4	0.875	1.333333333
ago3b	0.875	1.357142857
PMM1	0.875	2.761904762
impa2	0.875	0.428571429
gpr161	0.875	0.885714286
stard8	0.875	0.982758621
RNF5	0.875	0.992307692
myod1	0.875	1.205298013
dnajc11b	0.875	1.452380952
mpdu1b	0.875	1.63
zgc:92161	0.875	2.194444444
ldlrad2	0.875	0.333333333
htr2cl1	0.875	0.7
chst3b	0.875	0.75
neil3	0.875	0.888888889
CYSTM1 (1 of many)_1	0.875	0.900343643
znf574	0.875	0.901960784
slc3a1	0.875	0.92
srbd1	0.875	1.028571429
fzd9a	0.875	1.133333333
sstr5	0.875	1.149253731
znf569l	0.875	1.157894737
guca1d	0.875	1.166666667
si:ch211-133l11.10	0.875	1.2
vgll4a	0.875	1.238095238

si:ch73-54f23.4	0.875	1.3
FAM20C (1 of many)	0.875	1.3125
ccdc127a	0.875	1.5
oard1	0.875	2.076923077
cdk20	0.875	2.391304348
myh9b	0.87483531	0.919520548
hmgb2a	0.874789309	1.090147921
krt91	0.87472414	0.777746569
zgc:112982	0.874617737	1.225
rbm39b	0.874587459	0.901459854
CABZ01102632.1	0.874538745	0.675
ywhae2	0.874239351	0.922330097
itgb1a	0.874233129	0.907826087
sars	0.874157303	1.145228216
pex14	0.874074074	1.782608696
arid3b	0.874015748	0.751091703
si:dkey-199f5.8	0.874015748	1.380116959
ccnt2a	0.87398374	1.142857143
kif5ba	0.87394958	1.091295117
tbl3	0.873913043	0.792682927
hnrnpua	0.873913043	1.213414634
si:dkey-93h22.8	0.873873874	0.676190476
ccnt1	0.873786408	0.542168675
fam192a	0.873720137	1.023569024
mark2b	0.873684211	0.923664122
nolc1	0.873684211	0.618881119
zeb2a	0.873626374	0.723320158
ahctf1	0.873626374	0.889705882
kif20a	0.873563218	0.611940299
ccsapa	0.873563218	0.844827586
pcna	0.87352381	0.943961016
nxn	0.873517787	0.70212766
capn3b	0.873469388	1.145098039
ppil3	0.873417722	1.243243243
bfsp2	0.873357228	1.23880597
samsn1a	0.873333333	1.220183486
upf3a	0.873303167	0.940740741
pcdhb	0.873239437	0.897435897
ap1g2	0.873239437	0.943925234
mtus1b	0.873239437	1.072222222
bmp5	0.873239437	1.286821705
sh3d21	0.873188406	1.387434555
ptbp3	0.873170732	0.835294118
med16	0.873015873	0.729166667
baiap2l1a	0.873015873	1.72
DYRK3	0.872964169	0.939252336
strip1	0.872852234	1.023622047
ppm1e	0.872727273	0.659793814
prr5a	0.872727273	1
si:ch211-225o7.1	0.872727273	1.023809524
epha2a	0.872727273	1.054347826
tbrg1	0.872727273	1.259259259
si:ch211-191i18.2	0.872727273	1.366666667
strn3	0.872685185	1.14057508
mmp14b	0.872685185	1.284210526
eno1b	0.872670807	0.49490835
rmdn1	0.872628726	1.183006536
tmem209	0.872611465	1.20661157
mapk1	0.872586873	1.231707317
dip2cb	0.87254902	0.642857143
cnn3a	0.872536137	1.53765324

seh1l	0.872340426	1.309392265
matn3b	0.872340426	1.552083333
pde1a	0.872340426	0.384615385
si:ch1073-174d20.1	0.872340426	0.581395349
asb6	0.872340426	0.784313725
si:ch211-13f8.1	0.872340426	0.892857143
fmnl3	0.872340426	1.014492754
med21	0.872340426	1.085106383
pon1	0.872340426	1.428571429
plekha1b	0.872340426	1.580645161
sf3b3	0.872294372	0.718670077
slc12a7b	0.872262774	0.90403071
osbpl9	0.872246696	1.011695906
mibp	0.872246696	1.278688525
TSTA3 (1 of many)_2	0.872222222	1.569105691
ecd	0.872093023	0.964285714
ddx28	0.872093023	1
actn1	0.872037915	0.82585752
mcrs1	0.872037915	1.13368984
ca5a	0.872017354	1.844155844
si:dkey-205h23.1	0.87195122	0.812030075
tbc1d24	0.871921182	1.275229358
twf1b	0.871900826	1.213017751
ponzr5	0.871886121	1.112179487
zgc:158296	0.871875	1.064885496
mcm4	0.871794872	0.583333333
TIAM2 (1 of many)	0.871794872	0.705882353
AIMP1 (1 of many)	0.871794872	0.786833856
zgc:103625	0.871794872	0.851851852
jakmip1	0.871794872	1
rhogd	0.871794872	1.214285714
adra2da	0.871794872	1.290322581
uap1	0.871794872	1.295302013
CABZ01067761.1	0.871794872	1.361111111
prom2	0.871794872	1.636363636
COLEC10	0.871794872	1.681818182
lsr	0.871657754	0.907284768
scamp4	0.871657754	1.054545455
ptbp1a	0.871657754	1.092715232
vtnb	0.871601208	0.937972769
zhx3	0.871559633	0.9
serpinf1	0.871527778	1.290780142
arntl1b	0.871428571	0.698113208
uap1l1	0.871428571	1.300813008
slc25a46	0.871428571	1.38
tbc1d10aa	0.871287129	1.072463768
vkorc1l1	0.871212121	0.929133858
pdcd6	0.871212121	1.0626703
dnaja3a	0.871080139	0.972440945
gnaia	0.870967742	1.328282828
graf4b	0.870967742	0.464285714
slc22a17	0.870967742	0.64
si:dkey-280e21.3	0.870967742	0.717948718
aldh16a1	0.870967742	0.722222222
si:ch211-276i12.4	0.870967742	0.782608696
wrap73	0.870967742	0.893939394
gbp2	0.870967742	0.914285714
mdn1	0.870967742	0.923076923
stoml1	0.870967742	1.084745763
si:cabz01071911.4	0.870967742	1.155555556
tmprss13b	0.870967742	1.223880597

tmub2	0.870967742	1.185185185
ubr1	0.870967742	1.285714286
gstm.3	0.870967742	1.302702703
prrg1	0.870786517	1.340206186
stk38l	0.870786517	1.623762376
ppfibp2a	0.870748299	1.598360656
gng5	0.870707071	1.461922597
aldocb	0.870643432	0.661525278
hnrnpul1	0.870624049	1.223234624
EMILIN3	0.870588235	0.797101449
nav2a	0.870588235	0.907407407
neu1	0.870588235	1.34375
si:ch211-198n5.11	0.870588235	1.441176471
zc3h7a	0.870588235	1.488
iqsec1b	0.870503597	0.542056075
dgat1b	0.870503597	1.3
6-Sep	0.870466321	1.096551724
sdpra	0.870410367	1.063131313
cst3	0.870401811	1.977410947
RGR (1 of many)	0.87037037	0.772307692
si:ch211-153j24.3	0.87037037	0.8
ube2g1a	0.870292887	1.363636364
ldb2b	0.87027027	0.65625
snrpd3	0.87027027	1.233146067
slc24a2	0.87012987	0.296296296
nav1b	0.87012987	0.488372093
arhgap19	0.87012987	0.578313253
camk1db	0.87012987	0.895833333
oplah	0.87012987	0.912408759
trmt1	0.87012987	1.122807018
lrpprc	0.870106762	1.017156863
nutf2l	0.870098039	0.711790393
cbx3a	0.870056497	1.090163934
trim55b	0.87	1.127272727
fkbp9	0.869976359	1.06031746
rbm6	0.869863014	0.847826087
krr1	0.869822485	1.420689655
ubac1	0.86971831	1.865771812
si:dkey-253a1.2	0.869565217	0.434782609
c1ql4a	0.869565217	0.578947368
znf644b	0.869565217	0.75
gucy2f	0.869565217	0.941176471
hoxd12a	0.869565217	1.15
cep57l1	0.869565217	1.186046512
si:ch211-223a10.1	0.869565217	1.214285714
hdac3	0.869565217	1.340517241
si:ch73-110p20.1	0.869565217	1.653846154
snap23.2	0.869565217	1.739130435
CCDC134 (1 of many)	0.869565217	0.621621622
glyctk	0.869565217	0.72611465
pkn3	0.869565217	0.803921569
ankrd49	0.869565217	0.85
glud1a	0.869565217	0.954856361
kbtbd8	0.869565217	1.183673469
tia1l	0.869565217	1.252468265
sdf2	0.869565217	1.292134831
ptenb	0.869463869	0.876712329
naa40_1	0.869436202	0.946428571
hars	0.869431644	1.085714286
tnikb	0.869281046	0.962264151
myot	0.869281046	1.231884058

fundc1	0.869281046	1.645833333
rrbp1b	0.869266055	0.910480349
sphk2	0.869230769	1.069767442
tcf3b	0.869209809	0.896153846
tmem62	0.869158879	0.825396825
afap1	0.869158879	0.921052632
TIFA	0.869047619	1.384313725
cldna	0.869047619	1.119047619
golph3l	0.869047619	1.469387755
si:dkey-253d23.4	0.869047619	1.798245614
aldh1l1_2	0.869009585	1.289370079
slc6a15	0.868965517	0.53125
mafaa	0.868965517	0.701388889
lye	0.868913858	1.542709232
osbp2	0.868852459	0.670731707
CR381599.1	0.868852459	1.292307692
si:ch211-152c2.3	0.86878453	0.980169972
nt5c2b	0.868766404	1.419475655
cog1	0.868686869	1.014084507
tma16	0.868686869	1.194805195
alpi.2	0.868686869	1.942307692
vaspb	0.86862442	0.927404719
vip	0.868613139	1.121428571
cfdp1	0.868613139	1.179245283
si:dkey-27c15.3	0.868421053	1.05
SSH3	0.868421053	1.428571429
ube2d1b	0.868421053	1.51241535
zgc:110699	0.868421053	1.583333333
idua	0.868421053	1.7
grb2a	0.868421053	1.816326531
capns1a	0.868363636	1.258162491
anapc11	0.86835443	1.120204604
lrrc58b	0.868217054	1.131078224
ccz1	0.868217054	1.627218935
pdzrn4	0.868131868	0.472727273
lias	0.868131868	1.082872928
gsna	0.868131868	1.056206089
si:ch211-265g21.1	0.868089234	1.315736041
ank2b	0.868085106	0.625899281
mlpha	0.868055556	1.151079137
nsrp1	0.868055556	1.475409836
arl14ep	0.868020305	1.19939577
capn2b	0.868	1.013953488
hhla2a.1	0.868	1.052173913
si:ch73-152o7.1	0.867924528	1.066666667
bscl2	0.867924528	1.641025641
wdr89	0.867924528	0.833333333
ppm1j	0.867924528	0.923076923
tspan2b	0.867924528	0.986111111
etv2	0.867924528	1.25
tgfa	0.867924528	1.484848485
A2ML1 (1 of many)_8	0.867768595	1.004830918
serpinf2b	0.867758186	1.005
fzd8a	0.867724868	0.497326203
cars	0.86770428	1.191011236
prpf38a	0.86770428	1.566326531
SIPA1	0.867647059	0.288888889
CABZ01088149.1	0.867647059	1.016666667
mul1b	0.867647059	1.065217391
fkbp1ab	0.86746988	1.562091503
crk	0.86746988	1.238095238

si:ch73-290k24.6	0.86746988	0.413793103
tmem176l.2	0.86746988	0.767676768
cxl34b.11	0.867424242	1.591324201
bcat2	0.86741573	1.1966759
apmap	0.867374005	0.990066225
cpne4a	0.867346939	0.338028169
ptcd1	0.867346939	1.090909091
hnrnpa0l_2	0.867346939	1.362318841
zgc:103692	0.867346939	1.8
erbb3a	0.867256637	1.171052632
mpp7a	0.867256637	1.218390805
uba5	0.867256637	1.522012579
cnpy1	0.867256637	1.709090909
gdpd1	0.867208672	1.155462185
prdx6	0.867149758	1.024234694
si:ch73-25f10.6	0.867132867	1.181818182
emd	0.867088608	1.427480916
si:dkey-87o1.2	0.867088608	2.242990654
ryk	0.867021277	0.853107345
adsl	0.867005076	1.147428571
si:ch211-127l15.5	0.86695279	1.803030303
esrra	0.866935484	1.217647059
gnmt	0.866930171	1.3758967
srsf3a_1	0.866883117	0.863914373
ncalda	0.866834171	1.6816609
lim2.4	0.866820631	0.927272727
GABRR1 (1 of many)	0.866666667	0.454545455
SORCS3	0.866666667	0.526315789
gabrb2	0.866666667	0.581632653
cept1a	0.866666667	0.666666667
bag4	0.866666667	0.723684211
erap1b	0.866666667	0.75
tkfc	0.866666667	0.767123288
lrfn4a	0.866666667	0.840909091
zgc:174314	0.866666667	0.846153846
ftsj2	0.866666667	0.923076923
bach2a	0.866666667	1
wasb	0.866666667	1
supv3l1	0.866666667	1.042253521
fbxo10	0.866666667	1.130434783
klhl36	0.866666667	1.194444444
arhgap10	0.866666667	1.355769231
tmem41aa	0.866666667	1.363013699
atrnl1a	0.866666667	1.428571429
si:ch211-14k19.8	0.866666667	1.485714286
fev	0.866666667	1.535714286
si:dkey-23a23.3_1	0.866666667	1.727272727
chmp4bb	0.866587957	1.80704698
defbl1	0.866560763	1.047160731
id2a	0.866412214	1.091836735
arl4ca	0.866396761	1.218085106
ddx24	0.86631016	0.675799087
mtmr1a	0.866197183	0.988095238
dhrs4	0.866108787	1.162895928
six6b	0.866071429	1.005952381
pex3	0.866071429	1.271604938
si:ch211-197h24.9	0.866071429	1.55
calr3a	0.866019417	1.340650407
pde6a	0.865979381	1.044247788
si:dkeyp-77h1.4	0.865853659	0.428571429
SBK1	0.865853659	0.507246377

hdac9b	0.865853659	0.632653061
ppp1r3ca	0.865853659	0.75
yes1	0.865853659	0.869565217
xpr1a	0.865814696	0.452914798
xrcc1	0.865771812	1.045454545
fam46ab	0.865671642	0.914893617
llph	0.865671642	1.397321429
oraov1	0.865671642	1.975609756
slc7a2	0.865671642	0.913907285
smurf2	0.865671642	1.130081301
wdr82	0.865625	1.017006803
cryzl1	0.865546218	1.226415094
cmpk	0.865504359	1.225710015
lgals3b	0.865470852	1.021830986
paqr6	0.865384615	1.328205128
rab18a	0.865384615	1.43373494
zgc:158376	0.865384615	0.423076923
CABZ01088700.1	0.865384615	0.584615385
otofa	0.865384615	0.722222222
gcnt7	0.865384615	1.5
fbxo30a	0.865384615	1.96
bmf2	0.865384615	2.032258065
pitpnaa	0.865263158	1.155507559
fgfrl1a	0.865248227	0.833333333
slc35e3	0.865248227	1.45631068
si:dkey-191g9.5	0.865168539	0.807017544
pfn2	0.865086085	0.840901771
alpi.1	0.865079365	0.781512605
tdg.1	0.865030675	1.043478261
zgc:136410	0.865013774	0.883223684
xiap	0.864988558	1.103296703
kpna2	0.864978903	0.972789116
tecrb	0.864932886	0.873965041
ankrd13b	0.864864865	0.482758621
gas2l3	0.864864865	0.789473684
nck2b	0.864864865	0.921348315
tfe3a	0.864864865	0.925925926
ano5a	0.864864865	1
orai2	0.864864865	1.035714286
mafga	0.864864865	1.176470588
si:dkey-153m14.1	0.864796722	0.94058967
tmem33	0.864788732	0.77258567
fbn2b_2	0.864754098	1.073684211
dkk3b	0.864705882	0.729411765
dync1li2	0.864705882	1.196078431
acaa2	0.864693446	0.944769874
ptpn2b	0.864628821	1.125874126
nle1	0.864583333	1
si:dkeyp-110e4.6	0.864583333	1.293103448
mpzl2b	0.864583333	1.416666667
dynlt3	0.864583333	1.047008547
serpina1l	0.864561818	0.869047619
cyfip1	0.86453202	1.049707602
eya4	0.864485981	0.937219731
kdm2ba	0.864468864	0.482587065
ctsl.1	0.86440678	1.449392713
smpd5	0.86440678	1.027522936
cobll1b	0.864285714	0.945736434
arpin	0.864285714	1.861111111
celf3b	0.864253394	0.917197452
nptna	0.864211738	0.909509202

slc6a6b	0.864197531	1.091743119
unkl	0.864197531	1.152380952
gas8	0.864197531	2.4
nmrk2	0.86416185	2.315384615
nansa	0.864150943	1.188053097
dmgdh	0.864142539	0.865603645
dph7	0.86407767	1.25
sec63	0.864035088	1.335877863
jam3b	0.864022663	1.502347418
rbm4.2	0.863970588	0.604743083
stk38a	0.863945578	1.663157895
guk1b	0.86381323	0.842342342
BX908782.1	0.863772455	1.072524407
rasal2	0.863636364	0.593495935
si:ch211-120p12.3	0.863636364	1.12195122
SGMS1 (1 of many)	0.863636364	1.277227723
ugcg	0.863636364	1.6625
kcnh6a	0.863636364	0.590909091
si:ch211-255f4.5	0.863636364	0.62962963
edil3a	0.863636364	0.668367347
myh14	0.863636364	0.816326531
hrh3	0.863636364	0.875
CR450842.2	0.863636364	0.875
neto1	0.863636364	0.941176471
pih1d3	0.863636364	1.19047619
nlrx1	0.863636364	1.222222222
pcdh1gc5	0.863636364	1.416666667
odf2a	0.863636364	1.5
dkk1a	0.863636364	1.611111111
gtpbp6	0.863636364	1.739130435
rab11fip4b	0.863636364	2.153846154
chmp2bb	0.863560732	1.612903226
sar1b	0.863406408	1.027707809
trappc2l	0.863387978	1.407142857
tp53inp2	0.863354037	1.115384615
wls	0.863328823	0.817189632
gatad2ab	0.863247863	0.725274725
hmcn2_1	0.863247863	1.06097561
zgc:55582	0.863247863	1.119047619
dbn1	0.863192182	0.603448276
spp1	0.863157895	0.988888889
pttg1ipb	0.863157895	1.298076923
puf60a	0.863128492	0.925531915
twistnb	0.863013699	0.820224719
si:ch211-126i22.5	0.863013699	1.052631579
mlx	0.863013699	1.863636364
tango2	0.863013699	1.875
pctp	0.862944162	0.923566879
tmem258	0.862908838	0.909898477
atf6	0.862903226	0.945783133
yaf2	0.862903226	1.477419355
zgc:101858	0.862876254	0.925566343
gpr160	0.862745098	0.5
zgc:112998	0.862745098	0.885714286
zdhhc5b	0.862745098	1
MYPN	0.862745098	1.085714286
ugt1b2	0.862745098	1.18
si:dkey-188i13.11	0.862745098	1.016778523
rdh10a	0.862676056	1.106575964
hmga1a	0.862639338	0.813098346
znf362a	0.86259542	0.954954955

map1sb	0.8625	0.652173913
arhgap42a	0.8625	0.784615385
hpgd	0.862433862	1.471428571
cisd1	0.862359551	1.243801653
ranbp1	0.862318841	1.291666667
btbd6a	0.862318841	0.606837607
plrg1	0.862318841	0.921487603
hbbe2	0.862149533	0.56789623
apoeb	0.862135063	0.964946211
si:dkey-73n10.9p	0.862068966	0.5
si:dkey-283b15.2	0.862068966	0.617647059
fam58a	0.862068966	0.984848485
pak6b	0.862068966	1
si:dkey-57k17.1	0.862068966	1.208333333
mettl22	0.862068966	1.216216216
GLB1L2	0.862068966	1.238095238
opn1sw1	0.862038419	0.858704929
cyth1a	0.861904762	1.248447205
cx43	0.861848124	1.011494253
fam60al_2	0.861842105	1.081081081
anp32b	0.86163522	0.767757382
LRRC4C	0.861538462	0.515151515
zfpm2b	0.861538462	0.673913043
apba1a	0.861538462	0.684210526
cbln2b	0.861538462	0.780104712
crtc3	0.861538462	0.914893617
si:zfos-932h1.2	0.861538462	0.963414634
ncaldb	0.861538462	1.339285714
limch1b	0.861386139	0.93902439
arhgef12b	0.861313869	1.054945055
ldb2a	0.861313869	1.111111111
s100v1	0.861303745	1.070484581
mtap	0.861209964	1.568720379
hmgn2	0.861203854	1.179469671
ankrd33aa	0.861111111	0.733333333
si:dkey-181m9.8	0.861111111	0.756756757
lamb2l	0.861111111	0.784482759
tyw1	0.861111111	0.916666667
atxn3	0.861111111	0.938461538
si:ch73-204m7.3	0.861111111	0.939393939
parietopsin_1	0.861111111	0.947368421
stag1a	0.861111111	1.026666667
CASKIN2	0.861111111	1.075757576
mvk	0.861111111	1.084745763
si:ch1073-268j14.1	0.861111111	1.111111111
adamts8a	0.861111111	1.133333333
si:dkey-165a24.7	0.861111111	1.264150943
zgc:113223	0.861111111	1.34
pparda	0.861111111	1.428571429
zbtb22b	0.861111111	1.5
si:dkey-17m8.1	0.861111111	1.534883721
bloc1s3_1	0.861111111	1.583333333
si:dkeyp-115e12.6	0.861111111	1.631578947
zgc:92360	0.861003861	0.95112782
cebpz	0.861003861	1.309090909
cdk4	0.860927152	1.078740157
zgc:162707	0.860869565	0.689655172
zgc:136336	0.860869565	0.782608696
phldb2b	0.860869565	1.164556962
stx12l	0.860869565	1.355263158
cul2	0.860805861	1.190954774

si:dkey-48g21.7	0.860759494	2.105263158
ugt1ab	0.860696517	1.555555556
mansc1	0.860576923	0.604278075
zgc:194930	0.860465116	1.018181818
afap1l1a	0.860465116	1.178082192
stx2b	0.860465116	1.705882353
tbl1xr1b	0.860465116	0.882352941
arid3a	0.860465116	1.056179775
plp2	0.860349127	1.214175655
si:dkey-262j3.7	0.860240964	0.771084337
her6	0.860215054	1.058962264
si:ch73-91k6.2	0.860215054	1.824561404
gpn2	0.86013986	1.030674847
clic2	0.86013986	1.034482759
slc17a7a	0.860103627	0.724637681
slc2a1b	0.860103627	1.547169811
cart2	0.86	0.698795181
si:dkeyp-51b9.3	0.86	0.921052632
gpsm1b	0.86	1.040540541
lcorl	0.86	1.21875
prkacba	0.86	1.341463415
gabpb2b	0.86	1.84
tmem176l.4	0.859969559	1.379310345
zmiz2	0.859813084	0.921875
ap1m2	0.859756098	0.875968992
dhodh	0.859756098	1.104895105
acin1a	0.859728507	0.985714286
akap1b	0.85971223	0.889908257
lipf	0.859706362	1.138755981
adcy6b	0.859649123	0.888888889
cep131	0.859649123	1.05
cdk15	0.859649123	1.4375
znf1144_1	0.859649123	1.52173913
pes	0.859615385	1.167800454
spg21	0.859550562	1.405063291
zbtb17	0.859504132	0.926829268
me1	0.859504132	1.088235294
snx12	0.859459459	1.591836735
igsf21b	0.859459459	1.211267606
arl6ip4	0.859459459	1.464566929
VWA5A (1 of many)_1	0.859437751	0.864035088
csde1	0.859389454	1.236
fars2	0.859375	0.741935484
si:ch73-311h14.2	0.859375	0.873684211
SLC6A7	0.859375	1.038461538
slc37a2	0.859375	1.226950355
lpar6b	0.859375	2.421052632
chd2	0.859223301	0.797385621
gfod2	0.85915493	0.833333333
sypl1_1	0.85915493	1.163636364
pm20d1.2	0.85915493	1.270833333
rabl3	0.85915493	1.588235294
arpc5a	0.859116022	1.558558559
rbms2a	0.858974359	0.985294118
cdk10	0.858974359	1.013157895
ankrd40	0.858974359	1.037735849
CABZ01052290.1	0.858974359	1.547945205
ptcd3	0.858942065	1.488970588
si:ch211-201h21.5	0.858823529	0.918367347
grnb	0.858823529	0.960629921
crtap	0.858769932	1.159891599

scaf8	0.858695652	0.528409091
taok3b	0.858695652	0.591836735
ptk2aa	0.858695652	0.890625
pdca	0.858695652	1.583333333
fabp11a	0.858646617	1.044600939
ugt1b5	0.858638743	1.017045455
KIF1C	0.858585859	1.384615385
spint2	0.858571429	1.221162186
si:ch211-221f10.2	0.858490566	1.409638554
hsd17b12b	0.858433735	1.370656371
arrb2b	0.858333333	0.875
larp4aa	0.858237548	0.621890547
snap25a	0.858212241	0.948246118
ddr1	0.858208955	0.989473684
pltp	0.858156028	1.090909091
cdv3	0.858131488	0.978540773
si:dkey-66a8.7	0.858108108	1.038709677
ube2b	0.858064516	1.245283019
si:ch211-156j16.1	0.858044164	1.318763326
ube2g1b	0.857988166	1.260683761
med29	0.857819905	1.190909091
pdss1	0.857798165	1.05
wrb	0.857677903	1.519417476
rbbp4	0.857627119	1.093350384
calr	0.857482185	1.757142857
neb	0.857279693	1.302030457
flt4	0.857142857	0.597014925
mgat4c	0.857142857	0.617647059
fam155a	0.857142857	0.647058824
mpnd	0.857142857	0.680555556
plxdc1	0.857142857	0.705882353
g6pc3	0.857142857	0.777777778
abtb2b	0.857142857	0.863636364
itpr1a	0.857142857	0.869565217
mcf2lb	0.857142857	0.891304348
fitm1	0.857142857	0.932330827
si:busm1-71b9.3	0.857142857	1
vps52	0.857142857	1.133333333
mtbp	0.857142857	1.277777778
si:ch211-63o20.7	0.857142857	1.307692308
si:dkey-14k9.3	0.857142857	1.342857143
dbf4b	0.857142857	1.363636364
znf1120	0.857142857	1.461538462
CU467856.1	0.857142857	1.47826087
si:cabz01112025.1	0.857142857	1.636363636
drc1	0.857142857	1.642857143
tbc1d14	0.857142857	1.666666667
RETSAT (1 of many)	0.857142857	2
si:dkey-154b15.1	0.857142857	2.8
faxca	0.857142857	0.384615385
cacna1c	0.857142857	0.586206897
si:ch1073-358o18.3	0.857142857	0.602409639
chrm4a	0.857142857	0.609375
onecut2	0.857142857	0.645833333
tmem178	0.857142857	0.669902913
wdr44	0.857142857	0.670634921
nck2a	0.857142857	0.714285714
slc1a1	0.857142857	0.716535433
b3gnt9	0.857142857	0.791666667
afap1l1b	0.857142857	0.833333333
sirt7	0.857142857	0.846153846

si:ch211-63p21.1	0.857142857	0.857142857
stoml3a	0.857142857	0.875
thrab	0.857142857	0.890625
vegfc	0.857142857	0.916666667
GGT5 (1 of many)	0.857142857	0.928571429
si:ch73-341k19.1	0.857142857	0.941176471
rft1	0.857142857	0.941176471
kdrl	0.857142857	0.979381443
ifrd2	0.857142857	0.996
si:ch211-150o23.2	0.857142857	1
ak5l	0.857142857	1.02173913
arhgef37	0.857142857	1.103448276
zgc:101560	0.857142857	1.103448276
colgalt1	0.857142857	1.181818182
cyp1a	0.857142857	1.210045662
ctss2.1	0.857142857	1.25
fam210ab	0.857142857	1.251612903
erbb2	0.857142857	1.272727273
tmem51b	0.857142857	1.322033898
gnai3	0.857142857	1.388235294
SLC25A34	0.857142857	1.454545455
apitd1	0.857142857	1.464285714
tmem69	0.857142857	1.556701031
mc5ra	0.857142857	1.622222222
CR812792.1	0.857142857	1.894736842
dpf2	0.857142857	2.083333333
si:ch211-152p11.4	0.857142857	2.181818182
si:dkey-20i20.3	0.857142857	2.3
zgc:163143	0.857142857	2.538461538
znf1158	0.857142857	2.727272727
dhrs13a.1	0.856557377	1
hccsb	0.856382979	1.063973064
tmem176l.3b	0.85625	1.525423729
ldha	0.856227915	1.530985439
CABZ01087953.1	0.85620915	1.045112782
crhbp	0.856187291	1.044444444
gpatch8	0.856115108	0.951871658
qpct	0.856	0.828947368
syne3	0.855932203	1.040816327
col9a3	0.855929324	0.749503771
tmem94	0.855855856	0.954545455
ctdspla	0.855769231	1.062827225
si:ch211-180a12.2	0.855670103	0.417721519
gdf10a	0.855670103	1.092198582
atf7ip2	0.855670103	1.275362319
mtmr12	0.855555556	1.378378378
arhgef2	0.855491329	0.829268293
sftpba	0.855421687	1.363636364
col5a2a	0.85540409	0.956187093
kcnip3a	0.855371901	1.621468927
si:dkey-97i18.5	0.855263158	0.797153025
GNB4	0.855263158	1.362637363
si:dkey-36i7.3	0.85518591	1.141342756
RDH13 (1 of many)_3	0.855172414	0.87962963
cadm1a	0.855072464	0.748201439
dap3	0.855072464	0.853571429
tkta	0.855	0.862068966
zgc:77056	0.854961832	1.610169492
ccdc53	0.854961832	1.472727273
srsf7b	0.85483871	0.936507937
zgc:100920	0.85483871	1.142857143

zgc:112980	0.85483871	1.254545455
cecr2	0.85483871	1.258064516
cat	0.854781582	1.145185185
lrp12	0.854771784	1.046052632
serinc5	0.854748603	0.881818182
myoc	0.85467128	1.416666667
bscl2l	0.854651163	1.427631579
si:ch211-270g19.5	0.854545455	1.02
ccdc127b	0.854545455	1.105263158
notum1a	0.854545455	1.159574468
foxd1	0.854368932	0.791208791
sdf2l1	0.854330709	0.7
arhgef1b	0.854251012	1.480519481
snapin	0.854251012	1.711764706
COL13A1	0.854166667	0.983333333
fam184b	0.854166667	1
clcn1b	0.854166667	1.25
cyth1b	0.854166667	1.25
calhm2	0.854166667	1.277777778
cetn3	0.854166667	1.428571429
si:ch211-255f4.10	0.854166667	1.805555556
gnb3b	0.854153252	0.839380063
mcm2	0.854098361	0.670909091
gtf2e2	0.854077253	1.671428571
tspan35	0.854054054	1.280898876
zgc:77439	0.854037267	0.693181818
tspan18b	0.854014599	0.789237668
qdprb1	0.854014599	1.093457944
gria2b	0.853982301	0.93129771
tbrg4	0.853982301	1.064864865
heatr5a_1	0.853932584	0.852941176
serpind1	0.853932584	1
gpatch4	0.853932584	1.097222222
zgc:103499	0.853932584	1.269230769
impdh1b	0.853785901	0.652406417
sardh	0.853763441	0.790794979
acana	0.853658537	0.666666667
si:dkey-97o5.1	0.853658537	0.818181818
pcyt1ba	0.853658537	0.875
si:ch73-299h12.4	0.853658537	0.9375
mhc1zea	0.853658537	1.060606061
zgc:110410	0.853658537	1.346153846
trmt61b	0.853658537	1.473684211
rnf169	0.853658537	1.509803922
GPR161 (1 of many)	0.853658537	1.603174603
lyve1b	0.853658537	1.660714286
adam11	0.853658537	0.816901408
opa3	0.853658537	1.450980392
sorbs2b	0.853448276	0.755319149
triap1	0.853448276	1.542168675
eif5a2	0.853378666	1.145381121
abcc9	0.853333333	0.921875
clmn	0.853333333	1.179487179
si:dkey-226l10.6	0.853333333	1.333333333
hbae3	0.853288364	0.542662356
acads	0.853225806	1.067256637
hoxc9a	0.853211009	0.925
lrrn1	0.853211009	0.953623188
enpp2	0.853211009	1.14084507
phospho2	0.853211009	1.564705882
rbbp9	0.853211009	1.21484375

slc35f6	0.853164557	0.917525773
arntl1a	0.853146853	0.735042735
namptb	0.853107345	1.448275862
scinlb	0.853080569	0.882743363
nrap	0.853061224	1.604790419
ccdc120	0.852941176	0.5
npffl	0.852941176	0.589473684
zbtb16b	0.852941176	0.785714286
rbl2	0.852941176	0.911111111
trim35-13	0.852941176	1.033898305
st3gal3b	0.852941176	1.169811321
il17ra1a	0.852941176	1.193548387
GK3P	0.852941176	1.276836158
ccdc114	0.852941176	1.346153846
si:ch73-299h12.9	0.852941176	1.36
btc	0.852941176	1.371428571
atmin	0.852941176	1.4
hectd2	0.852941176	1.523809524
si:ch211-198p11.6	0.852941176	1.818181818
rab9b	0.852941176	2.617647059
tnc	0.852861035	0.852140078
sptlc2b	0.852842809	1.620320856
dnajc5aa	0.852747253	1.00952381
tmem18	0.852691218	1.137362637
tsr1	0.852579853	0.93452381
SLCO3A1 (1 of many)	0.852459016	0.666666667
si:dkey-183i3.9	0.852459016	0.913043478
clp1	0.852459016	1.5
METTL18	0.852459016	1.755555556
bpgm	0.852459016	1.50729927
metap2a	0.852348993	2.098901099
zgc:101566	0.852320675	1.657894737
pkdcca	0.852272727	0.594936709
fndc5b	0.852272727	0.827586207
ece1	0.852173913	0.920792079
znf296	0.852173913	1.0875
agpat4	0.852071006	0.911764706
si:ch1073-469d17.2	0.852071006	0.969863014
rlim	0.852040816	0.955223881
nr2e3	0.851851852	0.813559322
fn1a	0.851851852	0.882591093
PRDM15	0.851851852	0.636363636
brip1	0.851851852	0.68
prg4a	0.851851852	0.739130435
lrrk1	0.851851852	0.823529412
mnx2a	0.851851852	0.838709677
ppp6r3	0.851851852	1.01320132
cdc45	0.851851852	1.06122449
neurod6a	0.851851852	1.073529412
cenpk	0.851851852	1.244897959
srp19	0.851851852	1.607594937
si:ch211-181d7.3	0.851851852	1.666666667
cdca4	0.851724138	1.299145299
cbx3b	0.851724138	1.952631579
tfap2a	0.851706037	0.813780261
taf6_2	0.851694915	1.502994012
prkaa1	0.851648352	1.111940299
capn2a	0.851626016	1.080097087
podxl	0.851581509	1.557823129
nefla	0.851485149	0.973684211
si:ch211-137a8.4	0.851373183	0.713636364

zic2b	0.851351351	0.613333333
FADS6	0.851351351	0.955555556
mob1a	0.851301115	1.337606838
vsnl1a	0.85126162	0.658374793
eif4ebp3	0.851190476	0.733193277
igfbp3	0.851190476	0.923076923
si:ch211-161h7.8	0.851190476	1.2
mybpc2b	0.851138354	0.97731569
nadkb	0.85106383	0.628205128
efr3bb	0.85106383	0.882352941
cdc42se2	0.85106383	1.075268817
wnt4a	0.85106383	1.264705882
CCDC57	0.85106383	1.708333333
ywhag1	0.850896861	1.354344123
npr3	0.850877193	0.887755102
mgst1.2	0.850847458	1.361344538
higd1a	0.850775194	1.862433862
shhb	0.850746269	0.979591837
lpar3	0.850746269	1.02
isoc1	0.850746269	1.037037037
tbc1d1	0.850746269	1.084745763
itm2bb	0.850692354	1.126768534
si:dkey-267p21.2	0.850678733	0.837438424
cgref1	0.850649351	1.086956522
carm1	0.850649351	0.885558583
elp2	0.850574713	0.876712329
frya	0.850574713	0.923076923
ttl	0.850574713	1.083333333
slmapb	0.850574713	1.273504274
denr	0.850574713	1.128256513
ppp1r13l	0.850515464	1.046511628
rad51	0.85046729	1.1875
vim	0.850393701	0.738047809
mvp	0.850393701	0.645810056
nop2	0.850364964	1.440860215
ch1073-291c23.1	0.850299401	0.787985866
twf2a	0.850299401	1.30952381
mapkapk2a	0.850282486	1.335849057
vps26a	0.850282486	1.449056604
praf2	0.85026738	1.145833333
si:dkey-40c11.2	0.85	1.272277228
gpam	0.85	1.365853659
pif1	0.85	0.6
si:dkey-240h12.3	0.85	0.666666667
tmem121b	0.85	0.761904762
fzd7b	0.85	0.782051282
klhl8	0.85	0.8
suox	0.85	0.844827586
TARBP1	0.85	0.975
ercc6l	0.85	1.026315789
si:ch1073-145m9.1	0.85	1.043478261
ehd2b	0.85	1.048543689
rps6ka3a	0.85	1.119266055
eftud1	0.85	1.125
fam185a	0.85	1.152173913
zgc:152951	0.85	1.181818182
dact2	0.85	1.22
bach1a	0.85	1.28
TGFBR2 (1 of many)	0.85	1.285714286
si:dkey-185e18.7	0.85	1.326530612
zgc:101785	0.85	1.473684211

bbs12	0.85	1.5625
si:dkey-48n15.2	0.85	1.588235294
tsg101b	0.85	1.639534884
asb4	0.85	1.647058824
bsnd	0.85	1.7
eif4ea	0.85	1.737226277
si:ch211-194p6.11	0.85	1.75
si:dkey-93m18.6	0.85	1.8
si:ch211-255f4.2	0.85	2.470588235
CR407594.2	0.85	2.5
si:dkey-192d15.2	0.85	2.578947368
acox1	0.849829352	1.116504854
wu:fc46h12	0.849655172	0.848383872
pde4ba_1	0.849637681	1.325062035
cope	0.84957265	1.299892125
txnl1	0.849537037	1.016129032
kat2a	0.849462366	0.782051282
si:dkey-237p10.2	0.849462366	0.926315789
zgc:77880	0.849462366	1.2625
tacc1	0.849462366	1.555555556
si:dkey-245g22.3	0.849315068	1.183673469
fgf6a	0.849315068	1.388888889
krt97	0.849238876	0.909699971
grcc10	0.849137931	1.191588785
zgc:63568	0.849056604	0.416666667
slc1a8a	0.849056604	0.967741935
itih1	0.849056604	0.976190476
alg10	0.849056604	1.136363636
mfsd2b	0.849056604	1.333333333
hsd11b2	0.848993289	0.8101983
ficd	0.848837209	0.783333333
MPV17L	0.848837209	1.054054054
cacng8b	0.848780488	0.725925926
tomm22	0.84875	1.224222586
gpm6ba	0.848722986	0.785498489
cthrc1b	0.848684211	0.992248062
immp2l	0.848684211	1.389380531
pick1	0.848684211	2.370967742
atxn1b	0.848648649	0.8
top2a	0.848563969	0.894941634
mtmr1b	0.848484848	0.735294118
cabin1	0.848484848	0.772727273
zcchc9	0.848484848	0.865591398
ppp2r2bb	0.848484848	0.956140351
SBNO2 (1 of many)	0.848484848	1
chst14	0.848484848	1.035714286
LOX (1 of many)	0.848484848	1.189655172
smpd4	0.848484848	1.204081633
si:dkeyp-123h10.2	0.848484848	1.265486726
lrrfip2	0.848484848	1.298969072
shroom1	0.848484848	1.590909091
TMEM120A (1 of many)	0.848484848	1.642857143
musk	0.848484848	1.684210526
si:ch211-119d14.3	0.848484848	1.8
CABZ01084613.2	0.848484848	2.058823529
ube2d1a	0.848387097	1.620689655
tm9sf1	0.848275862	1.175
epn3a	0.848214286	0.977777778
bace2	0.848167539	1.212765957
si:ch211-226m16.2	0.848167539	1.89516129
rcan2	0.848101266	0.890756303

kcnj13_1	0.848101266	0.984615385
map2k7	0.848101266	1.641025641
hif1al	0.848095002	1.130356065
flnbl	0.848	0.733333333
gpr61	0.847826087	0.735294118
si:ch73-352p18.4	0.847826087	0.861111111
pparaa	0.847826087	0.947368421
pfkfb2a	0.847826087	1.25
TIMM22	0.847826087	1.325203252
mapk12a	0.847750865	1.224137931
pfn2l	0.847457627	1.458646617
gpc2	0.847457627	0.5
tnfsf10l	0.847457627	1
BX957322.1	0.847457627	1.033333333
C9orf172 (1 of many)_2	0.847457627	1.095238095
si:dkeyp-84f3.5	0.847457627	2.1875
helz	0.847389558	0.906593407
vps28	0.847328244	1.134165367
srrt	0.847222222	0.59807074
hoxb8a	0.847222222	0.961904762
fam206a	0.847222222	1.5
mbnl2	0.847107438	0.867924528
ptpn5	0.847058824	0.93877551
slc35f2	0.847058824	1.608695652
ssrp1a	0.846994536	0.939163498
spen	0.846938776	0.732142857
zgc:101663	0.846846847	0.975903614
zgc:92140	0.846774194	0.985294118
sytl3	0.846774194	1.05952381
cdk5rap3	0.846774194	1.223809524
emilin1b	0.846743295	1.349726776
ptpn9a	0.846715328	1.045977011
fam114a2	0.846715328	1.865168539
si:ch211-22i13.2	0.846441948	1.337423313
ttc36	0.846441948	1.451754386
nmt1a	0.846435101	0.89010989
cd82b	0.846335697	1.055555556
fam49ba	0.846153846	0.889502762
ppip5k2	0.846153846	0.960526316
prkag3b	0.846153846	1.111111111
si:rp71-46j2.7	0.846153846	1.803571429
CCDC89	0.846153846	0.5
efcab1	0.846153846	0.56
mn1b	0.846153846	0.637583893
hmx1	0.846153846	0.639344262
PDGFD	0.846153846	0.695652174
kif25	0.846153846	0.736842105
cox7a1	0.846153846	0.80861244
iffo1a	0.846153846	0.852941176
dcbld1	0.846153846	0.9375
gh1	0.846153846	0.951612903
fancb	0.846153846	1
znf326	0.846153846	1
si:ch73-193i22.1	0.846153846	1
si:ch73-44m9.2	0.846153846	1.058823529
hopx	0.846153846	1.059782609
adhfe1	0.846153846	1.085271318
lipin2	0.846153846	1.111702128
mtrf1	0.846153846	1.133333333
fam78bb	0.846153846	1.133333333
hbegfb	0.846153846	1.169811321

si:ch73-221f6.4	0.846153846	1.1875
daw1	0.846153846	1.210526316
rpgrip1	0.846153846	1.25
si:dkey-109j17.5	0.846153846	1.25
rc3h2	0.846153846	1.327272727
zgc:162344	0.846153846	1.333333333
zgc:113372	0.846153846	1.558823529
si:dkey-95h12.2	0.846153846	1.727272727
pigt	0.846153846	1.75
znf687a	0.846153846	1.764705882
sparc	0.846143808	0.999728298
p4hb	0.845975232	1.201853759
snapc5	0.845878136	1.896907216
tmem183a	0.845771144	1.272727273
dpf3	0.845588235	1.117021277
prkcba	0.845528455	1.088235294
fgd1_1	0.845454545	0.746478873
VDAC3 (1 of many)	0.845454545	1.195121951
serpina10a	0.845422117	1.75
sptbn1	0.845371312	0.824242424
szrd1	0.845360825	1.242718447
nuf2	0.845360825	1.692307692
kif11	0.845238095	0.934673367
pou3f3b	0.845238095	0.843450479
anln	0.845238095	0.696969697
commd1	0.845238095	0.892307692
irf6	0.845238095	1
dnajc3a	0.845238095	1.191176471
fdx1	0.845098039	1.349168646
ank3b	0.845070423	0.792792793
ttc13	0.845070423	1.028571429
si:dkey-3d4.3	0.845070423	1.060344828
zgc:153157	0.845070423	1.517241379
CABZ01030107.1	0.845070423	2.222222222
tgm1l1	0.844984802	0.933753943
rgs19	0.844827586	0.777777778
tmem120b	0.844827586	1.210526316
txnl4a	0.844827586	1.358778626
ddx19	0.844827586	1.425101215
ppp2ca	0.844789357	1.262626263
mrpl44	0.844776119	0.990353698
pex7	0.844660194	1.226890756
rbms2b	0.844660194	1.341772152
desi1a	0.844660194	1.484536082
atpif1b	0.844590884	1.409867173
zc2hc1a	0.844522968	1.661016949
cacybp	0.844512195	1.593220339
ndrg2	0.844470046	0.97108067
MEX3A	0.844444444	0.683333333
fibcd1	0.844444444	0.739130435
si:ch1073-111c8.3	0.844444444	0.81
ZNF217	0.844444444	0.820224719
syt13	0.844444444	1
orc3	0.844444444	1.073170732
TIMM21	0.844444444	1.549295775
adss	0.844285714	1.393305439
nup43	0.844155844	0.776
dus2	0.844155844	0.8875
pigr	0.844155844	1.754716981
pxk	0.844036697	1.175675676
trappc5	0.84398977	1.131034483

oxr1b	0.843971631	0.494736842
si:dkey-156n14.3_2	0.843971631	1.072164948
rell1	0.843971631	1.212765957
idh3b	0.843959732	1.158031088
aqp8a.2	0.843953186	1.373608903
igf2bp2b	0.843822844	1.210702341
CABZ01007893.1	0.84375	0.5
fanci	0.84375	0.666666667
chpfb	0.84375	0.714285714
si:ch211-285j22.3	0.84375	0.760416667
rictora	0.84375	0.909090909
lats2	0.84375	1.115702479
rnf150a	0.84375	1.25
wdr91	0.84375	1.410714286
plxnc1	0.84375	1.8
si:cabz01007812.1	0.84375	1.8125
rcc2	0.843669251	0.812600969
kri1	0.843575419	1.389830508
wisp3	0.843537415	0.9
mrc1a	0.843478261	1.96969697
smc1al	0.843462247	1.255255255
ddx39b	0.843434343	1.172413793
mrps22	0.84341637	1.697560976
CABZ01033205.1	0.843373494	0.727272727
PLOD3	0.843373494	0.873188406
furina	0.843373494	0.95
KLHL25	0.843373494	1.248062016
gpt2l	0.843342037	0.860962567
pola2	0.843283582	0.857142857
pax1b	0.843137255	0.811320755
yrk	0.843137255	0.9
USP53	0.843137255	1.051282051
vasna	0.843137255	1.17699115
emx1	0.843137255	1.317073171
gpsm2l	0.843137255	2.04
akirin1	0.843137255	1.147347741
itpkca	0.843049327	0.889534884
pdia4	0.842975207	1.074519231
myo5b	0.842857143	0.872928177
adamts17	0.842857143	1.137254902
si:ch73-376l24.3	0.842767296	1.797752809
si:ch211-217k17.9	0.842741935	2.957575758
tomm40l	0.842696629	0.551282051
srd5a3	0.842696629	0.929824561
si:dkey-17o15.2	0.842696629	1.344262295
fgd4a	0.842592593	1.112903226
si:ch1073-219n12.1	0.842519685	0.943820225
magi3a	0.842519685	1.047619048
nudt4a	0.842490842	1.421296296
msmo1	0.842424242	1.316939891
rpp14	0.842424242	1.354609929
nup62l	0.842364532	0.949868074
crx_1	0.842342342	0.391975309
pvalb8	0.842278719	1.091887046
NXPH4	0.842105263	0.4
zmynd8	0.842105263	0.40625
gdf7	0.842105263	0.666666667
BX908782.2	0.842105263	0.685393258
si:ch1073-429i10.1	0.842105263	0.78125
bokb	0.842105263	0.789473684
plek	0.842105263	0.833333333

grtp1b	0.842105263	0.84
acrc	0.842105263	0.866666667
VWDE	0.842105263	1
VWDE	0.842105263	1
slf2	0.842105263	1.034482759
hmcn1	0.842105263	1.037037037
si:ch211-62a1.1	0.842105263	1.048780488
col4a4	0.842105263	1.076923077
asb18	0.842105263	1.111111111
clec11a	0.842105263	1.12
fhl3b	0.842105263	1.1875
CABZ01071723.1	0.842105263	1.2
znf1013	0.842105263	1.333333333
pibf1	0.842105263	1.333333333
tapbp.1	0.842105263	1.416666667
spag6	0.842105263	1.705882353
hipk2	0.842105263	0.75
vclb	0.842105263	1.042857143
traf3ip2l	0.842105263	1.416666667
brd3a	0.841975309	1.308270677
emilin2b	0.841880342	1.246073298
pold3	0.841860465	1.386861314
zc3h11a	0.841849148	1.186131387
ppp4ca	0.84180791	1.117647059
pdlim4	0.841772152	0.96969697
klhl17	0.841726619	0.90625
si:ch211-132i7.2	0.841726619	1.324675325
trove2	0.841584158	1.393442623
txndc5	0.841558442	1
tulp1b	0.841463415	0.959183673
rhol	0.841463415	0.963636364
si:ch211-69g19.2	0.841423948	0.704545455
ccdc51	0.841269841	0.870503597
vps4a	0.841269841	1.492537313
rab11fip2	0.841269841	1.7
nucks1a	0.841262136	0.953302201
wdr75	0.841025641	0.822485207
sh3bp5lb	0.840909091	0.888888889
ARSJ	0.840909091	0.967741935
si:ch211-207n23.2	0.840909091	1.183333333
MRC2	0.840909091	1.230769231
PAQR9	0.840909091	1.275862069
hmgn6	0.840814051	1.327938072
slc7a10a	0.840764331	0.594520548
krt92	0.840764331	0.820504732
mrpl20	0.840707965	0.778554779
si:dkey-35i13.1	0.840707965	1.494699647
stx4	0.840677966	1.798165138
cpn1_3	0.840659341	0.921686747
fbp1b	0.840593585	1.317073171
cers4b	0.84057971	0.666666667
tnfsf12	0.84057971	0.861111111
grasp	0.84057971	0.86440678
aga	0.84057971	1.106666667
b4galt1l	0.840531561	1.181069959
cdk16	0.840517241	0.795031056
BTBD2 (1 of many)	0.840490798	1.215053763
gadd45ga	0.840425532	2.325581395
ankrd12	0.840375587	0.76056338
clint1a	0.840354767	1.04359673
hnrnpabb	0.840267983	0.75844806

ncoa4	0.840155945	1.013667426
abcf1	0.840130506	0.898648649
tmem26a	0.84	0.782608696
si:dkeyp-23e4.3	0.84	0.916666667
cnnm2b	0.84	0.966666667
cep89	0.84	1.125
si:ch211-225b11.4	0.84	1.235294118
zgc:112146	0.84	1.314814815
hoxa1a	0.84	1.315789474
nfic	0.84	1.416666667
ackr3a	0.84	1.476190476
lifrb	0.84	1.588235294
tlcd2	0.84	1.816568047
CHCHD5	0.84	0.978947368
map2k1	0.84	1.019607843
tead3a	0.84	1.2
ddrgk1	0.84	1.255813953
gng8	0.84	1.760869565
mcamb	0.839869281	1.341232227
tnmd	0.839762611	1.126829268
crip2	0.839694656	0.813679245
dolpp1	0.839694656	1.11627907
abcd3a	0.839643653	1.040816327
smad3a	0.839622642	1.586206897
sid4	0.839534884	0.783151326
cd82a	0.839524517	1.120754717
ca16b	0.839506173	0.62962963
znf330	0.839506173	1.323353293
rab9a	0.839449541	0.699421965
mpp6a	0.839416058	1.091836735
rock2b_2	0.839416058	1.149425287
asb5a	0.839285714	0.833333333
pi4kaa	0.839285714	0.933333333
calca	0.839285714	0.960784314
mpv17l2	0.839285714	1
wdr19	0.839285714	1.272727273
eif4e1c	0.839130435	1.166281755
alx1	0.83908046	1.381818182
nprl2	0.83908046	2.055555556
anxa1b	0.839007092	1.383617747
prmt3	0.838983051	1.080808081
slc25a43	0.838905775	1.575757576
cdc42ep1b	0.838709677	0.366666667
mafb	0.838709677	0.5
zgc:153115	0.838709677	0.591240876
sox18	0.838709677	0.646153846
elp4	0.838709677	0.696969697
TMEM151A (1 of many)	0.838709677	0.866666667
si:dkey-106c17.3	0.838709677	1
pfkfb2b	0.838709677	1.058823529
gstt2	0.838709677	1.104895105
cryz	0.838709677	1.164556962
cyp2p10	0.838709677	1.303030303
lipea	0.838709677	1.684210526
tacr1b	0.838709677	1.75
tnr	0.838709677	0.358208955
gtf3c5	0.838709677	1.205882353
mpc1	0.838589981	1.29002079
grsf1	0.838582677	1.392156863
ide	0.838574423	1.187692308
cdkn1ba	0.838541667	0.770186335

tsc1a	0.838541667	1.00729927
ptbp1b	0.838440111	1.503968254
CABZ01060453.1	0.838383838	0.8
ppm1k	0.838297872	1.834532374
eml2	0.838235294	0.890034364
si:ch211-237l4.6	0.838235294	0.966666667
col24a1	0.838235294	0.982142857
esyt2b	0.838235294	1.340425532
hadhab	0.838204593	1.053833605
glud1b	0.838190955	0.824674196
MREG	0.838174274	1.18018018
apaf1	0.838095238	0.929577465
fzd9b	0.838028169	0.8046875
prelid1a	0.838016529	1.222648752
si:dkeyp-91h9.1	0.837837838	0.790697674
zgc:162612	0.837837838	0.830769231
atf2	0.837837838	0.9375
si:dkey-54n8.4	0.837837838	0.952380952
si:ch73-21g5.7	0.837837838	1.027027027
si:ch211-121a2.4	0.837837838	1.095238095
si:dkey-247k7.2	0.837837838	1.318181818
NPNT (1 of many)	0.837837838	1.6
psma6l	0.837837838	1.65
hoxb3a	0.837719298	0.691860465
slc25a22	0.837514934	1.533453888
si:ch211-68a17.7	0.8375	1.024561404
poll	0.8375	1.137931034
gbe1b	0.8375	1.453703704
arhgef4	0.837398374	1.091954023
si:zfos-943e10.1	0.837301587	1.231182796
wif1	0.837270341	0.88
eif6	0.837259101	1.2825
si:ch211-239d6.4	0.837209302	0.416666667
tomm20a_2	0.837209302	0.666666667
CTIF	0.837209302	0.821428571
slco1c1	0.837209302	0.885714286
frem1b	0.837209302	0.925925926
heg1	0.837209302	0.945945946
fam210b	0.837209302	1.061538462
epas1b	0.837209302	1.129032258
tmem45b	0.837209302	1.435406699
ttf1	0.837209302	1.575757576
CCKBR (1 of many)_1	0.837209302	1.592592593
sh3gl2	0.837121212	1.715555556
qdpra	0.837104072	1.87804878
cdca7b	0.837078652	0.538961039
esf1	0.837078652	1.052173913
arfip1	0.837078652	1.467741935
si:ch211-254n4.3	0.836956522	1
cmc1	0.836956522	1.208333333
si:ch211-251f6.6	0.836879433	1.16025641
otulina_1	0.836879433	1.475728155
snx9a	0.836734694	1.061946903
cuedc1b	0.836734694	1.019230769
bcl7a	0.836689038	1.168195719
hrsp12	0.836678832	1.219165085
zgc:92664	0.836601307	1.295081967
ccdc80l1	0.836538462	1.724137931
nomo	0.836512262	0.746835443
sox11b	0.836387435	0.900651466
wbp1lb	0.836363636	1.297297297

zgc:174906	0.836363636	1.405660377
htr1aa	0.836363636	0.813559322
kbtbd4	0.836363636	1.055555556
slc17a9b	0.836363636	1.405405405
FYCO1 (1 of many)	0.836363636	1.416666667
ptpn11b	0.836363636	1.603448276
ldah	0.836363636	1.966666667
fabp2	0.836324916	1.427286357
acad11	0.836206897	0.666666667
msrb1a	0.836158192	1.279411765
mllt11	0.836143309	1.289589443
slc5a7a	0.836065574	0.617647059
hic1	0.836065574	0.827956989
zgc:85932	0.836065574	0.834677419
BX005395.1	0.836065574	1.171052632
BX005085.4	0.836065574	1.804347826
nuak1a	0.8359375	1.291262136
gcga	0.835820896	1.0625
PLPP1 (1 of many)	0.835820896	1.244897959
si:ch211-209a2.1	0.835820896	1.540983607
uchl3	0.835648148	1.680147059
lrrc1	0.835616438	0.706349206
sp1	0.835616438	0.738461538
rars	0.835616438	0.982269504
rem2	0.835443038	0.578125
tln2a	0.835365854	0.8
gstr	0.835344828	1.704244032
ccnf	0.835294118	0.893939394
h3f3b.1_1	0.835201649	0.776713048
tyrp1b	0.835098336	0.838560886
vezf1b	0.835051546	0.632352941
hmmr	0.835051546	0.75
si:ch211-11k18.4	0.835	1.005952381
zgc:158463	0.834896121	0.973136261
anos1b	0.834782609	0.936170213
chst1	0.834782609	1
chrnd	0.834782609	1.201005025
surf4l	0.834782609	1.213793103
si:ch211-136a14.2	0.834757835	1.512019231
tmx3	0.834645669	0.736
nsun5_1	0.834645669	0.958333333
ddx41	0.834645669	0.962655602
ntrk2a	0.834586466	0.654320988
nup133	0.834355828	0.705479452
morf4l1	0.834337349	1.133928571
gid8a	0.834196891	0.86
sec31a	0.834170854	0.776315789
poldip3	0.834061135	0.88700565
fam32a	0.833967047	1.565074135
aglb	0.833780161	1.345528455
srsf6b	0.833592535	0.746598639
RNF219	0.833333333	0.56
CABZ01092997.1	0.833333333	0.56
slc34a2b	0.833333333	0.588235294
nts	0.833333333	0.625
zak_1	0.833333333	0.673469388
tspan11	0.833333333	0.694915254
rgs12a	0.833333333	0.75
ar	0.833333333	0.863636364
cdr2a	0.833333333	0.870967742
uts2a	0.833333333	0.916666667

TMEM196 (1 of many)	0.833333333	0.923076923
si:dkeyp-72e1.6	0.833333333	0.936507937
foxa	0.833333333	0.952380952
haus7	0.833333333	1
stxbp5b	0.833333333	1.047619048
epha4l	0.833333333	1.058823529
tmem106a	0.833333333	1.088757396
wdr55	0.833333333	1.15
znf1184	0.833333333	1.153846154
cldn2	0.833333333	1.185714286
dhx57	0.833333333	1.2
fhdc1	0.833333333	1.214285714
pde8b	0.833333333	1.230769231
lsm5	0.833333333	1.253521127
si:ch211-117c19.1	0.833333333	1.263157895
tshz3a	0.833333333	1.267857143
ero1b	0.833333333	1.283333333
ms4a17a.14	0.833333333	1.386363636
klc3	0.833333333	1.5
zgc:100868	0.833333333	1.582463466
si:ch211-66k16.2	0.833333333	1.6875
BX927290.1	0.833333333	1.727272727
si:dkeyp-86f5.1	0.833333333	1.875
mlh3	0.833333333	2
si:ch211-66e2.3	0.833333333	0.48
c1qtnf2	0.833333333	0.5
kiz	0.833333333	0.648648649
melk	0.833333333	0.739130435
PRR7	0.833333333	0.75
erap2	0.833333333	0.846153846
dsg2l	0.833333333	0.869565217
CACUL1	0.833333333	1
mus81	0.833333333	1
slc12a4	0.833333333	1.021276596
si:ch1073-70f20.1	0.833333333	1.041666667
si:dkeyp-121d4.3	0.833333333	1.06557377
zgc:100829	0.833333333	1.083916084
si:dkey-261e22.4	0.833333333	1.111111111
crb2a	0.833333333	1.125
si:ch211-234p6.5	0.833333333	1.130434783
adamts3	0.833333333	1.166666667
gatsl3_1	0.833333333	1.208333333
spc25	0.833333333	1.220779221
si:dkeyp-41f9.4	0.833333333	1.235955056
p3h2	0.833333333	1.269230769
bcl6b	0.833333333	1.314285714
satb1a	0.833333333	1.358974359
si:zfos-979f1.2	0.833333333	1.384615385
dimt1l	0.833333333	1.730337079
cremb	0.833333333	1.769230769
cilp2	0.833333333	1.775
pkp3a	0.833024119	1.212121212
hspa9	0.832925835	1.438026474
etv4	0.832684825	0.761565836
mrpl57	0.832684825	1.089430894
12-Sep	0.832618026	1.024242424
eif4g1a	0.832565284	0.692164179
tssc1	0.832535885	0.777227723
fli1a	0.832535885	1.01910828
gnl2	0.83248731	1.304964539
crybb1l1	0.832339791	0.890130354

murcb	0.832320778	0.982826948
ctage5	0.832258065	0.930232558
hoxc3a	0.832167832	1.505263158
limd2	0.832142857	1.328358209
psme3	0.832089552	1.366515837
zgc:65895	0.832	0.540084388
si:ch211-266i6.3	0.831932773	1.472972973
ccdc177	0.831858407	0.984615385
eri1	0.831858407	1.008474576
cox6b1	0.83178726	0.930017351
palmdb	0.831775701	1.375
smad6b	0.831683168	1.042328042
mipa	0.831594635	1.220338983
klhl18	0.831578947	0.649122807
mak16	0.831541219	1.670103093
prickle1b	0.831521739	0.972413793
sox11a	0.831497797	1.222222222
ankzf1	0.831460674	1.071428571
si:zfos-2326c3.2	0.831460674	1.322580645
usp1	0.831395349	1.007575758
arsh	0.831325301	0.787878788
acacb	0.831325301	1.583333333
pdcd2l	0.831168831	1.222222222
eevs	0.831168831	1.496103896
wdr3	0.831125828	1.007905138
fam213b	0.831034483	1.78238342
si:ch1073-83n3.2	0.830985915	0.22
flj13639	0.830985915	0.757142857
knstrn	0.830985915	0.852941176
zbtb41	0.830985915	1
slc38a3b	0.830882353	1.442105263
nhsl1b	0.830769231	0.523809524
glipr2l	0.830769231	0.8
rab1ab	0.830754352	1.081753555
rras	0.830687831	1.159509202
gabra5	0.830645161	0.652631579
nedd4a	0.830645161	0.956043956
tfr1a	0.830645161	1.028571429
fam96a	0.830645161	2.036585366
itsn2b	0.830601093	1.483050847
sema6d	0.830508475	0.720930233
nog1	0.830508475	0.777777778
sftpbb	0.830508475	1.705882353
fam3c	0.830434783	1.605405405
fam13a	0.830434783	1.646616541
rtca	0.830409357	0.703703704
snx4	0.830388693	1.282485876
gne	0.830357143	0.765432099
ednrba	0.830357143	1.480519481
zgc:66156	0.83030303	0.885964912
abhd12	0.83030303	1.988372093
nme2a	0.830275229	0.820754717
si:dkey-21e2.15	0.830275229	0.905149051
zgc:171775	0.830275229	1.335078534
gbx1	0.830188679	0.41025641
sema5ba	0.830188679	0.428571429
irx3b	0.830188679	0.486486486
usp21	0.830188679	0.877777778
runx3	0.830188679	1.108108108
adpgk2	0.830188679	1.145833333
morc3a	0.830188679	2.064516129

arf6a	0.830128205	0.889705882
sepn1	0.830065359	1.065217391
setd7	0.830065359	1.652892562
txlnbb	0.830053667	1.048442907
prlra	0.83	1.619047619
SEC14L1 (1 of many)	0.829896907	2.115702479
ctcf	0.829824561	0.601366743
slc44a1b	0.829787234	0.833333333
spire1a	0.829787234	0.887323944
mtrf1l	0.829787234	1.075471698
isg20	0.829787234	1.347107438
ecm1b	0.829787234	1.362318841
si:dkeyp-69e1.8	0.829787234	1.847457627
prickle2b	0.82962963	0.581196581
znf395	0.82962963	1.776470588
faub	0.829568789	1.083682008
srp9	0.829545455	1.74617737
kctd1	0.829545455	1.01369863
col4a3bpa	0.829545455	1.14084507
si:ch73-359m17.9	0.829545455	1.271186441
kbtbd11	0.829457364	1.028571429
c1galt1c1	0.829457364	1.269565217
pcdh7b	0.829411765	0.798076923
klf15	0.829383886	1.38961039
zgc:165461	0.829292929	1.059683313
clocka_1	0.829268293	0.6
bub1bb	0.829268293	0.835820896
tk1	0.829268293	1.152542373
ism2a	0.829268293	1.5
clic1	0.829268293	1.298136646
msi2b	0.829145729	1.007042254
phf5a	0.829113924	0.855614973
micu3b	0.829090909	1.962686567
gad1b	0.829059829	0.83797054
si:dkey-22i16.3	0.829039813	0.800813008
yeats4	0.829032258	1.088888889
spam1	0.828947368	0.935064935
uacaa	0.828947368	1.5
mpp6b	0.828947368	0.759776536
ddx55	0.828828829	1.310344828
eif2d	0.828767123	0.891891892
nsfb	0.828767123	1.302083333
spint1b	0.828703704	1.354166667
si:ch73-28h20.1	0.828685259	0.284916201
ralab	0.828571429	1.620155039
dhcr24	0.828571429	0.607594937
hdac6	0.828571429	0.711864407
fance	0.828571429	1
adam8a	0.828571429	1.066666667
si:ch211-248k15.3p	0.828571429	1.15625
prelid1b	0.828571429	1.260869565
zgc:113102	0.828571429	1.28125
CR388042.3	0.828571429	1.366666667
ghdc	0.828571429	1.483870968
gsap	0.828571429	1.666666667
insl5a	0.828571429	1.696969697
si:rp71-1d10.8	0.828571429	1.714285714
si:dkey-16i5.8	0.828571429	2.647058824
sdhdb	0.828460039	1.310223267
ankrd11	0.828282828	0.753521127
arih1l	0.828282828	1.393442623

stmn1b	0.828170925	1.350788288
afp4_2	0.828150573	1.184816214
slc24a3	0.828125	0.772727273
asap3	0.828125	1.037735849
mkln1	0.827956989	1.133333333
mmab	0.827868852	1.433628319
oat	0.827868852	2.083969466
mcm6	0.827751196	0.734138973
ostc	0.827726809	1.366127024
fmoda	0.827715356	1.183615819
sgsm1b	0.827586207	0.428571429
pold2	0.827586207	0.71
homeza	0.827586207	1.459016393
sc:d217	0.827586207	2.705882353
si:ch211-112f3.4	0.827586207	0.791666667
2-Mar	0.827586207	0.806451613
nfxl1	0.827586207	0.836734694
sned1	0.827586207	0.884615385
plcl1	0.827586207	0.921052632
si:dkey-202e22.2	0.827586207	0.9375
rhof	0.827586207	1.09375
RASSF5	0.827586207	1.171428571
prcp	0.827586207	1.196078431
ADGRG2 (1 of many)_1	0.827586207	1.466666667
sirt2	0.8275	1.594095941
ppp2cb	0.827427822	1.126760563
sord	0.827411168	0.941176471
dcun1d1	0.827411168	1.961538462
prph	0.827338129	0.672566372
apoda.1	0.827338129	0.858293076
lace1a	0.827338129	1.648648649
tssc4	0.827205882	1.406542056
numb	0.827160494	0.78
mss51	0.827160494	0.839506173
pcolce2b	0.827160494	0.958677686
znf143b	0.827160494	1.148148148
cep85	0.827160494	1.290322581
rbmx	0.827137546	1.058823529
igf2bp1	0.827067669	0.86130137
tdo2a	0.827027027	1.738095238
si:zfos-169g10.3	0.826923077	0.72
fam207a_2	0.826923077	1.478571429
EVI5L	0.826923077	0.551724138
zgc:136472	0.826923077	1.703125
fam174b	0.826923077	2.102564103
slc22a6l	0.826771654	0.683908046
si:ch211-242e8.1	0.826666667	0.866666667
cnksr3	0.826666667	1.166666667
si:dkey-157g16.6	0.826530612	0.902777778
krit1	0.826530612	0.9625
CABZ01052291.1	0.826530612	1.0625
nudt3a	0.826530612	1.138686131
tuba2	0.826506024	1.126489459
rab8b	0.826446281	1.121212121
dnajb6b	0.826446281	1.555555556
scn1ba	0.82629108	2.203703704
scamp5b	0.826086957	0.578125
vwc2	0.826086957	0.631578947
arhgap6	0.826086957	0.638888889
zgc:110716	0.826086957	0.714285714
fli1b	0.826086957	0.744186047

hephl1a	0.826086957	0.894736842
si:dkey-20i10.7	0.826086957	0.930232558
mstnb	0.826086957	0.954545455
msx2a	0.826086957	1
myo6b	0.826086957	1.018181818
si:ch211-51h9.7	0.826086957	1.046153846
xrcc4	0.826086957	1.047619048
si:ch211-215a9.36p	0.826086957	1.058823529
znf1003	0.826086957	1.0625
tnfaip2b	0.826086957	1.066666667
hace1	0.826086957	1.233333333
mitfa	0.826086957	1.285714286
tmem220	0.826086957	1.326923077
c1qtnf1	0.826086957	1.363636364
sergef	0.826086957	1.375
ttf2	0.826086957	1.5
nudt2	0.826086957	1.524752475
znf983	0.826086957	2.769230769
abcg2b	0.826086957	3.1
micu2	0.825892857	1.758823529
si:ch211-113d11.6	0.825688073	2.4625
pax6a	0.825657895	1.067708333
srgn	0.825581395	0.928205128
itga5	0.825581395	0.985294118
chpfa	0.825581395	1.72
si:dkey-54h16.1	0.825581395	1.725806452
eif4a1a	0.825445605	0.854593523
ppp1r14c	0.825423729	0.977917981
pus7	0.825396825	0.920353982
ippk	0.825396825	1.161073826
fam219b	0.825396825	1.351351351
bambia	0.8252149	0.810344828
gjd1a	0.825174825	0.588541667
dhcr7	0.825136612	0.65
si:ch73-112n4.4	0.825136612	1.08490566
si:dkey-33c14.3	0.825105433	2.380655738
CU856539.3	0.825095057	0.93877551
skila	0.825	0.855072464
desmb	0.825	1.25
cacna1fb	0.825	0.666666667
synpo2lb	0.825	0.8125
klf7a	0.825	0.869565217
nadsyn1	0.825	1.075
depdc7	0.825	1.2
si:ch73-256g18.2	0.825	1.296511628
rfng	0.825	1.315789474
cyp2v1	0.825	1.467948718
tspan18a	0.82464455	1.157232704
sypl2a	0.82464455	1.22906793
pik3r1	0.824561404	1.186736475
gpr158a	0.824561404	0.53125
specc1	0.824561404	0.848484848
pdgfaa	0.824561404	0.86
steap4	0.824561404	1.382022472
hmx2	0.824561404	1.536585366
atp1b4	0.82454624	1.081447964
nfil3-5	0.824489796	0.729885057
PTS	0.824427481	1.056451613
insig2	0.824427481	1.733333333
si:ch73-86n18.1	0.824390244	1.258241758
rab42a	0.824324324	0.852272727

dnmt3ba	0.824324324	1.277777778
prickle1a	0.824324324	0.816326531
man2c1	0.824324324	1.553191489
dnajc27	0.824175824	1.254237288
si:ch73-281n10.2	0.823940487	1.312098188
c1galt1b	0.823863636	1.130434783
matr3l1.1_1	0.823863636	1.243478261
pcsk1nl	0.823684211	0.739495798
chpt1	0.823604061	1.111587983
ano1	0.823529412	0.571428571
si:dkey-25o16.2	0.823529412	0.95
dnajc5gb	0.823529412	1.144444444
tmeff1a	0.823529412	1.185185185
zgc:101715	0.823529412	1.4
si:ch211-232i5.1	0.823529412	1.405405405
tmx2a	0.823529412	1.405405405
si:ch211-87h14.3	0.823529412	1.913580247
rab3db	0.823529412	0.666666667
stat4	0.823529412	0.72972973
arhgef10	0.823529412	0.775862069
PAMR1	0.823529412	0.777777778
PAMR1	0.823529412	0.777777778
NPC2 (1 of many)	0.823529412	0.825991189
atp6v0a2b	0.823529412	0.859375
lgals9l1	0.823529412	0.939393939
adcy7	0.823529412	0.961538462
zgc:171459	0.823529412	1
si:dkey-166k9.2	0.823529412	1.021276596
kif20ba	0.823529412	1.1875
slc16a12b	0.823529412	1.248603352
lypd6b	0.823529412	1.266666667
setd4	0.823529412	1.321428571
hpse2	0.823529412	1.357142857
fancg	0.823529412	1.388888889
casp8	0.823529412	2.105263158
rwdd1	0.823420074	1.57002457
popdc2	0.823129252	1.371428571
vsnl1b	0.823076923	0.943714822
mrpl27	0.822894168	1.03164557
apln	0.822822823	0.976635514
prmt5	0.82278481	0.828125
zgc:56576	0.82278481	1.282178218
slc5a1	0.82278481	0.706896552
spryd4	0.82278481	1.217241379
cul3b	0.822727273	1.034246575
lta4h	0.822719449	1.086538462
kpna3	0.822714681	1.088709677
farp2	0.822695035	0.836206897
dock11	0.822695035	1.102564103
si:dkey-94l16.4	0.822580645	0.785714286
nrarpb	0.822580645	1.710526316
cbx8a	0.822580645	1.857142857
cnbpa	0.822550832	1.088130775
asmtl	0.822510823	1.37628866
tpm4a	0.8225058	1.153225806
acsl4a	0.8225	0.922558923
zgc:66160	0.822485207	1.28440367
gnaq	0.822429907	1.244444444
col6a2	0.82226762	1
si:ch211-112l17.3	0.822222222	0.673076923
roraa	0.822222222	0.83908046

nppc	0.822222222	1.023255814
tmem136a	0.822222222	1.068965517
mars2	0.822222222	1.243243243
rprd1a	0.822222222	1.514492754
zgc:111983	0.822222222	1.627868852
si:ch211-98n17.5	0.822222222	2.700729927
leng1	0.822033898	1.213333333
elp6	0.821917808	1.428571429
si:ch1073-184j22.1	0.821917808	1.547619048
il13ra1	0.821917808	1.661016949
pmp22a	0.821790541	1.22697056
dnaja3b	0.821782178	1.363636364
naa50	0.821678322	0.975778547
shmt1	0.821621622	1.06300813
fgfbp2b	0.821621622	0.619402985
postnb	0.821548822	1.107850514
xk	0.821428571	0.554054054
nanp	0.821428571	0.842105263
si:dkeyp-117h8.4	0.821428571	1.166666667
cpb2	0.821428571	1.181818182
arhgap20	0.821428571	1.307692308
serpina7	0.821428571	1.884615385
prkchb	0.821428571	2.235294118
tekt1	0.821428571	2.285714286
dhfr	0.82122905	1.265151515
aqp9b	0.821167883	0.830508475
opn1mw2	0.821052632	0.556451613
plekhg5a	0.821052632	0.782608696
osbpl7	0.821011673	0.917948718
pelo	0.821011673	1.094170404
prkcbp1l	0.820987654	0.6953125
tmem163b	0.820895522	0.947368421
krcp	0.820895522	0.971962617
nrbp2b	0.820895522	1.225806452
zgc:174928_2	0.820895522	1.274509804
rab11fip1a	0.820895522	1.333333333
notum2	0.820895522	1.692307692
amt	0.820833333	1.040909091
foxp4	0.820809249	0.734848485
sox1b	0.820754717	0.438202247
dennd2da	0.820754717	1.06122449
slitrk4	0.820754717	1.065789474
cyba	0.820754717	1.350515464
prim1	0.820652174	0.793103448
CABZ01055500.1	0.820627803	1.88028169
nmt1b	0.820512821	0.733333333
ADAMTSL5	0.820512821	0.870967742
abhd6a	0.820512821	1
rbck1	0.820512821	1.5
ppox	0.820512821	1.565217391
tbc1d2b	0.820512821	1.655172414
terf2ip	0.820512821	1.875
tdo2b	0.820512821	4.382352941
zgc:101800	0.820512821	1.428571429
tp53	0.820422535	0.723021583
stac3	0.820399113	1.018779343
gucy1b3	0.8203125	1.534246575
si:ch211-241a17.6	0.820276498	0.571428571
acot8	0.820224719	1.445945946
ctdspl2b	0.820175439	1.131428571
safb	0.820075758	0.701970443

rasgef1bb	0.82	1.366906475
tnk2b	0.82	0.5
CABZ01112877.1	0.82	0.703703704
krt93	0.82	1.06122449
si:ch211-274f20.2	0.82	1.159090909
zdhhc2	0.82	1.239130435
zfat	0.82	1.588235294
bnc2	0.81981982	0.986486486
nvl	0.81981982	0.992307692
ttr	0.819787986	1.102827763
LRIG3	0.819672131	0.836734694
camk1gb	0.819672131	0.868421053
polr1d	0.819672131	1.30994152
YIPF7	0.819672131	1.338028169
ca6	0.819557625	0.891708968
rab30	0.819512195	1.923809524
RNF122	0.819444444	1.317460317
hmp19	0.819314642	0.691056911
slc25a23a	0.819277108	0.9
ela2l	0.819229503	1.233777396
cnbpb	0.819228843	1.200841852
si:dkeyp-55f12.3	0.819047619	1.194174757
tspan13b	0.819047619	1.31292517
tmem55ba	0.818965517	0.935483871
sumo3b	0.818941504	1.136861314
ehhadh	0.818897638	0.581818182
inpp5b	0.818791946	0.992063492
gpx7	0.81871345	1.024390244
col12a1b	0.81871345	1.156097561
ddx10	0.818681319	1.222222222
kras	0.818594104	1.354581673
nop56	0.818540434	1.160822249
cnp	0.818505338	0.746361746
mfge8a	0.818428184	0.981751825
rrp12	0.818428184	1.031545741
zc4h2	0.818264014	1.415829146
si:dkey-28e7.3	0.818181818	0.593023256
trpc4a	0.818181818	0.620689655
PEA15	0.818181818	0.864
ftsj3	0.818181818	0.888888889
lzts2a	0.818181818	0.896551724
sept7a	0.818181818	0.92293578
phf23b	0.818181818	1.042105263
mettl13	0.818181818	1.125
lman1	0.818181818	1.178010471
tnnc1a	0.818181818	1.290909091
zgc:73340	0.818181818	1.377358491
mis18a	0.818181818	1.47826087
cpo	0.818181818	1.53968254
cyb5r2	0.818181818	1.635869565
rab38b	0.818181818	1.733333333
prkcq	0.818181818	1.806451613
casp6	0.818181818	1.933333333
si:dkey-102m7.3	0.818181818	2.088888889
etv1	0.818181818	0.371428571
cfhl1	0.818181818	0.714285714
wee1	0.818181818	0.75
diras1b	0.818181818	0.8
kif26ab	0.818181818	0.8
crygm2e	0.818181818	0.816326531
greb1l	0.818181818	0.857142857

galnt18a	0.818181818	0.904761905
s100a10a	0.818181818	0.955484897
rps6ka1	0.818181818	1.064516129
plekhg2	0.818181818	1.074074074
st3gal4	0.818181818	1.085106383
DAB2 (1 of many)	0.818181818	1.112781955
klhl42	0.818181818	1.538461538
MFSD3	0.818181818	1.636363636
myh7bb	0.817869416	1.448863636
srpx	0.817679558	1.020833333
agpat9l	0.817647059	1.359649123
arf1	0.817610063	1.563139932
CRAT (1 of many)	0.817610063	1.619047619
vwa1	0.817589577	0.941605839
tacc3	0.817567568	0.819047619
zgc:56106	0.817460317	1.117647059
zgc:101577	0.817391304	1.069892473
apodb	0.817378498	1.465116279
bgnb	0.817307692	0.888888889
LIMD1	0.817307692	1.08988764
rab11al	0.817307692	1.526315789
sall1a	0.817204301	0.410714286
dennd4a	0.817204301	0.786885246
pgrmc1	0.817010309	1.502114165
ebf2	0.816901408	0.614035088
si:ch1073-186i23.1	0.816901408	0.638297872
gdpd3b	0.816901408	0.974358974
galnt6	0.816901408	1.237113402
si:ch211-137i24.10	0.816720257	0.856687898
cacna2d3	0.816666667	0.421052632
si:dkey-258f14.3	0.816666667	1.52173913
KCNJ11 (1 of many)	0.816568047	0.857142857
zgc:92630	0.816546763	1.971014493
glsa	0.816455696	0.851851852
chchd4a	0.816425121	0.913043478
hebp2	0.816393443	2.509025271
DSP (1 of many)	0.816326531	1.104651163
si:dkeyp-47f9.4	0.816326531	0.8
mtfr2	0.816326531	0.909090909
rnpep	0.816326531	1.264150943
olfml3b	0.816216216	1.005813953
pmelb	0.816091954	0.582417582
si:ch73-332k15.1	0.816091954	0.651162791
larp1b	0.816091954	0.868632708
tmem86a	0.816	1.088607595
txlng	0.816	1.253012048
phka2	0.815920398	1.06779661
tspan5b	0.815899582	1.306569343
fhod3b	0.815789474	0.75
cry1bb	0.815789474	0.945454545
ptpdc1b	0.815789474	0.966101695
cenpn	0.815789474	1.131578947
rpgrb	0.815789474	1.388888889
smc2	0.815789474	0.773333333
gstz1	0.815718157	0.798165138
selm	0.81570997	1.285198556
gtf2a2	0.815699659	1.333333333
map6a	0.815602837	0.734693878
mrpl19	0.81557377	1.2325
nhp2	0.815533981	1.12195122
timp2b	0.815533981	1.2125

lcp1	0.815384615	0.639175258
nat10	0.815384615	1.117647059
vcpkmt	0.815384615	1.259259259
nlgn4b	0.815384615	1.304347826
pcbp3	0.81512605	0.430379747
dock8	0.81512605	0.666666667
epdl2	0.81495564	1.017751479
paqr4b	0.814814815	0.261904762
blm	0.814814815	0.545454545
znf341	0.814814815	0.647058824
si:dkeyp-72e1.9	0.814814815	0.8125
dpy19l1l	0.814814815	0.922077922
si:dkey-73n10.1	0.814814815	1.02
clcn5b	0.814814815	1.047619048
fto	0.814814815	1.166666667
si:dkey-16l2.17	0.814814815	1.409090909
fam83c	0.814814815	1.857142857
bag3	0.814814815	0.795031056
GRAMD2	0.814814815	1.102564103
cmtm3	0.814814815	1.222222222
si:dkey-208k22.3	0.814814815	1.75
etf1b	0.814723926	0.840112202
eif4e2rs1	0.814671815	0.940366972
synj2bp	0.814634146	0.917159763
nrip2	0.814569536	0.790909091
ivns1abpa	0.814492754	0.748979592
cttn	0.814371257	0.857740586
PPM1K (1 of many)	0.814345992	1.525714286
tubb6	0.814285714	1.169811321
arnt	0.814285714	1.25
lipeb	0.814285714	1.307692308
fam198a	0.814285714	1.320754717
mxra7	0.814285714	1.936170213
si:ch211-184m19.3	0.814178303	1.435308343
fxr2	0.814173228	0.970123023
fezf1	0.813953488	0.666666667
CABZ01057488.1	0.813953488	0.734693878
gnrh3	0.813953488	0.964285714
si:ch211-173b16.4	0.813953488	1.035714286
ppfibp2b	0.813953488	1.267605634
slc35b4	0.813953488	1.533632287
tigara	0.813953488	1.84375
swt1	0.813953488	1.923076923
si:ch211-206a7.2	0.813559322	0.27027027
ppp1r1b	0.813432836	0.72972973
mgat1b	0.813333333	1.306122449
rca2.1	0.813304721	1.827683616
gfpt1_1	0.813229572	1.217391304
f2	0.813199105	0.902439024
pcdh1g33	0.813186813	1.122807018
kank2	0.813084112	1.022988506
ptges	0.813084112	1.368421053
cald1l2	0.813031161	1.021276596
frmd4ba	0.81300813	1.125
cyp3c1	0.812967581	1.799342105
rhoab	0.812817904	1.060714286
msi2a	0.812749004	1.151685393
lzts3b	0.8125	0.390243902
topbp1	0.8125	0.666666667
CABZ01048053.1	0.8125	0.676470588
cspp1a	0.8125	0.714285714

MYADM (1 of many)_2	0.8125	0.784313725
btbd11b	0.8125	0.789473684
shda	0.8125	0.814814815
ccdc79	0.8125	0.823529412
si:ch211-129o13.2	0.8125	0.88
taok3a	0.8125	0.904761905
ccser1	0.8125	0.909090909
ttyh2	0.8125	0.923076923
nudt13	0.8125	0.941176471
SLC46A2	0.8125	1
inpp5d	0.8125	1.0625
malt2	0.8125	1.071428571
stambpl1	0.8125	1.121212121
pex1	0.8125	1.175
samd10b	0.8125	1.176470588
ypel5	0.8125	1.2
gpd2	0.8125	1.205128205
SGMS2 (1 of many)	0.8125	1.217391304
apobec2b	0.8125	1.3
cdc42bpaa	0.8125	1.36
mef2ab	0.8125	1.4
sparcl1	0.8125	1.41
si:ch211-15d5.11	0.8125	1.447761194
pcsk5a	0.8125	1.45
adrb3a	0.8125	1.466666667
si:dkey-219e21.4	0.8125	1.5
p2rx3b	0.8125	1.625
glipr2	0.8125	1.692307692
znf1046	0.8125	1.916666667
WNT7A (1 of many)	0.8125	2.625
pomt1	0.8125	1.671052632
emc8	0.8125	1.935135135
vkorc1	0.8125	2.112676056
si:ch211-120j21.1	0.812030075	1.008474576
tmem242	0.811965812	1.311320755
col18a1	0.811936937	0.878246753
chchd10	0.811891892	1.099787686
trak1	0.811881188	0.909090909
hic2	0.811827957	1.195121951
chadlb	0.811764706	0.637931034
tax1bp1a	0.811764706	1.458333333
zgc:195001	0.811746988	2.082228117
plekho1b	0.811688312	0.714285714
sox13	0.811688312	1.064516129
kcnj2a	0.811594203	1.384615385
scamp2l	0.811594203	1.893617021
porb	0.811538462	1.113513514
mbd6	0.811320755	0.769230769
CU682355.1	0.811320755	1.054054054
si:ch211-197g15.9	0.811320755	1.111111111
dok1b	0.811320755	1.176470588
si:ch73-27e22.4	0.811320755	2.305555556
tyms	0.811188811	0.797909408
prkaa2	0.811111111	1.2
lyar	0.811059908	0.75
col6a1	0.810895429	1.212268744
pitpnab	0.810810811	1.614084507
kif26aa	0.810810811	0.72
nim1k	0.810810811	0.833333333
bcl2a	0.810810811	1.12244898
gtf3c2	0.810810811	1.148148148

rassf2a	0.810810811	1.313131313
ifit16	0.810810811	1.518518519
zdhhc20a	0.810810811	2.294117647
esrp2	0.810526316	0.926666667
sema3e	0.810483871	1.224852071
gbgt1l4	0.810457516	1.646464646
ompa	0.810344828	0.788359788
ARHGAP28	0.810344828	1.205128205
negaly6	0.810344828	1.319148936
sh2d4a	0.810344828	1.404761905
fgf24	0.810344828	1.409090909
slc25a29	0.810344828	1.457142857
si:dkey-56m19.5	0.810218978	0.778801843
apof	0.810218978	0.902834008
rab24	0.810218978	1.870967742
cel.1	0.810169492	0.913963329
sncb	0.810032017	0.935799782
arhgap17b	0.809954751	1.117283951
si:ch211-235e9.8	0.809904153	1.357476636
flncb	0.809859155	0.943089431
letm1	0.80952381	0.804597701
ecm2	0.80952381	0.929824561
rgs12b	0.80952381	0.987012987
derl2	0.80952381	1.177272727
ap1s3a	0.80952381	1.548780488
esrrb	0.80952381	0.526315789
si:ch211-250e5.2	0.80952381	0.535714286
klhl5	0.80952381	0.633333333
dlg2	0.80952381	0.666666667
mboat2a	0.80952381	0.814814815
zbtb7a	0.80952381	0.866666667
impg1a	0.80952381	0.903846154
znf532	0.80952381	0.95
atg10	0.80952381	1
VGLL3	0.80952381	1.071428571
adamtsl3	0.80952381	1.153846154
znf1039	0.80952381	1.166666667
si:ch1073-209e23.1	0.80952381	1.290322581
hinfp	0.80952381	1.304347826
zgc:158654	0.80952381	1.342105263
si:ch73-257c13.2	0.80952381	1.413043478
zgc:153311	0.80952381	1.5
nsun3	0.80952381	1.777777778
si:dkey-111k8.2	0.80952381	2
TFR2	0.80952381	2
si:dkey-109l4.3	0.80952381	2
hgfa	0.80952381	2.4
SFT2D2 (1 of many)_2	0.80952381	3.291666667
tcn2	0.809430255	0.807277628
zgc:66479	0.809358752	1.396984925
lect2l	0.809352518	0.822222222
rrp9	0.809302326	1.054455446
B4GALNT2 (1 of many)	0.809210526	1.5
zgc:77748	0.809210526	1.038343558
ptrh2	0.809160305	1.097560976
agxtb	0.809141445	1.931948424
rhoua	0.809090909	1.048780488
tnk1	0.809090909	1.474358974
cfhl2	0.809045226	0.9296875
mrps31	0.808988764	1.503246753
si:ch73-167c12.2	0.808988764	2.270833333

abcg2a	0.808955224	1.018726592
tsen34	0.808917197	1.2
exosc6	0.808823529	0.772222222
pcdh8	0.808823529	1.470588235
ablim2	0.808641975	1.033333333
slc43a1a	0.80861244	0.92
dusp23a	0.808510638	0.959641256
espl1	0.808510638	0.8125
snu13a	0.808510638	0.875
nrgna	0.808510638	0.910204082
papd5	0.808510638	0.957055215
gsg1l	0.808510638	0.978723404
fam19a1a	0.808510638	1.095238095
abcb10	0.808510638	1.215384615
lipt1	0.808510638	1.239130435
si:ch211-158d24.4	0.808510638	1.675
lpl	0.808357349	1.428
coro2ba	0.808333333	0.875
tagln2	0.808290155	1.138912856
si:ch211-93f2.1_1	0.808259587	0.93038822
tmtc2a	0.808219178	0.66
CR855389.1	0.808219178	1.078947368
smim12	0.808219178	1.440528634
adkb	0.808199122	1.191119691
rpl7l1	0.808184143	1.314465409
lrsam1	0.808080808	1.219178082
zgc:153240	0.808035714	1.165467626
pdxdc1	0.808	0.802083333
srebf2	0.807971014	0.706422018
fxyd6	0.807843137	0.886699507
fxyd1	0.807831762	1.196460177
trmt1l	0.807692308	0.652173913
si:dkey-188i13.6	0.807692308	0.727272727
phyhiplb	0.807692308	0.739130435
mmd2a	0.807692308	0.85
rdh14a	0.807692308	0.886075949
tpcn2	0.807692308	1
rasgrp3	0.807692308	1.125
FO704846.1	0.807692308	1.391304348
adamts15b_1	0.807692308	1.491666667
si:dkey-157l19.2	0.80733945	0.917431193
scp2b	0.807302231	1.560471976
bcar3	0.807228916	0.6125
cited1	0.807228916	1.08
ndnl2	0.807228916	1.246376812
sltm	0.807174888	1.023255814
phf20l1	0.807142857	0.89010989
c1qtnf5	0.807142857	1.32
adra2b	0.807017544	0.833333333
ascc1	0.807017544	1.093333333
snrpa1	0.806962025	0.937293729
CYTH2	0.806930693	1.353535354
slc25a24	0.806896552	1.272727273
rgmd	0.806866953	1.005319149
vmo1b	0.806818182	0.77294686
pycard	0.806590258	1.403985507
FO704819.2	0.806451613	0.505882353
samd1a	0.806451613	0.682242991
abrab	0.806451613	0.852459016
larsb	0.806451613	0.935114504
gmeb1	0.806451613	0.955223881

svila	0.806451613	1.080645161
trmt6	0.806451613	1.337349398
CU633479.2	0.806451613	1.365714286
zgc:103755	0.806451613	1.409090909
fgf8b	0.806451613	1.421052632
si:ch211-127b11.1	0.806451613	1.833333333
brms1	0.806451613	2.060606061
st14b	0.80620155	1.144578313
rtn2b	0.806146572	1.208609272
nhsl2	0.806122449	0.854545455
ap4s1	0.806122449	1.23853211
caspb	0.806054872	1.693589744
txnbb	0.805970149	0.606299213
dscc1	0.805970149	1.050847458
h3f3b.1_3	0.805905131	0.803073421
fbl	0.805855162	0.964776632
agt	0.805769231	1.34375
lmna	0.805714286	1.387931034
tsg101a	0.805668016	1.530434783
si:ch211-188p14.5	0.805555556	0.708333333
col28a1a	0.805555556	0.782608696
p2rx1	0.805555556	1.4
si:dkey-65b12.6	0.805555556	1.425373134
lgalsla	0.805555556	1.434782609
cmc4	0.805194805	1.491525424
top2b	0.805172414	1.210526316
aspdh	0.805031447	0.76097561
lsm6	0.804924242	1.171624714
dvl1b	0.804878049	0.407407407
rnf183_1	0.804878049	1.022727273
papss2b	0.804878049	1.029166667
nphp3	0.804878049	1.2
col27a1b	0.804878049	1.272727273
wu:fb18f06	0.804878049	1.875
nid1a	0.804780876	0.823943662
arhgdig	0.804709141	1.15
si:ch211-84k18.3p	0.8046875	1.065934066
snrnp25	0.8046875	1.65625
si:ch211-190h11.2	0.8046875	2.636363636
fam234a	0.804597701	1.436363636
rac3b	0.804521277	1.366666667
cndp2	0.80449827	1.319838057
myct1a	0.804347826	0.428571429
gab2	0.804347826	0.727272727
ajap1	0.804347826	0.787878788
si:ch211-272n13.3	0.804347826	0.787878788
rhoq	0.804347826	0.873417722
si:dkey-34d22.1	0.804347826	0.984375
dhx38	0.804347826	1.08
kif13bb	0.804347826	1.090909091
frem2a	0.804347826	1.395833333
tac3a	0.804347826	1.406593407
nr2f6b	0.804347826	0.967567568
vash2	0.804347826	0.701030928
zgc:85777	0.804289544	1.241322314
wnt11r	0.804210526	0.997867804
aurkaip1	0.804210526	1.031662269
hoxa10b	0.804123711	0.987804878
slka	0.804054054	1.625
wu:fb59d01	0.803956835	0.701834862
jupa	0.803941909	0.99863388

si:ch73-233f7.8	0.803921569	0.727272727
opn8b	0.803921569	1.413793103
si:ch211-172l8.4	0.803921569	2.088235294
taok2a	0.803738318	0.865546218
CABZ01077217.1	0.803571429	1.195652174
itga11a	0.803571429	1.322580645
scoca	0.80349345	1.466292135
ctgfb	0.803468208	1.083333333
ppp3cca	0.803418803	1.108108108
RABL6 (1 of many)	0.803278689	0.972222222
slc35a3a	0.803278689	1.322580645
pax6b	0.803191489	0.885416667
ptrfa	0.803149606	1.634146341
ywhag2	0.803111909	0.750397456
brox	0.803108808	1
fkbp10a	0.803030303	1.333333333
dlc	0.803030303	1.268656716
gad2	0.80293501	0.666666667
gnb5b	0.80293501	0.818731118
calua	0.802919708	1.034013605
kctd12.2	0.802816901	0.940860215
NPBWR2	0.802816901	1.040540541
nrn1lb	0.802721088	1.568
snx5	0.802675585	1.827225131
parvab	0.802631579	0.864197531
mbnl1	0.802197802	0.818181818
zbtb33	0.802197802	1.218181818
oatx	0.802083333	0.8
tbx15	0.802083333	1.075
ccny	0.802083333	2.042553191
hspd1	0.802063185	0.948540707
h3f3b.1_2	0.801923077	0.727391874
mroh1	0.801886792	1.265625
sult2st2	0.801857585	0.929588608
cacna2d4b	0.801724138	0.830769231
atp11a	0.801724138	1.102941176
fam172a	0.801724138	1.478873239
dek	0.801724138	1.879746835
pah	0.801664355	1.637826962
itih2	0.801652893	1.180100756
hoga1	0.801587302	0.868421053
plxdc2	0.801587302	1.284916201
si:ch211-218c6.8	0.801242236	1.521428571
rnf144b	0.801242236	1.554545455
dab2	0.801204819	0.815068493
eif1axa	0.801104972	1.546184739
phrf1	0.80104712	1.246031746
gorasp1a	0.801020408	1.310344828
nefma	0.801007557	0.411764706
hnrpdl	0.800227015	0.77457405
scai	0.8	0.413793103
sowahcb	0.8	0.431372549
supt7l	0.8	0.617021277
snapc4	0.8	0.657142857
abhd15a	0.8	0.697674419
rhbdl3	0.8	0.707692308
necab2	0.8	0.794612795
hbl4	0.8	0.80952381
ap1ar	0.8	0.846153846
rfx5	0.8	0.846153846
apbb1ip	0.8	0.972222222

stard7	0.8	0.975510204
fbxo40.1	0.8	0.984375
rgn	0.8	0.995901639
mst1ra	0.8	1
rps6ka3b	0.8	1
prph2l	0.8	1.03030303
CABZ01076047.1	0.8	1.083333333
adamts10	0.8	1.088235294
zgc:158403	0.8	1.098360656
gm2a	0.8	1.151376147
CABZ01079984.1	0.8	1.166666667
slc41a1	0.8	1.170731707
ska3	0.8	1.178571429
chka	0.8	1.206896552
pcdh1g18	0.8	1.208333333
abi3bpa	0.8	1.214285714
si:dkey-183k8.2	0.8	1.222222222
panx1b	0.8	1.227272727
cspg4	0.8	1.24
tnip2	0.8	1.266666667
ckap2l	0.8	1.3
stc2b	0.8	1.307692308
dcaf17	0.8	1.4
primpol	0.8	1.428571429
si:ch211-89o9.6	0.8	1.431372549
si:zfos-1056e6.1	0.8	1.583333333
si:dkey-58f10.7_2	0.8	1.588235294
grhl1	0.8	1.594339623
glsl	0.8	1.6
brd1a	0.8	1.603174603
dnai1.2	0.8	1.615384615
gpr37a	0.8	1.85
si:ch211-119e14.9	0.8	2.45
arl10	0.8	2.615384615
gc3	0.8	0.345588235
si:ch211-203d7.1	0.8	0.358490566
cacng7b	0.8	0.571428571
ccdc80	0.8	0.75
rad9a	0.8	0.807692308
pld1b	0.8	0.888888889
znf407	0.8	0.892857143
si:dkey-239i20.2	0.8	0.923076923
tnfrsf9a	0.8	0.923076923
cass4	0.8	1
AL935280.1	0.8	1.034482759
cnot6b	0.8	1.12
pwp2h	0.8	1.185430464
zgc:162898	0.8	1.202380952
sec16b	0.8	1.230769231
chrne	0.8	1.27972028
si:ch73-105b23.6	0.8	1.285714286
dusp12	0.8	1.333333333
tcf3a	0.8	1.339622642
ntpcr	0.8	1.518518519
zgc:113411	0.8	1.529411765
manea	0.8	1.886363636
gls2b	0.8	1.9
grk5	0.8	1.941176471
znf1180	0.8	2.0625
desi1b	0.8	2.953488372
etnk1	0.799450549	1.376666667

prkag1	0.799342105	2.372262774
isoc2	0.799295775	1.117647059
naprt	0.79916318	0.912037037
rrs1	0.799086758	1.364640884
arr3b	0.799013564	1.886737657
gucd1	0.798913043	1.044585987
ino80c	0.798850575	1.281045752
CTBP1	0.798701299	0.914691943
dmrt2a	0.798507463	1.277227723
fndc3bb	0.798449612	0.9375
anapc15	0.798449612	1.195979899
akr7a3	0.798353909	1.034934498
acot13	0.798353909	1.189300412
m6pr	0.798353909	1.890243902
si:dkey-172m14.1	0.798319328	1.178947368
nacad	0.798165138	1.1
fscn2b	0.798076923	0.833333333
RAP1GDS1	0.797979798	0.6
lysmd4	0.79787234	1.983050847
mat1a	0.797867184	1.520819564
actr2a	0.797752809	1.244755245
nufip1	0.797752809	1.4
zmp:0000001167	0.797687861	0.796296296
ccdc47	0.79766537	0.977272727
si:ch211-214p16.2	0.797619048	0.676923077
hoxb9a	0.797619048	1.017857143
exosc9	0.797619048	1.96875
fam89a	0.79757085	1.503355705
rrbp1a	0.797468354	0.643617021
phldb1b	0.797468354	0.698113208
si:ch73-236j9.2	0.797468354	0.767241379
lum	0.79739777	0.955863808
hnmt	0.797297297	1.404494382
acp6	0.797297297	1.551724138
ipo4	0.797202797	1.224489796
abhd17b	0.797101449	1.193548387
dap	0.796992481	0.842176871
si:ch73-308l14.2	0.796875	1.139784946
si:ch73-109d9.1	0.796875	1.181818182
ptk6b	0.796875	1.346938776
plekha8	0.796747967	2.283333333
mpv17	0.796703297	0.93956044
crnkl1	0.796610169	0.663507109
atp1a1a.4	0.796511628	0.707152497
sf3a3	0.796420582	1.366754617
ntd5	0.79638009	1.090697674
esyt1b	0.796296296	0.868421053
PITPNC1	0.796296296	1.430379747
cerk	0.796296296	1.518518519
CABZ01046954.1	0.796296296	3.173913043
dnajc5ab	0.796116505	1.025547445
prmt7	0.796116505	0.6875
wdr12	0.796116505	1.166666667
si:ch211-196i2.1	0.796116505	1.223684211
cetp	0.796052632	0.907801418
cacng6b	0.795918367	1.596491228
gtf3c4	0.795918367	0.770833333
SFT2D2 (1 of many)_1	0.795918367	1.05
acot11a	0.795918367	1.051282051
exoc3l1_1	0.795918367	1.275
xkr9	0.795918367	1.595744681

dgke	0.795918367	1.666666667
ilk	0.795865633	0.82111437
adka	0.795737123	0.84501845
si:ch211-140m22.7	0.795731707	1.015698587
sept4a	0.795698925	0.911764706
smyd5	0.795698925	1.132653061
aadat	0.795698925	1.52
six1b	0.795620438	0.918518519
parvaa	0.795620438	1.51552795
rrm1	0.795532646	0.750957854
aff2	0.795454545	0.535714286
itsn2a	0.795454545	0.818181818
crtc2	0.795454545	0.837837838
tpk1	0.795454545	0.907407407
GABRA2 (1 of many)_1	0.795454545	1
sh3bgrl	0.795454545	1.371134021
ptmaa	0.795442287	1.346052632
si:ch211-71n6.4	0.795180723	1.037735849
mybl2a	0.795180723	1.245283019
sec24d	0.795081967	0.776649746
urah	0.795061728	1.112094395
neurl1aa	0.794871795	0.634615385
dnajc2	0.794871795	0.872
drd4-rs	0.794871795	1.037037037
asb15a	0.794871795	1.239130435
CABZ01033206.1	0.794871795	1.283018868
BX324216.4	0.794871795	1.5
crtc1a	0.794871795	1.5625
calcoco2	0.794871795	1.583333333
tmem119b	0.794871795	0.909547739
CFP (1 of many)	0.794736842	0.7
cyp26b1	0.794642857	1.175
cpt1ab	0.794628752	1.417391304
nsdhl	0.794520548	0.975903614
ahsa1a	0.794520548	0.98974359
map3k14a	0.794520548	1.078431373
cyp2x9	0.794520548	1.466666667
NPR2	0.794392523	0.8125
rnf183_2	0.794285714	1.654545455
efhd2	0.794217687	1.225382932
cap2	0.794212219	0.794117647
cadps2	0.794117647	0.607142857
CABZ01085924.1	0.794117647	0.633333333
si:dkey-199l17.8	0.794117647	0.65
fgfbp1b_1	0.794117647	0.768
si:zfos-1897c11.1	0.794117647	1.513157895
FO904965.1	0.793991416	1.114754098
prph2a	0.793875147	0.460244648
gria3b	0.793814433	0.80952381
LTBP3	0.793814433	0.819148936
phldb1a	0.793814433	1.064102564
FLNB	0.79375	0.816513761
si:ch1073-205c8.5	0.793650794	0.423076923
SRMS	0.793650794	1.113636364
kcna6a	0.793650794	1.271186441
nppa	0.793650794	1.391304348
nkx2.3	0.793478261	0.779220779
meox1	0.793478261	1.025
wwtr1	0.793478261	1.17721519
slc35a2	0.793478261	1.307692308
tpm4b	0.793333333	0.825396825

rwdd	0.793333333	1.924855491
arl3l1	0.793296089	1.341666667
dpydb	0.793201133	0.601226994
kank1a	0.793103448	0.963190184
neurod2	0.793103448	0.970149254
si:dkeyp-8h9.1	0.793103448	1
rad50	0.793103448	1.310344828
rhpn2	0.793103448	1.36
celf5b	0.793103448	0.297297297
appl2	0.793103448	0.714285714
ptger2a	0.793103448	1.173913043
slc25a15b	0.793103448	1.2
znf1067	0.793103448	1.2
SLC9B2	0.792899408	0.652631579
cdk1	0.792899408	1.104377104
vps36	0.792857143	1.316326531
dhx37	0.792792793	1.0625
elovl6	0.792763158	1.170403587
tmem240b	0.79245283	0.608108108
ncapg2	0.79245283	0.795918367
si:dkeyp-44a8.4	0.79245283	1.128205128
pgm5	0.79245283	1.2
cyp3c3	0.79245283	1.28
klhdc4	0.79245283	1.117647059
asns	0.79245283	1.328125
sh3glb1a	0.79245283	1.495495495
plod2	0.792307692	1.030927835
PLIN3	0.792207792	1.102362205
FBLN7	0.792207792	1.125
ube2w	0.792	1.88
foxd2	0.791946309	1.149122807
pard6a	0.791946309	1.252525253
idh1	0.791743119	1.450134771
si:dkey-221l4.13	0.791666667	0.5
satb2	0.791666667	0.566666667
tube1_1	0.791666667	0.708333333
rragd	0.791666667	0.733333333
inaa	0.791666667	0.937142857
alkbh6	0.791666667	0.987179487
cfap77	0.791666667	1
cyb561d1	0.791666667	1.090909091
CPNE8 (1 of many)	0.791666667	1.097826087
CABZ01073329.1	0.791666667	1.151515152
lgi1b	0.791666667	1.164179104
mical2b	0.791666667	1.202702703
luzp1	0.791666667	1.209302326
ptk6a	0.791666667	1.3125
si:dkey-286j17.3	0.791666667	1.434782609
baiap2l2b	0.791666667	1.571428571
ompb	0.791666667	1.703703704
si:dkey-183c16.7	0.791666667	1.75308642
si:ch73-334d15.2	0.791666667	1.941176471
BX957331.1	0.791650099	1.096377307
COX7A2 (1 of many)	0.79144385	1.097959184
ttc1	0.791411043	1.25
si:ch211-262h13.3	0.791304348	0.815217391
vegfaa	0.791208791	1.245901639
chchd3b	0.791208791	1.398734177
smad5	0.791111111	0.683168317
dusp14	0.791044776	0.446428571
oip5-as1	0.790937178	2.607883817

fxyd6l	0.790909091	1.100360268
npat	0.790909091	0.926829268
zgc:110283	0.790909091	1.458333333
yipf1	0.790909091	2.328571429
hnrnpa0a_1	0.790782292	0.436860068
fam162a	0.790780142	1.201271186
hs3st3l	0.790697674	0.7
tns2a	0.790697674	0.984848485
ZNF335	0.790697674	1.083333333
plekhg6	0.790697674	1.363636364
acox3	0.790697674	1.373333333
inpp5l	0.790697674	1.625
ugt5a2	0.790697674	4.235294118
clk4a	0.790633609	1.324324324
polr1a	0.790575916	1.161764706
grk7a	0.790533736	0.60658083
sfxn3	0.79040404	1.513207547
mid1ip1b	0.7904	1.065099458
mrs2	0.790322581	1.526315789
slc25a15a	0.790322581	1.702702703
aldh1a3	0.790123457	1.121212121
becn1	0.79	1.952
srsf6a	0.789915966	0.742996346
pnp4a	0.789893617	0.975524476
agxt2	0.789883268	1.082524272
prkrira	0.789855072	1.068376068
si:dkey-14d8.6	0.789855072	1.297709924
vapb	0.78978979	1.024390244
aqp3a	0.78976234	1.135186057
acbd5b	0.789473684	0.625
thbs2a	0.789473684	0.639344262
adgre5b.2	0.789473684	0.681818182
si:ch211-202h22.9	0.789473684	0.714285714
sacs	0.789473684	0.916666667
CEP192	0.789473684	0.933333333
si:dkey-239n17.4	0.789473684	1.185185185
poc1bl	0.789473684	1.197183099
oit3	0.789473684	1.444444444
ldlrad4b	0.789473684	1.466666667
zdhhc12a	0.789473684	1.56097561
si:dkey-253d23.11	0.789473684	1.961538462
si:dkey-102c8.2_1	0.789473684	2
si:dkey-156m18.1	0.789473684	2.16
kdelr3	0.789473684	0.801724138
zcchc24	0.789215686	1.387096774
skilb	0.789115646	1.289156627
si:ch211-142k18.1	0.7890625	1.196721311
mthfd1b	0.789029536	0.788702929
cox4i2	0.788888889	0.733333333
tbc1d7	0.788888889	1.085365854
purbb	0.788856305	0.910505837
wsb2	0.788732394	1.140350877
si:ch211-134a4.1	0.788732394	2.323076923
c1d	0.788617886	1.482954545
cpox	0.788461538	0.557971014
mlf1	0.788461538	0.985119048
loxl2b	0.788461538	1.052459016
commd7	0.788461538	1.388461538
ncs1a	0.788461538	0.8
dtna	0.788461538	0.952380952
trim2a	0.788461538	1

gal3st1b	0.788461538	1.037735849
slc5a3b	0.788461538	1.3125
rnf181	0.788461538	1.461538462
camk2n2	0.788359788	1.119760479
fis1	0.788359788	1.343283582
tmed6	0.788321168	0.839622642
arhgef16	0.788321168	0.956521739
CU929052.1	0.788235294	0.822580645
hoxb7a	0.788235294	0.835820896
smtnl	0.788235294	0.967320261
slc7a7	0.788235294	1.011049724
birc5a	0.788235294	1.228571429
thbs4a	0.788235294	1.46
itga1	0.788235294	1.511363636
cx23	0.788135593	1.095652174
golga1	0.788135593	1.306666667
hspg2	0.788043478	1.008403361
gucy2c	0.787878788	0.608695652
arhgef1a	0.787878788	0.708333333
aanat2	0.787878788	0.714285714
col5a2b	0.787878788	0.761904762
BX548067.1	0.787878788	0.916666667
zcchc7	0.787878788	0.93877551
ccdc136b	0.787878788	0.9875
sh3bp4	0.787878788	1.7
gabra1	0.787878788	1.112582781
jph1a	0.787878788	1.189781022
gmppab	0.787610619	1.76119403
pora	0.787535411	1.568281938
gpr27	0.7875	0.754385965
NINJ1	0.7875	1.568181818
tfe3b	0.7875	2.01754386
lactbl1a	0.787401575	0.709677419
tmem86b	0.787313433	1.71686747
baiap3	0.787234043	0.545454545
cyp2k8	0.787234043	0.875
TANC1 (1 of many)	0.787234043	0.967741935
dhrs1	0.787234043	1.09380531
rab32a	0.787234043	1.25
si:dkey-13a3.5	0.787234043	1.375
ckmt1	0.787208607	0.692307692
b4galt1	0.787096774	1.545454545
zbtb37	0.787037037	1.218181818
cog6	0.786885246	1.045977011
si:dkeyp-79f4.1	0.786885246	1.261904762
mao	0.78680203	1.126984127
pdzrn3b	0.786764706	1.052083333
osbpl3a	0.786666667	1.236363636
gpx3	0.786516854	1.5
rab22a	0.786407767	2.75
glra1	0.786259542	0.662162162
cmtm6	0.786259542	1.457831325
tmem184a	0.786206897	1.389380531
bcl2l13	0.786144578	1.388127854
AC024175.6	0.786069652	1.235436893
si:dkey-67c22.2	0.78597786	1.14516129
adra1bb	0.785714286	0.611111111
rbp1	0.785714286	0.628571429
gmip	0.785714286	0.666666667
si:ch211-105f12.2	0.785714286	0.684210526
hsd3b1	0.785714286	1

dusp10	0.785714286	1.142857143
CABZ01041002.1	0.785714286	1.333333333
cln8_1	0.785714286	1.363636364
zcchc10	0.785714286	1.369565217
irf8	0.785714286	1.416666667
si:ch211-125e6.5	0.785714286	1.629032258
CU914776.1	0.785714286	1.727272727
aatf	0.785714286	1.773333333
znf1174	0.785714286	1.8
CR361561.2	0.785714286	1.842105263
slc12a10.1	0.785714286	1.916666667
st6gal1	0.785714286	0.948717949
foxn2b	0.785714286	0.970588235
cxxc1b	0.785714286	1
ptrh1	0.785714286	1.135135135
apoob	0.785714286	1.307692308
rbm17	0.785714286	1.558139535
olfml3a	0.785714286	2.24691358
SP6	0.785714286	0.823529412
gpr17	0.785714286	1.034482759
wnt3	0.785714286	1.090909091
cxcr3.3	0.785714286	1.321428571
cyp3c4	0.785714286	2.407407407
kdelr2a	0.785630153	1.209756098
myo1eb	0.785276074	1.099009901
cpa1	0.785276074	1.260869565
si:ch73-138n13.1	0.785185185	0.944954128
fbln2	0.785123967	0.922680412
csf1ra	0.785123967	1.383838384
pip5k1aa	0.785046729	2.018867925
sorbs3	0.784946237	0.985074627
cxxc4	0.784946237	1.616666667
EFEMP1 (1 of many)_2	0.784753363	1.554140127
plekho1a	0.784615385	0.634920635
lbx2	0.784615385	1.416666667
rgs4	0.784461153	1.833333333
si:ch211-106n13.3	0.784313725	1
phf19	0.784313725	1.081081081
nr0b1	0.784313725	1.291666667
rargb	0.784313725	1.34375
dnajb9a	0.784313725	2.235294118
prpf3	0.784172662	1.260869565
lmbrd2b	0.784140969	1.037974684
ric8a	0.784037559	0.813793103
fbxo5	0.783783784	0.479452055
bean1	0.783783784	0.695652174
si:ch211-235p24.2	0.783783784	1.28
si:dkey-57h18.2	0.783783784	1.297297297
dusp23b	0.783783784	1.395744681
arhgef38	0.783783784	1.9375
rhbdd3	0.783783784	2.269230769
clpp	0.783715013	1.024464832
si:ch211-167b20.8	0.783606557	2.627586207
pld2	0.783505155	1.305084746
cldnc	0.783464567	0.834558824
fnbp1l	0.783410138	0.917721519
capgb	0.783365571	0.973076923
hoxb1b	0.783333333	0.807017544
kntc1	0.783333333	0.84
gfra2a	0.783333333	0.977777778
ADAMTS14	0.783333333	1.022222222

ifitm5	0.783333333	1.186813187
CAMK2N1	0.783289817	0.783281734
zgc:153845	0.783185841	1.574193548
si:ch73-335m24.2	0.78313253	0.702702703
dtymk	0.78313253	1
sv2ba	0.782841823	0.787037037
vcam1b	0.782608696	0.656565657
si:dkey-61l1.4	0.782608696	0.846153846
mpp5b	0.782608696	0.885714286
ccnb2	0.782608696	0.964285714
si:ch73-109d9.3	0.782608696	1.066666667
camkk1a	0.782608696	1.128205128
hspa12a	0.782608696	1.155172414
hdac10	0.782608696	1.162790698
spi1b	0.782608696	1.2
si:dkey-246g23.4	0.782608696	1.631578947
trim108_1	0.782608696	2.277777778
si:dkey-165e24.1	0.782608696	2.461538462
wdr46	0.78245614	0.966527197
rsu1	0.78245614	1.264573991
tmem135	0.782442748	1.190954774
si:ch211-256m1.8	0.78238342	1.017647059
nanos1	0.782343988	1.23877551
si:ch211-288g17.4	0.782258065	1.652173913
col4a1	0.782157676	1.012012012
colec12	0.782142857	1.35828877
anxa3a	0.782101167	1.314720812
CYSTM1 (1 of many)_2	0.782051282	0.515789474
galk1	0.782051282	0.85915493
sfrp5	0.782051282	0.943548387
e2f3	0.782051282	1.210526316
fastkd1	0.782051282	1.416666667
xirp2b	0.781954887	1.587628866
sult4a1	0.781954887	1.677290837
fam102ba	0.781954887	2.195121951
gpm6ab	0.781929348	0.771944923
itgb3a	0.781893004	1.588235294
fam49a	0.781879195	0.703389831
atp8b5a	0.781818182	0.772727273
cped1	0.781818182	0.945054945
triqk	0.781818182	1
rnf11b	0.781818182	1.058823529
si:dkey-30d10.1	0.781818182	1.323529412
kdelc2	0.781818182	1.402439024
slc7a3b	0.781818182	1.405405405
sema3d	0.781690141	0.891213389
ddx56	0.781690141	1.262711864
thg1l	0.781609195	1.8125
si:dkey-164f24.2	0.781565657	1.408547009
tppp3	0.781512605	1.231213873
ptrfb	0.781472684	1.047337278
tmpoa	0.781420765	0.808510638
sgk3	0.781376518	1.567567568
tgm2b	0.781333333	0.771356784
opn3	0.78125	0.5
dmbx1b	0.78125	0.536585366
sgol1	0.78125	0.672727273
plekhh1	0.78125	1.037037037
ppp1r14aa	0.78125	1.051136364
rasa4	0.78125	1.12244898
si:ch211-236l14.4	0.78125	1.2

ppp4r2a	0.78125	1.505050505
si:ch73-269m23.4	0.781094527	0.810055866
rcor2	0.780952381	0.455555556
si:ch211-222k6.3	0.780952381	1.6
hmga2	0.78093076	0.899186992
hprt1	0.780927835	1.618374558
si:ch211-246e12.3	0.780821918	0.86259542
si:dkey-30c15.10	0.780821918	1.135135135
nfe2l2a	0.780753968	2.0856102
fbln5	0.780701754	0.769230769
pllp	0.78057554	1.069090909
pex2	0.780487805	1.482758621
atp2a2b	0.780361757	0.551971326
hemk1	0.78030303	1.074766355
dusp3a	0.78030303	1.28358209
gba2	0.78021978	0.823943662
ckba	0.780201342	1.135080645
exosc8	0.780141844	0.888157895
sema3gb	0.780104712	0.94214876
fen1	0.780104712	1.063694268
ezra	0.780082988	0.645502646
elna	0.78	0.772727273
si:dkeyp-97b10.3	0.78	0.780487805
mocs1	0.78	1.09375
si:dkey-190e14.2	0.779874214	0.984962406
si:ch73-91k6.6	0.779816514	1.344444444
hsd17b8	0.779661017	0.776315789
sgpp1	0.779661017	0.95
ctbs	0.779661017	1
slc25a44a	0.779661017	1.277777778
trmt61a	0.779661017	1.361702128
nol8	0.779661017	1.777777778
si:rp71-1c10.7	0.779527559	1.295238095
tk2	0.779527559	1.5
si:dkey-22o12.7	0.779497099	1.378125
si:dkey-266f7.9	0.779411765	0.942307692
LYRM4 (1 of many)	0.779411765	1.238095238
nipsnap3a	0.779220779	1.014285714
crlf1a	0.779220779	1.117647059
itih3b	0.779220779	1.5
si:dkey-122a22.2	0.779220779	1.655172414
FP236526.1	0.779069767	0.905660377
wdr48a	0.779069767	1.218181818
pitx1	0.779005525	0.630872483
nrgnb	0.779005525	1.13125
kctd9b	0.778947368	0.95
si:ch73-281k2.5	0.778846154	0.935483871
myo1ea	0.778846154	1.179104478
hnrnpd	0.778699862	1.275789474
phb2b	0.778675283	1.360986547
creb3l2	0.778625954	1.15625
plcxd3	0.778443114	0.822033898
ebna1bp2	0.77827051	1.800653595
cldne	0.778037383	1.257641921
slc39a8	0.777777778	0.685714286
ccdc186	0.777777778	1.12345679
si:ch73-62l21.1	0.777777778	0.5
utp15	0.777777778	0.814371257
rps6ka5	0.777777778	0.826086957
tchp	0.777777778	0.836065574
elovl8b	0.777777778	0.845070423

nbas	0.777777778	0.848484848
thraa	0.777777778	0.871794872
CU929447.2	0.777777778	0.875
aak1a	0.777777778	0.909090909
cart3	0.777777778	0.914893617
klhdc2	0.777777778	0.965986395
ccdc85a	0.777777778	1.023809524
smpd2a	0.777777778	1.086021505
lyn	0.777777778	1.111111111
ccdc130	0.777777778	1.15
cfhl5	0.777777778	1.155555556
msl1a	0.777777778	1.227272727
nifk	0.777777778	1.466463415
trmo	0.777777778	1.615384615
snapc3	0.777777778	1.6875
snx18b	0.777777778	1.7
crtac1b	0.777777778	0.6875
nudt15	0.777777778	0.714285714
fhdc2	0.777777778	0.8125
tbx22	0.777777778	0.866666667
fhod1	0.777777778	0.962962963
haus1	0.777777778	1.025641026
hmgxb3	0.777777778	1.083333333
bard1	0.777777778	1.090909091
entpd2a.1	0.777777778	1.153846154
pla2r1	0.777777778	1.238095238
si:dkey-28a3.2	0.777777778	1.277777778
prc1b	0.777777778	1.30952381
myo1f	0.777777778	1.4
si:ch211-214e3.5	0.777777778	1.422222222
tapt1a	0.777777778	1.5
si:ch73-347e22.8	0.777777778	1.637931034
tescb	0.777777778	1.642857143
si:ch1073-90m23.1	0.777777778	1.692307692
hspa4a	0.777464789	0.838709677
capn3a	0.777365492	0.661237785
cthrc1a	0.777227723	0.676470588
unc119a	0.777202073	1.225165563
sh3gl1a	0.777173913	1.27480916
prpf4ba	0.777108434	1.068627451
myh9a	0.777078086	1.480392157
pacsin1a	0.776937618	1.338403042
cpamd8	0.776859504	1.012048193
barx2	0.776785714	1.580246914
hmx3a	0.776699029	1.46875
lipia	0.776699029	1.52
pfkmb	0.776666667	1.481981982
six4a	0.776595745	0.884615385
COL18A1 (1 of many)	0.776595745	0.942857143
mrps18b	0.776566757	1.388059701
fahd1	0.776536313	0.775862069
rgl3a	0.776470588	1.666666667
atg4c	0.776470588	1.921568627
mybbp1a	0.776435045	0.917355372
msna	0.776367188	1.437869822
pdzk1ip1	0.776315789	0.819672131
myo9b	0.776315789	1.018181818
slc2a1a	0.776315789	1.055045872
zgc:113210	0.776315789	1.36
sybl1	0.776119403	1.784810127
aim1b	0.775862069	0.928571429

nlrc3l	0.775862069	1.918918919
dsc2l	0.775800712	1.243093923
cldn15la	0.775735294	1.387779083
TMEM38B	0.775700935	0.67816092
crim1	0.775700935	0.878378378
zgc:174938	0.775641026	0.917241379
cltb	0.775641026	1.051948052
FP085428.1	0.775510204	0.424242424
PARD3B	0.775510204	0.764705882
si:dkey-28n18.9	0.775510204	1.217391304
aldh3a1	0.775510204	1.5
fxn	0.775510204	1.8
zgc:113295	0.775510204	1.9
htra1b	0.775362319	0.981651376
si:ch1073-309n14.1	0.775362319	1.827160494
gins4	0.775280899	0.742268041
si:ch211-160d20.6	0.775280899	0.825
plcd1a	0.775280899	1.380952381
ggh	0.775280899	1.928571429
si:dkey-181m9.5	0.775252525	1.553435115
uox	0.775147929	1.233400402
nhlh2	0.775061125	1.360594796
fbxl22	0.775	0.779411765
ppm1db	0.775	0.867924528
EPS8L2	0.775	0.878787879
syt16	0.775	0.894736842
hoxc10a_1	0.775	0.971428571
myl2b	0.775	1.160493827
smim1	0.775	1.25
si:dkeyp-38g8.5	0.775	1.583333333
cav1	0.774968394	1.697286013
atp1b2b	0.774907749	0.788235294
hp_2	0.774877651	1.285171103
zgc:92107	0.774834437	0.877192982
pbxip1b	0.774774775	0.835365854
parp2	0.774774775	1.057471264
smarcad1a	0.774774775	1.285714286
bcl2b	0.774725275	2.303370787
fap	0.774691358	1.154471545
klf6b	0.774647887	1.803921569
cds1	0.774590164	1.73943662
plk1	0.774545455	0.851162791
tsku	0.774509804	1.298780488
elp3	0.77443609	1.038095238
kcnc4	0.774193548	0.666666667
slc39a5	0.774193548	0.876712329
myrf	0.774193548	1.078431373
drd2b	0.774193548	1.2
triobpa	0.774193548	1.318181818
CU469384.1	0.774193548	1.433333333
SYDE1	0.774193548	1.739130435
fstl3	0.774074074	1.393305439
tomm70a	0.774032459	1.542914172
col4a2	0.773913043	0.830721003
slc4a1b	0.773913043	1.212765957
coa7	0.77385159	1.081196581
tmem70	0.773809524	1.062111801
pfkfb4a	0.773809524	1.657407407
si:ch211-222e20.4	0.773809524	1.913043478
efr3a	0.773722628	1.115384615
rpz4	0.773722628	1.34

aoc2	0.773696682	1.224519941
pigf	0.773662551	1.385
ddc	0.773584906	0.657142857
fam117bb	0.773584906	0.717948718
si:ch73-6k14.2	0.773584906	0.723684211
gramd1c	0.773584906	1.727272727
gpnmb	0.773480663	1.186781609
PRODH2	0.7734375	1.04
arntl2	0.773333333	0.555555556
phyhipla	0.773333333	0.930232558
trnau1apa	0.773333333	1.469387755
dgat1a	0.773109244	0.645833333
serpinc1	0.773081201	1.540298507
c5	0.773076923	0.922131148
gata5	0.773049645	0.669421488
acvrl1	0.773006135	1.007142857
aqp8a.1	0.772959184	0.870792617
mylk4a	0.772870662	2.47752809
cfh	0.7728	1.166374781
fa2h	0.772727273	0.84375
kank4	0.772727273	1.170731707
lrmp	0.772727273	0.448275862
ITPRIPL2	0.772727273	0.631578947
SLC47A1 (1 of many)_1	0.772727273	1.03030303
si:dkey-238f9.1	0.772727273	1.149606299
hint3	0.772727273	1.197368421
zgc:158258	0.772727273	1.2
sh2d5	0.772727273	1.3125
spata18	0.772727273	1.411764706
e4f1	0.772727273	1.507936508
cpa6	0.772727273	1.6
ctps1a	0.77260274	0.950819672
mrpl35	0.772522523	1.261845387
si:ch211-195h23.3	0.772413793	1.341880342
fam89b	0.772357724	0.8
smo	0.772357724	1.107843137
gga3	0.772357724	1.630769231
si:dkey-10p5.10	0.772321429	0.950672646
mpz	0.772277228	0.666666667
ntmt1	0.772151899	1.121546961
ucmaa	0.772151899	0.835164835
lepr	0.772151899	1.079365079
phpt1	0.772058824	2.542857143
MAIP1	0.772020725	1.333333333
crybb1l3	0.771929825	0.76
lmod2b	0.771929825	0.931818182
fam175a	0.771929825	1.1875
tnfaip8l1	0.771929825	1.236842105
rock2b_1	0.771929825	1.290909091
antxr2a	0.771929825	1.55
capn5a	0.771929825	0.910344828
ccndbp1	0.771929825	1.346456693
calb2b	0.771871539	0.545338442
nip7	0.771604938	1.268
si:dkey-16l2.16	0.771604938	1.516949153
cubn	0.771428571	0.44
pacs1a	0.771428571	0.875
kif5bb	0.771428571	0.94972067
irx4b	0.771428571	1.064516129
zgc:63882	0.771428571	1.208333333
bach1b	0.771428571	1.365079365

abce1	0.771428571	0.907051282
si:ch211-206k20.5	0.771300448	1.105820106
gnl3	0.771186441	1.412234043
prkra	0.771084337	0.828282828
nkx1.2lb	0.771084337	1.018867925
zgc:63863	0.771084337	1.34
coro1a	0.770992366	0.638888889
anp32e	0.770978491	0.772978959
homer3	0.770949721	1.846938776
c1ql3a	0.770833333	0.53125
hps1	0.770833333	1.388888889
fam102aa	0.770833333	1.857142857
myo10	0.770642202	0.8
taz	0.770642202	1.460526316
tes	0.770588235	0.890909091
commd9	0.77037037	1.639175258
nrn1a	0.770247934	1.651399491
tpbga	0.770114943	1.305555556
aqp1a.1	0.770032573	0.800256082
cx39.9	0.77	1.125
slco2a1	0.769911504	0.921568627
eps15	0.76969697	1.049586777
mapk8ip2	0.769662921	0.329896907
ltv1	0.769647696	1.093023256
foxc1b	0.769565217	0.714932127
si:dkey-81l17.6	0.769547325	1.037037037
fkbp11	0.76953125	0.9375
s100v2	0.76949443	1.142259414
epyc	0.769360269	0.873267327
bcan	0.769230769	0.574074074
hao2	0.769230769	0.904761905
osbpl5	0.769230769	0.924528302
efna3a	0.769230769	0.962962963
pax9	0.769230769	1.018348624
ppp1r8a	0.769230769	1.103448276
chst2b	0.769230769	1.222222222
pgpep1	0.769230769	1.581395349
si:dkey-261i16.5	0.769230769	0.723404255
svep1	0.769230769	0.829268293
BX927282.1	0.769230769	0.928571429
CR394535.1	0.769230769	1.058823529
KCP	0.769230769	1.075
stra6l	0.769230769	1.15
noa1	0.769230769	1.21875
si:dkey-112e17.1	0.769230769	1.235294118
gja3	0.769230769	1.25
zgc:198329	0.769230769	1.4
si:ch211-51c14.1	0.769230769	1.537974684
cplx4c	0.769230769	1.692307692
si:dkey-189o20.1	0.769230769	1.7
fam78aa	0.769230769	1.909090909
mydgf	0.768729642	1.523206751
stk17b	0.768707483	0.862385321
si:ch211-270n8.1	0.768656716	0.599264706
phf21aa	0.768595041	0.96
c1qtnf4	0.768518519	1.030456853
zgc:103508	0.768518519	1.566666667
si:ch211-229d2.5	0.768339768	1.694570136
eps8l3a	0.768292683	0.720930233
znf1010	0.768292683	0.794117647
si:cabz01113374.2	0.768292683	1.05

stx8	0.768292683	1.455696203
fam45a	0.768292683	2
ddt	0.768149883	1.736925515
stim1b	0.768115942	0.827586207
xdh	0.768115942	1.1625
pex13	0.768115942	1.336734694
bpnt1	0.768060837	1.317708333
aspn	0.768025078	1.274590164
kdr	0.767857143	0.837209302
wu:fb55g09	0.76778784	1.319469027
tmem178b	0.767676768	0.47826087
sfxn1	0.767676768	0.775
ddah2	0.767634855	0.871287129
acta1a	0.767624811	0.64362851
cast	0.76746507	1.194701987
plxnb2a_2	0.76744186	0.434782609
si:ch211-196f5.2	0.76744186	0.481481481
nfasca	0.76744186	0.614285714
eng1a	0.76744186	0.933333333
thumpd2	0.76744186	1.048780488
nemp1	0.76744186	1.161290323
RASA2	0.76744186	1.181818182
zgc:114174	0.76744186	1.264705882
impg2a	0.76744186	1.704545455
pak1ip1	0.767213115	1.270588235
zgc:174904	0.767195767	1.644444444
g6pca.1	0.767123288	1.574585635
dynlt1	0.767123288	0.651515152
abcb11b	0.767123288	0.931818182
RAP1GAP (1 of many)	0.767123288	1.089285714
epd	0.767096774	0.954218107
sgcd	0.767015707	1.609561753
cdk5r2b	0.766990291	0.581395349
CSGALNACT1 (1 of many)_2	0.766990291	1.029850746
inpp5ka	0.766917293	1.224299065
si:ch1073-443f11.2	0.766917293	1.294736842
dao.2	0.766839378	0.979020979
nt5c1bb	0.766666667	1.583333333
SGPP2	0.766666667	0.6
rem1	0.766666667	0.765625
baiap2l2a	0.766666667	0.782608696
zgc:112332	0.766666667	0.838709677
orc1	0.766666667	0.852459016
CU655961.6	0.766666667	1
xkr8.2_1	0.766666667	1.126582278
mid1	0.766666667	1.266666667
ulk1a	0.766666667	1.404761905
si:ch211-15d5.12	0.766666667	1.484848485
tex2l	0.766666667	1.666666667
fpgt	0.766666667	2
mia	0.766609881	1.118518519
cacna2d1a	0.766536965	1.317365269
pttg1ipa	0.766519824	2.012121212
eef2a.1	0.766423358	1.453488372
adgra2	0.76635514	0.86746988
zgc:153990	0.76635514	0.930434783
abcf2a	0.766304348	1.237947123
zgc:103586	0.766304348	1.448818898
nhsl1a	0.766233766	0.533333333
ehd1b	0.766062603	0.836772983
odam	0.765957447	0.5

comtb	0.765957447	0.660377358
ssh1a	0.765957447	0.833333333
zgc:86764	0.765957447	0.881578947
borcs5	0.765957447	1.1
taf1a	0.765957447	1.153846154
wnt2	0.765957447	1.2
KCNIP4 (1 of many)_2	0.765957447	1.272727273
rapgef5b	0.765957447	1.423076923
snx24	0.765957447	1.888888889
stmn1a	0.765834933	1.321933962
ube2kb	0.765822785	1.425925926
slc4a1a	0.765765766	0.552238806
tsta3	0.765765766	1.012048193
golga4	0.765765766	1.077922078
NDUFAF8	0.765765766	1.247933884
dok7	0.765625	0.957446809
cep85l	0.765625	1.260869565
rbsn	0.765625	1.833333333
rilpl2	0.765625	2.162790698
asb5b	0.765432099	1.184397163
cyb561a3a	0.765432099	1.403846154
gar1	0.765333333	1.05511811
ppa1a	0.765306122	0.680851064
polr1b	0.765306122	0.951807229
lpar1	0.765306122	1.86
dhtkd1	0.765258216	0.916666667
cdc42ep5	0.765151515	1.771428571
rgs16	0.765060241	1.781553398
shha	0.764705882	0.3
foxg1d	0.764705882	0.516129032
epb41b	0.764705882	0.647058824
si:ch211-255f14.2	0.764705882	0.76
cblc	0.764705882	0.861111111
asmt2	0.764705882	0.864864865
cnksr2a	0.764705882	0.875
si:ch1073-358c10.1	0.764705882	0.9
fam198b	0.764705882	1.036363636
zgc:100997	0.764705882	1.066666667
rad52	0.764705882	1.333333333
alkbh7	0.764705882	1.752688172
si:rp71-1c10.11	0.764617691	1.388254486
ppfibp1a	0.764397906	0.97260274
palm2_1	0.764150943	1.827586207
rgs5a	0.764127764	1.520134228
si:ch211-117m20.4	0.764044944	0.754716981
vrk3	0.764044944	1.865384615
dpm3	0.764	1.379146919
cacna2d2a	0.763888889	0.685714286
si:ch73-352p4.8	0.763888889	1.119047619
ARMC6	0.763888889	1.342105263
rnd3b	0.763819095	1.071090047
ndc80	0.763779528	1.278350515
NFASC (1 of many)	0.763636364	0.857142857
frmpd3	0.763636364	0.870967742
CABZ01084935.1	0.763636364	0.898305085
cabp7b	0.763636364	1.84
calr3b	0.763522013	1.366935484
si:ch73-197b13.1	0.76344086	0.8
atrip	0.763157895	0.736111111
rpusd2	0.763157895	1
nagpa	0.763157895	1.098039216

pigk	0.763157895	1.326086957
fkbp7	0.763157895	1.115511551
pmm2	0.763033175	1.349315068
mrpl1	0.76300578	1.354037267
gpr39	0.762987013	0.673913043
tnfrsf19	0.762962963	1.204545455
plk2a	0.762820513	1.109090909
pole	0.762711864	0.704545455
si:ch1073-147h9.1	0.762711864	1.14084507
lhpp	0.7625	1.329411765
lhfpl2a	0.7625	0.611764706
PTGFRN (1 of many)_2	0.7625	0.742857143
zc3h10	0.7625	0.75
hoxb10a	0.7625	0.757142857
isg20l2	0.7625	1.280701754
alpk3a	0.7625	2.02
zgc:110339	0.762335526	0.87211094
SENP1	0.762237762	1.133928571
MYO1D	0.762237762	1.573529412
hyou1	0.762057878	0.743119266
chata	0.761904762	1.715909091
cyp17a1	0.761904762	0.64516129
SAC3D1	0.761904762	0.722222222
ponzr6	0.761904762	0.808823529
IKZF2	0.761904762	0.894736842
zgc:66433	0.761904762	0.97080292
cx47.1	0.761904762	1.090909091
si:ch211-225h24.2	0.761904762	1.103448276
gjd1b	0.761904762	1.214285714
pde5ab	0.761904762	1.222222222
lhx8b	0.761904762	1.24
zgc:174688	0.761904762	1.282608696
htr1ab	0.761904762	1.421052632
agtrap	0.761904762	1.454545455
sec22ba	0.761904762	1.488888889
tmem186	0.761904762	1.529411765
vsig10	0.761904762	1.588888889
cradd	0.761904762	1.941176471
mns1	0.761904762	1.944444444
si:ch211-149k12.3	0.761904762	2.181818182
timp4a	0.761904762	2.5
ccdc80l2	0.761658031	1.248062016
mapre3a	0.761658031	1.265957447
mybpc2a	0.761538462	1.450704225
asl	0.76146789	0.927835052
camk1b	0.76146789	1.274193548
prune	0.761363636	1.510204082
sept8b	0.76119403	0.587628866
FRRS1 (1 of many)	0.76119403	1.014492754
fech	0.76119403	1.196428571
abcd3b	0.761061947	1.661016949
arl3l2_1	0.761006289	0.587155963
slc15a2	0.760869565	0.85915493
si:cabz01071907.1	0.760869565	0.888888889
si:ch211-212k18.9	0.760869565	1.04109589
bbs1	0.760869565	1.105263158
mcmbp	0.760869565	0.74137931
peo1	0.76056338	0.75
wdyhv1	0.76056338	3.913043478
znf385a	0.760416667	0.714285714
pola1	0.760330579	0.840909091

slc10a7	0.760330579	1.123893805
slitrk6	0.760330579	1.285714286
slc7a5	0.760233918	1.048387097
pwp1	0.760147601	0.975
cd164	0.760104302	1.438979964
BAZ2B (1 of many)	0.76	0.514285714
agfg1a	0.76	0.692307692
si:ch211-255f4.7	0.76	0.791666667
spred2a	0.76	0.966666667
map2k5	0.76	1
ntn1a	0.76	1.098765432
spns3	0.76	1.111111111
mapkapk2b	0.76	1.111111111
c1ql3b_1	0.76	1.166666667
im:7136021	0.76	1.2
eif2ak2	0.76	1.272727273
zgc:158222	0.76	1.275641026
stk10	0.76	1.302631579
pcdh1g3	0.76	1.466666667
jmjd4_2	0.76	1.583333333
cyldl	0.76	1.75
si:dkey-33c14.6	0.76	2.285714286
FNDC4 (1 of many)	0.76	0.996226415
slc6a5	0.759803922	0.481818182
ppan	0.75974026	1.213333333
si:ch211-150i13.1	0.759615385	1.203125
mmp11a	0.759493671	0.74375
traf2b	0.759493671	1.333333333
svbp	0.759398496	1.52
reep3a	0.759398496	1.888888889
igfbp2a	0.759259259	0.925888325
cdh26.1	0.759259259	1.275
b3gnt5a	0.759124088	1.171806167
nsmce4a	0.759036145	1.066666667
suv39h1b	0.759036145	1.253968254
si:ch211-284e20.4	0.758928571	0.755555556
lmod3	0.758865248	1.692307692
acss3	0.75862069	0.485714286
tep1	0.75862069	0.615384615
slc8a4b	0.75862069	1.071428571
zgc:113452	0.75862069	1.384615385
cyp4t8	0.75862069	1.55
nsmce1	0.75862069	2.095238095
si:dkey-238c7.16	0.75862069	0.969230769
col6a3_1	0.758522727	0.965665236
cyp2p8	0.758426966	1.59375
eif4ebp3l	0.758342923	1.007900677
MAP7	0.758333333	1.106666667
zgc:77838	0.758241758	0.916666667
znf618	0.758241758	1.037037037
pet100	0.758122744	1.668292683
flrt1b	0.758064516	0.621621622
slc1a2a	0.758064516	0.840277778
lrch3	0.758064516	1.305555556
stk24a	0.758064516	1.40625
klhl24b	0.758064516	1.677248677
thoc2	0.758007117	1.213483146
clu	0.757894737	0.958563536
si:dkey-8k3.2	0.757894737	0.980952381
zgc:92287	0.757894737	1.005076142
SRL (1 of many)	0.757800892	1.92722372

ALX3	0.757575758	0.84
fam83e	0.757575758	1.15942029
fam175b	0.757575758	1.194029851
syce2	0.757575758	1.219512195
ypel2b	0.757575758	1.24137931
GID4	0.757575758	1.255813953
entpd1	0.757575758	1.272413793
tsnaxip1	0.757575758	1.275862069
shbg	0.757575758	1.325
si:dkey-9i23.16	0.757575758	1.628571429
smpdl3a	0.757575758	2.333333333
tmem110l	0.757475083	1.458041958
si:dkey-222f8.3	0.757446809	1.122807018
cldnf	0.757352941	1.543478261
prkar1b	0.757246377	0.950276243
tp53i11a	0.757225434	1.409090909
arfip2b	0.757225434	1.442622951
nupr1	0.757151197	2.682758621
zgc:195050	0.757142857	0.484210526
OXSM	0.757142857	0.732142857
rnf113a	0.757142857	1.1
aurkb	0.757062147	0.658986175
msrb3	0.75698324	1.047272727
abcc4	0.756880734	1.395522388
gfpt1_2	0.756862745	1.201219512
pak4	0.756756757	1
nabp1a	0.756756757	1.265822785
cdx1b	0.756756757	0.272727273
chrm2a	0.756756757	0.361111111
dpp4	0.756756757	1.018867925
macrod1	0.756756757	1.083333333
vgll2a	0.756756757	1.210526316
cldn5a	0.756756757	1.287356322
anks1aa	0.756756757	1.45
ttc39a	0.756756757	1.642857143
tmem98	0.756756757	1.851851852
slc38a5b	0.756493506	1.031746032
mapk14a	0.756476684	1.202898551
klhl4	0.756410256	0.815789474
mfap5	0.756410256	1.204081633
col4a6	0.756393001	0.945533769
zpr1	0.756345178	1.363057325
si:ch211-145n14.3	0.756302521	0.985507246
si:dkeyp-57d7.4	0.75625	0.753968254
osbpl3b	0.756097561	0.777777778
mmel1	0.756097561	1.291666667
rad18	0.756097561	1.565217391
lrrc8da	0.756097561	1.75
g0s2	0.756097561	1.967741935
sntb1	0.756097561	0.696629213
nop16	0.755952381	2.321243523
ms4a17a.7	0.755813953	0.819047619
boka	0.755813953	0.963414634
acsf2	0.755789474	0.759615385
masp2	0.755725191	0.613445378
rrn3	0.755681818	1.439252336
tmem200a	0.755555556	0.692307692
cacng6a	0.755555556	0.988764045
syk	0.755555556	1.027777778
lrpap1	0.755555556	1.675675676
si:ch73-6l19.2	0.755555556	4.84375

slc6a1a	0.755511022	0.452755906
glra3	0.755395683	1.086419753
tmem82	0.755319149	1.285714286
aldh3b1	0.755102041	1.030769231
BX005085.6	0.755102041	1.125
cyp7b1	0.755102041	1.258426966
dbnla	0.755102041	1.306666667
pcdh18b	0.755033557	0.644736842
si:ch211-196c10.12	0.754716981	2.1
abca1b	0.754716981	1.239495798
zgc:77739	0.75464684	1.179372197
abcc1	0.754545455	1.362318841
TESK1 (1 of many)_2	0.754385965	0.955555556
p3h4	0.754385965	1.151898734
alx4b	0.754385965	1.225806452
micall1a	0.754385965	1.310344828
dennd3a	0.754385965	1.59375
pck2	0.754237288	0.778361345
riok1	0.754098361	1.578947368
nkd3_5	0.754098361	0.779661017
itga2.3	0.754098361	1.666666667
vangl1	0.753846154	0.7125
gpr155a	0.753846154	0.945205479
hoxc11a	0.753846154	1.069767442
si:ch211-147g22.5	0.753846154	1.207317073
abraa	0.753846154	1.746987952
parla	0.753623188	1.276595745
piezo1	0.753623188	1.536585366
nop58	0.753605769	0.726823239
OLFM4 (1 of many)_1	0.753333333	1
sertad4	0.753246753	0.672727273
si:dkey-250l23.4	0.753246753	0.761904762
cnksr1	0.753246753	1.574074074
ptpn2a	0.753246753	1.640625
s1pr2	0.753138075	1.284946237
pla2g15	0.753094059	0.914830256
pmt	0.75308642	0.842857143
rcvrnb	0.75308642	1.173076923
scel	0.75304878	2.340527578
NANS (1 of many)	0.752941176	1.714285714
rbm12	0.752747253	0.783216783
snx13	0.752688172	1.148148148
acbd7	0.752515091	1.147420147
cyyr1	0.752427184	0.75
myo18aa_1	0.752380952	1.2125
tuft1b	0.752293578	1.583333333
alx4a	0.752293578	1.768115942
pik3cb	0.752066116	1.449275362
ntn5	0.751937984	1.094117647
GNAZ	0.751879699	0.631578947
trim55a	0.751879699	0.78343949
slc25a42	0.75177305	1.295454545
si:dkey-61n16.5	0.75177305	1.577319588
rgra	0.75170068	0.763819095
vwde	0.751227496	0.68429003
vwde	0.751227496	0.68429003
stk40	0.751054852	0.854748603
cyp7a1	0.75088968	0.687315634
sri	0.750849377	1.25974026
rhag	0.750440399	1.156060606
st8sia7.1	0.75	0.379310345

si:dkey-6n6.1	0.75	0.603174603
cntn3a.1	0.75	0.605263158
kidins220a	0.75	0.876923077
IQGAP3	0.75	0.882352941
col7a1	0.75	0.971428571
mthfd2l	0.75	1.041666667
mettl1	0.75	1.088235294
hsf4	0.75	1.157894737
s100u	0.75	1.487804878
ensab	0.75	1.589473684
si:dkey-147f3.4_2	0.75	1.625
rapgef5a	0.75	0.59375
zdhhc14	0.75	0.620689655
nsd1b	0.75	0.764705882
arpc1b	0.75	0.816176471
tbl2	0.75	0.843537415
kazald2	0.75	0.863636364
CABZ01058261.1	0.75	0.86627907
edar	0.75	0.888888889
mcf2a	0.75	0.9375
si:dkey-183n20.2	0.75	0.944444444
csf1a	0.75	0.961538462
gli3	0.75	1
klf7b	0.75	1.096774194
DLC1	0.75	1.126213592
pik3r2	0.75	1.1328125
kcnv2a	0.75	1.256410256
tp53i11b	0.75	1.311258278
uck2a	0.75	1.329268293
syt11b	0.75	1.554621849
si:dkeyp-120h9.1	0.75	1.626666667
si:dkeyp-110e4.11	0.75	1.652173913
zgc:158868	0.75	1.666666667
si:ch73-130a3.4	0.75	1.764705882
gas5	0.75	1.76744186
si:dkey-30k6.5	0.75	1.807692308
si:dkeyp-4c4.1_2	0.75	2.416666667
cabp5b	0.75	2.551724138
ponzr2	0.75	2.56
crfb12	0.75	3.4
zgc:110425	0.75	0.625
btd	0.75	0.682926829
oafa	0.75	0.773255814
si:dkey-231j24.3	0.75	0.8125
sgk494a	0.75	0.857142857
zgc:113340	0.75	0.866666667
slc15a4	0.75	1
txnipb	0.75	1.041474654
dck	0.75	1.075
crb3b	0.75	1.24742268
cdhr1a	0.75	1.268292683
si:dkey-228a15.1	0.75	1.285714286
CR391998.1	0.75	1.5
si:ch211-261n11.7	0.75	1.832061069
smc6	0.75	1.9
rilp	0.75	2.196969697
rhoca	0.749347258	1.265625
rcn3	0.749174917	1.626760563
hmgcs1	0.748898678	0.872093023
tmem41ab	0.748878924	1.340540541
rab4a	0.748792271	1.687022901

vcanb	0.748677249	1.191897655
si:dkey-38p12.3	0.748387097	0.925925926
setdb1a	0.748387097	1.082352941
adgrg6	0.74829932	1.008849558
amigo1	0.748201439	1.24691358
nat8l	0.748148148	1.407008086
tradd	0.74796748	1.571428571
RASSF8 (1 of many)	0.74789916	1.194805195
rab6ba	0.747851003	1.880681818
h6pd	0.747747748	0.896551724
KCNA3	0.747663551	0.888888889
atp2b1b	0.747619048	0.553191489
ckmt2a	0.747600768	0.637059724
pard6gb	0.747252747	0.96969697
ppm1na	0.747252747	1.107142857
PKN2 (1 of many)	0.747252747	1.122807018
wapla	0.747252747	1.295454545
tmem263	0.747252747	1.523316062
mbd1b	0.747191011	1.298969072
ngef	0.747126437	0.794117647
egfl7	0.747126437	1.020408163
PLEKHB1	0.747126437	1.28358209
grhl2a	0.747126437	1.685185185
prph2b	0.747019868	0.330357143
wsb1	0.746892655	1.093150685
calml4a	0.746753247	1.157534247
ablim1b	0.746724891	1.184
mgaa	0.746666667	1
esyt3	0.746666667	1.22
hsp90b1	0.746657754	0.760364842
cox4i1l	0.746478873	0.666666667
zgc:112964	0.746478873	0.865853659
slc5a2	0.746478873	1.169014085
rac1b	0.746478873	1.178438662
rspo3	0.746478873	1.295454545
emc9	0.746411483	1.32460733
slc43a3b	0.746376812	0.857142857
nipa2	0.746268657	1.258741259
cpped1	0.746153846	1.228571429
zgc:153284	0.74611399	0.967871486
CAPN1 (1 of many)_1	0.74611399	1.01875
bicc1a	0.746031746	0.894736842
col7a1l	0.746031746	0.962264151
haus6	0.746031746	1
zgc:136493	0.746031746	1.239130435
prdm8b	0.745856354	0.806896552
shcbp1	0.745762712	1.25
ighmbp2	0.745762712	1.5625
si:ch211-243a20.3	0.745535714	0.926553672
cracr2b	0.745454545	0.557692308
kcnab2a	0.745454545	0.615384615
rxrga	0.745454545	0.666666667
rgs7bpb	0.745283019	1.219512195
pls3	0.745145631	1.172839506
CR524823.1	0.745098039	0.421052632
adamts18	0.745098039	0.633333333
foxe1	0.745098039	0.784313725
sulf1	0.745098039	0.81981982
tceb2_1	0.745098039	0.890909091
tinf2	0.745098039	0.97826087
TMEM179B (1 of many)	0.745098039	1.041666667

arhgef28	0.745098039	1.045454545
tmem238a	0.745098039	1.234177215
smtnl1	0.745098039	1.759259259
knca7	0.745	1.679104478
si:dkey-281i8.1	0.744897959	1.80733945
bub3	0.744855967	0.688372093
CABZ01084566.1	0.744680851	0.761904762
zgc:113516	0.744680851	0.926470588
JMJD8	0.744680851	1.153846154
lrit2	0.744680851	1.37037037
igf1	0.744680851	2.157894737
pnkp	0.744444444	0.797101449
krt222	0.744444444	0.967741935
kif15	0.744444444	0.971830986
cgnl1	0.744444444	1.02173913
spon2a	0.744186047	0.787878788
si:dkey-82f1.1	0.744186047	0.973684211
si:ch211-196p9.1	0.744186047	1.688888889
kitb	0.744186047	1.833333333
scin	0.744186047	0.697247706
palm2_2	0.744186047	1.231707317
anks4b	0.743902439	0.862068966
adck5	0.743902439	1.181818182
zgc:110626	0.743697479	1.299435028
igfbp7	0.74368231	1.059808612
rpz5	0.743661972	1.25
slc46a1	0.743589744	0.875
ccdc160	0.743589744	1.222222222
slc16a1a	0.743589744	1.285714286
tmem222b	0.743589744	1.304347826
masp1	0.743589744	1.35483871
si:dkey-16n15.3	0.743589744	1.5
pus7l_2	0.743589744	1.6
myh6	0.743589744	1.739130435
zgc:153215	0.743589744	2.444444444
ing2	0.743455497	0.830065359
lrrc8c_2	0.743421053	1.819148936
si:dkey-17e16.8	0.743362832	1.512820513
tjp2a	0.743315508	0.827338129
MRPL55	0.743243243	1.289256198
MYADM (1 of many)_1	0.743169399	0.96460177
rreb1b	0.743119266	1.058823529
dhrs11b	0.743055556	1.290322581
zgc:114119	0.742971888	1.206185567
nsmfb	0.742857143	0.5
si:ch211-22d5.2	0.742857143	0.558823529
si:ch211-210g13.5	0.742857143	0.583333333
nrg1	0.742857143	0.64
si:ch211-193l2.3	0.742857143	0.803571429
plp1a	0.742857143	0.882352941
CNGA1 (1 of many)	0.742857143	1.083333333
mtpap	0.742857143	1.155555556
ccdc141	0.742857143	1.166666667
add3b	0.742857143	1.244897959
ptf1a	0.742857143	1.416666667
zgc:171971	0.742857143	1.432432432
ccsapb	0.742857143	1.462068966
gxylt1b	0.742857143	1.5
si:dkey-253d23.2	0.742857143	1.6
zgc:113220	0.742857143	1.634615385
rnf144aa	0.742424242	1.175

snx15	0.742424242	1.735294118
si:ch211-212k18.8	0.742331288	0.884615385
dhrs9	0.7421875	1.014018692
trabd	0.7421875	1.047619048
col17a1b	0.741935484	0.919117647
cisd2	0.741935484	1.46875
chrna4b	0.741935484	0.714285714
opn4b	0.741935484	0.75
gpr78a	0.741935484	1
hs3st1l2	0.741935484	1.018181818
FP102464.1	0.741935484	1.12244898
plekhj1	0.741935484	1.594594595
ICA1	0.741935484	1.953488372
gfi1aa	0.741935484	2.230769231
lnx2b	0.741721854	0.974789916
agtr2	0.741721854	1.087301587
frem1a	0.741666667	1.06122449
prr14	0.741496599	1.348314607
casc5	0.74137931	0.80952381
tmem88a	0.74137931	1.088888889
cep170b	0.74137931	1.125
SLC27A3	0.74137931	1.416666667
bmp2a	0.741176471	0.795698925
deptor	0.740890688	1.158940397
pcxa	0.740837696	0.640569395
smarcd2	0.740740741	0.914285714
g2e3	0.740740741	1.205882353
tnnt2b	0.740740741	1.646153846
SLC6A3	0.740740741	0.333333333
megf8_2	0.740740741	0.47826087
si:ch73-281f12.4	0.740740741	0.555555556
si:dkey-280e8.1	0.740740741	0.733333333
SBK2	0.740740741	0.846153846
cfap45	0.740740741	0.928571429
hvcn1	0.740740741	1.448979592
poc5	0.740740741	1.625
nt5dc3	0.740740741	1.928571429
hgfb	0.740740741	2.454545455
crfb1	0.740740741	2.5
si:ch211-209l18.2	0.740740741	2.8
jade3	0.740384615	0.876712329
pdap1b	0.740359897	1.552631579
npm1a	0.740268456	1.216888889
fam57bb	0.74025974	0.5
thbs4b	0.740206186	1.042821159
ddx51	0.74015748	1.04587156
gpr37l1a	0.74	0.648648649
prom1a	0.74	0.858695652
mocs3	0.74	0.935483871
tfap2c	0.739884393	1.291666667
slc7a10b	0.739726027	0.3125
grp	0.739726027	1.046875
fam92a1	0.739726027	1.681818182
matn1	0.739642857	0.723136496
rhogb	0.739495798	0.741071429
nme7	0.739495798	1.021052632
sec14l7	0.739495798	1.304347826
itga3b	0.739336493	1.284482759
zic6	0.739130435	0.744680851
crhr1	0.739130435	0.846153846
ddx11	0.739130435	0.894736842

si:ch211-12e13.12	0.739130435	1
pcgf5a	0.739130435	1.157894737
erap1a	0.739130435	1.210526316
tnni2a.3	0.739130435	1.387096774
ak7a	0.739130435	1.470588235
znf1143	0.739130435	1.625
pcdh12	0.739130435	1.681818182
tnw_1	0.739083558	1.076691729
crabp2a	0.738872404	1.680628272
slc35c2	0.738853503	1.188034188
rpf1	0.738831615	1.811688312
jam2b	0.738738739	1.07
si:dkeyp-84f3.9	0.738738739	1.271186441
pdk2a	0.738724728	2.402234637
tsen2	0.738636364	1.127906977
tnnt2d	0.738520408	1.777046096
WDCP	0.738461538	0.758064516
sec61a1l	0.738396624	1.094936709
hnf1ba	0.738317757	0.632911392
chrnb1	0.738255034	1.333333333
esama	0.738095238	0.710144928
rpe65b	0.738095238	0.989361702
lgals9l3	0.738095238	1.009009009
si:ch1073-459b3.2	0.738095238	1.137931034
si:ch73-379j16.2	0.738095238	1.315789474
gsnb	0.738045738	0.857566766
rph3ab	0.737864078	0.918367347
slc18b1	0.737704918	0.725490196
THEM6 (1 of many)_1	0.737704918	0.735849057
myo1cb	0.737704918	1.030456853
sgpl1	0.737704918	1.226027397
si:ch211-74m13.3	0.737704918	1.232142857
ice1	0.737704918	1.254237288
rdh12l	0.737704918	1.977777778
cant1b	0.737556561	1.047008547
lsp1	0.737541528	0.796992481
ANK2	0.7375	0.555555556
thyn1	0.7375	0.83908046
prkcda	0.737430168	1.146153846
cyp27a7	0.737430168	1.472
itpka	0.737373737	1.194444444
gas1b	0.737373737	1.333333333
brms1la	0.737373737	1.797687861
NAALADL1	0.737288136	0.796296296
commd5	0.737288136	1.058823529
atp6ap1lb	0.737226277	1.308411215
gcgb	0.737142857	0.663461538
dennd2db	0.736842105	0.7
mamdc2a	0.736842105	0.72
fam161b	0.736842105	0.736842105
rgs3b	0.736842105	0.891891892
cpeb3	0.736842105	0.921568627
sass6	0.736842105	0.933333333
cntnap5a	0.736842105	0.956521739
si:dkey-266m15.7	0.736842105	1
tcf15	0.736842105	1.097560976
ccdc135	0.736842105	1.1
clic5b	0.736842105	1.112582781
si:ch73-156l6.2	0.736842105	1.142857143
ttc26	0.736842105	1.155172414
camkva	0.736842105	1.15625

mctp2a	0.736842105	1.166666667
VWA5A (1 of many)_2	0.736842105	1.210526316
znf1164	0.736842105	1.266666667
si:ch211-265o23.1	0.736842105	1.285714286
si:ch211-186e20.7	0.736842105	1.301886792
si:ch211-108c6.2	0.736842105	1.394736842
si:rp71-78h1.10	0.736842105	1.454545455
zgc:193593_2	0.736842105	1.461538462
ppp1r3aa	0.736842105	1.777777778
si:dkey-286j17.4	0.736842105	2
fgf19	0.736842105	2.615384615
ccdc167	0.736434109	1.542857143
hyal2b	0.736363636	0.958333333
rab11fip3	0.736111111	0.755102041
pid1	0.736111111	0.808988764
far1	0.736111111	1.294117647
six2a	0.735849057	0.640243902
si:ch211-232b12.5	0.735849057	0.820512821
sema3ga	0.735849057	0.85
fam69c	0.735849057	0.906666667
acbd4	0.735849057	1.097560976
arrdc1b	0.735849057	1.151515152
ticam1	0.735849057	1.523809524
twist1b	0.735849057	1.01734104
abi3bpb	0.735729387	1.078534031
si:ch211-157b11.14	0.735483871	1.72972973
atp1b1a	0.735424354	1.14422592
foxf2b	0.735294118	1.115384615
ccdc97	0.735294118	1.37037037
clrn2	0.735294118	1.681818182
cnfn	0.735135135	1.194285714
fcer1gl	0.735042735	0.866141732
si:dkey-11n6.3	0.734939759	0.712121212
lypd6	0.734939759	1.136363636
lrrc17	0.734870317	1.256603774
rom1a	0.734848485	0.393939394
zgc:136858	0.734848485	0.973214286
gbgt1l3	0.734848485	1.189781022
si:ch211-1a19.3	0.734693878	1.16
mpp2a	0.734693878	1.327586207
rdh8a	0.734597156	0.844339623
gal3st1a	0.734375	0.619047619
fgf11b	0.734375	0.714285714
cd3eap	0.734375	0.838709677
cnih3	0.734375	0.942307692
si:dkey-34m19.3	0.734375	1.227272727
si:dkeyp-12a9.5	0.734265734	1.032110092
zgc:158564	0.734219269	1.415204678
zgc:77651	0.734177215	0.8
atpaf1	0.734104046	1.761261261
klhl40a	0.733766234	1.848837209
si:ch211-39i22.1	0.733705773	1.077306733
imp3	0.73364486	2.013157895
neto1l	0.733333333	0.6875
si:ch211-197l9.2	0.733333333	0.714285714
si:dkeyp-19e1.3	0.733333333	0.857142857
slc27a1b_2	0.733333333	0.866666667
zgc:77112	0.733333333	1
imp4	0.733333333	1.082051282
c13h10orf11	0.733333333	1.2
l3mbtl3	0.733333333	1.3

rab33a	0.733333333	1.3
si:cabz01083838.1	0.733333333	1.333333333
NCEH1 (1 of many)	0.733333333	1.538461538
si:dkey-14k9.2	0.733333333	1.62745098
nek2	0.733333333	1.666666667
best4	0.733333333	1.666666667
znf770	0.733333333	2.035714286
si:dkey-242e21.4	0.733333333	2.153846154
slc35a3b	0.733333333	2.666666667
kif18a	0.733333333	0.6875
rmi1	0.733333333	0.738095238
A2ML1 (1 of many)_7	0.733333333	1.03030303
wwc1	0.733333333	1.146341463
pdp1	0.733333333	1.269503546
si:dkey-238i5.3	0.733333333	1.75
nckap5l	0.732758621	0.638554217
rab34a	0.732673267	1.527027027
lbr	0.732441472	0.751908397
snx8b	0.732394366	0.923076923
snupn	0.732394366	0.946236559
uhrf1	0.732283465	0.73015873
arl14	0.732142857	0.862068966
papd7	0.732142857	0.875
zgc:171740	0.732026144	1.351851852
si:ch211-203b8.6	0.731707317	0.892857143
RNPEPL1	0.731707317	0.925
si:ch211-154c21.1	0.731707317	1.44
si:ch211-225b7.5	0.731707317	2.891891892
dspa	0.731707317	1.064638783
syt9b	0.731543624	0.627272727
fam102ab	0.731543624	1.469387755
hapln1a	0.731506849	0.936827957
cdk5r1a	0.731481481	0.694915254
slco3a1	0.731343284	0.72
si:ch211-250c4.4	0.731343284	1.234042553
ca4b	0.73125	1.413114754
aep1	0.731197772	1.04587156
tmem176l.3a	0.731182796	1.875
rgs9a	0.730994152	0.797619048
CABZ01111496.1	0.730769231	0.8
nprl3	0.730769231	1.134615385
si:ch211-214b16.2	0.730769231	1.317073171
traf6	0.730769231	1.354166667
commd6	0.730769231	1.75
ccdc137	0.730769231	1.795698925
spon1a	0.730769231	0.785714286
zgc:113363	0.730769231	0.941176471
si:dkey-1b17.3	0.730769231	0.947368421
dok4	0.730769231	0.964285714
efemp2b	0.730769231	0.971428571
rrnad1	0.730769231	1
mesdc1	0.730769231	1.293333333
borcs8	0.730769231	1.471264368
si:ch211-207e14.4	0.730769231	1.8
btr05	0.730769231	1.866666667
klhl38b	0.730769231	2.538461538
kazald3	0.730612245	1.028708134
clpb	0.730434783	0.907894737
vwa2	0.730337079	0.992063492
eif3hb	0.73015873	0.788732394
slc26a6l	0.73015873	1.016393443

wdr74	0.73015873	1.20212766
si:ch211-153l6.6	0.73015873	1.451612903
dnajb2	0.73015873	1.785714286
lig1	0.729927007	1.348837209
hoxc11b	0.72972973	0.931034483
SLC25A29 (1 of many)	0.72972973	1.048780488
OMA1	0.72972973	1.115384615
si:dkey-224j12.6	0.72972973	1.285714286
zbtb43	0.72972973	1.461538462
atf5b	0.72972973	1.763358779
suclg2	0.729533679	0.946022727
nme6	0.729411765	0.784313725
lifra	0.729411765	1.291666667
dab2ipb	0.729166667	0.762711864
trim35-12	0.729166667	1.075471698
SLC47A1 (1 of many)_2	0.729166667	1.903225806
znf654	0.729032258	1.808988764
zgc:66484	0.728915663	1.178947368
zgc:77151	0.728682171	0.841584158
gabrb3	0.728571429	0.96031746
camk2a	0.728506787	0.710743802
zgc:92594	0.728395062	1.433333333
fabp7b	0.728323699	0.96
ndor1	0.72826087	1.163934426
fndc3ba	0.72826087	1.230769231
creld2	0.72826087	1.307692308
rhbdl2	0.728	1.175
pdk3a	0.728	2.018518519
sar1ab	0.727941176	1.491961415
pum3	0.727915194	2
sh3tc2	0.727891156	1.216981132
lamc3	0.72755418	1.062745098
vasnb	0.727272727	1.636363636
nme4	0.727272727	0.551724138
zgc:77778	0.727272727	0.606837607
rpusd4	0.727272727	0.875
flrt2	0.727272727	0.891891892
tfec	0.727272727	0.933333333
FAAH	0.727272727	0.966666667
f11r.2	0.727272727	1.013157895
ankrd16	0.727272727	1.037037037
zgc:101731	0.727272727	1.222222222
plekhg7	0.727272727	1.25
MAL (1 of many)_2	0.727272727	1.304568528
si:ch211-149l1.2	0.727272727	1.307692308
lrch2	0.727272727	1.416666667
si:dkey-11n14.1	0.727272727	1.428571429
ing5b	0.727272727	1.510638298
CCDC181	0.727272727	1.533333333
sdhaf4	0.727272727	1.545454545
si:ch73-269m23.5	0.727272727	1.619047619
zgc:174275	0.727272727	1.842105263
RNF14 (1 of many)_3	0.727272727	2.083333333
zpld1a	0.727272727	2.269230769
AKAP13 (1 of many)_2	0.727272727	0.844827586
top1l	0.727142857	1.30617284
rom1b	0.726907631	0.404651163
pfas	0.726666667	0.804054054
spegb	0.726618705	1.102272727
igfbp5b	0.726577438	1.152238806
scn4ab	0.7265625	0.943820225

si:cabz01068815.1	0.7265625	1.35
nr1i2	0.7265625	1.53125
slc6a4a	0.726415094	1.212121212
si:dkey-65b13.13	0.726315789	0.741666667
lpcat3	0.726277372	1.255952381
cd36	0.726190476	0.610429448
si:dkeyp-86h10.3	0.726098191	1.995283019
rnmtl1b	0.725806452	1.244444444
zdhhc21	0.725806452	1.852941176
si:ch1073-392o20.2	0.725752508	1.298245614
cd99	0.725714286	1.728813559
adgre5b.3	0.725490196	1.073170732
b3gnt5b	0.725490196	1.083333333
ZC3H7B (1 of many)	0.725490196	1.194444444
cst14a.1	0.725490196	1.243243243
rnf213a	0.725490196	1.5
si:dkey-21e13.3	0.725490196	1.541666667
nexn_2	0.72541744	1.132124352
fam169ab	0.725321888	1.397350993
aspa	0.725274725	1.666666667
prune2	0.725225225	2.146551724
si:ch211-243g18.2	0.72519084	0.923255814
thnsl2	0.725	0.710526316
si:ch73-108h4.3	0.725	0.764705882
gas1a	0.725	1.103542234
b4galnt3b	0.725	1.473684211
bambib	0.725	1.644067797
cx30.3	0.724637681	1
htra1a	0.724358974	0.878378378
fhl2a	0.724310777	0.878437048
lima1a	0.724242424	0.80565371
katnal1	0.724137931	0.5125
kcnf1a	0.724137931	0.833333333
si:ch73-335l21.1	0.724137931	0.896
hif1al2	0.724137931	1
NPFFR2 (1 of many)	0.724137931	1.066666667
rnf182	0.724137931	1.086956522
fmnl2a	0.724137931	1.27027027
opn5	0.724137931	1.304347826
si:cabz01070527.3	0.724137931	1.333333333
fam129aa	0.724137931	2.183673469
si:ch73-261i21.5	0.724137931	2.285714286
ms4a17a.9	0.724056604	0.989556136
mdh1ab	0.723938224	1.389057751
pgm2	0.723880597	0.932291667
glmnb	0.723809524	1.222222222
coro1ca	0.723744292	1.293040293
im:7150988	0.723606168	1.014833127
si:ch211-214j8.15	0.723577236	1.435897436
gch1	0.723577236	2.404255319
zbtb2b	0.723529412	1.022900763
gabrg2	0.723404255	0.640625
traf4b	0.723404255	1.130434783
manf	0.723320158	1.736111111
si:ch211-116m6.3	0.723214286	0.950617284
cbsa	0.723214286	1.314285714
nos1apa_1	0.723076923	0.693877551
dcn	0.723066755	0.720332577
cdc20	0.722972973	1.361904762
RNF115 (1 of many)	0.722891566	1.105263158
btbd3b	0.722689076	0.930555556

upb1	0.722488038	0.933884298
cpxm1b	0.722222222	0.606060606
ulk4_3	0.722222222	0.666666667
aplnra	0.722222222	0.6875
crygm2f	0.722222222	0.74522293
slc35f3b	0.722222222	0.75
nhsa	0.722222222	0.791666667
si:dkey-187a12.4	0.722222222	0.84
si:dkeyp-14d3.1	0.722222222	0.88
c6ast3	0.722222222	0.952380952
tmem37	0.722222222	0.952380952
cgna	0.722222222	1.081632653
slc44a5b	0.722222222	1.161764706
si:dkey-226m8.10	0.722222222	1.162790698
plcd1b	0.722222222	1.255102041
si:ch211-141i4.3	0.722222222	1.357142857
coil	0.722222222	1.463414634
slc17a9a	0.722222222	1.7
si:dkey-250d21.1	0.722222222	1.912280702
si:busm1-105l16.2	0.722222222	2
ccnd2a	0.721910112	1.75
arf4a_2	0.721804511	1.237623762
ndrg1a	0.721749696	0.985123967
cyp4v8	0.721698113	1.125748503
si:ch211-288d18.1	0.721649485	1.375
si:ch211-210k13.2	0.721649485	1.967213115
ptprea	0.721518987	1.372093023
slc26a1	0.721311475	0.971428571
pcolcea	0.721153846	0.708920188
zgc:112437	0.721153846	0.760869565
snx11	0.721153846	2.50877193
rnasekb	0.72095151	1.419243986
col27a1a	0.720930233	0.771428571
lgi3	0.720930233	0.777777778
oxsr1b	0.720930233	0.990909091
ano6	0.720930233	1.379310345
si:ch211-261n11.8	0.720930233	1.9
si:dkeyp-110c7.4	0.720930233	2.3
RDH13 (1 of many)_1	0.720670391	1.745192308
si:dkey-238o13.4	0.720588235	1.219512195
alg8	0.720588235	1.369565217
si:dkey-195m11.8	0.720338983	0.949367089
capn9	0.72027972	1.033755274
serpinf2a	0.720207254	1.089473684
cyp2ad6	0.72	1.263636364
gfra4a	0.72	0.64
mcm5	0.72	0.800395257
disc1	0.72	0.9
f5	0.72	1
tmem168b	0.72	1.166666667
sts	0.72	1.424242424
zgc:153119	0.72	1.642857143
si:dkey-220o10.1	0.72	2.125
srm	0.719827586	1.308823529
si:ch211-43f4.1	0.719806763	1.0234375
fnbp1a	0.719512195	0.630434783
si:ch211-106m9.1	0.719512195	1.484848485
igsf9a	0.719512195	1.536585366
tspan14	0.719457014	2.039215686
myo1b	0.719211823	1.042471042
stoml3b	0.719167905	1.124752475

plcb3	0.71875	1.03030303
neurl1b	0.71875	0.728070175
wasf3a	0.71875	0.736842105
zgc:173443	0.71875	0.96875
lactbl1b	0.71875	1
gnpat	0.71875	1.23255814
uck1	0.71875	1.928571429
lrrc14b	0.71875	2.277777778
col17a1a	0.718660969	0.979012346
hpda	0.718631179	0.776632302
si:ch211-113d11.5	0.718309859	1.076923077
si:ch73-37h15.2	0.718309859	1.607142857
col6a4a	0.718137255	0.942446043
larp4ab	0.718061674	0.75
mylka_1	0.717948718	0.95505618
hoxa13a	0.717948718	1.25
zgc:193801	0.717948718	1.333333333
p2rx2	0.717948718	1.535714286
msrb2	0.717757009	0.975982533
nxnl1	0.717741935	0.827868852
hlfb	0.717647059	1.036363636
acsl1a	0.717647059	1.037878788
tbx2a	0.717514124	1.22
ltbp1_2	0.717391304	0.827586207
caspa	0.717391304	0.988764045
cables2b	0.717391304	1.035714286
dnttip2	0.717391304	1.371069182
msantd1	0.717391304	1.44
asb2a.1_1	0.716981132	0.808510638
pou2f3	0.716981132	0.945945946
pim2	0.716981132	0.979674797
pop1	0.716981132	1
slc48a1a	0.716981132	1.38
dhrs11a	0.716981132	1.446153846
neurl2	0.716981132	1.882352941
ugt5b3	0.716666667	1.494845361
cnep1r1	0.716535433	1.801104972
hhipl1	0.71641791	1.106382979
cyb5b	0.716346154	1.470198675
vamp5	0.716262976	1.587155963
serpinb1	0.716216216	0.693045564
zgc:66474	0.716216216	0.913793103
ppp3r1a	0.716174975	1.787815126
slc37a4b_1	0.716157205	0.923076923
arrb1	0.716049383	0.693877551
cxcl12a	0.715873016	1.619047619
mmrn2a	0.715789474	1.196721311
mphosph10	0.71563981	1.296774194
pls1	0.715447154	1.107526882
adam17b	0.715447154	1.362318841
CABZ01044277.1	0.715365239	2.143646409
olfml2a	0.715328467	1.5
tulp1a	0.714975845	1.03875969
syngr1a	0.714759536	1.275773196
selenbp1	0.71459695	0.733532934
zgc:194990	0.714285714	0.377358491
KCNJ10 (1 of many)_1	0.714285714	0.392857143
si:dkey-15h19.2	0.714285714	0.52
slc4a5	0.714285714	0.625
si:rp71-62i8.2	0.714285714	0.647058824
foxa2	0.714285714	0.655172414

cx39.4	0.714285714	0.7
olfm2b	0.714285714	0.709677419
EGFR (1 of many)	0.714285714	0.821428571
chst15	0.714285714	0.833333333
kcnq5b	0.714285714	0.866666667
si:dkey-45k15.3	0.714285714	0.866666667
coro2bb	0.714285714	0.928571429
avil	0.714285714	0.9375
hells	0.714285714	0.955555556
rgs9bp	0.714285714	0.96
rgs9bp	0.714285714	0.96
tmem45a	0.714285714	0.969387755
si:ch211-207i1.2	0.714285714	1
si:dkey-93m18.3	0.714285714	1
st6galnac3	0.714285714	1
itpr2	0.714285714	1.03125
mtus1a	0.714285714	1.035714286
rapgef3	0.714285714	1.041666667
slc6a2	0.714285714	1.052631579
STX19	0.714285714	1.08
cenpv	0.714285714	1.117647059
fgf2	0.714285714	1.135135135
trim25l	0.714285714	1.2
OAF (1 of many)	0.714285714	1.2
si:dkey-7i4.13	0.714285714	1.210526316
CU984579.1	0.714285714	1.233333333
pcdh1g1	0.714285714	1.266666667
fam212aa	0.714285714	1.280487805
B3GNT3 (1 of many)_3	0.714285714	1.307692308
map3k20	0.714285714	1.30952381
man1b1b	0.714285714	1.384615385
hdr	0.714285714	1.40625
zgc:153039	0.714285714	1.571428571
tnni1b	0.714285714	1.597826087
si:dkey-7i4.11	0.714285714	1.6
acot9.2	0.714285714	1.689655172
uevld	0.714285714	1.705882353
CLDN22	0.714285714	1.75
acsl4b	0.714285714	1.770833333
ttn.2	0.714285714	1.775510204
calcrla	0.714285714	1.816666667
fabp10b	0.714285714	1.888888889
CABZ01050663.1	0.714285714	1.958333333
FO704584.2	0.714285714	2.409090909
ephx2	0.713567839	0.775641026
cahz	0.713559322	0.929118774
cyp2x7	0.713414634	2.032967033
unc45b	0.713286713	1.161904762
gnpnat1	0.713286713	2.53164557
zgc:123010	0.713235294	0.902439024
sc5d	0.712962963	1.303797468
sema4ab	0.712871287	1.290322581
si:ch73-138e16.7	0.712765957	1.84
hdac8_2	0.71257485	0.9609375
si:dkey-4p15.5	0.712365591	1.277419355
slc25a1b	0.712351946	1.014851485
khk	0.712328767	1.048780488
emg1	0.712328767	1.525773196
rltgr	0.712244898	0.757062147
RNF182 (1 of many)_2	0.712121212	0.813559322
slc13a1	0.712121212	1.041666667

ppp1r14bb	0.712121212	1.139393939
rassf1	0.712041885	0.826086957
p3h1	0.712041885	1.765957447
orc4	0.711864407	0.853107345
wnt5b	0.711864407	1.204968944
suv420h2	0.711764706	1.860759494
synm	0.711711712	2.209090909
ttc7a	0.711538462	0.71875
itga9	0.711538462	1
g3bp1	0.71124031	0.717105263
cbln13	0.711111111	0.66
zgc:113276	0.711111111	1.142857143
SLC29A2 (1 of many)	0.711111111	1.461538462
si:ch1073-219h3.1	0.711111111	2.346153846
barx1	0.7109375	1.182608696
ttn.1	0.710843373	2.12195122
mpeg1.2	0.710526316	0.903614458
slc7a6	0.710526316	0.923076923
trpm4a	0.710526316	0.926605505
si:dkey-230p4.1	0.710526316	0.96
sdsl	0.710526316	1.133333333
dnajb11	0.710526316	1.285140562
znhit2	0.710526316	1.44
zgc:101016	0.710526316	1.734693878
B3GNT3 (1 of many)_1	0.710526316	1.774193548
mtg1	0.710526316	2.194444444
tram1	0.710382514	1.229965157
trim35-28	0.710344828	2.128205128
prkcz	0.710280374	0.459459459
kita	0.710280374	1.298507463
tmem55a	0.710280374	1.315789474
cyp46a1.3	0.71	2.028571429
faah2a	0.709677419	0.745614035
si:ch211-217n8.5	0.709677419	0.772727273
nt5e	0.709677419	0.864583333
acss1	0.709677419	0.935483871
sfi1	0.709677419	1.35
traip	0.709677419	1.5
mblac1	0.709677419	1.578947368
foxa1	0.709677419	0.393258427
AMDHD1	0.709677419	1.1875
rps8b	0.709677419	1.677419355
galnt7	0.709459459	0.880382775
CABZ01059119.1	0.709401709	1.5
prss35	0.709259259	0.814553991
ahr1b	0.709090909	0.625
TADA1	0.709090909	0.836363636
tprg1	0.709090909	1.570652174
enpp1	0.709090909	0.80952381
crabp1b	0.708920188	0.709497207
fam98b	0.708860759	0.886792453
itpr3	0.708860759	1.414634146
si:ch211-261n11.5	0.708860759	1.727272727
zgc:136872	0.708737864	0.910447761
si:ch1073-406l10.2	0.70846395	1.75210084
gldn	0.708333333	0.552631579
mamdc2b	0.708333333	0.6
tmem196b	0.708333333	0.696969697
nfat5b	0.708333333	1.083333333
angptl5	0.708333333	1.921875
slc7a11	0.708333333	1.923076923

grk1a	0.708333333	0.409090909
ptgs1	0.708333333	0.707692308
map3k15	0.708333333	1.348837209
TST	0.708185053	0.923913043
pag1	0.708133971	0.911111111
pkp1b	0.707964602	0.81372549
si:ch211-161h7.4	0.707964602	1.46875
pnpla3	0.707774799	1.082317073
mgea5l	0.707692308	0.639534884
SYCE3	0.707692308	0.833333333
abcb6a	0.707692308	1.27027027
ugt5c3	0.707692308	1.795454545
slco1d1	0.707616708	0.460606061
erlin2_2	0.707602339	1.863636364
lpin1	0.707530648	1.748768473
rorcb	0.707317073	0.939393939
rwdd3	0.707317073	1.2
si:dkey-238k10.1	0.707317073	1.289473684
esyt1a	0.707317073	1.428571429
sema4e	0.707317073	1.028571429
cplx2	0.707165109	1.144508671
si:ch211-133l5.7	0.707142857	1.732758621
si:ch211-207l3.2	0.706896552	0.74
grb10a	0.706896552	1.105263158
kcnb2	0.706521739	0.714285714
SERPINA10	0.705882353	0.363636364
kcnab1a	0.705882353	0.684210526
klb	0.705882353	0.736842105
ogg1	0.705882353	0.761904762
si:ch73-252i11.1	0.705882353	0.8125
atp8a1_2	0.705882353	0.909090909
si:dkey-163m14.7	0.705882353	1.083333333
plekhg4	0.705882353	1.106382979
arhgap24	0.705882353	1.183673469
si:ch73-334d15.4	0.705882353	1.3
zdhhc23b	0.705882353	1.463414634
fam132a	0.705882353	1.507692308
cplx3a	0.705882353	1.512820513
aclyb	0.705882353	1.571428571
si:ch211-76l23.7	0.705882353	1.6
zgc:174895	0.705882353	1.657142857
pdk4	0.705882353	1.702970297
obscnb	0.705882353	1.705882353
lig4	0.705882353	1.739130435
arf4b	0.705882353	2.25
eaf2	0.705882353	1.414634146
si:ch211-66o10.6	0.705607477	1.577777778
elovl7a	0.705501618	1.394495413
frem3	0.705357143	1.142857143
camkvb	0.705263158	0.462962963
star	0.705263158	1.413333333
pole2	0.705128205	0.620689655
LRAT	0.705128205	0.638297872
ppargc1b	0.705035971	1.078431373
grk1b	0.704968944	1.063106796
cplx4a	0.704918033	0.769662921
bcl2l16	0.704918033	1.153846154
si:ch211-223c9.2	0.704918033	1.234042553
med30	0.704697987	1.239316239
kitlga	0.70464135	1.379746835
EPHB3 (1 of many)	0.704545455	1.06557377

htr1d	0.704545455	1.318181818
SAYSD1	0.704545455	2.095238095
efemp2a	0.704347826	1
wdr4	0.704347826	1.223404255
olfml2ba	0.704225352	0.833333333
cmah	0.704225352	0.996124031
aff3_1	0.704225352	1.125
gmnn	0.704225352	2.034482759
sost	0.704225352	1.084848485
vars2	0.703703704	1.0625
mpp5a	0.703703704	1.229166667
zgc:103559	0.703703704	0.863636364
hoxa5a	0.703703704	1.151898734
hpn	0.703703704	1.388888889
slc6a9	0.703601108	0.90821256
mast4	0.703296703	0.733333333
rbm19	0.703125	0.991666667
kin	0.702970297	1.108695652
palm1a	0.70292887	0.941176471
ankrd33ba	0.702702703	0.590909091
snx33	0.702702703	0.757575758
CABZ01056321.1	0.702702703	0.846153846
nupl2	0.702702703	0.923076923
AKAP13 (1 of many)_1	0.702702703	1.043478261
ABHD12B (1 of many)_2	0.702702703	1.127659574
kat2b	0.702702703	1.272727273
palm3	0.702702703	1.476190476
si:dkey-25o16.4	0.702702703	1.756756757
dgat2	0.702544031	1.668831169
slc24a4a	0.702380952	0.491525424
lrit1a	0.702380952	0.626865672
fam117aa	0.702380952	0.885714286
cldn15lb	0.70212766	1
gdpd4a	0.70212766	0.815789474
yars2_1	0.70212766	0.906976744
si:ch211-241e1.5	0.70212766	1.333333333
cfp	0.70212766	1.62962963
abca4b	0.701754386	0.428571429
ube2t	0.701754386	0.928571429
zgc:109744	0.701754386	1.486486486
acaa1	0.701657459	1.03630363
gulp1a	0.701570681	1.345132743
gipc2	0.701550388	1.203947368
c1qtnf9	0.701492537	0.745454545
adma	0.701298701	1.194805195
thoc6	0.701149425	1.173913043
ppardb	0.701149425	1.365217391
lamb4	0.700854701	0.957142857
slc2a2	0.700729927	1.007575758
CABZ01075204.1	0.700729927	1.588235294
si:ch211-105j21.9	0.700680272	2.244186047
fndc7rs4	0.700636943	1.401960784
SLC46A3 (1 of many)	0.700460829	1.7421875
ubash3ba	0.7	0.761904762
thsd1	0.7	0.826086957
sigirr	0.7	0.945945946
usp42	0.7	0.986111111
hoxc6b	0.7	1
tmc4	0.7	1.038461538
card11	0.7	1.05
zgc:171470	0.7	1.15

agmat	0.7	1.181818182
BX957278.1	0.7	1.25
BX547934.1	0.7	1.298850575
clk4b	0.7	1.358974359
kctd2	0.7	2.210526316
zgc:109949	0.7	0.590909091
si:dkey-88e18.6	0.7	0.647058824
csrnp2	0.7	0.652173913
kctd4	0.7	0.691729323
mfsd4a	0.7	0.913043478
dvl1a	0.7	1.045454545
zgc:153219	0.7	1.076923077
vdra	0.7	1.083333333
arid3c	0.7	1.157894737
pth1ra	0.7	1.2
pdxka	0.7	1.212765957
lurap1	0.7	1.217391304
rab11fip5a	0.7	1.333333333
rab11fip1b	0.7	1.394230769
colec11	0.7	1.411764706
sod3b	0.7	1.5
THNSL1	0.7	1.529411765
si:dkey-162h11.2	0.7	1.588235294
CT027832.1	0.7	1.772727273
si:dkeyp-106c3.3	0.7	2
STUM	0.7	2.090909091
igfbp6b	0.7	2.333333333
crfb4	0.7	3.055555556
cilp	0.699494949	1.140495868
dnmt3bb.1	0.699386503	0.865079365
nob1	0.699300699	1.800970874
fn1b	0.699042408	0.71399177
il13ra2	0.699029126	1
si:ch1073-287p18.1	0.698630137	1.76744186
pcdh1g13	0.698412698	0.891891892
si:ch73-14h10.4	0.698412698	1.679245283
st3gal7	0.698412698	2.679245283
pde6c	0.69837587	0.558467742
gabrp	0.698275862	1.029411765
hsd20b2	0.698275862	1.838235294
zgc:165423	0.698159509	1.087173101
sv2c	0.698113208	0.516129032
cyp2aa2	0.698113208	0.707692308
fhl1b	0.698113208	0.941176471
mlphb	0.698113208	1.757575758
ctss2.2	0.697986577	1.042253521
si:dkeyp-73d8.9	0.697841727	0.781818182
bag2	0.697761194	1.122881356
dlgap4b	0.697674419	0.666666667
st8sia4	0.697674419	0.939393939
psmc3ip	0.697674419	1.59375
golt1ba	0.697674419	1.7
rab35b	0.697560976	1.121621622
si:dkey-239b22.2	0.697530864	1.976744186
tnni1d	0.697478992	1.436842105
pcyox1	0.697142857	1.336206897
sfrp1b	0.696969697	1.317460317
hs3st2	0.696969697	0.620689655
efnb3a	0.696969697	0.918918919
c7a	0.696969697	1.02739726
marveld3	0.696969697	1.071428571

antxr2b	0.696969697	1.533333333
def6c	0.696969697	2.1875
stc2a	0.696774194	1.172839506
negr1	0.696666667	1.158783784
rab23	0.696629213	1.093333333
kazna	0.696629213	1.224489796
glra4a	0.696629213	1.3
ldlrap1a	0.696551724	0.923076923
FMC1	0.696428571	0.8125
scpp1	0.696428571	2.074074074
hand2	0.696261682	1.331395349
si:dkey-148a12.2	0.696202532	0.404255319
rai14	0.696202532	1.12244898
rgs20	0.696078431	0.480769231
scrt2	0.695906433	0.884210526
oacyl	0.695652174	0.64516129
si:ch73-380n15.2	0.695652174	0.782608696
chl1a	0.695652174	1.172413793
fes	0.695652174	1.235294118
fam110a	0.695652174	1.357142857
dhrs13b_1	0.695652174	1.392857143
thtpa	0.695652174	1.5
cers3b	0.695652174	1.692307692
si:ch73-184c24.1	0.695652174	0.724637681
klf12b	0.695652174	0.782608696
rnaseh2c	0.695652174	0.96875
hspa13	0.695652174	0.97752809
pde4d	0.695652174	1.113636364
tspan15	0.695652174	1.5
padi2	0.695454545	1.304347826
ralba	0.695402299	1.6
cnn2	0.695340502	1.545454545
npr1b	0.695121951	0.547945205
mtmr10	0.695121951	1.291666667
dio1	0.694736842	0.602564103
haao	0.694690265	1.271676301
chrm3a	0.694444444	1
CHST13	0.694444444	1.035714286
zgc:172121	0.694444444	1.086956522
cenpw	0.694444444	1.113207547
adrb1	0.694444444	1.590909091
zgc:113333	0.694444444	1.830769231
bmp3	0.694323144	0.847682119
rbm28	0.694244604	2.201438849
inadl	0.694117647	0.867924528
nadka_2	0.694117647	0.960526316
KRTCAP3	0.694117647	1.954545455
zc3h15	0.69391635	1.441640379
eif3jb	0.69391635	2
si:dkey-264d12.1	0.693877551	0.807692308
crispld1a	0.693877551	0.85
mastl	0.693877551	0.973684211
si:ch211-195o20.7	0.693877551	1.108108108
alpk1	0.693877551	1.84
sult1st1	0.693661972	1.866242038
capn12	0.693452381	1.487603306
si:ch211-285j16.4	0.693430657	1.285714286
ush1c	0.693333333	0.873015873
ripk2	0.693333333	1.020408163
hoxd11a	0.693333333	1.041666667
gna14	0.693333333	1.833333333

zdhhc23a	0.693333333	2.482758621
ntng2b	0.693181818	0.4
rhov	0.693181818	1.216216216
cdk8	0.692913386	1.075268817
entpd3	0.692893401	1.337962963
mal	0.692592593	0.748618785
si:dkey-222l13.1	0.692307692	1.208333333
map1lc3cl	0.692307692	0.6
map6b	0.692307692	0.705882353
drc3	0.692307692	0.75
si:ch73-361h17.1	0.692307692	0.788888889
fibina	0.692307692	0.809798271
adprhl1	0.692307692	1.19047619
ngfrb	0.692307692	1.375
klhl30	0.692307692	1.461538462
postna	0.692307692	1.592592593
si:rp71-80o10.4	0.692307692	1.833333333
si:ch73-295i22.2	0.692307692	2.4
CABZ01061495.1	0.692307692	2.466666667
coch	0.691891892	1.551401869
mgst1.1	0.691823899	1.347826087
slc9a3r1	0.691666667	0.813131313
mvda	0.691588785	1.393939394
cdc42se1	0.691542289	1.309677419
unga	0.691489362	0.64
tcea3	0.691489362	1.033333333
rab3il1	0.691176471	0.943396226
sema3ab	0.691176471	1.176470588
vgll4l	0.690909091	1.030927835
si:ch73-340n8.4	0.690909091	1.076923077
chst12a	0.690909091	1.416666667
stat6	0.690909091	1.489583333
pik3cg	0.690909091	1.514285714
phka1b	0.690909091	2.625
igf2bp3	0.690839695	0.647058824
si:ch73-234b20.5	0.690751445	1.949771689
MB21D2	0.69047619	0.64516129
ppp1r26	0.69047619	0.897959184
FAM107A (1 of many)_2	0.69047619	0.936507937
MAP3K6	0.69047619	0.942857143
RHOBTB1	0.69047619	0.952380952
alkbh1	0.69047619	1.117647059
col8a2	0.69047619	1.159292035
zgc:77938	0.690346084	1.016216216
trub1	0.690265487	1.405063291
col9a1b	0.690217391	0.567164179
si:ch211-193k8.5	0.690140845	0.783783784
si:dkey-21e2.13	0.690140845	0.975903614
gfra1a	0.690140845	1.2
obsl1b	0.690140845	1.727272727
rlf	0.69	0.789473684
knop1	0.689922481	1.173076923
cideb	0.689839572	1.090909091
nxph2b	0.689655172	0.461538462
zgc:158689	0.689655172	0.675
si:dkey-117a16.2	0.689655172	0.681818182
hoxc6a	0.689655172	0.962616822
si:ch211-199c19.4	0.689655172	0.978723404
fgf18a	0.689655172	1
il34	0.689655172	1.260869565
znf1034	0.689655172	3.5

dkc1	0.689612015	0.96099844
kcnip1b	0.689497717	1.428571429
slc2a15b	0.689189189	1.042328042
fermt1	0.68907563	0.837837838
stra6	0.688995215	0.959537572
si:dkey-13i19.8	0.688845401	1.773700306
sntb2	0.688679245	1.246153846
cyp26c1_2	0.68852459	1.75862069
scpp9	0.688442211	0.160493827
si:dkey-100n23.5	0.688311688	1.5
degs2	0.688212928	0.906779661
thbs2b	0.688172043	0.85483871
asph	0.688057041	1.643835616
UTP14C	0.687804878	1.091549296
suz12a	0.6875	0.731958763
hk2	0.6875	1.53030303
ppp1r35	0.6875	0.454545455
si:dkey-126g1.9	0.6875	0.739130435
pgbd5	0.6875	0.76
arhgap22	0.6875	0.761904762
si:dkey-90m5.4	0.6875	0.813559322
TMPRSS7	0.6875	0.941176471
si:ch211-38m6.6	0.6875	1
si:cabz01087415.1	0.6875	1.096153846
itprip	0.6875	1.107142857
si:ch211-200e2.1	0.6875	1.153846154
tmem121a	0.6875	1.25
znf1112	0.6875	1.5
si:ch211-277g23.5	0.6875	1.571428571
tor4aa_2	0.6875	1.581395349
si:dkey-208m12.2_1	0.6875	1.588235294
fbxl3a	0.687179487	1.137096774
cdh5	0.687022901	2.105263158
si:ch73-264i18.3	0.686956522	2.584615385
nfe2l3	0.686813187	1.084033613
mad2l1	0.686746988	1.010714286
nos1	0.68627451	0.405405405
npr1a	0.68627451	0.933333333
HSPA8 (1 of many)	0.68627451	0.948717949
aspm	0.68627451	1.322033898
si:ch73-103l1.2	0.68627451	3.533333333
chmp5a	0.686131387	1.876923077
igfbp2b	0.685897436	0.870229008
si:ch211-209j12.1	0.685714286	0.625
FQ378018.1	0.685714286	0.692307692
nr3c2	0.685714286	0.727272727
pipox	0.685714286	0.891566265
pde6b	0.685714286	1.086956522
usp46	0.685714286	1.3
znf576.1	0.685714286	1.423076923
prkca	0.685421995	1.088803089
znf711	0.685393258	1.1875
si:dkeyp-89c11.3	0.685393258	1.337078652
tmod2	0.685365854	1.880829016
gk5	0.685314685	0.606060606
si:dkey-234h16.7	0.685185185	0.615384615
SEMA4D	0.685185185	0.823529412
rpap2	0.685185185	1.292682927
srfb	0.684782609	1.078651685
abcc12	0.684782609	1.607843137
tpd52l1	0.684782609	1.78313253

b2ml	0.68469657	2.90078329
zgc:194209	0.684684685	0.710843373
prmt6	0.684684685	1.193877551
myh11a	0.684615385	1.373390558
zbtb8os	0.684587814	1.796178344
hbl1	0.684210526	0.52173913
SLC22A13	0.684210526	0.583333333
hcar1-4	0.684210526	0.714285714
rims4	0.684210526	0.75
s100z	0.684210526	0.885714286
zgc:173624	0.684210526	0.925925926
si:ch211-28p3.3	0.684210526	1
chrng	0.684210526	1
acot11b	0.684210526	1.35483871
si:ch211-134a4.2	0.684210526	1.529411765
stx7l	0.684210526	1.742857143
si:ch211-255i20.3	0.684210526	1.777777778
si:ch211-262i1.3	0.684210526	1.884615385
FUT9 (1 of many)_4	0.684210526	2.4
srek1ip1	0.684210526	1.45
tnfaip2a	0.684210526	1.847058824
esco2	0.683908046	0.847328244
ccl25b	0.68372093	1.386292835
plscr3a	0.683673469	1.16
zgc:162193	0.683673469	1.2
aox5	0.683673469	1.626865672
tm4sf4	0.683544304	0.63908046
nudt7	0.683544304	1.477272727
RGS9BP	0.683467742	1.721362229
npm1b	0.683453237	1.436781609
apbb1	0.683257919	0.721804511
slc25a48	0.683168317	1.320512821
ace2	0.683060109	1.923076923
macf1b	0.682926829	0.6
serping1	0.682926829	0.960784314
si:dkey-101k6.5	0.682926829	1.064516129
si:dkey-7n6.2	0.682926829	1.154929577
ace	0.682795699	0.795454545
tpd52	0.682692308	1.908333333
slc38a5a	0.682634731	1.806451613
lmnb2	0.682580645	1.272076372
ovol1	0.682242991	1.097560976
glrx2	0.682242991	1.694444444
inab	0.682151589	0.73800738
mphosph6	0.682080925	1.26056338
lctla	0.681818182	1.155172414
fut9b	0.681818182	2.159090909
kcnj8	0.681818182	0.4
dok6	0.681818182	0.6875
ccdc173	0.681818182	0.888888889
scarf1	0.681818182	1
zgc:123295	0.681818182	1
si:ch1073-165f9.2	0.681818182	1.035714286
p4ha1b	0.681818182	1.12962963
aatka	0.681818182	1.314814815
iffo2b	0.681818182	1.428571429
card14	0.681818182	1.9
si:dkey-250k10.1	0.681818182	2.181818182
sigmar1	0.681388013	1.095435685
arhgap27l	0.681318681	1.355932203
ngb	0.681081081	1.701923077

si:dkeyp-13a3.10	0.681034483	1.430379747
AMIGO3 (1 of many)	0.680851064	1.363636364
MYADML2	0.680851064	1.391304348
zgc:153901	0.680851064	1.5
zgc:174863	0.680851064	1.923913043
baz1b	0.680701754	0.753012048
si:dkey-24l11.2_1	0.680672269	0.917647059
kcnj11	0.680555556	1.49
frzb	0.680272109	0.855855856
BX537263.2	0.68	0.889201878
guca1g	0.68	0.90625
ngfra	0.68	0.980769231
PLEKHG5 (1 of many)	0.68	1
il20ra	0.68	1.05
cntf	0.68	1.095238095
LPL (1 of many)	0.68	1.235294118
mfng	0.68	1.25
CABZ01020840.1	0.68	1.285714286
trim33l	0.68	1.352941176
paqr8	0.68	1.692307692
wrn	0.68	1.928571429
si:dkey-7i4.21	0.68	2.666666667
smn1	0.679775281	0.778443114
glsb	0.679775281	1.97
litaf	0.67961165	1.252427184
angptl2b	0.679425837	1.078014184
smdt1a	0.679287305	1.112709832
atp8b1	0.679012346	1.175438596
polr3gla	0.679012346	1.591836735
zgc:92818	0.67877095	1.176271186
clic6	0.678571429	0.404761905
trpa1b	0.678571429	0.52
espn	0.678571429	0.625
a1cf	0.678571429	0.76419214
nfe2	0.678571429	0.882352941
mcf2l2	0.678571429	0.941176471
si:ch1073-296i8.2	0.678571429	0.961538462
isy1	0.678571429	0.968
fynrk	0.678571429	1.297297297
si:dkey-19b23.7	0.678571429	1.333333333
cdkn1cb	0.678571429	1.344827586
lmod1a	0.678571429	1.45
ugt5d1	0.678571429	1.558823529
spaca4l	0.678416821	1.198630137
hspb8	0.678321678	1.762886598
pgap2	0.67816092	2.081632653
si:ch211-282b22.1	0.678030303	1.330143541
gcgra	0.677966102	0.947368421
col19a1_1	0.677966102	1.083333333
tmprss2	0.677966102	1.13559322
si:dkey-52l18.4	0.677966102	1.352941176
hoxb6b	0.67768595	1.224719101
si:cabz01007802.1	0.677419355	1.2
cxcr3.2	0.677419355	1.233333333
CABZ01054962.1	0.677419355	1.5
tmem163a	0.677419355	0.896551724
stxbp1b	0.677233429	1.063218391
mkrn4	0.677083333	1.076923077
actr2b	0.676923077	1.568627451
lhfpl4b	0.676470588	0.770833333
ralgps1	0.676470588	0.826086957

si:ch211-57n23.1	0.676470588	1.25
si:dkey-187k19.2	0.676470588	1.409090909
CR759791.1	0.676470588	1.535714286
lgals1l1	0.676432292	1.419117647
cxcl12b	0.676348548	1.351351351
b3gat1a	0.676056338	0.552631579
fscn2a	0.676056338	0.705882353
dao.1	0.676056338	1.133333333
exoc3l2b	0.675675676	0.666666667
dnmt3bb.2	0.675675676	1.111111111
GNPAT (1 of many)	0.675675676	1.368421053
si:cabz01071909.2	0.675675676	1.421052632
surf6	0.675675676	1.506097561
crlf1b	0.675675676	1.578947368
si:dkey-4e7.3	0.675531915	0.854545455
hdhd2	0.675438596	1.677966102
prex2	0.675324675	0.907407407
si:ch211-59o9.10	0.675213675	0.905263158
AEBP1 (1 of many)	0.675213675	1.012711864
pcp4l1	0.675182482	1.20855615
CABZ01076737.1	0.675	1
si:ch211-163l21.8	0.675	1
prom1b	0.675	1.027210884
zc3h3	0.675	1.08
tmem30c	0.675	1.157894737
tfeb	0.675	1.166666667
nudt14	0.675	1.232876712
ramp2	0.674603175	1.302083333
zgc:162144	0.674418605	0.488372093
si:cabz01100188.1	0.674418605	0.571428571
arhgap11a_1	0.674418605	1.038461538
SLC16A3 (1 of many)	0.674418605	1.043478261
si:ch211-37e10.2	0.674418605	1.142857143
mafa	0.674157303	0.674698795
acad9	0.674157303	1.487341772
hsd17b14	0.674157303	1.653061224
sfrp1a	0.674008811	0.81773399
plag1	0.673913043	1.037037037
myo5ab_1	0.673913043	1.09375
slc30a10	0.673913043	1.24
cep104	0.673913043	2.142857143
nefmb	0.673913043	0.445578231
ipmkb	0.673684211	1.277777778
cdh15	0.673611111	1.450549451
si:dkey-203a12.9	0.67357513	0.592057762
pdgfrl	0.673515982	0.818615752
ecrg4b	0.673469388	0.876606684
slc10a1	0.673469388	0.537313433
shtn1	0.673469388	1.206896552
gata4	0.673469388	1.413793103
fam114a1	0.673267327	1.119402985
grik1a	0.673076923	0.58974359
hmgcra	0.673076923	0.75
lingo4b	0.673076923	1.303030303
adgrg1_1	0.673076923	1.315789474
ucmab	0.672770701	0.774647887
hapln3	0.672727273	0.951612903
snx22	0.672727273	0.979591837
zgc:194887	0.672727273	1.041666667
ZC3H12A	0.672727273	1.84
gorab	0.672727273	2.24137931

scn2b	0.67264574	1.729411765
lrrc8c_1	0.67251462	1.854166667
gng12a	0.67219917	1.061111111
si:dkey-14d8.7	0.672131148	0.848484848
tesca	0.672131148	1.12195122
sat1b	0.672	1.679487179
cpxm1a	0.671875	0.797619048
cab39l1	0.671875	1.781914894
macc1	0.671875	1.4375
klf5a	0.671755725	0.652173913
fam173b	0.671755725	1.07079646
svopl	0.671641791	0.910447761
sult2st3	0.671428571	2.741935484
kti12	0.671052632	1.923076923
TM6SF2 (1 of many)	0.670886076	0.670588235
acvr2ab	0.670731707	1.252873563
si:ch73-23l24.1	0.67036626	0.513957307
cplx3b	0.67032967	0.590163934
eng2b	0.670212766	1.322580645
ca4a	0.67	0.352941176
gtf3c6	0.669811321	1.322916667
si:dkeyp-1h4.6	0.669724771	1.974025974
mier3b	0.669642857	2.127272727
nucb2b	0.669172932	1.611111111
cfd	0.669166667	0.692877493
clic3	0.668874172	1.765151515
ednraa	0.668711656	0.903225806
rcc1	0.668508287	0.669172932
tet2	0.668316832	1.196261682
kmo	0.668202765	0.777777778
si:dkey-204f11.64	0.668141593	1.9921875
map1aa	0.667553191	0.90052356
kcna4	0.666666667	0.666666667
si:dkey-71b5.2	0.666666667	0.711111111
rd3	0.666666667	0.733333333
sh3pxd2aa	0.666666667	0.833333333
REPS2	0.666666667	0.901960784
f8	0.666666667	0.928571429
wnk4b	0.666666667	1.03125
zgc:77158	0.666666667	1.043478261
tmprss3a	0.666666667	1.16
mettl11b	0.666666667	1.215686275
nsl1	0.666666667	1.303030303
cav2	0.666666667	1.333333333
scara3	0.666666667	1.375
sypl2b	0.666666667	1.385714286
TMEM116	0.666666667	1.5
si:dkey-9i23.15	0.666666667	1.529411765
ftr14	0.666666667	1.538461538
si:cabz01080295.1	0.666666667	1.555555556
tbc1d19	0.666666667	1.608695652
si:dkey-193c22.2	0.666666667	1.625
kcnk18	0.666666667	1.777777778
elmo3	0.666666667	1.8
si:ch211-127b16.4	0.666666667	1.928571429
faah2b	0.666666667	0.318181818
rlbp1b	0.666666667	0.469565217
si:dkey-156n14.3_1	0.666666667	0.566666667
cntn4_1	0.666666667	0.578947368
gpr88	0.666666667	0.666666667
si:dkey-89b17.7	0.666666667	0.675675676

mctp2b	0.666666667	0.68
slc5a8	0.666666667	0.684210526
fam129ab	0.666666667	0.6875
gcgrb	0.666666667	0.692307692
rad54b	0.666666667	0.736842105
tp73	0.666666667	0.75
tbata	0.666666667	0.80952381
tnfsf10l3	0.666666667	0.84
dfnb31a	0.666666667	0.909090909
adam12	0.666666667	0.916666667
znf750	0.666666667	0.916666667
si:dkeyp-73c8.3	0.666666667	1
si:dkeyp-82a1.1	0.666666667	1
agrp2	0.666666667	1.02688172
TMEM42 (1 of many)_2	0.666666667	1.037037037
adra1d	0.666666667	1.05
nt5c1aa	0.666666667	1.057142857
si:dkey-283b1.7	0.666666667	1.076923077
wnt3a	0.666666667	1.1
dock5	0.666666667	1.16
slc8a1a	0.666666667	1.166666667
sh3d19	0.666666667	1.175438596
map1lc3c	0.666666667	1.19047619
esr2b	0.666666667	1.2
zgc:136870	0.666666667	1.285714286
F2RL3	0.666666667	1.3
SEMA4F	0.666666667	1.307692308
bbox1	0.666666667	1.31
smyd3	0.666666667	1.325581395
dbh	0.666666667	1.333333333
si:dkey-166f24.2	0.666666667	1.333333333
mxra5b	0.666666667	1.352941176
chgb	0.666666667	1.365384615
slc2a15a	0.666666667	1.369047619
nkx2.7	0.666666667	1.5
phf20a	0.666666667	1.555555556
sowahaa	0.666666667	1.590909091
nrn1b	0.666666667	1.615384615
ccdc40	0.666666667	1.6875
PIK3R6 (1 of many)	0.666666667	1.692307692
tnfrsf1b	0.666666667	1.7
cysltr1	0.666666667	1.727272727
rfxank	0.666666667	1.727272727
pla1a	0.666666667	1.829268293
znf644a	0.666666667	1.842857143
JAG2 (1 of many)	0.666666667	1.846153846
p2rx7	0.666666667	1.933333333
prob1	0.666666667	2.142857143
si:rp71-68n21.12	0.666666667	2.166666667
si:dkey-84h14.2	0.666666667	2.666666667
slc14a2	0.666666667	3.6
si:ch211-160f23.7	0.666666667	1.5
pnp4b	0.665625	2.201219512
plekhf2	0.665048544	0.919786096
gcshb	0.664756447	1.216890595
dpys	0.664670659	0.920634921
si:ch73-185c24.2	0.664122137	0.806451613
si:dkeyp-74b4.3	0.663865546	1.921052632
dnajb1a	0.663551402	1.230769231
dnajb14	0.663551402	2.075471698
cyp8b1	0.663461538	0.714765101

pkhd1l1	0.663366337	1.097560976
srd5a2a	0.663101604	1.434210526
lcat	0.662983425	1.44
hmox1a	0.662962963	1.22962963
fbxo40.2	0.662921348	1.607142857
st6galnac1.1	0.662921348	1.637931034
adamtsl7	0.662790698	0.734939759
opcml	0.662790698	1.024390244
slc15a1b	0.662721893	0.622222222
asb10	0.662337662	0.632075472
wu:fb44b02	0.661971831	1.342105263
bco1	0.661904762	1.336065574
bin2a	0.661870504	1.196363636
dixdc1b	0.661764706	0.977777778
lad1	0.661764706	0.982142857
SLC16A6 (1 of many)	0.661764706	1.268292683
zgc:92137	0.661654135	1.666666667
mmp30	0.661616162	1.633495146
cpt1cb	0.661538462	1.324324324
si:dkeyp-89c11.1	0.661417323	1.263157895
erv	0.661290323	1.188405797
si:ch211-66k16.27	0.661290323	1.71875
chia.2	0.661097852	0.990599295
hspb15	0.661016949	1.348484848
bmp4	0.660377358	1.875
thbs3b	0.660377358	0.976190476
zgc:172079_2	0.66	1.456790123
si:ch211-229g11.6	0.66	1.660377358
cd59	0.659942363	0.978947368
fat2	0.659574468	1
fam83d	0.659574468	1.037037037
thbd	0.659574468	3.785714286
igsf8	0.65942029	1.026666667
dpf1	0.659090909	0.637681159
si:ch211-136a13.2	0.659090909	0.675
snx8a	0.659090909	0.805555556
man2a1	0.659090909	1.2
slc22a16	0.659090909	1.284313725
thy1	0.658878505	1.085427136
vegfab	0.658823529	1.245283019
lrrc16a	0.658823529	1.547619048
gpn3	0.658682635	1.007633588
enpp6	0.658536585	0.435897436
si:ch211-267e7.3	0.658536585	0.909090909
gb:eh507706	0.658536585	1.192982456
tmem161a	0.658536585	1.859813084
si:ch73-306e8.2	0.658227848	0.957446809
pon3.2_1	0.658119658	1.220183486
si:dkey-31n5.4	0.657894737	0.75
fndc4a	0.657894737	1.032258065
CABZ01090021.1	0.657894737	1.225806452
rassf9	0.657894737	1.705882353
eng2a	0.657894737	1.028169014
cyp2k19	0.657718121	1.380434783
hirip3	0.657657658	0.986577181
stat5b	0.657657658	1.849056604
taok2b	0.657534247	0.861111111
cadm2a	0.657303371	0.93902439
urad	0.657142857	0.589041096
slc7a4	0.657142857	1
mylk3	0.657142857	1.037037037

hs1bp3	0.657142857	1.4
uhmk1	0.657142857	1.545454545
si:ch211-214p16.3	0.657142857	1.5625
otulina_2	0.657142857	1.976190476
si:ch211-217g15.3	0.657142857	2
fam129bb	0.656862745	1.403225806
zgc:162780	0.656716418	1.02247191
osgn1	0.656603774	1.027027027
ghrb	0.65625	0.961038961
AC024175.24	0.65625	0.988235294
zgc:158328	0.65625	1.195121951
COL21A1	0.65625	1.25
tatdn3	0.65625	1.375
tatdn3	0.65625	1.375
slc22a7b.1	0.65625	1.666666667
si:ch211-42i9.5	0.65625	1.763157895
nrip1a	0.656050955	1.255813953
irf9	0.655913978	1.157142857
sptb	0.655913978	1.180555556
gabra6a	0.655737705	0.555555556
fam131bb	0.655737705	0.842105263
pdzd3a	0.655737705	1
mxd4	0.655172414	1.46
brf2	0.655172414	0.444444444
mfsd2aa	0.655172414	0.692307692
ugt5a1	0.655172414	0.96875
frs2a	0.655172414	1.259259259
caln2	0.655172414	1.555555556
iqck	0.655172414	1.8125
crispld1b	0.654929577	1.091836735
camk2d1	0.654822335	1.516129032
stat1a	0.654676259	1.993333333
plppr5a	0.654545455	1.242424242
eps8l1	0.654545455	1.379310345
trdn	0.654501217	1.684824903
arhgef25b	0.654320988	0.684931507
zgc:113307	0.653992395	0.84
plekhd1	0.653846154	0.653846154
si:ch211-102c2.4	0.653846154	0.666666667
PRKCA (1 of many)	0.653846154	1
ulk4_1	0.653846154	1
ugt5g1	0.653846154	1.131578947
si:dkey-202l16.5	0.653846154	1.347826087
crfb16	0.653846154	1.461538462
CLRN3	0.653846154	1.470588235
si:dkey-79d12.4	0.653846154	1.909090909
lyrm9	0.653846154	2.272727273
wscd2	0.653333333	1.103448276
si:ch211-86h15.1	0.653333333	4.217391304
mapre1a	0.653266332	1.116438356
alpl	0.653225806	1.243243243
mmd	0.653061224	1.489208633
zgc:110782	0.653061224	0.606060606
dkk2	0.653061224	1.34375
zgc:153184	0.652777778	0.942857143
ppie	0.652542373	0.944444444
si:ch1073-82l19.1	0.652173913	0.6
si:dkey-181d19.6	0.652173913	0.608695652
spag9a	0.652173913	0.731707317
CABZ01054965.1	0.652173913	1.222222222
fgd6	0.652173913	1.315789474

si:ch211-212d10.1	0.652173913	1.388888889
trmt2b	0.652173913	1.473684211
ifi35	0.652173913	1.47826087
mki67	0.652061856	1.509803922
hbae1	0.651718984	0.715447154
hhipl2	0.651515152	1.486486486
mycla	0.651376147	0.43220339
tbcelb	0.651376147	1.183333333
dnajc5b	0.651162791	1.090909091
kcnip3b	0.651162791	1.129032258
lzts1	0.651162791	1.294117647
ikbip	0.651162791	1.52
agxta	0.650909091	0.857894737
si:dkey-56f14.4	0.650793651	1.350649351
LEPROT	0.650684932	1.568421053
tnfaip6	0.65060241	0.903614458
rab25b	0.65060241	1.148148148
mylk4b	0.650485437	2.574468085
adipoqb	0.65	0.630434783
avpr1ab	0.65	0.923076923
klhl38a	0.65	0.923076923
si:ch211-241f5.3	0.65	1.090909091
micall2b	0.65	1.153846154
cobll1a	0.65	1.166666667
HPX (1 of many)	0.65	1.214285714
lsm11	0.65	1.333333333
proser3	0.65	1.416666667
egln2	0.65	1.571428571
ZNF513 (1 of many)	0.65	1.588235294
srfbp1	0.65	2.065789474
si:ch211-159i8.4	0.649484536	1.18
tfr1b	0.649415693	1.164305949
si:dkey-283b1.6	0.649350649	1.75862069
loxl1	0.649006623	1.387755102
si:dkey-113d16.9	0.648888889	1.036809816
ddx26b	0.648648649	0.88372093
tmem150ab	0.648648649	0.897435897
moxd1	0.648648649	1.16
ccdc125	0.648648649	1.470588235
itpk1a	0.648514851	1.2
scarb2	0.648373984	1.068452381
gss	0.648351648	0.93442623
si:ch211-252f13.5	0.648148148	0.880952381
mhc1uma	0.648148148	1.75
hgh1	0.648	0.715447154
bckdhbl	0.647727273	0.729281768
hif1aa	0.647727273	0.838235294
pparg	0.647727273	1.027027027
si:dkeyp-94b4.1	0.647577093	1.210144928
serpinh1a	0.647249191	1.247572816
cnga1	0.647058824	0.566666667
zmynd10	0.647058824	0.733333333
zgc:171844	0.647058824	0.916666667
reep1	0.647058824	0.933333333
si:dkey-20d21.12	0.647058824	1.070422535
tgm5l	0.647058824	1.176470588
si:ch73-302a4.4	0.647058824	1.230769231
ERGIC2 (1 of many)	0.647058824	1.615384615
ppl	0.646868948	1.713956171
rgs8	0.646778043	1.511551155
slc37a4a	0.646723647	1.050445104

fscn1b	0.646464646	1.6
slco2b1	0.646153846	1.064814815
itgb1b.2	0.646067416	0.936708861
si:ch211-226f24.4	0.645833333	0.730769231
tnfaip3	0.645833333	1.5
tmem17	0.645833333	1.810810811
gpc1a	0.645833333	2.391304348
muc13b	0.645714286	1.477777778
fndc7a	0.64556962	1.326923077
elovl4b	0.645514223	0.897196262
tfpia	0.64516129	1.294117647
nkain2	0.64516129	0.303571429
si:ch73-66d24.2	0.64516129	0.794871795
si:dkey-19b23.13	0.64516129	0.92
lmo7b	0.64516129	0.952380952
SLC35G1	0.64516129	1.166666667
micall2a	0.64516129	1.333333333
cbln2a	0.64516129	1.875
tank_2	0.64516129	2.055555556
rce1a	0.64516129	2.363636364
txlnba	0.644927536	1.54491018
tagln3b	0.644897959	0.841059603
abca12	0.644808743	1.084337349
SI	0.644444444	0.697916667
emilin3a	0.644444444	0.689655172
hepacam2	0.644444444	1.157894737
ugt5e1	0.644444444	1.32
sall4	0.644067797	0.764705882
ankrd1b	0.643973214	2.06175772
zmp:0000000758	0.643835616	0.826666667
rbp5	0.643776824	0.747989276
zgc:92658	0.643678161	1.933333333
rnaset2	0.643564356	3.95
st6galnac	0.64321608	1.244604317
tac1	0.643171806	0.944827586
antxr1b	0.642857143	0.458333333
nkx2.1	0.642857143	0.75862069
rprmb	0.642857143	1.014925373
ubtd1b	0.642857143	1.129032258
CU571081.1	0.642857143	1.146341463
anks3	0.642857143	1.3
adprm	0.642857143	1.380952381
akap12b	0.642857143	1.64028777
si:ch73-340m8.2	0.642857143	1.714285714
slc25a17	0.642857143	1.814814815
ugt8	0.642857143	1.855263158
si:dkey-21c1.4	0.642857143	2.3
si:ch211-286h12.6	0.642857143	3.357142857
dpm2	0.642384106	1.910526316
sec14l8	0.642335766	1.027027027
ccdc136a	0.642201835	0.786666667
hsd11b1la	0.642105263	0.904191617
si:ch211-114n24.7	0.642045455	1.295238095
si:dkey-189n19.3	0.641975309	0.75
sc:d189	0.641975309	0.85915493
slc39a14	0.641791045	1.333333333
epas1a	0.641686183	1.286821705
fam160a1a	0.641509434	1.35483871
ergic1	0.641509434	1.472727273
si:dkey-117m1.4	0.641304348	0.660377358
serpinh1b	0.641304348	1.031496063

xylb	0.641025641	1
golim4b	0.641025641	1.157894737
pkp1a	0.641025641	1.8
zgc:113442	0.641025641	2.642857143
slc5a9	0.640625	0.452830189
zgc:162331	0.64	0.41509434
slc13a3	0.64	0.596153846
CU855947.1	0.64	0.615384615
nfatc4	0.64	0.75
IYD	0.64	0.85106383
sod3a	0.64	1.034482759
atp10a	0.64	1.066666667
mettl8	0.64	1.1
HS3ST3B1	0.64	1.105263158
zgc:153631	0.64	1.117647059
TMEM255B	0.64	1.4
si:ch211-238e22.1	0.64	1.507246377
si:dkey-27i16.2	0.64	1.583333333
znf1141	0.64	1.666666667
si:dkey-208m12.2_2	0.64	2.296296296
paqr5b	0.64	3.25
tlcd1	0.639705882	1.467889908
sgk2a	0.639534884	1
cnrip1b	0.639344262	0.6
sncga	0.639344262	0.651515152
SLC6A13	0.639344262	1.012658228
zgc:101040	0.639344262	1.025316456
ptpn21	0.639344262	1.375
rbp7a	0.639175258	1.784810127
CABZ01102109.1	0.638888889	0.733333333
HIP1R (1 of many)	0.638888889	1.347826087
ankk1	0.638888889	2.25
ugt5b2_3	0.638554217	1.534482759
vstm4b	0.638297872	0.619047619
mmrn2b	0.638297872	1
hpdl	0.638297872	1.12
slc35d1a	0.638297872	1.338709677
copz2	0.638095238	1.233333333
slc13a4	0.638059701	0.995983936
cgnb	0.637931034	1.055555556
rhoj	0.637931034	1.348837209
fer	0.637931034	2.130434783
hhla2b.2	0.637837838	2
cyp2p6	0.637795276	1.289719626
ppp1r3b	0.637681159	1.585365854
ihha_2	0.6375	0.794871795
tnfaip8l2b	0.637362637	1.772727273
laptm4b	0.637295082	1.828358209
PXN	0.637254902	1.183098592
map2k4a	0.637096774	1.225806452
mapk13	0.637037037	1.5
vil1	0.637007078	0.597449909
VAMP1 (1 of many)	0.6367713	1.876106195
slc46a3	0.636363636	0.928571429
tmem79a	0.636363636	1
prss60.2	0.636363636	1.075630252
cyp2k21	0.636363636	1.181818182
slc2a13b	0.636363636	1.24137931
AL954146.1	0.636363636	1.333333333
si:dkey-240a24.1	0.636363636	1.458333333
lysmd1	0.636363636	1.485714286

bin3	0.636363636	2.680851064
ptdss1b	0.636363636	0.875
si:dkey-188i13.10	0.636363636	0.958333333
si:ch211-168b3.2	0.636363636	1.181818182
si:dkey-56e3.2	0.636363636	1.428571429
znf365	0.636363636	1.5
mctp1b	0.636363636	1.909090909
ak3	0.636132316	0.96013289
slc25a3a	0.635883905	0.676
slc25a10	0.635761589	0.935691318
tmlhe	0.635514019	1.245283019
tdp1	0.635135135	1.090909091
slc5a6a	0.634920635	0.942857143
si:dkey-71h2.2	0.634920635	1.657894737
si:ch211-106h4.9	0.634615385	0.806451613
PRIMA1	0.634615385	0.939393939
fads2	0.634408602	0.731182796
wdr20b	0.634146341	0.868421053
scube1	0.634146341	1.238095238
si:ch73-43g23.1	0.634146341	1.304347826
sync	0.634146341	1.526315789
FP085398.1	0.634146341	1.636363636
ccdc43	0.633663366	1.449275362
zgc:64106	0.633561644	0.87012987
slc13a5a	0.633333333	0.8
si:dkey-227h16.2	0.633333333	0.923076923
zgc:162160	0.633333333	0.947368421
galr1b	0.633333333	0.958333333
pudp	0.633333333	1.019230769
FAM46A (1 of many)	0.633333333	1.1875
pcdh1g22	0.633333333	1.266666667
mb	0.633270321	0.623471883
hoxa11b	0.632911392	0.680555556
si:ch73-204p21.2	0.632850242	1.357615894
ccdc3b	0.632653061	1.369565217
kif20bb	0.632653061	1.138888889
si:dkey-71d15.2	0.632653061	1.432432432
gins3	0.632352941	0.836065574
cxcl14	0.632231405	1.40247678
tmem176	0.631696429	1.013953488
s1pr3a	0.631578947	0.80952381
mdm1	0.631578947	0.833333333
armc2	0.631578947	0.846153846
PROZ (1 of many)	0.631578947	0.888888889
adamtsl2	0.631578947	1
si:ch211-236d3.3	0.631578947	1
tmtops2a	0.631578947	1.1
stk32a	0.631578947	1.25
spice1	0.631578947	1.675675676
si:dkey-253d23.5	0.631578947	1.727272727
bcam	0.631217839	1.508196721
C2CD4C (1 of many)_2	0.630952381	0.75
fam129ba	0.630952381	0.970588235
timm17b	0.630952381	1.520833333
si:dkeyp-89c11.2	0.630769231	1.148148148
CABZ01057488.2	0.630434783	1.090909091
sfmbt2	0.630434783	1.653846154
gna14a	0.630434783	2.6
rap1gap	0.630136986	2.076923077
birc6-as2	0.62992126	0.731182796
sgcg	0.629807692	1.333333333

phf23a	0.62962963	1.833333333
gabrr3b	0.62962963	0.916666667
tmem123	0.62962963	1.125
zgc:173729	0.62962963	1.15
si:ch73-215f7.1	0.62962963	1.192307692
casp3b	0.62962963	1.344827586
lrrc31	0.62962963	1.642857143
rbm45	0.62962963	1.727272727
arhgef39	0.62962963	2
ston2	0.629441624	1.471153846
FRMD1	0.628865979	1.296875
5S_rRNA_664	0.628787879	2.75
crp1	0.628571429	0.705882353
drd3	0.628571429	0.857142857
plcg2	0.628571429	1.076923077
igsf5a	0.628571429	1.529411765
rorb	0.628378378	1.513157895
abcb5	0.628078818	1.292682927
cntnap1	0.627906977	0.454545455
si:dkey-14f6.1p	0.627906977	1.083333333
spega	0.627906977	1.285714286
RAB7B	0.627906977	1.333333333
rerglb	0.627906977	1.842105263
papss2a	0.627906977	1.198300283
pbk	0.627737226	1.08045977
ctns	0.627659574	2.023255814
cd248b	0.62745098	0.987730061
myof	0.627272727	1.229508197
plecb	0.627218935	1.442105263
thap7	0.627118644	0.936170213
5S_rRNA_544	0.627118644	1.010869565
sdr42e1	0.627118644	2.52173913
sfrp2	0.627090301	1.548295455
CABZ01077402.1	0.626865672	0.771428571
b4galt2	0.626865672	1.111111111
kera	0.626609442	1.274193548
si:ch211-269i23.2	0.626506024	1.375
cox6a2	0.62585034	1.064655172
ifitm1	0.625806452	1.175438596
adamts13	0.625	0.512195122
plch1	0.625	0.588235294
pex12	0.625	0.666666667
VILL	0.625	0.822222222
tectb	0.625	0.836363636
nbeal2	0.625	0.846153846
orc6	0.625	0.95
pyroxd2	0.625	0.955223881
si:ch73-171a6.2	0.625	1
si:dkey-150k17.2	0.625	1.105263158
foxb1b	0.625	1.125
si:dkey-244a7.1	0.625	1.307692308
si:ch211-194p6.10	0.625	1.375
lrrc51	0.625	1.384615385
ggt5a	0.625	1.394366197
mxra8a	0.625	1.470588235
fam83ha	0.625	1.510204082
rab4b	0.625	1.527777778
PTGES3L	0.625	1.564102564
BX571711.1	0.625	1.578947368
si:dkey-23k10.5	0.625	1.909090909
si:dkey-56e3.3	0.625	2.264705882

myo1ca	0.623931624	0.871287129
birc7	0.623762376	1.274509804
calb1	0.62345679	0.844444444
col14a1a	0.623376623	0.8
ppp1r3ab	0.623376623	2.833333333
gnav1	0.62295082	1.130434783
trpv4	0.622641509	0.837837838
mdc1	0.62244898	1.423076923
slc16a12a	0.622377622	1.083333333
slc47a1	0.622222222	0.829268293
si:ch211-127i16.2	0.622222222	0.886363636
tmem234	0.622222222	2.36
slc5a6b	0.621621622	1.142857143
hyal2a	0.621621622	1.153846154
zgc:153759	0.621621622	1.166666667
pdlim5a	0.621621622	1.368421053
si:dkey-7i4.19	0.621621622	1.75
rhbg	0.621341463	1.462550607
zgc:101664	0.621212121	1.135416667
ctu2	0.621212121	1.166666667
tead3b	0.621118012	1.290322581
ccbl2_2	0.620689655	0.461538462
si:ch211-253p18.5	0.620689655	0.806451613
CABZ01059120.1	0.620689655	0.923076923
CABZ01085241.1	0.620689655	0.956521739
foxq2	0.620689655	1.2
entpd2a.2	0.620689655	1.5
gig2o	0.620689655	1.5
foxq1b	0.620689655	1.567901235
SHISA2	0.620437956	1.582278481
wfdc1	0.62037037	0.697802198
mis12	0.620253165	0.833333333
si:ch211-103n10.5	0.62	0.673076923
si:ch211-246i5.5	0.62	0.692307692
minal	0.62	0.965517241
tbx18	0.62	1.03030303
itgb2	0.62	1.45
c1ql4b	0.62	1.458333333
ccdc88aa	0.619047619	0.68
si:ch73-29l19.1	0.619047619	0.72972973
si:ch1073-100f3.2	0.619047619	0.733333333
cdc14aa	0.619047619	0.761904762
bnip1a	0.619047619	1
frmpd1a	0.619047619	1.033333333
mat2al	0.619047619	1.133802817
lct	0.619047619	1.266666667
dgkzb	0.619047619	1.272727273
abcg8	0.619047619	1.545454545
prkar1ab	0.618834081	1.156028369
ralbp1	0.618705036	1.012048193
erbb3b	0.618644068	1.022222222
s100w	0.618181818	1.527272727
tmem160	0.617886179	0.785714286
cxcl20	0.617647059	1.066666667
urb2	0.617647059	1.076923077
tmem218	0.617647059	1.181818182
entpd8	0.617647059	1.703703704
slc27a6	0.617647059	1.736842105
commd3	0.617647059	1.796116505
si:ch211-71m22.1	0.617486339	1.060606061
pdx1	0.617021277	0.753846154

tril	0.616666667	1.056603774
FO704810.2	0.616666667	1.533333333
sprn	0.616600791	1.078787879
neu3.2	0.616541353	1.46031746
lratb	0.616438356	0.964285714
guk1a	0.616161616	1.535483871
ino80e_2	0.616161616	1.285714286
rrp36	0.61589404	2.065217391
slc5a8l	0.615384615	0.416666667
plppr5b	0.615384615	0.823529412
trpc6a	0.615384615	0.947368421
zgc:174862	0.615384615	1.2875
edn2	0.615384615	1.3125
si:ch211-81n22.1	0.615384615	1.333333333
si:ch73-206d17.1	0.615384615	1.333333333
SAT2 (1 of many)	0.615384615	1.636363636
ENKD1	0.615384615	1.675675676
im:7143333	0.615384615	1.85
slc25a51a	0.615384615	2.105263158
chia.1	0.615062762	1.048048048
loxa	0.614814815	0.892473118
irf2a	0.614814815	1.795454545
zgc:162509	0.614583333	4.272727273
syndig1l	0.614285714	0.511627907
ttyh3a	0.614285714	1.162162162
csad	0.614213198	1.485915493
mylk5	0.614173228	0.971563981
pof1b	0.61409396	1.459893048
pdzd2	0.614035088	0.647058824
si:ch211-248a14.8	0.614035088	0.857142857
si:ch211-286c4.6	0.613636364	0.790697674
sult2st1	0.613636364	1.109181141
slc23a1	0.613636364	1.142857143
CR855387.1	0.613636364	1.52173913
hyal4	0.613445378	1.343283582
fmodb	0.613333333	2.058823529
map1b	0.613065327	0.716216216
gb:ai877918	0.612903226	0.96
gpr157	0.612903226	1.074074074
st6gal2b	0.612903226	1.162790698
si:dkey-79f11.7	0.612903226	1.727272727
vamp8	0.612	2.491935484
crhb	0.611940299	1
comta	0.611702128	1.226415094
si:ch211-152f6.6	0.611510791	0.728571429
aurka	0.611510791	0.768518519
cx28.9	0.611510791	0.866666667
btbd17a	0.611111111	0.413793103
cyp2x10.2_1	0.611111111	0.846153846
samhd1	0.611111111	0.852941176
PARP9	0.611111111	1.142857143
ssuh2.4	0.611111111	1.466666667
C7orf50	0.611111111	1.7
PQLC1	0.611111111	3.16
dnajc21	0.611111111	1.222222222
myhb	0.610856269	1.049235993
cyp51	0.610738255	0.825396825
anpepa	0.610169492	1.379310345
gltpa	0.609929078	1.541666667
aldh3a2b	0.60989011	1.161290323
CABZ01056516.1	0.609756098	0.806451613

itga3a	0.609756098	0.915254237
CR855996.1	0.609756098	1.090909091
clec3ba	0.609756098	1.238095238
peli1a	0.609756098	1.421052632
rassf7a	0.60952381	1.174603175
nipsnap1	0.609090909	0.765957447
hnf4g	0.608695652	0.625
nudt1	0.608695652	0.761904762
rprma	0.608695652	0.842105263
CU571315.1	0.608695652	1
zgc:194392	0.608695652	1.550724638
si:busm1-163l24.4	0.608695652	1.571428571
mis18bp1	0.608695652	1.615384615
dram1	0.608695652	2.357142857
si:ch211-155k24.9	0.608695652	2.454545455
BX548067.3	0.608695652	3.571428571
rab27a	0.608490566	1.333333333
hspb7	0.608490566	2.950980392
slc26a2	0.608333333	1.44047619
CABZ01029822.1	0.608108108	0.791666667
btr12	0.608108108	1.634146341
pym1	0.608	0.580645161
gabrz	0.607843137	0.913043478
arl4aa	0.607843137	1.014285714
gls2a	0.607692308	2.958762887
aqp8b	0.607329843	0.40942029
TMEM229A	0.607142857	0.588235294
ccbe1	0.607142857	0.766666667
grap2a	0.607142857	0.85
marveld2b	0.607142857	1.230769231
ntm	0.607142857	1.420289855
si:dkey-82k12.7	0.607142857	2.04
aldh8a1	0.606557377	0.778481013
cyp2p7	0.606382979	2.512820513
rubcn	0.606060606	1.105263158
si:ch1073-184j22.2	0.606060606	1.6
STIM2 (1 of many)	0.606060606	2.032258065
pdzk1	0.605820106	0.763837638
rnasel3	0.605769231	1.062200957
si:ch73-74h11.1	0.605504587	2.5
si:ch73-52p7.1	0.605263158	0.536585366
ca7	0.605263158	0.714285714
trip13	0.605263158	1.03125
si:dkey-94e7.2	0.605263158	1.034482759
fam151b	0.605263158	1.161290323
pigh	0.605095541	2.876923077
coa3b	0.605042017	1.22972973
si:ch211-125o16.4	0.604982206	2.003058104
elovl2	0.604651163	0.913043478
fam131a	0.604651163	1.5
zgc:158852	0.604651163	0.9
elmod3	0.604166667	0.541666667
GUCY2C (1 of many)	0.604166667	0.903225806
mgat3a	0.604166667	1.730769231
rassf4	0.604166667	1.913043478
sptlc3	0.603896104	0.945054945
hkdc1	0.603603604	0.7
fabp11b	0.603448276	0.527272727
si:rp71-39b20.4	0.603448276	1.233333333
mtp	0.603174603	1.233606557
fdxr	0.602941176	0.6

mhc1zca	0.602941176	1.444444444
cdh17	0.602580645	0.996350365
pcyt2	0.602179837	2.335260116
rgs6	0.602040816	1.111111111
lgals3bpb	0.601941748	1.013333333
lrrfip1a	0.601941748	1.263888889
angptl7	0.601866252	1.207048458
si:ch211-215c18.3	0.6	1.448275862
syt6b	0.6	0.433333333
si:dkey-96n2.1	0.6	0.555555556
slc12a1	0.6	0.6
eps8l3b	0.6	0.7
sh2d3cb	0.6	0.703703704
crip3	0.6	0.726027397
nr1d4a	0.6	0.733333333
col8a1b	0.6	0.916666667
pmvk	0.6	0.933333333
hs3st4	0.6	1.05
kcnq5a	0.6	1.083333333
adap2	0.6	1.095238095
si:ch211-207c6.2	0.6	1.1
slc2a3b	0.6	1.142857143
PLD5	0.6	1.2
RFESD	0.6	1.3
ano8a	0.6	1.3
tmco4	0.6	1.32
adamts15b_2	0.6	1.384615385
RAB19	0.6	1.4
f2rl2	0.6	1.454545455
rab25a	0.6	1.491525424
dcakd	0.6	1.555555556
utp11l	0.6	1.754237288
si:dkey-77f5.14	0.6	1.8
tmem74b	0.6	1.925925926
si:ch73-27e22.3	0.6	1.947368421
si:ch211-273j9.2	0.6	2
tmem65	0.6	2.102272727
tmigd1	0.6	2.133333333
cdh29	0.6	2.23255814
fibinb	0.599118943	1.496296296
ponzr1	0.598984772	0.591666667
ANPEP (1 of many)_2	0.598290598	1.306666667
pm20d1.1	0.598214286	0.805
setbp1	0.598039216	1.204545455
muc5.1	0.597826087	1.813333333
ddr2b	0.597560976	0.836363636
ebp	0.597560976	1.078125
prss59.1	0.597363772	0.994205052
nmbb	0.597222222	1.05
ltk	0.597014925	1.138888889
syt6a	0.596491228	0.575757576
efna2b	0.596491228	1.365853659
ppp1r18	0.596153846	0.875
sycn.2	0.596045198	1.024778761
CABZ01088484.1	0.59602649	0.80952381
kcna1a	0.595959596	0.953488372
ccdc187	0.595959596	1.373134328
zgc:163073	0.595744681	1.744444444
plcxd1	0.595744681	0.891891892
si:dkey-110g7.8	0.595238095	1.043478261
si:ch211-51a6.2	0.595238095	1.185185185

adcyap1r1b	0.594771242	1.591397849
ephx1	0.594736842	0.89380531
fuom	0.594594595	0.666666667
CABZ01054405.1	0.594594595	0.791666667
zgc:162255	0.594594595	1.065934066
setd8b	0.594594595	1.29787234
CSGALNACT1 (1 of many)_1	0.594594595	1.357142857
rprml	0.594594595	1.473684211
adgrg4a	0.594594595	1.5
rp1l1a	0.594594595	1.555555556
si:ch211-244b2.4	0.594594595	3.0625
lrrc8aa	0.594444444	2.473684211
si:rp71-77l1.1	0.59437751	0.894444444
ociad2	0.594339623	0.764227642
gins1	0.594339623	1.202380952
slc12a5b	0.593908629	0.685714286
si:ch211-214p13.3	0.59375	0.629032258
scgn	0.593220339	1.348837209
apoa4a	0.593073593	0.862068966
tgfbr3	0.593023256	1.392156863
psip1	0.592920354	1.947368421
apobb.1	0.592640037	1.516203294
si:ch73-334e23.1	0.592592593	0.6
si:ch73-147f11.1	0.592592593	0.666666667
hapln4	0.592592593	0.714285714
itga4	0.592592593	0.827586207
ppp1r16a	0.592592593	1.071428571
trpt1	0.592592593	1.2
kif16bb	0.592592593	1.222222222
si:ch73-57a19.4	0.592592593	1.397379913
zgc:162879	0.592592593	1.75
abcb6b	0.592592593	1.8
RRP8	0.59223301	1.527272727
col8a1a	0.591549296	0.65
gdpd3a	0.591194969	1.414414414
spdl1	0.590909091	1.127659574
slc5a11	0.590909091	0.634146341
mettl23	0.590909091	0.823529412
TGM1 (1 of many)_2	0.590909091	0.871794872
cbln11	0.590909091	0.878787879
tmem8c	0.590909091	1.076923077
si:ch211-244b2.3	0.590909091	1.2
si:dkey-33c14.4	0.590909091	1.272727273
zgc:153935	0.590909091	1.594594595
si:dkey-286h2.5	0.590909091	1.909090909
phldb2a	0.59047619	1.214285714
znf648	0.59047619	1.391752577
kcnf1b	0.58974359	0.814814815
nradd	0.58974359	1.230769231
fam20a	0.58974359	1.322580645
xpnpep2	0.58974359	1.535714286
adrb2a	0.58974359	1.541666667
cnsta	0.58974359	1.730769231
tmem254	0.589442815	0.721003135
itga2.2	0.589285714	1.303030303
ngs	0.589041096	0.555555556
opn6a	0.588235294	0.379310345
crym	0.588235294	0.53125
gtse1	0.588235294	0.685714286
dpt	0.588235294	0.8
si:ch211-14a11.5	0.588235294	0.818181818

clcnk	0.588235294	1.071428571
arhgef10la	0.588235294	1.32
snx21	0.588235294	1.461538462
htr3b	0.588235294	1.538461538
CR450808.1	0.588235294	1.588235294
tmie	0.587301587	0.472727273
5S_rRNA_44	0.587301587	1.18
itgb3b	0.586956522	0.972972973
nde1	0.586956522	1.683333333
gprc5c	0.586538462	1.151515152
scn4bb	0.586538462	2.131578947
rab13	0.586419753	1.453781513
cx32.3	0.58630137	1.259385666
abca4a	0.586206897	0.588235294
si:dkey-16j16.4	0.586206897	1.285714286
si:dkey-29d8.3	0.586206897	1.6
ehbp1l1a	0.586206897	1.6
si:dkey-43f9.4	0.586206897	1.846153846
uts1	0.585365854	0.842105263
METTL12	0.585365854	0.909090909
zgc:171482	0.585365854	1.538461538
efcc1	0.585106383	0.948275862
si:dkey-91i10.3	0.58490566	0.994219653
si:ch211-256e16.11_1	0.58490566	1.086956522
si:dkey-228d14.5	0.58490566	3.15
zgc:112496	0.584615385	0.925373134
cbln10	0.584415584	0.92
ptpn6	0.584269663	0.967741935
rdh8b	0.583333333	0.432432432
mmachc	0.583333333	0.558823529
si:ch211-207d6.2	0.583333333	0.95
lama3	0.583333333	1
si:ch211-207j7.2	0.583333333	1.222222222
bin1b	0.583333333	1.227642276
zgc:172120	0.583333333	1.25
si:ch73-378g22.1	0.583333333	1.272727273
si:ch73-158p21.7	0.583333333	1.333333333
blf	0.583333333	1.823529412
si:dkey-188h10.3	0.583333333	2
il4r.1	0.583333333	2.178571429
nol12	0.582781457	1.094117647
cygb1	0.582733813	1.294520548
znf1035	0.582375479	2.175572519
chp1	0.582142857	2.4
znf292a	0.582089552	1.731343284
CR848841.1	0.581818182	0.620689655
f9b	0.581818182	0.821428571
si:ch73-376l24.2	0.581818182	1.025641026
nphp1	0.581395349	0.730769231
si:dkey-1j5.4	0.580645161	0.52
meox2a	0.580645161	1
rgl2	0.580645161	1.027027027
si:dkey-205h13.1	0.580645161	1.37037037
si:ch211-141e20.2	0.580645161	1.588235294
mfge8b	0.580357143	1.417910448
eml3	0.58	1.161290323
spon2b	0.579908676	1.211678832
ldlra	0.579908676	1.354166667
fam208b	0.579310345	1.670731707
si:dkeyp-77c8.3	0.578947368	0.515151515
pacrg	0.578947368	0.62962963

PLEKHG3	0.578947368	0.666666667
itgb7	0.578947368	1
glulc	0.578947368	1
si:ch73-59c19.2	0.578947368	1.090909091
trmu	0.578947368	1.12
itpkb	0.578947368	1.6
znf981	0.578947368	2.3
si:dkey-189n19.4	0.578947368	5.090909091
zgc:172145	0.578947368	1.714285714
arhgap31	0.578947368	1.833333333
zgc:112185	0.578947368	2.029411765
ogn	0.578512397	1.155487805
emilin1a	0.578512397	1.311688312
lgalslb	0.578125	0.744186047
si:ch211-130m23.2	0.577777778	1
drd4a	0.577777778	1.333333333
hspa12b	0.577777778	1.333333333
fndc1	0.576923077	1
TRIP10 (1 of many)	0.576923077	1
avpr2aa	0.576923077	1.105263158
si:dkey-93n13.4	0.576923077	1.128205128
si:dkey-240h12.4	0.576923077	1.869565217
si:ch211-286o17.1	0.576923077	1.934782609
grik1b	0.576271186	0.636363636
ca14	0.576271186	2.291666667
sept9b	0.576086957	2.125
galr2b	0.575757576	0.714285714
nmnat1	0.575757576	1.636363636
bco1l	0.575757576	2.095238095
scpp5	0.575342466	0.845303867
ugt5b2_1	0.575342466	1
si:dkey-222b8.1	0.575342466	1.046511628
si:dkey-192g7.3	0.575	1.090909091
fgf8a	0.575	1.185185185
MDFIC	0.575	1.357142857
trim69	0.574468085	1.275862069
vsig8a	0.574468085	1.954545455
si:dkey-202l22.6	0.573684211	2.654320988
aldh1l1_1	0.573426573	1.478991597
angptl2a	0.573333333	0.901785714
iqgap2	0.572916667	0.807692308
si:ch211-214p16.1	0.572916667	1.076923077
gdpd2	0.572815534	1.435643564
ece2b	0.572727273	1.15
cdc42ep1a	0.571428571	0.87804878
cldn15b	0.571428571	1.274509804
palb2	0.571428571	1.333333333
si:dkey-11n14.2	0.571428571	1.5
CABZ01039096.1	0.571428571	1.545454545
rca2.2	0.571428571	0.652173913
tfcp2l1	0.571428571	0.666666667
si:dkey-27b3.8	0.571428571	0.684210526
slc35e4	0.571428571	0.96969697
slc30a2	0.571428571	1
slc25a21	0.571428571	1.043478261
si:ch1073-249f22.1	0.571428571	1.242424242
si:ch211-120k19.1	0.571428571	1.25
b4galt5	0.571428571	1.285714286
hoxa9a_1	0.571428571	1.333333333
fam83b	0.571428571	1.6
znf977_1	0.571428571	2.052631579

gda	0.571428571	3.390243902
slc20a2	0.569364162	1.655367232
mcu	0.569037657	1.297468354
si:ch211-224m9.4	0.568965517	0.666666667
sema7a	0.568965517	0.9375
ppp1r14ab	0.568807339	1.814285714
tinagl1	0.568627451	1.171428571
igflr1	0.568181818	0.575757576
si:ch211-176l24.4	0.568181818	1.846153846
nr5a2	0.567567568	0.583333333
chek1	0.567567568	0.724137931
si:ch73-308m11.1	0.567567568	0.954545455
prf1.5	0.567567568	1
adamts9	0.567567568	1.411764706
bnipl	0.567567568	1.710144928
myo15b	0.567164179	0.590909091
SNTA1	0.566878981	1.346153846
asb12b	0.566666667	0.75862069
dnajc24	0.566666667	1.45
si:ch73-334d15.1	0.566371681	1.3
FAR2	0.56626506	1
CABZ01087906.1	0.565789474	0.865671642
lama2	0.56557377	1.174418605
si:dkey-12l12.1	0.56547619	1.311111111
AC024175.2	0.565371025	1.416666667
CAPN1 (1 of many)_2	0.565217391	1
si:rp71-5o12.3	0.565217391	1.055555556
fbn2a	0.565217391	1.133333333
si:ch211-145b13.6	0.565217391	1.178571429
prl	0.565217391	1.243243243
mical2a	0.565217391	1.285714286
hhat	0.565217391	1.357142857
FAM109B	0.565217391	1.5
slc9a3.2	0.565217391	1.5
si:ch211-133l5.8	0.565217391	1.666666667
tlr5b	0.565217391	2.875
TYMP	0.564516129	1.240740741
cbln12	0.564516129	1.305084746
baxb	0.564516129	1.605263158
casp8ap2	0.564516129	2.107142857
si:ch211-220i18.4	0.564102564	1.129032258
cyb5a	0.56400507	2.3075
zgc:63831	0.563829787	0.893333333
si:dkey-121j17.5	0.563636364	0.9
dlx4b	0.563636364	1.333333333
etnppl	0.563492063	1.694117647
b3gnt7l	0.562962963	1.207920792
si:ch73-44m9.5	0.562801932	1.091525424
scn4ba	0.562790698	1.165217391
cyp2r1	0.5625	0.64556962
MPP4 (1 of many)_2	0.5625	0.789473684
C9orf69	0.5625	0.916666667
smpd2b	0.5625	1.727272727
si:dkey-196j8.2	0.5625	0.625
bgna	0.5625	0.921568627
tnfrsf11a	0.5625	1.036363636
klhl40b	0.5625	1.21875
ap1m3	0.5625	1.53125
cyp2x8	0.5625	2.647058824
chia.6	0.561403509	0.511627907
fam65b	0.561403509	1.097222222

si:ch211-13k12.2	0.56122449	1.4375
si:ch211-207i20.2	0.56097561	0.964285714
znf1007	0.56097561	1.727272727
fhl2b	0.560509554	0.6953125
alas2	0.560283688	0.59
golgb1	0.560283688	1.385714286
slc26a3.2	0.56	1.164556962
slc25a18	0.56	1.314285714
si:rp71-68n21.9	0.56	0.558823529
GRB14	0.56	0.842105263
zgc:171489	0.56	0.842105263
invs	0.56	0.863636364
tspan13a	0.56	0.883116883
si:dkey-177p2.18	0.56	0.909090909
slc10a4	0.56	0.909090909
cyp46a1.1	0.56	1
pde11al	0.56	1.272727273
tbc1d2	0.56	1.3125
angpt2b	0.56	1.454545455
si:ch211-139a5.9	0.559800664	1.001788909
CABZ01059391.1	0.55952381	0.820895522
grhl3	0.559322034	1.404761905
ankrd6a	0.559322034	1.47826087
ccdc59	0.559322034	1.972222222
si:ch211-170d12.2	0.559139785	0.612244898
si:dkey-234i14.6	0.558823529	0.875
st8sia2	0.558823529	1.125
si:dkey-162h11.3	0.558823529	2.647058824
znf990	0.558823529	3.454545455
tspan4b	0.558685446	1.773109244
cnga3a	0.558659218	1.38028169
asah2	0.558558559	1.206896552
si:dkey-117n7.2	0.558333333	0.927272727
ddit3	0.558252427	2.141361257
elovl8a	0.558139535	0.770833333
nr1h4	0.558139535	1.387096774
slc7a1	0.558139535	1.708333333
rdh5	0.557894737	0.351351351
CABZ01069595.1	0.557692308	0.757894737
SYTL2	0.557692308	1.387096774
TATDN1	0.557142857	1.461538462
si:ch211-107p11.3	0.557142857	1.5
tmem88b	0.556338028	0.822429907
sult1st6	0.556034483	2.398148148
emp3b	0.555555556	0.451612903
itln3	0.555555556	0.666666667
si:dkey-70b23.2	0.555555556	1
wnt10a	0.555555556	1.083333333
cyp4v7	0.555555556	1.096774194
zgc:174315	0.555555556	1.1875
slc6a11a	0.555555556	1.434782609
dnmbp	0.555555556	1.520833333
pln	0.555555556	1.529411765
xirp1	0.555555556	1.782608696
FO904872.1	0.555555556	4.416666667
PTGDR2	0.555555556	1.105263158
c3a.2	0.554744526	0.659259259
s100b	0.554054054	1.136363636
si:ch211-236g6.1	0.554054054	1.474576271
rnf126	0.554054054	1.787878788
chs1	0.553846154	0.757575758

si:ch211-194e15.5	0.553846154	1.026315789
si:ch211-281l24.3	0.553571429	1.947916667
pdzd3b	0.553191489	0.714285714
grn1	0.553191489	0.803278689
mcoln1a	0.552147239	2.544303797
st6galnac4	0.551724138	0.636363636
casp6l1	0.551724138	1
pde4cb	0.551724138	1.117647059
foxo1b	0.551724138	1.470588235
cyp2ad2	0.551724138	1.580357143
EFNA4	0.551724138	1.9375
ttpa	0.551470588	2.164179104
si:dkeyp-11e3.1	0.551020408	2.186046512
ism2b	0.55	0.777777778
SLC9A1	0.55	0.8125
P2RY6	0.55	1.205882353
gfpt2	0.54950495	1.361111111
si:ch211-113j14.1	0.548780488	0.802083333
slc6a19a.2	0.548523207	0.895833333
lhx1b	0.548387097	0.85
cacnb4a	0.547619048	0.666666667
gltpd2	0.547619048	1.225
si:ch211-215k15.5	0.547619048	1.954545455
sytl2b	0.547619048	2.263157895
krt17	0.546325879	0.689879472
CABZ01067232.1	0.546255507	1.18128655
utp3	0.54587156	2.074380165
fbxl8	0.545454545	0.942857143
zgc:109982	0.545454545	0.954545455
MYO7B (1 of many)	0.545454545	0.447368421
ccl19a.1	0.545454545	0.470588235
calcrlb	0.545454545	1.121212121
MCOLN2	0.545454545	1.166666667
cenpj	0.545454545	1.733333333
zgc:65851	0.544927536	0.595092025
rx2	0.544642857	0.956521739
rsf1b.1_1	0.544600939	1.03875969
marveld1	0.544554455	1.538461538
slc25a47a	0.544117647	1.714285714
CABZ01016547.1	0.543859649	1.617647059
si:ch211-202f3.3	0.54368932	0.954545455
acvr1bb	0.543624161	1.108433735
frmd5	0.543478261	1.388888889
enpep	0.543046358	0.784090909
oip5	0.542857143	1.103448276
stx11b.2	0.542857143	1.142857143
wu:fc34e06	0.542857143	2.357142857
si:ch211-225p5.8	0.542372881	1.5
lbx1b	0.541666667	0.666666667
fut9d	0.541666667	1.17765043
si:dkeyp-10a3.2	0.540983607	1.292682927
zgc:175280	0.540540541	0.679245283
tgm1	0.54	1.205128205
fam78ab	0.538461538	0.736842105
prkd3	0.538461538	1.095238095
rin1a	0.538461538	1.3125
si:dkey-184p9.7	0.538461538	1.771428571
smtna	0.538461538	2
tmpob	0.53816047	1.260450161
si:ch211-203c7.2	0.537815126	1.553571429
hid1a	0.5375	1.352941176

xirp2a	0.5375	1.88372093
sult3st1_1	0.5375	2.903225806
zgc:193711	0.537313433	0.881355932
sh3yl1	0.537313433	1.545454545
slc15a1a	0.537313433	1.795918367
si:ch211-193k8.2	0.536585366	1.052631579
acap1	0.536585366	1.115384615
lca5	0.536585366	1.210526316
rpp40	0.536585366	1.344262295
si:ch1073-13h15.3	0.536443149	0.764102564
si:ch211-198d23.1	0.535714286	0.607142857
cadm1b	0.535714286	1.263157895
stard9	0.535714286	1.333333333
st3gal1l2	0.535714286	1.363636364
ostn	0.535714286	1.461538462
RBP1	0.534883721	0.565217391
si:dkey-206p8.1	0.534482759	0.910714286
camk1a	0.534482759	1.208333333
rabgap1l2	0.534246575	1.375
zgc:153896	0.534246575	1.450980392
rpe65c	0.533333333	0.371428571
CABZ01079986.1	0.533333333	0.625
slc25a27	0.533333333	0.8
haus3	0.533333333	1
cpz	0.533333333	1.245614035
pcxb	0.533081285	0.737569061
zgc:113531	0.53250774	1.434343434
hivep3a	0.531914894	1.102564103
tnfrsf1a	0.531914894	2.256410256
ca2	0.53164557	2.56039604
si:dkey-103j14.5	0.53125	0.52
ahsg1	0.53125	0.688888889
npvf	0.53125	0.763636364
adora2b	0.53125	0.96875
nkpd1	0.53125	1.5
bco2l	0.53125	1.538461538
zgc:172075	0.53125	2.133333333
lgr6	0.530864198	1.515151515
cdkn2d	0.530612245	0.387096774
adra2c	0.530612245	0.833333333
zbtb10	0.53030303	1.166666667
E2F2	0.529411765	0.675
BX088653.1	0.529411765	1.03125
slc16a7	0.529411765	0.5
CU459012.1	0.529411765	0.821428571
C14orf1	0.529411765	0.93814433
rtkn2b_2	0.529411765	1.333333333
tapbpl	0.528846154	1.484375
pprc1	0.52866242	0.823529412
FP236735.2	0.528571429	1.944444444
golt1a	0.528301887	3.19047619
itih6	0.527777778	0.636363636
muc5.2	0.527777778	1.818181818
aqp10a	0.527777778	2.310344828
appl1	0.527607362	1.915662651
si:dkey-25e11.10p	0.526666667	1.224489796
slc52a3	0.526315789	0.44
mtnr1aa	0.526315789	0.933333333
gulp1b	0.526315789	0.954545455
si:dkey-11o18.5	0.526315789	1
si:dkey-239i20.4	0.526315789	1.090909091

palmda	0.526315789	1.157894737
si:ch73-112l6.6	0.526315789	1.333333333
pmch	0.526315789	1.371428571
ptgir	0.523809524	1
si:dkey-250k15.7	0.523809524	1.117647059
alox5a	0.523809524	1.238095238
cenph	0.523809524	1.363636364
METTL4	0.523809524	1.533333333
cd44a	0.523809524	2.052631579
aoc1	0.522727273	0.710843373
echdc3	0.522727273	1.108433735
mylkb	0.522522523	1.533333333
si:ch211-105c13.3	0.522460938	2.636627907
guca2b	0.52238806	1.224
prelp	0.522292994	0.720930233
si:dkey-193p11.2	0.522292994	1.82815735
tm4sf5	0.522222222	1.294117647
sele	0.52173913	0.722222222
si:ch73-380l10.2	0.52173913	0.869565217
si:dkey-170l10.1	0.52173913	0.956521739
pign	0.52173913	1
si:dkey-11f4.14	0.52173913	1.294117647
pth1rb	0.52173913	2.071428571
TSC22D3	0.521008403	1.407407407
vav3a	0.520547945	0.65625
si:ch211-240l19.6	0.52027027	0.699481865
5-Mar	0.520231214	1.357798165
CU466240.1	0.52	0.703703704
abcb4	0.52	0.719101124
si:ch211-119e14.1	0.52	1.285714286
pcdh20	0.52	1.56097561
si:dkey-35m8.1	0.518518519	0.466666667
acmsd	0.518518519	0.760869565
zgc:56622	0.518518519	1.128205128
si:dkeyp-73d8.6	0.518518519	1.266666667
si:ch211-110e21.3	0.518518519	1.580246914
fgf1a	0.518518519	1.736842105
mogat2	0.517766497	0.993150685
adora1b	0.517241379	0.814814815
ntf3	0.517241379	1
slc25a37	0.517241379	1.363636364
fndc7rs2	0.517241379	1.757575758
ms4a17a.17_2	0.516666667	0.783783784
fgf11a	0.516129032	0.625
prnpb	0.516129032	0.682926829
CABZ01071407.1	0.516129032	0.75
fpr1	0.516129032	0.829268293
scap	0.516129032	1.042553191
golga7ba	0.515625	2.899371069
CABZ01068356.1	0.515625	0.49122807
FQ311892.1	0.515151515	1.285714286
rxrab	0.515151515	1.391304348
si:ch73-263o4.4	0.515151515	2.379310345
opn4xb	0.514285714	1.888888889
si:dkey-22f5.9	0.513513514	1.117647059
nkx3.3	0.513513514	1.409090909
mpdu1a	0.513157895	0.981132075
parvg	0.512820513	1.222222222
wnt16	0.512195122	0.703703704
si:ch211-212c13.6p	0.512195122	0.807692308
sostdc1b	0.512195122	1.096774194

gpc6a	0.511627907	0.730769231
TGM1 (1 of many)_1	0.511627907	0.821428571
CT990561.1	0.511111111	0.625
il11ra	0.510869565	0.984848485
mgll	0.510869565	1.756756757
slc31a1	0.510344828	1.708955224
si:dkey-10o6.2	0.510204082	1.086956522
mrc1b	0.510204082	1.483870968
creb3l3a	0.509803922	0.890625
egln3	0.50877193	0.71875
FAM84B (1 of many)	0.50877193	1.069444444
ackr4b	0.50877193	1.487179487
dio3b	0.507317073	2.358778626
si:ch211-220f16.2	0.507246377	1.951219512
OLFML2A (1 of many)	0.50617284	0.983050847
slc6a19a.1	0.505376344	0.328125
si:dkey-19n13.5	0.505050505	1.071428571
abcg5	0.5	0.555555556
laynb	0.5	0.606060606
rab42b	0.5	0.733333333
si:ch73-15n15.3	0.5	0.823529412
MYOF (1 of many)	0.5	0.833333333
ARHGAP18	0.5	0.920634921
fgf1b	0.5	0.954545455
obscna	0.5	1
si:ch211-277e21.2	0.5	1
si:ch211-208h16.4	0.5	1.028571429
gpr185b	0.5	1.04
mustn1b	0.5	1.090322581
st6galnac5a	0.5	1.095238095
si:ch211-287a12.9	0.5	1.113636364
ebi3	0.5	1.15
pla2g4f.1	0.5	1.157894737
chad	0.5	1.162790698
kcng3	0.5	1.181818182
si:ch211-95o16.4	0.5	1.235294118
cmtm4	0.5	1.271186441
mvb12ba	0.5	1.293103448
pdlim3a	0.5	1.379310345
akap12a	0.5	1.392857143
ikbkb	0.5	1.428571429
si:dkey-56m15.5	0.5	1.538461538
si:ch1073-228h2.2	0.5	1.545454545
bbc3	0.5	1.545454545
GADD45G (1 of many)	0.5	1.571428571
CR376821.1	0.5	1.611111111
slc9a2	0.5	1.681818182
zgc:113294	0.5	1.75
si:ch211-145o7.3	0.5	1.785714286
brca2	0.5	1.866666667
FAM107A (1 of many)_1	0.5	1.947368421
arhgef5	0.5	2.027027027
GPR89B	0.5	3.208333333
upp1	0.5	4.566666667
zgc:194839	0.5	5
csrp2	0.497512438	2.376237624
cyp1d1	0.496503497	0.956140351
lcn15	0.496062992	1.175257732
si:ch211-198m17.1	0.495049505	1.133333333
apoba	0.494264859	1.683848797
kctd9a	0.493975904	1.512820513

rpe65a	0.492662474	0.377300613
pip4k2ab	0.492537313	1.745454545
si:ch211-220p4.1	0.492307692	1.363636364
ido1	0.4921875	1.324675325
slc34a2a	0.491525424	0.698795181
slc26a6_2	0.490909091	0.961538462
fgfbp2a	0.490566038	0.972972973
plac8.1	0.490277778	1.983539095
napgb	0.490196078	1.253164557
si:ch211-135f11.1	0.489795918	1.619047619
mep1a.1	0.489795918	1.732142857
tmem97	0.489361702	1.015873016
syncripl	0.489236791	1.224899598
arid5b	0.488888889	1.151515152
lrrc15	0.488372093	1.75
abcb11a	0.487804878	0.523809524
smap2	0.487804878	1.5625
zgc:100918	0.486792453	1.570552147
ccdc85ca	0.486187845	2.32
lenep	0.486111111	0.782608696
dapp1	0.485714286	1.555555556
map7a	0.483870968	1.5
adgrd1	0.483870968	1.769230769
si:dkey-1f1.3_1	0.483870968	1.909090909
cacnb3b	0.481927711	1.909090909
slc27a4	0.481751825	0.696428571
cald1b	0.481481481	1.603448276
pcdh1g2	0.481481481	1.666666667
si:ch73-92e7.4	0.481481481	3.1
si:ch211-62a1.3	0.481283422	0.961832061
AL845295.3	0.48	1.294117647
CR382337.1	0.48	1.529411765
epm2a	0.48	2.545454545
cnga3b	0.478873239	0.612903226
S1PR3	0.47826087	0.571428571
si:ch73-281f12.1	0.47826087	1
myo7bb	0.47826087	1.125
b3gnt3	0.47826087	1.282051282
bco2a	0.477272727	1.222222222
CU104709.1	0.477272727	2.380952381
rbm24b	0.47706422	1.779069767
si:dkeyp-87e3.1	0.476496784	1.718922652
si:dkeyp-69c1.9	0.476190476	0.590909091
si:dkey-269o24.2	0.476190476	0.769230769
tex30	0.476190476	1.071428571
loxl5b	0.475	0.666666667
unc93b1	0.475	0.863636364
penkb	0.474226804	1.020408163
si:dkey-95h12.1	0.474226804	1.944954128
clul1	0.473684211	0.416666667
si:ch211-212c13.7p	0.473214286	0.972972973
bhlha9	0.472868217	1.512195122
pla2g1b	0.472527473	0.840909091
CU302253.1	0.472222222	1.25
popdc3	0.471698113	1.529411765
CABZ01034698.4	0.471698113	2.875
hcls1	0.471615721	2.071428571
cngb3.2	0.471428571	0.277777778
cx27.5	0.470588235	0.739130435
cts12	0.470588235	1.25
si:dkey-224j12.3	0.470588235	0.846153846

tspan9a	0.46969697	1.068965517
asb14b	0.469387755	0.641025641
si:ch73-140j24.4	0.469026549	3.130434783
si:dkey-27m7.4	0.46835443	2.159090909
HACD4	0.467889908	1.75
CABZ01055347.1	0.467105263	1.05952381
cx44.2	0.466666667	0.943396226
COLGALT1 (1 of many)	0.466666667	1.018518519
si:dkey-238d18.4	0.466666667	1.071428571
enpp4	0.466666667	1.428571429
BX548028.1	0.464285714	0.916666667
si:ch211-165b10.3	0.463414634	2.5
galnt12	0.463157895	1.711538462
gpr31	0.462686567	2.571428571
ahnak	0.461832061	1.633333333
rpusd1	0.461538462	2.055555556
abcd1	0.461538462	2.222222222
ppp2r5a	0.461538462	1.545454545
sqlea	0.46	1.310344828
impg2b_2	0.459770115	1.189189189
nnt2	0.458333333	1
si:dkey-53f14.5	0.458333333	1.8
si:dkey-259j3.5	0.458333333	1.923076923
RALYL	0.458333333	2.076923077
rfk	0.458333333	2.2
si:ch211-76l23.4	0.458333333	0.935483871
AC024175.19	0.457831325	0.872180451
zswim7	0.457142857	1.166666667
mettl7a	0.456140351	1.095238095
bfb	0.454545455	0.486111111
PPEF2	0.454545455	0.869565217
HBZ	0.454545455	1.296296296
nkx2.4b	0.454545455	0.5625
pygo1	0.454545455	1.058823529
cfap126	0.454545455	1.1
gramd4a	0.454545455	1.166666667
zgc:194626	0.454258675	1.072463768
cxcl8b.1	0.453125	1.628571429
caln1	0.452380952	0.923076923
si:ch211-69b7.6	0.452380952	2.074074074
gal3st2	0.451612903	1.571428571
cd40	0.451612903	2.75
rcvrna	0.450704225	1.359073359
si:dkeyp-97a10.2	0.45	1.260869565
CABZ01055168.1	0.45	1.4
FO681350.1	0.448275862	1.125
wisp1b	0.448275862	1.5625
faim2b	0.447761194	1.448979592
mpp4a	0.447368421	1.066666667
nicn1	0.446428571	2.68
si:ch211-133j6.3	0.445454545	1.603174603
ankrd37	0.444444444	1.417910448
CU467828.1	0.444444444	1.769230769
si:ch211-67o16.3p	0.444444444	0.728395062
slc6a6a	0.444444444	0.846153846
gyg2	0.444444444	1.294117647
si:ch73-288o11.5	0.442857143	1.842105263
fam151a	0.441176471	1.388888889
si:ch1073-126c3.2	0.441176471	1.681818182
cdc42ep2	0.440677966	1.4
5S_rRNA_350	0.440298507	0.959459459

fam26e.2	0.44	2.230769231
p2rx3a	0.44	2.909090909
anpepb	0.439516129	1.427184466
CU651662.2	0.43902439	1.555555556
si:ch211-145g9.7	0.4375	1.142857143
CABZ01045212.1	0.4375	1.529411765
asip1	0.436363636	2.461538462
si:ch211-274e20.4	0.434782609	0.761904762
ahsg2	0.434782609	0.8
si:dkey-73n8.3	0.434782609	0.84375
cdc14b_1	0.434782609	1.1
CT574587.1	0.434782609	2.352941176
si:dkey-78a14.2p	0.433962264	1.212121212
crygmxl1	0.433962264	1.260869565
RNF222	0.433333333	3.857142857
cyb561d2	0.432432432	1.473684211
gpc5a	0.431818182	0.882352941
dapk2b	0.431372549	1.175
si:dkey-86l18.10	0.430769231	0.953846154
si:dkey-125i10.3	0.430555556	2.894736842
FP102052.1	0.428571429	1.166666667
si:dkey-246g23.2	0.428571429	1.318181818
si:dkey-76i15.1	0.428571429	0.615384615
dfna5a	0.428571429	0.911764706
SYCN (1 of many)	0.428571429	1.186666667
FO904873.1	0.428571429	1.285714286
mfi2	0.427672956	0.654761905
NAT16	0.427230047	1.022988506
cmya5	0.426666667	1.586956522
serinc2l	0.426356589	1.538461538
TXNRD2	0.426229508	2.04
mylka_2	0.425925926	0.913043478
tm6sf2	0.425531915	0.325
mical1	0.425531915	1.2
si:dkey-246e1.3	0.424242424	0.962962963
si:dkey-32e6.3	0.424242424	1.142857143
aim1a	0.423357664	1.779411765
CR855996.2	0.423312883	0.447368421
si:ch211-167j9.4	0.423076923	0.846153846
atg4db	0.423076923	1.04
ptx3b	0.423076923	1.083333333
si:ch73-347e22.4	0.423076923	1.518518519
CABZ01079764.1	0.418867925	0.336065574
zgc:194224	0.418181818	1.588235294
si:ch211-240l19.5	0.41754386	1.287234043
lrrc38a	0.416666667	0.414634146
stat5a	0.416666667	1.875
slc6a19b	0.415841584	1.115384615
pho	0.415584416	1.959183673
cers3a	0.413793103	0.580645161
nceh1b	0.413793103	1.36
rbp7b	0.412698413	1
tbx21	0.411764706	1.083333333
si:dkey-18p12.4	0.411764706	2.545454545
matn3a	0.411504425	0.552083333
crb2b	0.410714286	1.333333333
CABZ01072043.1	0.409090909	0.485714286
lss	0.409090909	0.647058824
kitlgb	0.407407407	1.181818182
plbd1	0.406779661	2.233333333
kcnk2b	0.405405405	1.25

si:cabz01117780.1	0.405063291	1.179487179
ADGRG2 (1 of many)_2	0.404761905	1.21875
FO704618.1	0.403846154	1.52
dpep1	0.403225806	1.52
pleca	0.402810304	1.563157895
zgc:163121	0.4	0.9375
dnal4a	0.4	2.307692308
fgfr1bl_2	0.4	1.789473684
SLC25A22 (1 of many)_2	0.397435897	1.298969072
CR933839.1	0.396551724	0.514285714
stbd1	0.394736842	1.057692308
si:ch73-263o4.3	0.394736842	1.166666667
sfxn5b	0.393939394	2.014925373
si:dkey-95p16.1	0.393442623	2.5
si:dkey-262k9.2	0.392070485	1.919642857
ugp2a	0.392045455	1.885416667
c6ast4	0.391891892	0.787037037
LGALS3BP (1 of many)	0.391304348	0.8
prx	0.391025641	1.564705882
fam169aa	0.39047619	1.583333333
c1qtnf6a	0.389830508	0.794117647
zgc:153968	0.388101983	1.636363636
tnks1bp1	0.386792453	0.907894737
aco1	0.386075949	1.304964539
ftr14l	0.384615385	0.692307692
TMEM150B	0.384615385	1.041666667
si:dkey-58f10.12	0.383333333	2.476190476
si:ch211-286k11.4	0.382352941	0.407407407
igf2bp2a	0.381818182	1.011627907
si:ch211-199i15.5	0.380952381	1.578947368
slc29a1b	0.38	1
si:ch211-60d22.1	0.378947368	1.026315789
gpa33	0.378947368	2.111111111
si:ch211-173m16.1	0.378378378	0.239130435
si:dkey-222e15.4	0.375	0.863636364
dyrk4	0.375	2.127272727
map1ab	0.371794872	0.954022989
hbl3	0.370588235	0.928571429
ftr97_2	0.37037037	1.692307692
rap1gap2a	0.369230769	1.027777778
si:dkey-33c14.2	0.368421053	1
zanl	0.367816092	0.655172414
glt8d2	0.366666667	0.866666667
znf185	0.366666667	1.955555556
dnajc22	0.365853659	1.696969697
sult3st3_2	0.363636364	0.819875776
si:dkey-7c18.24	0.363636364	4.01754386
AC024175.14	0.363636364	0.754342432
aqp4	0.362962963	1.313432836
crfb17	0.359375	2.090909091
CR376783.1	0.357142857	1.033333333
chmp2ba	0.357142857	1.114285714
ube2d4	0.355072464	0.775510204
plekhf1	0.353535354	1
cmn	0.352112676	1
adgrf6	0.350877193	2.125
si:ch211-108d22.2	0.348314607	2.285714286
si:ch211-170d8.5	0.346938776	2.529411765
si:ch211-266g18.10	0.345537757	1.92244898
stk35l	0.345	2.385714286
mxra5a	0.344262295	1.111111111

CSDC2 (1 of many)	0.343283582	3.523809524
ebpl	0.342857143	0.677419355
si:ch211-150g5.4	0.342105263	2.457142857
vhll	0.341463415	1.875
lrata	0.340425532	0.416666667
cabp2a	0.340425532	0.642857143
si:dkeyp-77c8.1	0.340425532	1.541666667
si:ch73-31d8.2	0.338461538	0.450704225
si:dkey-22i16.7	0.333333333	0.432432432
AC024175.7	0.333333333	3.19047619
evpla	0.326271186	2.330645161
tnxba	0.325203252	1.533333333
si:ch211-196f2.3	0.320754717	2.105263158
sult3st2	0.319148936	1.761904762
dera	0.319047619	3.131868132
prg4b	0.317073171	1.172413793
csrp3	0.3125	1
eppk1	0.3125	1.306666667
fam167b	0.308333333	1.116666667
KCNIP4 (1 of many)_1	0.307692308	1.055555556
si:dkey-84h14.4	0.307692308	1.5625
rhcgl1	0.307432432	4.555555556
crygm5	0.306666667	2.148148148
CR735126.1	0.3	0.734693878
acsbg1	0.3	1.111111111
si:ch211-213d14.2	0.294871795	0.676923077
ccl19b	0.294117647	0.694805195
cenpf	0.290697674	1.862745098
zgc:194398	0.28436019	1.650406504
si:dkey-77p23.8	0.28340081	2.94534413
tbl1x	0.276923077	2.043478261
si:dkey-201i2.1	0.273809524	2.7625
cers1	0.266055046	2.097560976
ACER2	0.264150943	2.217391304
zgc:136461	0.258799172	0.9543379
si:ch211-250g4.3	0.25	0.625
si:ch211-153f2.3	0.244444444	0.891891892
rab8a	0.23030303	2.046153846
si:ch211-161m3.7	0.217391304	3.133333333
CABZ01059415.2	0.213740458	5.272727273
ptch1_1	0.210526316	0.644444444
si:dkey-31i7.1	0.196721311	2.785714286
vwa11	0.183206107	1.263157895
