# Supplementary material for: Soy-Derived Equol Induces Antioxidant Activity in Zebrafish in an Nrf2-Independent Manner
Source: Int J Mol Sci. 2022 May 8;23(9):5243. doi: 10.3390/ijms23095243 (PMC9105299; doi:10.3390/ijms23095243)
Supplement: Supplementary file 1 [file ijms-23-05243-s001.zip › ijms22.watanabe.TableS2.pdf]

Table S2. Upregulated genes by equol treatment in both wild-type and *nfe2l2a*^{fh318} larvae.

Genes	Gene names in ZFIN	Human homologs	Equol-treated vs untreated in wild-type larvae	Equol-treated vs untreated in *nfe2l2a*^{fh318} larvae
egr2a_2	egr2a	EGR2	4.615384615	1.697674419
fosl1a	fosl1a	FOSL1	3.630434783	1.960526316
btg2	btg2	BTG2	3.557291667	1.666666667
gsta.1	gsta.1	GSTA3	3.468235294	1.963636364
rgs2	rgs2	RGS2	3.028571429	2.791666667
hspb9	hspb9	HSPB9	2.730519481	3.135371179
sgk1	sgk1	SGK1	2.719587629	1.83032491
CR855274.1			2.547169811	1.633757962
enpp7.1	enpp7.1	ENPP7	2.545454545	3.526315789
si:dkey-14d8.23_1	si:dkey-14d8.23		2.545454545	4.153846154
fosb	fosb	FOSB	2.53125	1.968253968
epgn	epgn	EPGN	2.509433962	3.058823529
BX005085.3			2.466666667	2.125
igfbp1b	igfbp1b	IGFBP1	2.463414634	2.5
FO704777.1			2.461538462	1.7
csrnp1a	csrnp1a	CSRNP1	2.45631068	2.427083333
egr3	egr3	EGR3	2.405405405	2.256410256
adamts1	adamts1	ADAMTS1	2.3515625	1.570588235
si:ch211-130h14.6_2	si:ch211-130h14.6		2.333333333	2.708333333
dusp1	dusp1	DUSP1	2.286021505	1.503793627
si:dkeyp-55h4.2	si:dkeyp-55h4.2		2.285714286	2.25
atoh1c	atoh1c	ATOH1	2.272727273	1.894736842
nr4a1	nr4a1	NR4A1	2.23495702	1.820247934
gadd45ba	gadd45ba	GADD45B	2.217665615	1.765072765
nr4a3	nr4a3	NR4A3	2.214285714	1.557377049
si:ch211-227n13.3	si:ch211-227n13.3		2.2	1.6
OTUD1		OTUD1	2.192771084	1.802469136
si:dkeyp-2e4.5	si:dkeyp-2e4.5		2.133333333	2.333333333
rgs13	rgs13	RGS1	2.130434783	1.90625
cyp24a1	cyp24a1	CYP24A1	2.103286385	2.649350649
si:dkey-29b5.3	si:dkey-29b5.3		2.1	1.926229508
si:dkey-79d12.5	si:dkey-79d12.5	BTG3	2.077777778	4.24137931
ms4a17a.2	ms4a17a.2	MS4A8	2.076923077	1.928571429
tcap	tcap	TCAP	2.072380952	1.939265537
slc25a38a	slc25a38a	SLC25A38	2.071428571	1.5
si:dkey-88n24.12	si:dkey-88n24.12		2.066666667	3.090909091
dusp5	dusp5	DUSP5	2.064516129	1.689265537
ERRFI1 (1 of many)		ERRFI1	2	2.007575758
helt	helt	HELT	2	1.785714286
si:dkey-242g16.2	ppp1r3c2a		2	3.539473684
si:dkey-267n13.1	si:dkey-267n13.1		2	1.8125
f2rl1.2	f2rl1.2	F2RL1	1.974683544	2.475
srsf3a_2	srsf3a	SRSF3	1.96	2.941176471
si:ch211-212k5.1	si:ch211-212k5.1		1.933333333	1.631578947
si:dkey-108k21.21	si:dkey-108k21.21		1.928571429	1.5
bsx	bsx	BSX	1.888888889	1.894736842
gadd45bb	gadd45bb	GADD45B	1.884615385	1.683168317
hamp	hamp	HAMP	1.873015873	3.145833333
znf1161	znf1161	ZNF729	1.869565217	1.789473684
si:dkey-47k20.6	si:dkey-47k20.6		1.853658537	1.857142857
zgc:109934	fthl31	FTMT	1.849315068	1.647181628
spx	spx	SPX	1.846153846	1.583333333
zgc:174259	zgc:174259	SERPINA12	1.842105263	2.590909091
zgc:175284	zgc:175284		1.842105263	1.888888889
rasd1	rasd1	RASD1	1.841791045	1.538461538
nfkbiaa	nfkbiaa	NFKBIA	1.830827068	2.116618076
zgc:113274	zgc:113274		1.823529412	1.6
kcnj1a.4	kcnj1a.4	KCNJ1	1.819148936	2.293103448
zgc:152698	zgc:152698		1.818181818	1.590909091
zgc:153911	rasd3		1.808510638	2.783783784
znf1171	znf1171	ZNF729	1.8	2.111111111
cebpd	cebpd	CEBPD	1.781094527	1.661966156

si:dkey-47k16.4_2	si:dkey-47k16.4		1.777777778	2.846153846
plek2	plek2	PLEK2	1.777777778	1.837837838
ppcdc	ppcdc	PPCDC	1.772727273	1.615384615
CABZ01012147.1			1.769230769	2.083333333
si:dkey-15h8.16	si:dkey-15h8.16		1.769230769	2.071428571
si:ch211-114l13.11	si:ch211-114l13.11		1.765060241	1.788018433
btr04	btr04	TRIM11	1.740740741	1.80952381
si:dkey-50i6.6	si:dkey-50i6.6		1.725806452	1.964912281
si:dkey-246j7.1	si:dkey-246j7.1		1.714285714	2
sik1	sik1	SIK1	1.711934156	1.617586912
LECT2 (1 of many)_1		LECT2	1.709677419	1.625
ccr9a	ccr9a	CCR9	1.705882353	2.357142857
klf2a	klf2a	KLF2	1.696048632	1.5
mlnl	mlnl		1.689655172	1.518518519
si:ch211-113a14.22	si:ch211-113a14.22	H2BC21	1.666666667	2.083333333
ropn1l	ropn1l	ROPN1L	1.666666667	2
si:cabz01023255.2	si:cabz01023255.2		1.666666667	2.5
CABZ01021450.4			1.666666667	1.727272727
si:dkey-21o19.2	si:dkey-21o19.2		1.666666667	1.631578947
stk19	stk19	STK19	1.657142857	2.151515152
kcnj1a.3	kcnj1a.3	KCNJ1	1.653465347	2.651515152
hspb11	hspb11	HSPB9	1.649350649	2.014388489
ca4c	ca4c	CA4	1.625	2.391304348
kcnj1a.6	kcnj1a.6	KCNJ1	1.62195122	2.71875
mppe1	mppe1	MPPE1	1.615384615	1.714285714
si:ch73-59f11.3	si:ch73-59f11.3		1.611111111	2.230769231
si:dkey-33c14.7	si:dkey-33c14.7		1.611111111	1.689655172
minpp1b	minpp1b	MINPP1	1.6	1.652173913
si:ch73-92e7.6	si:ch73-92e7.6		1.6	2.769230769
rbm41	rbm41	RBM41	1.588235294	2.272727273
upp2	upp2	UPP2	1.583333333	5
si:dkey-71b5.3	si:dkey-71b5.3		1.583333333	1.677419355
si:dkey-85k7.7	si:dkey-85k7.7		1.576923077	1.951020408
cx32.2	cx32.2	GJA4	1.571428571	1.916666667
si:dkey-221l4.10	si:dkey-221l4.10		1.571428571	1.535714286
si:ch211-186e20.6p	si:ch211-186e20.6p		1.568965517	1.857142857
si:ch73-299h12.6	si:ch73-299h12.6		1.555555556	1.652173913
myf6	myf6	MYF6	1.553191489	2.515151515
si:dkey-248g15.2	ccdc9b	CCDC9	1.545454545	1.608695652
BX004873.4			1.538461538	2.227272727
si:ch1073-365n21.1	si:ch1073-365n21.1		1.531914894	1.725
lmx1ba	lmx1ba	LMX1B	1.53125	2.074074074
aplnr2	aplnr2	APLNR	1.52	1.583333333
myd88	myd88	MYD88	1.514018692	2.008403361
ogfod2	ogfod2	OGFOD2	1.512820513	2
gadd45ab	gadd45ab	GADD45A	1.502538071	1.864321608
renbp	renbp	RENBP	1.5	1.971428571
erich3	erich3	ERICH3	1.5	1.9
bcl2l11	bcl2l11	BCL2L11	1.5	2.071428571
cdc42l2	cdc42l2	CDC42	1.5	2.9
si:dkey-30k6.7	si:dkey-30k6.7		1.5	2
si:ch73-80e5.4	si:ch73-80e5.4		1.5	1.916666667
