# Supplementary material for: Soy-Derived Equol Induces Antioxidant Activity in Zebrafish in an Nrf2-Independent Manner
Source: Int J Mol Sci. 2022 May 8;23(9):5243. doi: 10.3390/ijms23095243 (PMC9105299; doi:10.3390/ijms23095243)
Supplement: Supplementary file 1 [file ijms-23-05243-s001.zip › ijms22.watanabe.TableS3.pdf]

Table S3. Downregulated genes by equol treatment in both wild-type and *nfe2l2a*^{fh318} larvae.

Genes	Gene names in ZFIN	Human homologs	Equol-treated vs untreated in wild-type larvae	Equol-treated vs untreated in *nfe2l2a*^{fh318} larvae
ptch1_1	ptch1	PTCH1	0.210526316	0.644444444
si:ch211-250g4.3	si:ch211-250g4.3		0.25	0.625
si:dkey-22i16.7	si:dkey-22i16.7		0.333333333	0.432432432
si:ch73-31d8.2	si:ch73-31d8.2		0.338461538	0.450704225
cabp2a	cabp2a	CABP2	0.340425532	0.642857143
lrata	lrata	LRAT	0.340425532	0.416666667
zanl	zanl	ZAN	0.367816092	0.655172414
si:ch211-173m16.1	si:ch211-173m16.1		0.378378378	0.239130435
si:ch211-286k11.4	cdhr5b	CDHR5	0.382352941	0.407407407
CR933839.1			0.396551724	0.514285714
lss	lss	LSS	0.409090909	0.647058824
CABZ01072043.1			0.409090909	0.485714286
matn3a	matn3a	MATN3	0.411504425	0.552083333
cers3a	cers3a	CERS3	0.413793103	0.580645161
lrrc38a	lrrc38a	LRRC38	0.416666667	0.414634146
CABZ01079764.1			0.418867925	0.336065574
CR855996.2			0.423312883	0.447368421
tm6sf2	tm6sf2	TM6SF2	0.425531915	0.325
mfi2	meltf	MELTF	0.427672956	0.654761905
si:dkey-76i15.1	vimr2		0.428571429	0.615384615
nkx2.4b	nkx2.4b	NKX2-4	0.454545455	0.5625
bfb	bfb	CFB	0.454545455	0.486111111
asb14b	asb14b	ASB14	0.469387755	0.641025641
cngb3.2	cngb3.2	CNGB3	0.471428571	0.277777778
clul1	clul1	CLUL1	0.473684211	0.416666667
loxl5b	loxl5b	LOXL4	0.475	0.666666667
si:dkeyp-69c1.9	si:dkeyp-69c1.9		0.476190476	0.590909091
S1PR3		S1PR3	0.47826087	0.571428571
cnga3b	cnga3b	CNGA3	0.478873239	0.612903226
abcb11a	abcb11a	ABCB11	0.487804878	0.523809524
rpe65a	rpe65a	RPE65	0.492662474	0.377300613
abcg5	abcg5	ABCG5	0.5	0.555555556
laynb	laynb	LAYN	0.5	0.606060606
slc6a19a.1	slc6a19a.1	SLC6A19	0.505376344	0.328125
CT990561.1			0.511111111	0.625
CABZ01068356.1			0.515625	0.49122807
fgf11a	fgf11a	FGF11	0.516129032	0.625
si:dkey-35m8.1	si:dkey-35m8.1		0.518518519	0.466666667
vav3a	vav3a	VAV3	0.520547945	0.65625
slc52a3	slc52a3	SLC52A3	0.526315789	0.44
itih6	itih6	ITIH6	0.527777778	0.636363636
slc16a7	slc16a7		0.529411765	0.5
cdkn2d	cdkn2d	CDKN2D	0.530612245	0.387096774
si:dkey-103j14.5	si:dkey-103j14.5	ASRGL1	0.53125	0.52
rpe65c	rpe65c	RPE65	0.533333333	0.371428571
CABZ01079986.1			0.533333333	0.625
RBP1		RBP1	0.534883721	0.565217391
si:ch211-198d23.1	si:ch211-198d23.1		0.535714286	0.607142857
lbx1b	lbx1b	LBX1	0.541666667	0.666666667
zgc:65851	zgc:65851		0.544927536	0.595092025
ccl19a.1	ccl19a.1	CCL19	0.545454545	0.470588235
MYO7B (1 of many)		MYO7B	0.545454545	0.447368421
cacnb4a	cacnb4a	CACNB4	0.547619048	0.666666667
st6galnac4	st6galnac4	ST6GALNAC4	0.551724138	0.636363636
c3a.2	c3a.2	C3	0.554744526	0.659259259
emp3b	emp3b	EMP3	0.555555556	0.451612903
itln3	itln3	ITLN1	0.555555556	0.666666667
rdh5	rdh5	RDH5	0.557894737	0.351351351
si:ch211-170d12.2	si:ch211-170d12.2		0.559139785	0.612244898
si:rp71-68n21.9	si:rp71-68n21.9	KLHL9	0.56	0.558823529
alas2	alas2	ALAS2	0.560283688	0.59
chia.6	chia.6	CHIA	0.561403509	0.511627907

si:dkey-196j8.2	si:dkey-196j8.2		0.5625	0.625
cyp2r1	cyp2r1	CYP2R1	0.5625	0.64556962
myo15b	myo15b	MYO15B	0.567164179	0.590909091
nr5a2	nr5a2	NR5A2	0.567567568	0.583333333
igflr1	igflr1	IGFLR1	0.568181818	0.575757576
si:ch211-224m9.4	si:ch211-224m9.4		0.568965517	0.666666667
rca2.2	rca2.2	C4BPA	0.571428571	0.652173913
tfcp2l1	tfcp2l1	TFCP2L1	0.571428571	0.666666667
grik1b	grik1b	GRIK1	0.576271186	0.636363636
pacrg	pacrg	PACRG	0.578947368	0.62962963
PLEKHG3		PLEKHG3	0.578947368	0.666666667
si:dkeyp-77c8.3	si:dkeyp-77c8.3		0.578947368	0.515151515
si:dkey-1j5.4	si:dkey-1j5.4		0.580645161	0.52
CR848841.1			0.581818182	0.620689655
mmachc	mmachc	MMACHC	0.583333333	0.558823529
rdh8b	rdh8b	RDH8	0.583333333	0.432432432
abca4a	abca4a	ABCA4	0.586206897	0.588235294
tmie	tmie	TMIE	0.587301587	0.472727273
crym	crym	CRYM	0.588235294	0.53125
opn6a	opn6a	OPN5	0.588235294	0.379310345
ngs	ngs		0.589041096	0.555555556
slc5a11	slc5a11	SLC5A11	0.590909091	0.634146341
col8a1a	col8a1a	COL8A1	0.591549296	0.65
si:ch73-147f11.1	dipk2b	DIPK2B	0.592592593	0.666666667
si:ch73-334e23.1	si:ch73-334e23.1		0.592592593	0.6
si:ch211-214p13.3	si:ch211-214p13.3		0.59375	0.629032258
fuom	fuom	FUOM	0.594594595	0.666666667
syt6a	syt6a	SYT6	0.596491228	0.575757576
ponzr1	ponzr1		0.598984772	0.591666667
slc12a1	slc12a1	SLC12A1	0.6	0.6
syt6b	syt6b	SYT6	0.6	0.433333333
si:dkey-96n2.1	si:dkey-96n2.1		0.6	0.555555556
fdxr	fdxr	FDXR	0.602941176	0.6
fabp11b	fabp4b	FABP4	0.603448276	0.527272727
elmod3	elmod3	ELMOD3	0.604166667	0.541666667
si:ch73-52p7.1	si:ch73-52p7.1		0.605263158	0.536585366
TMEM229A		TMEM229A	0.607142857	0.588235294
aqp8b	aqp8b	AQP8	0.607329843	0.40942029
pym1	pym1	PYM1	0.608	0.580645161
hnf4g	hnf4g	HNF4G	0.608695652	0.625
btbd17a	btbd17a	BTBD17	0.611111111	0.413793103
pdzd2	pdzd2	PDZD2	0.614035088	0.647058824
syndig1l	syndig1l	SYNDIG1L	0.614285714	0.511627907
slc5a8l	slc5a8l	SLC5A8	0.615384615	0.416666667
ccbl2_2	kyat3	KYAT3	0.620689655	0.461538462
adamts13	adamts13	ADAMTS13	0.625	0.512195122
pex12	pex12	PEX12	0.625	0.666666667
plch1	plch1	PLCH1	0.625	0.588235294
cntnap1	cntnap1	CNTNAP1	0.627906977	0.454545455
mb	mb	MB	0.633270321	0.623471883
vil1	vil1	VIL1	0.637007078	0.597449909
vstm4b	vstm4b	VSTM4	0.638297872	0.619047619
cnrip1b	cnrip1b	CNRIP1	0.639344262	0.6
sncga	sncga	SNCG	0.639344262	0.651515152
slc13a3	slc13a3	SLC13A3	0.64	0.596153846
zgc:162331	zgc:162331	SUSD3	0.64	0.41509434
CU855947.1			0.64	0.615384615
slc5a9	slc5a9	SLC5A9	0.640625	0.452830189
si:dkey-117m1.4	si:dkey-117m1.4		0.641304348	0.660377358
antxr1b	antxr1b	ANTXR1	0.642857143	0.458333333
nkain2	nkain2	NKAIN2	0.64516129	0.303571429
cnga1	cnga1b	CNGA1	0.647058824	0.566666667
adipoqb	adipoqb	ADIPOQ	0.65	0.630434783
mycla	mycla	MYCL	0.651376147	0.43220339
si:ch1073-82l19.1	zyg11l	ZYG11B	0.652173913	0.6
si:dkey-181d19.6	si:dkey-181d19.6		0.652173913	0.608695652
zgc:110782	zgc:110782		0.653061224	0.606060606

plekhd1	plekhd1	PLEKHD1	0.653846154	0.653846154
si:ch211-102c2.4	si:ch211-102c2.4		0.653846154	0.666666667
brf2	brf2	BRF2	0.655172414	0.444444444
gabra6a	gabra6a	GABRA6	0.655737705	0.555555556
urad	urad	URAD	0.657142857	0.589041096
enpp6	enpp6	ENPP6	0.658536585	0.435897436
dpf1	dpf1	DPF1	0.659090909	0.637681159
asb10	asb10	ASB10	0.662337662	0.632075472
slc15a1b	slc15a1b	SLC15A1	0.662721893	0.622222222
cntn4_1	cntn4	CNTN4	0.666666667	0.578947368
faah2b	faah2b	FAAH2	0.666666667	0.318181818
gpr88	gpr88	GPR88	0.666666667	0.666666667
rlbp1b	rlbp1b	RLBP1	0.666666667	0.469565217
si:dkey-156n14.3_1	si:dkey-156n14.3	ZXDB	0.666666667	0.566666667
kcna4	kcna4	KCNA4	0.666666667	0.666666667
