# Supplementary material for: Soy-Derived Equol Induces Antioxidant Activity in Zebrafish in an Nrf2-Independent Manner
Source: Int J Mol Sci. 2022 May 8;23(9):5243. doi: 10.3390/ijms23095243 (PMC9105299; doi:10.3390/ijms23095243)
Supplement: Supplementary file 1 [file ijms-23-05243-s001.zip › ijms22.watanabe.TableS4.pdf]

Table S4. Biological processes upregulated by equol treatment.

Term	Count	%	P Value	Genes	List Total	Pop Hits	Pop Total	Fold Enrichment	Bonferroni	Benjamini	FDR
GO:0045944~positive regulation of transcription from RNA polymerase II promoter	18	26.47	1.73E-07	CSRNP1, EGR2, EGR3, HELT, CEBPD, BSX, KLF2, NFKBIA, NR4A1, ZNF729, NR4A3, MYF6, F2RL1, FOSB, SPX, LMX1B, HAMP, ATOH1	64	1198	19349	4.54249	1.23E-04	1.23E-04	1.22E-04
GO:0046330~positive regulation of JNK cascade	5	7.35	2.60E-04	CDC42, GADD45B, GADD45A, F2RL1, MYD88	64	95	19349	15.91201	0.16915	0.06847	0.06827
GO:0061469~regulation of type B pancreatic cell proliferation	3	4.41	2.88E-04	NR4A1, ERRFI1, NR4A3	64	8	19349	113.37305	0.18570	0.06847	0.06827
GO:0035914~skeletal muscle cell differentiation	4	5.88	4.23E-04	NR4A1, EGR2, BTG2, MYF6	64	45	19349	26.87361	0.26031	0.07537	0.07515
GO:0006357~regulation of transcription from RNA polymerase II promoter	15	22.06	0.00102	CSRNP1, EGR2, EGR3, HELT, CEBPD, BSX, KLF2, FOSL1, NR4A1, ZNF729, NR4A3, MYF6, FOSB, LMX1B, ATOH1	64	1713	19349	2.64736	0.51701	0.14548	0.14507
GO:0045444~fat cell differentiation	4	5.88	0.00161	NR4A1, EGR2, NR4A3, CEBPD	64	71	19349	17.03257	0.68155	0.19056	0.19002
GO:0006915~apoptotic process	8	11.76	0.00282	NFKBIA, CSRNP1, NR4A1, BCL2L11, GADD45B, GADD45A, SGK1, MYD88	64	584	19349	4.14148	0.86611	0.28685	0.28604
GO:0042059~negative regulation of epidermal growth factor receptor signaling pathway	3	4.41	0.00646	CDC42, ERRFI1, EPGN	64	37	19349	24.51309	0.99007	0.57470	0.57309
GO:0043065~positive regulation of apoptotic process	6	8.82	0.00771	FOSL1, NR4A1, BCL2L11, GADD45B, DUSP1, GADD45A	64	378	19349	4.79886	0.99596	0.60989	0.60818
GO:2000341~regulation of chemokine (C-X-C motif) ligand 2 production	2	2.94	0.00974	F2RL1, MYD88	64	3	19349	201.55208	0.99906	0.66441	0.66254
GO:0051591~response to cAMP	3	4.41	0.01026	FOSL1, DUSP1, FOSB	64	47	19349	19.29754	0.99935	0.66441	0.66254
GO:0071376~cellular response to corticotropin-releasing hormone stimulus	2	2.94	0.01618	NR4A1, NR4A3	64	5	19349	120.93125	0.99999	0.88516	0.88267
GO:0008285~negative regulation of cell proliferation	6	8.82	0.01647	FOSL1, BTG3, BTG2, DUSP1, ADAMTS1, ENPP7	64	457	19349	3.96930	0.99999	0.88516	0.88267
GO:0009612~response to mechanical stimulus	3	4.41	0.01740	FOSL1, BTG2, FOSB	64	62	19349	14.62878	1.00000	0.88516	0.88267
GO:0035767~endothelial cell chemotaxis	2	2.94	0.02893	NR4A1, EGR3	64	9	19349	67.18403	1.00000	1.00000	0.99859
GO:0001701~in utero embryonic development	4	5.88	0.03110	FOSL1, BCL2L11, LMX1B, KLF2	64	210	19349	5.75863	1.00000	1.00000	0.99859
GO:0002262~myeloid cell homeostasis	2	2.94	0.03209	BCL2L11, HAMP	64	10	19349	60.46562	1.00000	1.00000	0.99859
GO:0000185~activation of MAPKKK activity	2	2.94	0.03209	GADD45B, GADD45A	64	10	19349	60.46562	1.00000	1.00000	0.99859
GO:0031274~positive regulation of pseudopodium assembly	2	2.94	0.04152	CDC42, F2RL1	64	13	19349	46.51202	1.00000	1.00000	0.99859
GO:0001706~endoderm formation	2	2.94	0.04152	DUSP5, DUSP1	64	13	19349	46.51202	1.00000	1.00000	0.99859
GO:0007512~adult heart development	2	2.94	0.04465	APLNR, TCAP	64	14	19349	43.18973	1.00000	1.00000	0.99859
