# Supplementary material for: Soy-Derived Equol Induces Antioxidant Activity in Zebrafish in an Nrf2-Independent Manner
Source: Int J Mol Sci. 2022 May 8;23(9):5243. doi: 10.3390/ijms23095243 (PMC9105299; doi:10.3390/ijms23095243)
Supplement: Supplementary file 1 [file ijms-23-05243-s001.zip › ijms22.watanabe.TableS5.pdf]

Table S5. Biological processes downregulated by equol treatment.

Term	Count	%	P Value	Genes	List Total	Pop Hits	Pop Total	Fold Enrichment	Bonferroni	Benjamini	FDR
GO:0007601~visual perception	11	10.185	9.33E-08	RDH8, ABCA4, CABP2, CNGA1, RDH5, CNGB3, LRAT, CNGA3, OPN5, RLBP1, RPE65	99	206	19349	10.43635	5.96E-05	5.96E-05	5.93E-05
GO:0006776~vitamin A metabolic process	4	3.7037	4.35E-06	RBP1, LRAT, RLBP1, RPE65	99	7	19349	111.68254	0.00277	0.00139	0.00138
GO:0001523~retinoid metabolic process	6	5.5556	1.81E-05	ABCA4, RBP1, RDH5, LRAT, RLBP1, RPE65	99	64	19349	18.32292	0.01150	0.00385	0.00384
GO:0042572~retinol metabolic process	5	4.6296	1.39E-04	RDH8, RBP1, RDH5, LRAT, RPE65	99	52	19349	18.79274	0.08501	0.02221	0.02210
GO:0006814~sodium ion transport	5	4.6296	0.00109	SLC13A3, SLC5A8, SLC5A9, SLC12A1, SLC5A11	99	89	19349	10.98002	0.50084	0.11941	0.11885
GO:0007605~sensory perception of sound	6	5.5556	0.00112	CDKN2D, TMIE, SLC52A3, CABP2, MYO7B, CRYM	99	154	19349	7.61472	0.51172	0.11941	0.11885
GO:0009617~response to bacterium	5	4.6296	0.00245	VIL1, C3, FABP4, ADIPOQ, LRAT	99	111	19349	8.80380	0.79135	0.22360	0.22255
GO:0050896~response to stimulus	4	3.7037	0.00606	RDH8, CNGA1, RDH5, RLBP1	99	73	19349	10.70928	0.97949	0.48437	0.48210
GO:0050873~brown fat cell differentiation	3	2.7778	0.01073	FABP4, MB, ADIPOQ	99	31	19349	18.91398	0.99899	0.68572	0.68250
GO:0006694~steroid biosynthetic process	3	2.7778	0.01073	RDH8, FDXR, LSS	99	31	19349	18.91398	0.99899	0.68572	0.68250
GO:0042632~cholesterol homeostasis	4	3.7037	0.01507	ABCG5, FABP4, NR5A2, ABCB11	99	102	19349	7.66449	0.99994	0.87551	0.87140
GO:0032526~response to retinoic acid	3	2.7778	0.02960	CDKN2D, PTCH1, LRAT	99	53	19349	11.06289	1.00000	1.00000	0.99687
GO:0043687~post-translational protein modification	6	5.5556	0.03098	KLHL9, C3, ASB10, ASB14, MELTF, MATN3	99	345	19349	3.39903	1.00000	1.00000	0.99687
GO:0055088~lipid homeostasis	3	2.7778	0.03169	RBP1, ABCB11, TM6SF2	99	55	19349	10.66061	1.00000	1.00000	0.99687
GO:0032532~regulation of microvillus length	2	1.8519	0.03493	VIL1, CDHR5	99	7	19349	55.84127	1.00000	1.00000	0.99687
